# Autoinhibitory feedback preserves intestinal stem cell maintenance and fate commitment

**DOI:** 10.1038/s44318-026-00808-x

**Published:** 2026-05-20

**Authors:** Siamak Redhai, Nick Hirschmüller, Tianyu Wang, Tyler Jackson, Stefan Peidli, Erica Valentini, Shivohum Bahuguna, Svenja Leible, Sarina Möller, Christina Ko, Michaela Holzem, Sviatoslav Kharuk, Lea Bräckow, Fillip Port, David Ibberson, Hongjie Le, Wolfgang Huber, Michael Boutros

**Affiliations:** 1 German Cancer Research Center (DKFZ), Division Signaling and Functional Genomics, Heidelberg, Germany; 2 Heidelberg University, Institute of Human Genetics, Medical Faculty Heidelberg, Heidelberg, Germany; 3 Heidelberg University, Department of Cell and Molecular Biology, Medical Faculty Mannheim & BioQuant, Heidelberg, Germany; 4 European Molecular Biology Laboratory (EMBL), Meyerhofstraße 1, Heidelberg, Germany; 5 Huffington Center on Aging & Department of Molecular and Human Genetics, Baylor College of Medicine, Houston, TX USA; 6 BioSciences Department, Rice University, Houston, TX USA; 7 Department of Biochemistry and Biotechnology, Vasyl Stefanyk Precarpathian National University, Ivano-Frankivsk, Ukraine; 8 Cellnetworks Core Technology Platform (CCTP), Deep Sequencing Labor, Heidelberg, Germany; 9 Present Address: Cardiovascular Epidemiology Unit, Department of Public Health and Primary Care, University of Cambridge, Cambridge, UK

**Keywords:** Chromatin, Transcription & Genomics, Development, Signal Transduction

## Abstract

Intestinal stem cells (ISCs) continuously renew the gut epithelium by producing specialised cell types, yet the mechanisms that couple ISC renewal with lineage commitment remain poorly characterised. Here, we identify a self-limiting transcriptional program, mediated by the zinc-finger transcription factor *Chronophage* (*Cph*), that promotes both ISC maintenance and differentiation into enteroendocrine (EE) cells in the *Drosophila* midgut. *Cph* expression is transiently induced by the proneural factor *scute* at the onset of ISC-to-EE specification. Genetic and single-cell transcriptomic approaches revealed that *Cph* is required to reprogramme ISCs and sustain normal lifespan. Cph binds to genes involved in proliferation and differentiation, and directly represses its own expression. This autoinhibitory feedback safeguards ISCs from accumulating autophagosomes and undergoing cell death, thus preserving ISC function. Our findings uncover a key regulatory mechanism that balances stem cell maintenance and differentiation, highlighting principles relevant to regenerating tissues.

## Synopsis

The mechanisms that balance stem cell renewal with lineage commitment remain poorly characterized. This study identifies an autoinhibitory feedback mechanism mediated by the transcription factor Chronophage (*Cph*) that promotes enteroendocrine cell (EE) specification while preventing autophagosome accumulation and cell death in intestinal stem cells (ISCs) of the *Drosophila* midgut.

- *Cph* expression is induced in the course of EE differentiation from ISCs during low Notch signaling.
- *Cph* transcriptionally reprograms ISCs, while sustaining their self-renewal and organismal lifespan.
- Genome-wide characterization of transcription factor binding sites identifies *Cph* binding to genes linked to lineage commitment and cell cycle.
- Autoinhibition of *Cph* safeguards ISCs against autophagosome accumulation and cell death.

## Abstract (web summary)

Chronophage promotes enteroendocrine cell differentiation in the *Drosophila* midgut, while preventing intestinal stem cell death and maintaining organismal lifespan.

## Graphical abstract

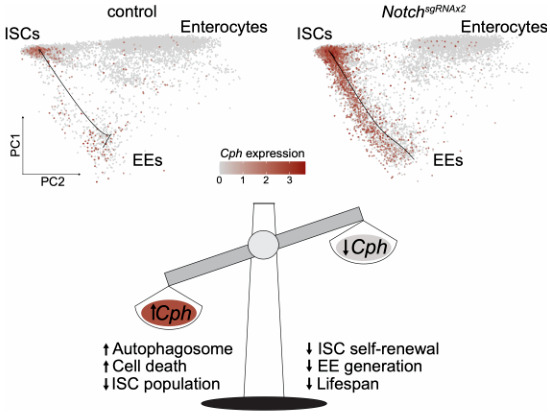

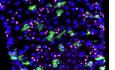

## Introduction

Replenishment of epithelial cells in adult tissue is central for homeostatic balance (Biteau et al, 2011). The intestinal epithelium consists of spatially diverse cell types which are derived from multipotent intestinal stem cells (ISCs). The process of differentiating into lineage-specific cell types depends on various processes, including asymmetric cell division, detachment from the ISC niche, mechanical regulation, transcription factor and signalling dynamics (Carley et al, 2022; Ferraro et al, 2010; Yamashita et al, 2010). Such mechanisms ensure that turnover of epithelial cells is faithfully coordinated during environmental stress, tissue damage, and to maintain homeostasis. The majority of the intestinal epithelium consists of enterocytes (ECs), which are primarily responsible for absorptive functions (Kiela and Ghishan, 2016). This is in contrast to the neuropeptide-secreting enteroendocrine cells (EEs), which comprise a small proportion of epithelial cells that regulate endocrine processes, including food intake, appetite, gut motility and metabolism (Guo et al, 2022a; Sanchez et al, 2022).

Due to many biologically conserved processes between flies and mammals, the *Drosophila melanogaster* midgut has proven to be an excellent model for understanding ISC fate determination (Apidianakis and Rahme, 2011; Casali and Batlle, 2009; Medina et al, 2022). The fly contains self-renewing multipotent ISCs that are scattered along the midgut and give rise to enteroblast (EBs) and enteroendocrine progenitors (EEPs), which terminally differentiate into ECs and EEs, respectively (Micchelli and Perrimon, 2006; Ohlstein and Spradling, 2006; Zeng and Hou, 2015). Recent studies have identified 10 subpopulations of EEs that are spatially confined to specific regions of the midgut (Guo et al, 2019; Guo et al, 2022b; Hung et al, 2020). These subpopulations are broadly categorised into two mutually exclusive groups according to. the expression of neuropeptides: class I: Allatostatin C^+^ (AstC) cells and class II: Tachykinin^+^ (Tk) cells. A third class has also been reported recently which are AstC and Tk negative and found predominantly in the anterior midgut. Notch signalling plays an important role in determining the fate of ISCs, favouring ECs when Notch activity is high in ISCs, and EEs when Notch activity is low in ISCs (Fre et al, 2005; Guo and Ohlstein, 2015; Ohlstein and Spradling, 2007; van Es et al, 2005). Inactivation of the Notch pathway in ISCs results in excessive proliferation and accelerates the formation of AstC^+^ EEs, leading to neuroendocrine tumour-like structures that develop mainly in the posterior midgut and are detrimental to lifespan (Beehler-Evans and Micchelli, 2015; Patel et al, 2015).

Previous studies highlighted a number of transcription factors (TFs) that regulate commitment to an EE fate. For example, Notch signalling negatively regulates the expression of the Achaete-Scute Complex (AS-C) basic helix–loop–helix TF *scute* (*sc*) (Bardin et al, 2010; Chen et al, 2018). When Notch signalling is low in ISCs, *sc* is activated through a transcriptional self-stimulatory loop, which results in asymmetric cell division and terminal differentiation of ISCs into EEs (Chen et al, 2018), a process that is regulated by the homeobox TF *prospero* (*pros*), which governs the differentiation programme in EEs (Guo et al, 2022b). Upstream TFs such as *klumpfuss* (*klu*) normally suppress *sc* expression and regulate apoptosis to drive EC lineage commitment (Korzelius et al, 2019; Reiff et al, 2019). Similarly, the TF *tramtrack* (*ttk*) also functions as a negative regulator of *sc* expression (Wang et al, 2015). The Ttk protein is kept in check by the E3 ubiquitin ligase Seven in Absentia (Sina) and the adaptor protein Phyllopod (Phyl) which work together to ubiquitinate Ttk, leading to its degradation by the proteasome (Yin and Xi, 2018). Despite these findings, the temporal dynamics of TF expression and their involvement in determining the EE lineage in specific regions of the intestine remain poorly understood.

In this study, we profiled the transcriptome of 50,067 single cells from the adult *Drosophila* midgut during steady state and under multiple perturbed conditions involving *Notch* and the C2H2 zinc-finger TF *Chronophage* (*Cph*), which we identify as a crucial regulator of progenitor cell proliferation and EE fate commitment. *Cph* expression is induced early along the ISC-to-EE lineage when Notch activity is diminished and is required for generating EEs. We show that *sc* binds directly to the *Cph* locus and promotes its expression. Single-cell RNA-sequencing in perturbed and co-perturbed conditions revealed that *Cph* is required for reprogramming ISCs and EEPs when Notch is depleted. By characterising *Cph* DNA-binding profiles in vivo, we identified a number of key target genes that are required for ISC proliferation and lineage commitment. Importantly, we found that Cph represses its own expression to guard against increased abundance of autophagosomes and cell death and ensure differentiation is faithfully executed. Our findings therefore highlight a previously uncharacterised autoinhibitory mechanism centred on *Cph* that tunes intestinal epithelial identity.

## Results

### scRNA-seq of *Notch* mutant intestinal cells reveals major changes in cell type composition

**Figure 1:**
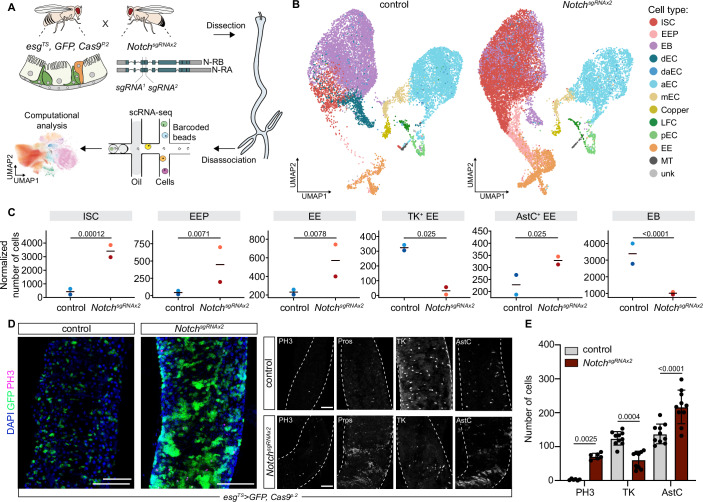
scRNA-seq of intestinal cells reveals major changes in cell type composition upon Notch inactivation. (**A**) Schematic of single-cell RNA-sequencing experimental workflow. CRISPR mutagenesis of *Notch* in progenitor cells (green) was achieved by expressing two *sgRNA* targeting the *Notch* locus with the *esg*^*TS*^*, GFP, Cas9*^*p.2*^ system. An Enteroendocrine cell is coloured in orange. (**B**) Uniform Manifold Approximation and Projection (UMAP) plot of the integrated scRNA-seq dataset for the control condition and *Notch* perturbed condition. (**C**) Quantification of cell type abundance coloured according to scRNA-seq replicates. (**D**) In vivo CRISPR mutagenesis of *Notch* in progenitor cells results in an expansion of the progenitor population, an increase in the number of PH3^+^ mitotically active cells, an increase in AstC^+^ EEs and a decrease in TK^+^ EEs. Note that immunostaining for grey panels was obtained from separate midguts. Scale bar 100 μm. (**E**) Quantification of different cell types in control and *Notch* mutant conditions, either in the entire midgut (PH3) or within the posterior region (TK and AstC). Two independent replicates of scRNA-seq were done on 20 flies per condition for (**B**, **C**). For (**E**), a minimum of three independent replicates were performed with the following sample sizes from left to right: *n* = 6, 6, 10, 9, 10, 10. Error bars indicate SEM. One-way ANOVA test with Tukey post hoc comparison was used for (**E**), while DESeq2 was used for (**C**). Source data are available online for this figure.

**Figure EV1:**
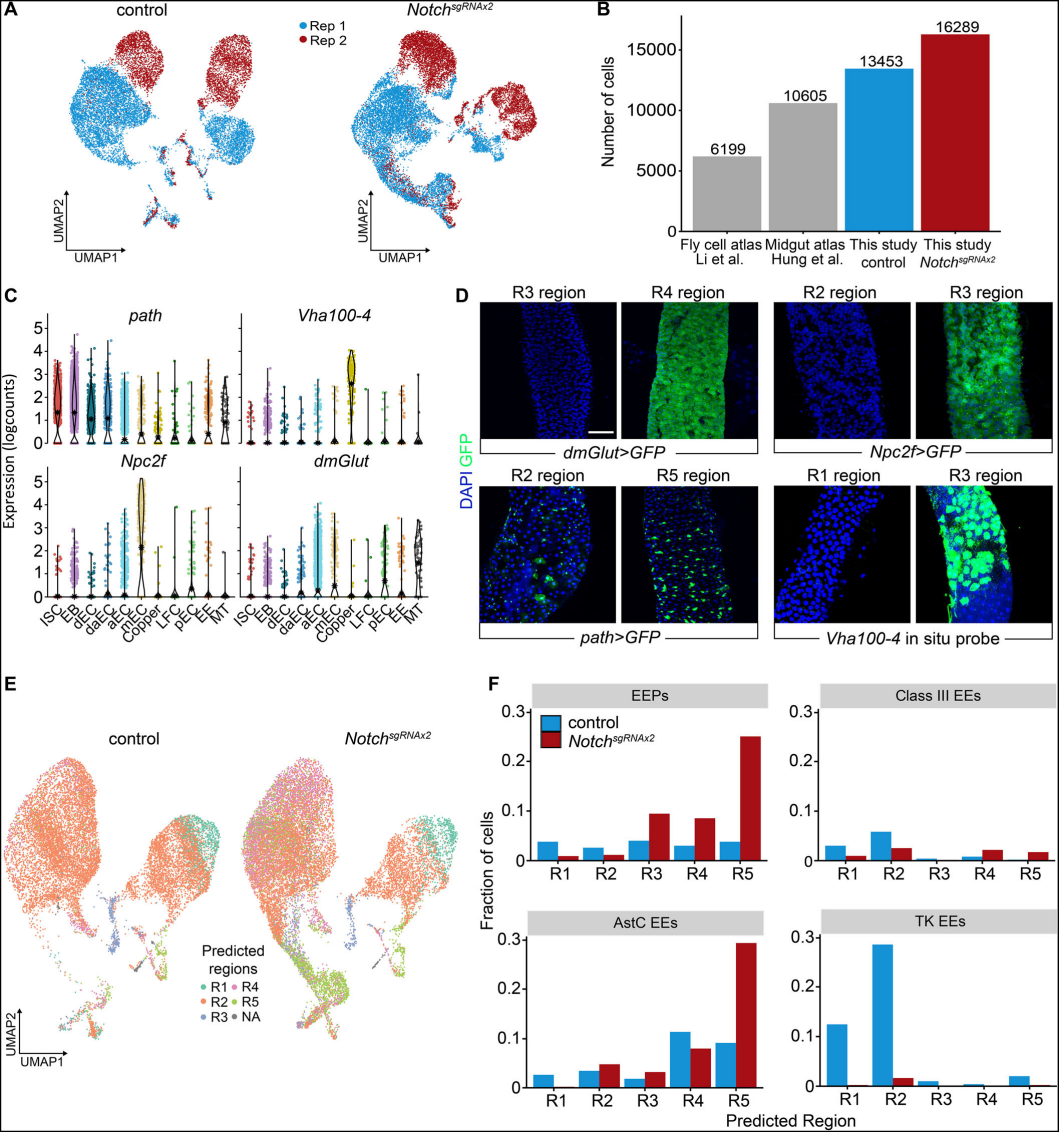
Quality control, marker gene expression and regional scRNA-seq dataset, related to Fig. 1. (**A**) UMAP uncorrected for batch effects for each scRNA-seq replicate. (**B**) The number of cells recovered after QC from this study compared to two other studies. (**C**) Violin plot of genes with interesting expression profiles in different intestinal cell types. (**D**) Validation of marker genes in different intestinal regions in vivo, related to (**C**). Scale bar 100 μm. (**E**) UMAP of the regional prediction of cell types from the control and *Notch* mutant conditions. (**F**) Regional predictions for EEs and their subclasses in control and *Notch* mutant conditions. For all images, nuclei are labelled with DAPI. For (**C**), expression profiles are shown from two independent replicates of scRNA-seq with a sample size of 20 flies.

To explore how different intestinal cell types respond to impairment of Notch signalling, we performed single-cell RNA sequencing (scRNA-seq) of the adult *Drosophila* midgut at homoeostatic condition and under progenitor-specific CRISPR mutagenesis of the Notch receptor with two single guide RNA simultaneously (*sgRNA*^*x2*^) (herein referred to as *Notch*^*sgRNAx2*^) (Port et al, 2020) using the *esg*^*TS*^ driver system (*esg-Gal4, tub-Gal80*^*TS*^ > *GFP, Cas9*^*p.2*^) (Fig. 1A). The *esg*^*TS*^ driver permits temporally controlled expression of transgenes, including GFP, specifically within the progenitor population of the midgut. With each condition assayed in duplicate, we obtained 29,741 high-quality single-cell gene expression profiles (control: 13,452 cells; *Notch*^*sgRNAx2*^: 16,289 cells), surpassing previously published scRNA-seq datasets for the *Drosophila* midgut (Hung et al, 2020; Li et al, 2022) (Fig. EV1A,B). To facilitate community use, we provide a Shiny app to enable interactive visualisation and exploration of our scRNA-seq dataset: https://shiny-portal.embl.de/shinyapps/app/16_IntestiMap

We clustered the cells’ transcriptomic profiles, taking guidance from previous cell type catalogues (Hung et al, 2020; Li et al, 2022) and manually categorised our data into ten major cell types, including ISCs, EBs, EEPs, ECs, and EEs and their respective subtypes (Fig. 1B). We validated cluster-specific marker genes in vivo. For instance, in situ probes against *Vha100-a* identified specifically the copper cell population in the middle midgut, while specific *Gal4* enhancers confirmed *Npcf2* and *dmGlut* expression in various EC populations (Fig. EV1C,D). We also found that the amino acid transporter *path* marked ISCs, suggesting a potential requirement for nutrient sensing in this cell type (Fig. EV1C,D), as previously described (Hung et al, 2020). Moreover, by mapping bulk regional expression profiles from dissected midguts (Dutta et al, 2015), we inferred the spatial coordinates of all major cell types (Fig. EV1E,F). In conclusion, our dataset under homeostatic conditions provides a comprehensive single-cell atlas of the intestine, offering insights into the regional and functional properties of this organ and serves as a reference map to compare the effects of genetic perturbations.

Next, we quantified the abundance of cell types during homeostasis and under progenitor-specific *Notch*^*sgRNAx2*^ mutagenesis. We observed a drastic expansion of Dl^+^ ISCs, pros^+^ EEs and pros^+^/hdc^+^/esg^+^/Dl^+^ EEPs when *Notch* was deactivated in progenitor cells, especially in the R3 to R5 regions (Figs. 1C and EV1E,F). We verified these findings in vivo by observing an increase in ISC proliferation and EE generation in progenitor-specific *Notch* mutant flies (Fig. 1D,E). We also found that the EB population significantly decreased and there was a trend towards a smaller EC population, providing further evidence that loss of Notch activity in the progenitor population causes major changes in cell type composition (Fig. 1C).

After observing major changes in the EE populations, we sought to explore this in more detail by subcategorising EEs based on the expression of different neuropeptides and their spatial coordinates. We found that loss of Notch signalling drastically increased the class I AstC^+^ EEs in the R5 region, while simultaneously decreasing class II Tk^+^ EEs in the same region (Figs. 1C and EV1F). There was no change in the fraction of total class III EEs that were positive for CCHa2, but variation across different regions was observed (Fig. EV1F). We validated these findings in vivo, by performing immunostaining for AstC^+^ and TK^+^ EEs and confirmed that inactivation of Notch signalling in progenitor cells increased the production of class I AstC^+^ EEs at the expense of class II Tk^+^ EEs (Fig. 1D,E).

### Notch inactivation causes significant cell-type-specific transcriptional changes

**Figure EV2:**
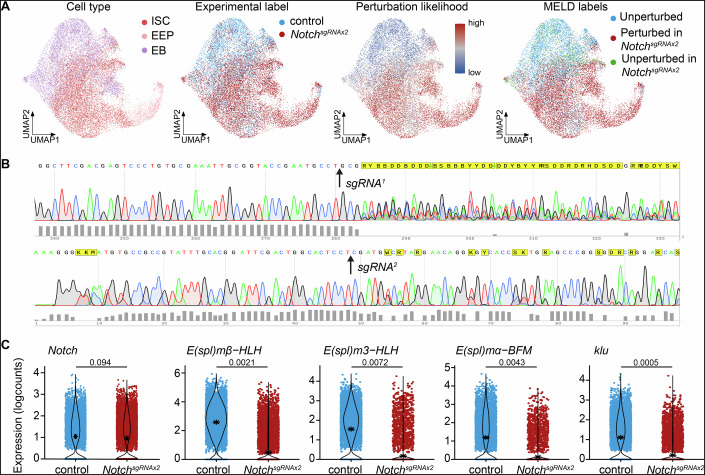
Identification of perturbed cells using MELD, related to Fig. 2. (**A**) UMAPs of progenitor cells arranged by cell type, experimental labels and perturbation likelihood, which were used to determine MELD labels. (**B**) Sanger sequencing traces of progenitor-specific *Notch* mutant intestine, highlighting where *sgRNA* starts. (**C**) Expression of Notch and various *Notch* target genes in control and *Notch* mutant conditions. For all comparisons, an asterisk denotes the mean. For (**C**), two independent replicates of scRNA-seq were performed for each condition with a sample size of 20 flies per condition. Statistical test for (**C**) was calculated using the pseudobulk approach and edgeR.

CRISPR mutagenesis in cell populations often gives rise to genetic mosaics that contain cells with functional alleles of the target gene. To assess the efficiency of *Notch*^*sgRNAx2*^ CRISPR mutagenesis in progenitor cells at single-cell resolution, we used MELD along with vertex frequency clustering (VFC) (Burkhardt et al, 2021). This method aims to find changes in the probability density of cell states between the control and perturbed conditions to identify cell populations affected by the perturbation. Using MELD on the *Notch*^*sgRNAx2*^ perturbed dataset, we observed that 89.4% of progenitor cells were assigned as perturbed and 10.6% as unperturbed (Fig. EV2A). We confirmed CRISPR editing at both *sgRNA* targeting locations using Sanger sequencing (Fig. EV2B). Interestingly, we detected no change in the mRNA expression level of mutant *Notch* in the perturbed cells, an observation that suggests CRISPR-induced edits impact Notch protein function rather than mRNA levels (Fig. EV2C). To assess the state of Notch signalling, we characterised previously validated target genes that report on Notch activity using our scRNA-seq dataset, including *E(spl)mα-BFM*, *E(spl)m3-HLH*, *E(spl)mβ-HLH* and *klu*. All tested target genes showed a significant reduction in their expression in the *Notch*^*sgRNAx2*^ perturbed progenitor population (Fig. EV2C). These data indicate that expression of *Notch*^*sgRNAx2*^ results in the loss of Notch activity in the majority of progenitor cells.

**Figure EV3:**
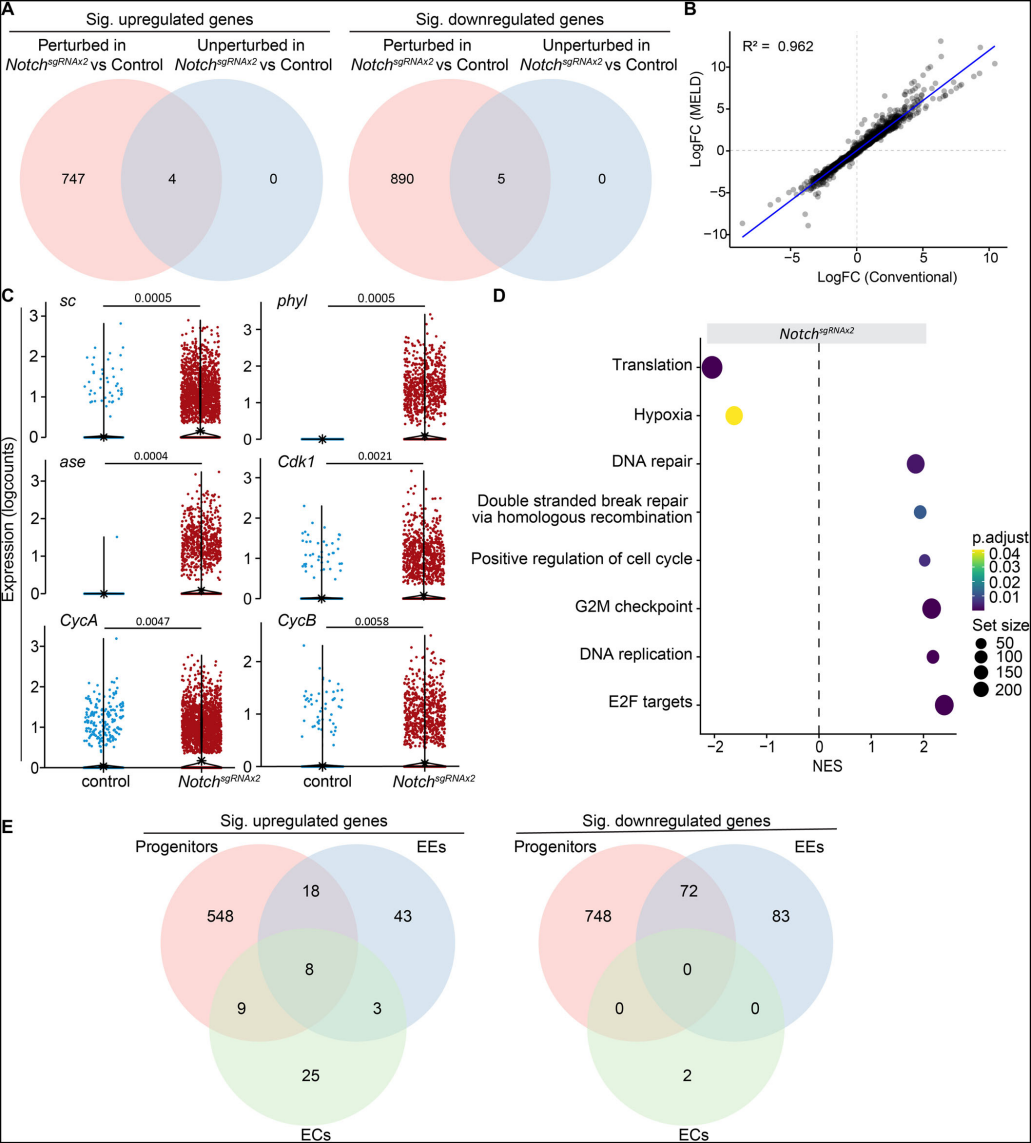
Identification of DEGs and gene set enrichment analysis, related to Fig. 2. (**A**) Number of up- and downregulated DEGs in perturbed and unperturbed progenitor cells compared to the control condition. (**B**) Correlation coefficient of DEGs identified by MELD and the conventional method (see “Methods”). (**C**) Expression of various genes in control and perturbed *Notch* mutant progenitor cells. (**D**) Gene set enrichment analysis for *Notch*^*sgRNAx2*^ condition. (**E**) Number of DEGs that are up- and downregulated in different intestinal cell types. For (**C**), two independent replicates of scRNA-seq were performed for each condition, with a sample size of 20 flies per condition. Significance for (**C**) was calculated using the pseudobulk approach and edgeR, whereas for (**D**) clusterProfiler’s GSEA function was used to calculate adjusted significance.

To understand transcriptional changes in the progenitor population, we performed differential gene expression analysis by comparing perturbed to unperturbed cells as inferred by MELD. This analysis revealed 751 upregulated and 895 downregulated genes in the *Notch*^*sgRNAx2*^ perturbed progenitor population (Fig. EV3A). Similar results were obtained when directly comparing cells from the *Notch*^*sgRNAx2*^ condition with those from the control, disregarding the perturbation’s success, which highlights the perturbation efficiency (Fig. EV3B). We identified only nine differentially expressed genes (DEGs) in the unperturbed progenitor population from the *Notch*^*sgRNAx2*^ condition when compared to control, consistent with limited transcriptional changes in these cells (Fig. EV3A). In the perturbed population, we detected an increase in expression of a number of genes previously reported to be upregulated upon loss of *Notch*, including *scute*, *phyl*, and *asense*, and further identified genes related to the cell cycle, reflecting the increased mitosis observed in vivo (Fig. EV3C). Progenitor-specific upregulated genes were significantly enriched in gene sets related to DNA replication, cell cycle and homologous recombination, while downregulated genes were associated with hypoxia and translation (Fig. EV3D). Interestingly, we found that E2F and Myc target genes were significantly increased in *Notch* perturbed progenitor cells, indicating that this may be the main driver of proliferation in progenitor cells (Fig. EV3D). Since CRISPR mutations are heritable after differentiation, we investigated how *Notch* mutation elicits transcriptional changes in all major intestinal cell types. We identified that the majority of DEGs are specific to one cell type, demonstrating that *Notch* mutations result in cell-type-specific transcriptional responses (Fig. EV3E). Moreover, the majority of DEGs were identified in progenitors and EEs, indicating that these cell types are particularly sensitive to the loss of Notch signalling (Fig. EV3E).

### Cph expression along the ISC-EEP-EE lineage is negatively regulated by Notch signalling

**Figure EV4:**
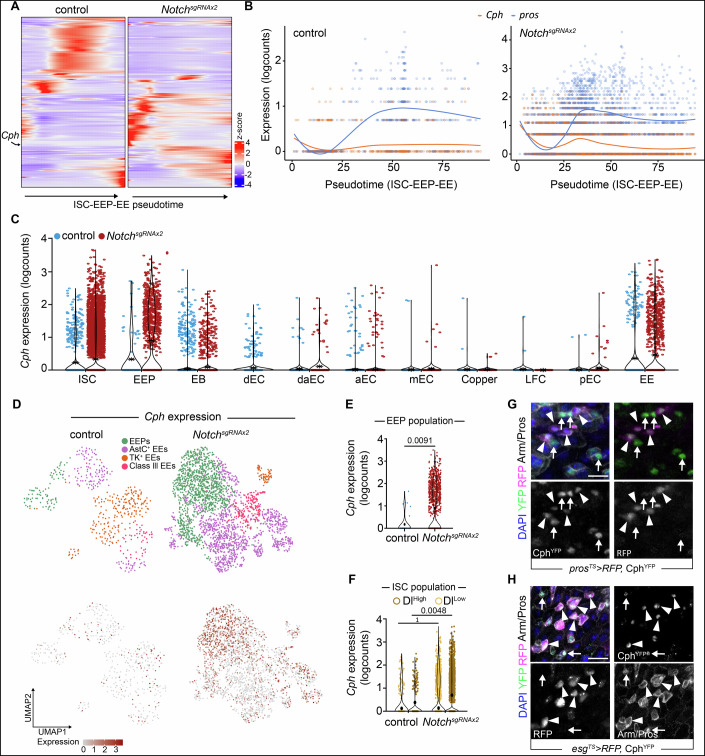
*Cph* expression across intestinal cell types and lineages, related to Fig. 2. (**A**) Pseudotime expression analysis of all differentially expressed genes along the ISC-EEP-EE lineage under control and *Notch* mutant conditions. (**B**) Expression of *Cph* and *pros* during ISC-EEP-EE differentiation in the homoeostatic condition and *Notch* mutant condition. (**C**) Expression of *Cph* in all intestinal cell types during homeostasis or *Notch* mutant conditions. (**D**) *Cph* expression in the EEP and EE population in control and *Notch* mutant conditions. *Cph* is present in some EEs during homeostasis, and its expression increases mainly in the EEP population. (**E**) Quantification of *Cph* expression specifically within the EEP population under control and *Notch* mutant condition. (**F**) Log counts for *Cph* expression within ISCs that are sub-clustered based on Dl^High^ and Dl^Low^ expression, highlighting that *Cph* expression increases in Dl^High^ ISCs when Notch signalling is perturbed. (**G**) *RFP* expression using the *Pros*^*TS*^ driver overlaps with endogenously tagged Cph^YFP^ (arrowheads). Cph^YFP^ is also expressed in non-RFP cells that represent ISCs (arrows). Scale bar 20 μm. (**H**) Cph^YFP^ is found in some progenitor cells (arrowheads) and EEs (arrows) in the male midgut. For all images, nuclei are labelled with DAPI. For (**C**–**E**), two independent replicates of scRNA-seq were performed for each condition, with a sample size of 20 flies per condition. Scale bar 20 μm. Seurat’s FindMarkers function with test.use = “MAST“ was used to calculate *P* value for (**E**, **F**).

**Figure 2:**
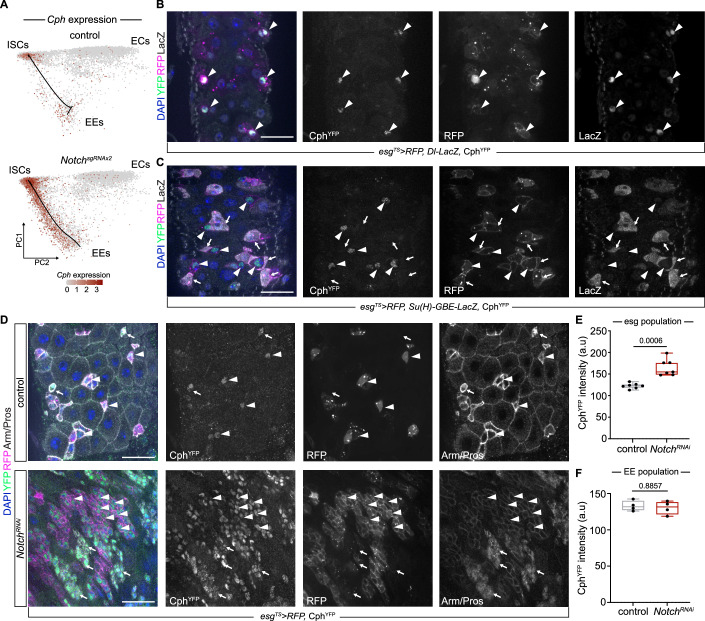
Notch signalling negatively regulates Cph along the ISC-EEP-EE lineage. (**A**) PCA projection displaying *Cph* expression in control and *Notch* mutant condition, showing that *Cph* expression increases along the ISC-EEP-EE lineage when *Notch* is inactivated. (**B**, **C**) β-gal staining of midgut demonstrated that Cph is expressed specifically in Delta-LacZ^+^ ISCs (B, arrowheads) and not Su(H)-GBE-LacZ^+^ EBs (C, arrows). Confocal images taken from the posterior midgut. Scale bar for (**B**, **C**) 100 μm. (**D**) Endogenous expression of Cph is present within RFP^+^ progenitor cells (arrowheads), as well as EEs (arrows) marked by Pros. Silencing Notch in progenitor cells increases Cph^YFP^ levels within RFP^+^ progenitor cells. Confocal images taken from the posterior midgut. Scale bar 100 μm. (**E**, **F**) Quantification of Cph^YFP^ intensity within either the progenitor population (**E**) or EE population (**F**). For (**E**, **F**), two independent replicates were done with the following sample sizes from left to right: (**E**) *n* = 7, 7 and (**F**) *n* = 4, 4. Box plots: line, median; box, 75th–25th percentiles; whiskers, minimum to maximum. For all images, nuclei are labelled with DAPI. Mann–Whitney test was used to calculate the *P* value for (**E**, **F**). Source data are available online for this figure.

Since we observed major changes in the abundance of EEs upon expression of *Notch*^*sgRNAx2*^ in progenitor cells, we performed differential gene expression analysis along the ISC-EEP-EE differentiation trajectory and identified 179 deregulated genes, of which 12 were TFs (Fig. EV4A). One particular TF that had a distinct expression profile was the C2H2 zinc-finger TF Chronophage (*Cph -* CG9650) (Fig. EV4A). *Cph* is homologous to mammalian *BCL11a* and *BCL11b*, which are implicated in acute myeloid leukaemia (AML) (Sunami et al, 2022), haemoglobin switching(Liu et al, 2003; Sankaran et al, 2009) and intellectual disability disorder (Seigfried and Britsch, 2024). Recently, *BCL11b* was shown to enhance Wnt signalling and promote injury-induced regeneration in the intestine (Li et al, 2024). Genetic screens in the past have implicated *Cph* in regulating Notch signalling in the developing *Drosophila* eye (Shalaby et al, 2009). During development, *Cph* regulates the timing of neuronal stem cell differentiation (Fox et al, 2022) and locomotor behaviour (Murthy and Nongthomba, 2024), but its functions in adult somatic stem cells are yet to be described. Under control conditions, *Cph* expression was largely restricted to ISCs and EEs, and not detected in EBs (Fig. 2A). Upon loss of Notch signalling, *Cph* expression increased specifically in ISCs and EEPs, without induction along the ISC–EB–EC lineage (Figs. 2A and EV4B,C). Next, we sought to investigate the characteristics of *Cph*-expressing cells. We observed that loss of Notch signalling induces *Cph* expression in Dl^+^ Pros^+^ immature EEPs but not IA-2^+^ Pros^+^ mature EEs expressing AstC or Tk (Figs. 2A and EV4C–E). By grouping ISCs into either *Dl*^high^ and *Dl*^low^ populations, we observed that the expression of *Cph* was largely in *Dl*^high^ ISCs and increased specifically in this population upon loss of Notch signalling (Fig. EV4F). Using an endogenously tagged Cph^YFP^ protein trap line, we confirmed Cph^YFP^ expression in Dl^+^ ISCs and Pros^+^ EEs both in males and females (Figs. 2B,C and EV4G,H). Importantly, Cph^YFP^ was absent in Su(H)-GBE^+^ EBs. Inactivation of Notch signalling increased Cph^YFP^ intensity in progenitor cells and not EEs, demonstrating that the increased expression of *Cph* under low Notch signalling conditions translates to increased protein production (Fig. 2D–F). Moreover, there was an expansion of both Cph^YFP^-ISCs and Cph^YFP^-EEs when Notch signalling was perturbed (Fig. 2D). Thus, *Cph* expression is induced early along the ISC-EEP-EE lineage when Notch signalling is low.

### Cph is required during low Notch signalling to maintain epithelial turnover, EE identity and normal lifespan

**Figure 3:**
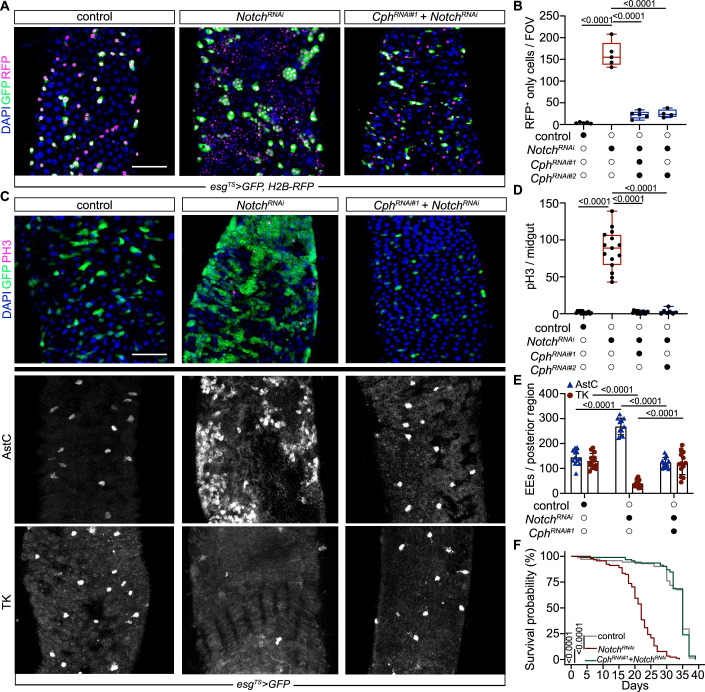
Cph maintains and specifies ISCs during low Notch signalling. (**A**) Silencing Notch with the ReDDM system increases both GFP + /RFP+ cells and RFP-only cells. Co-silencing Cph and Notch reduces the excess GFP + /RFP+ and RFP-only cells observed with Notch silencing only. Scale bar 100 μm. (**B**) Quantification of the number of RFP-only cells in the posterior midgut. (**C**) Genetic interaction between Cph and Notch assessed through changes in progenitor populations, AstC+ enteroendocrine cells, and TK+ enteroendocrine cells. Note that immunostaining for grey panels were obtained from separate midguts. Scale bar 100 μm. (**D**) Quantification of mitotic cells throughout the midgut. (**E**) Quantification of different populations of EEs within the posterior region. (**F**) Quantification of lifespan. For (**B**, **D**, **E**), a minimum of three independent replicates were done with the following sample sizes from left to right: (**B**) *n* = 5, 5, 5, 4; (**D**) *n* = 13, 15, 15, 7; (**E**) *n* = 12, 13, 11, 13, 13, 13. For all images, nuclei are labelled with DAPI. Box plots: line, median; box, 75th–25th percentiles; whiskers, minimum to maximum. Error bars indicate SEM (**E**). One-way ANOVA test with Tukey post hoc comparison was used for all graphs, except for (**F**) where a logrank test was used for lifespan. Source data are available online for this figure.

**Figure EV5:**
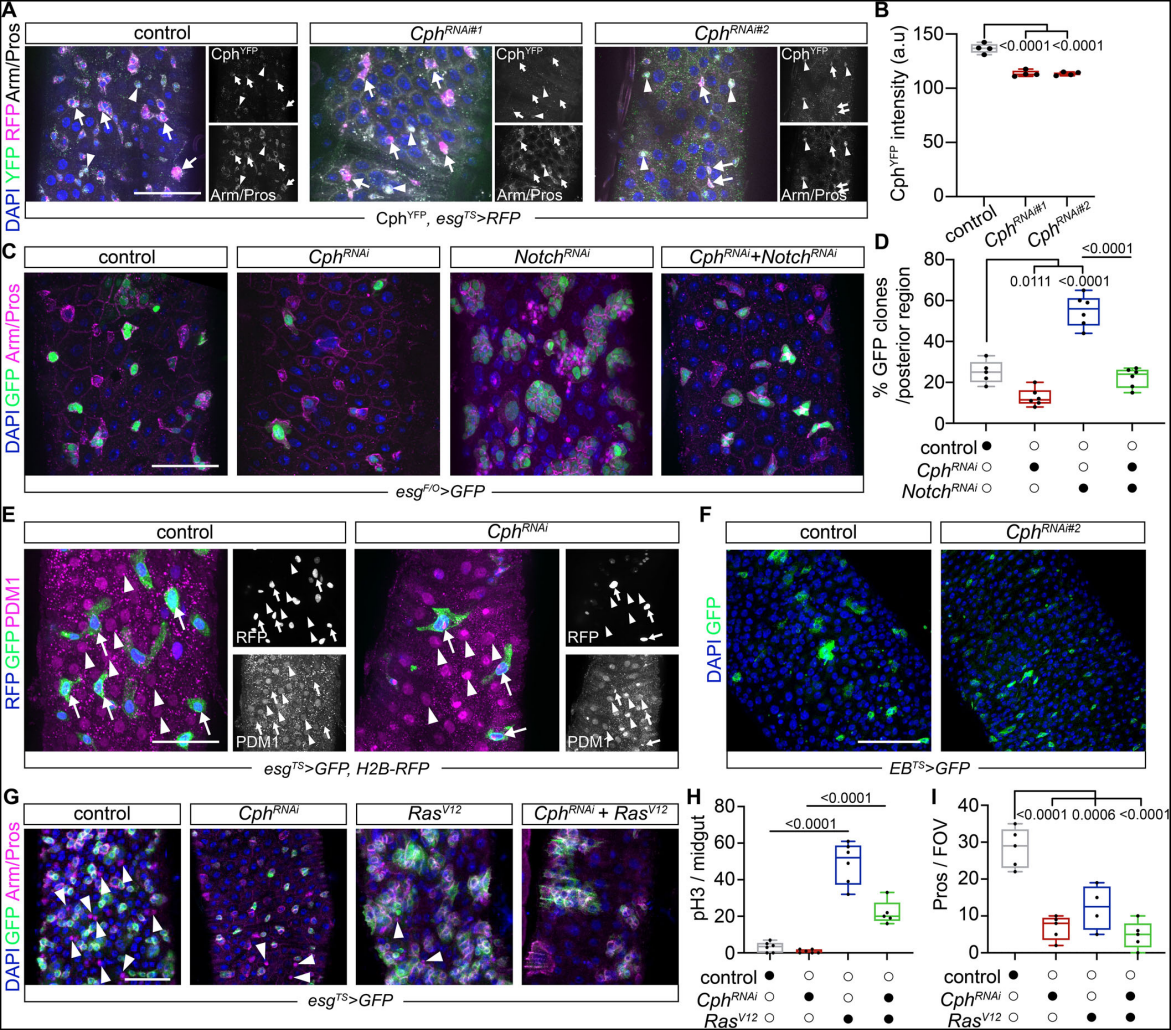
Functional characterisation of Cph, related to Figs. 3 and 4. (**A**) Silencing Cph in progenitor cells decreases the intensity of Cph^YFP^ in progenitor cells (arrows) and not in EE (arrowheads). Scale bar 50 μm. (**B**) Quantification of Cph^YFP^ in RFP^+^ progenitor cells. (**C**) Confocal images of Flip-out clones within the posterior midgut under different genetic conditions. Scale bar 100 μm. (**D**) Quantification of the number of GFP clones within the posterior midgut. (**E**) REDDM lineage tracing highlighted that *Cph* depletion causes some progenitor cells to become abnormally large and contain nuclear PDM1. Scale bar 100 μm. (**F**) Confocal images of the posterior midgut when Cph is silenced within EBs. Scale bar 100 μm. (**G**) Confocal images of the posterior midgut under different genetic conditions. Arrowheads point to Pros^+^ enteroendocrine cells. Scale bar 100 μm. (**H**) Quantification of the number of pH3+ mitotically active cells in the midgut. Scale bar 100 μm. (**I**) Quantification of the number of Pros+ enteroendocrine cells in the field of view (FOV). For (**B**), intensity measurements for each condition were taken from one matched experiment, For (**D**), clones for each condition were quantified from one matched experiment, For (**H**, **I**), two independent replicates were done with the following sample size from left to right: (**B**) *n* = 4, 4, 4; (**D**) *n* = 5, 6, 6, 6; (**H**) *n* = 6, 6, 6, 5; (**I**) *n* = 5, 5, 4, 5. For all images, nuclei are labelled with DAPI. Box plots: line, median; box, 75th–25th percentiles; whiskers, minimum to maximum. One-way ANOVA test with Tukey post hoc comparison was used for statistical analysis of all graphs.

Since we observed that *Cph* expression significantly increased in *Notch* mutant conditions, we sought to find out whether silencing *Cph* could rescue the excessive proliferation and differentiation defects associated with inactivating Notch signalling in progenitor cells. To investigate this, we first used the ReDDM lineage tracing system (Antonello et al, 2015), which labels progenitors with a short-lived mCD8-GFP and long-lived H2B-RFP and their differentiated cells in H2B-RFP-only. *RNAi*-mediated depletion of *Notch* resulted in a significant increase in GFP^+^/RFP^+^ progenitor cells as well as RFP^+^ only cells with small nuclei resembling EEs (Fig. 3A). Silencing *Cph* with two independent *RNAi* lines curbed the excessive progenitor proliferation and EE turnover when Notch signalling was perturbed, suggesting that *Cph* is required during low Notch activity to generate new ISCs and EEs (Fig. 3A,B). We validated that both *Cph*^*RNAi*^ lines reduced the level of Cph^YFP^ in progenitor cells, demonstrating their silencing efficiency (Fig. EV5A,B). Using an independent lineage tracing tool termed *esg*^*F/O*^, which uses temperature-dependent FLPase expression to constitutively activate an Act>STOP>Gal4 driver by removing the STOP cassette located between FRT sites (Jiang et al, 2009), we confirmed that loss of *Cph* decreased progenitor and EE cell turnover in the context of silencing *Notch* (Fig. EV5C,D).

Next, we examined the role of *Cph* in maintaining intestinal cell type identities when Notch signalling is inactivated. Immunostaining for enteroendocrine (EE) subtypes showed that silencing *Notch* in progenitor cells increased the number of mitotically active ISCs and AstC^+^ EEs while decreasing Tk^+^ EEs (Fig. 3C–E). This perturbation results in the formation of multi-layered tumour-like structures that consist predominantly of ISCs and EEs, which significantly decreased the survival of flies (Fig. 3F). Interestingly, conditional knockdown of both *Cph* and *Notch* decreased the number of progenitors that were mitotically active and restored both AstC^+^ EEs and Tk^+^ EEs back to wild-type levels (Fig. 3C–E). Importantly, we were also able to rescue the declining survival of *Notch*^*RNAi*^-expressing flies by co-expressing *Cph*^*RNAi*^ in progenitor cells (Fig. 3F). Therefore, *Cph* is crucial for maintaining the progenitor population, EE fate and is physiologically important for lifespan under low Notch signalling.

### Cph regulates stem cell maintenance and EE differentiation during homeostasis

**Figure 4:**
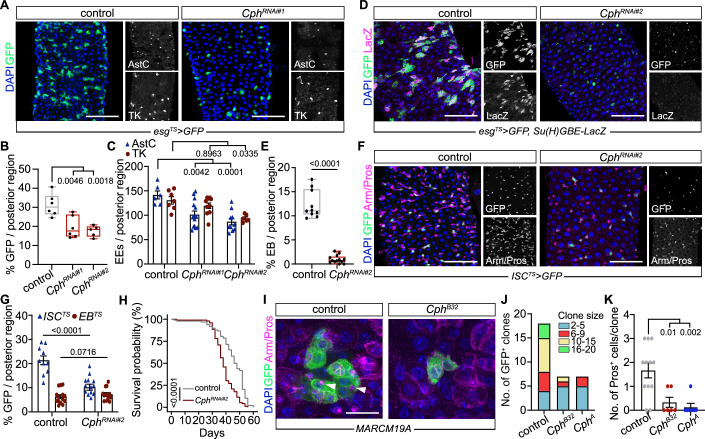
Cph regulates ISC maintenance and EE differentiation. (**A**) Confocal images of the posterior midgut showing that silencing Cph using the esg^TS^ system decreases the number of progenitor cells and AstC^+^ EEs. Note that immunostaining for grey panels was performed on separate midguts. Scale bar 100 μm. (**B**) Quantification of the number of GFP^+^ progenitor cells in the posterior midgut. (**C**) Quantification of the number of AstC^+^ and TK^+^ EEs in the posterior midgut. (**D**) β-gal staining of the midgut demonstrated that loss of Cph reduces the number of Su(H)-GBE^+^ EBs, indicating that cell cycle is arrested at the ISC stage. Scale bar 100 μm. (**E**) Quantification of the percentage of Su(H)-GBE^+^ EBs in the posterior midgut. (**F**) Confocal image of the posterior midgut highlighted that silencing Cph specifically within the ISC population decreases the number of GFP^+^ ISCs. Scale bar 100 μm. (**G**) Quantification of the number of GFP^+^ cells in the posterior midgut after silencing Cph in either ISCs (ISC^TS^) or EBs (EB^TS^). Note that loss of GFP^+^ cells only occurs once Cph is silenced within the ISC population. (**H**) Lifespan of adult flies, demonstrating that silencing Cph within stem cells using ISC^TS^, decreases survival. (**I**) MARCM clones of Cph null mutants are markedly smaller than control clones and contain few, if any, EEs (arrowheads). Scale bar 50 μm. (**J**) Quantification of the number of cells per clone. (**K**) Quantification of the number of EEs within medium clones (containing 6–15 cells). For (**B**, **C**, **G**, **K**), a minimum of three independent replicates were done, for (**E**) two independent replicates, with the following sample size from left to right: (**B**) *n* = 6, 6, 5; (**C**) *n* = 6, 7, 14, 10, 10, 6; (**E**) *n* = 10, 13; (**G**) *n* = 14, 14, 10, 13; (**K**) *n* = 12, 6, 7. For all images, nuclei are labelled with DAPI. Box plots: line, median; box, 75th–25th percentiles; whiskers, minimum to maximum. Error bars indicate SEM (**D**, **G**, **K**). One-way ANOVA test with Tukey post hoc comparison was used to calculate the *P* value for (**B**, **C**, **D**, **G**, **K**). Mann–Whitney test was used to calculate the *P* value for (**E**). The log-rank test was used for lifespan (**H**). Source data are available online for this figure.

Next, we investigated the function of *Cph* during homeostasis. We depleted *Cph* with two independent *RNAi* lines in progenitor cells and observed a marked decrease in the number of GFP^+^ progenitor cells and a reduction in the number of EEs, especially those containing the neuropeptide AstC (Fig. 4A–C). These results suggest that loss of *Cph* reduces the proliferative capacity of progenitor cells and their differentiation toward subclasses of EEs. To determine the stage at which progenitor differentiation is impaired, we examined *Su(H)GBE-LacZ*, a reporter that specifically marks EBs. Under control conditions, a fraction of GFP-positive progenitor cells co-expressed *Su(H)GBE-LacZ*, consistent with normal EB numbers (Fig. 4D,E). By contrast, silencing *Cph* in progenitor cells abolished *Su(H)GBE-LacZ* expression in the midgut, indicating that the cell cycle is arrested at the ISC stage (Fig. 4D,E). Using two independent lineage tracing systems, we observed that loss of *Cph* significantly reduced midgut epithelial turnover (Fig. EV5C,D). Immunostaining for PDM1, a marker of enterocytes (ECs), revealed that loss of *Cph* led to the accumulation of large progenitor-like cells containing nuclear PDM1 foci, a phenotype rarely observed under control conditions and suggestive of defects in differentiation (Fig. EV5E). These results indicate that loss of *Cph* results in ISC arrest and impacts their differentiation programme.

To map *Cph* function across progenitor cell types, we carried out cell-type-specific knockdown experiments. Silencing *Cph* in EBs did not alter EB numbers, whereas ISC-specific expression of *Cph*^*RNAi*^ led to a pronounced reduction in the ISC population (Figs. 4F,G and EV5F), suggesting defects in ISC maintenance. Furthermore, survival assays revealed that *Cph* is required for normal lifespan, as ISC-specific silencing of *Cph* significantly shortened the lifespan of flies (Fig. 4H). These findings indicate that *Cph* functions in ISCs rather than EBs, consistent with *Cph* being predominantly expressed in ISCs. To examine how proliferation and differentiation are linked, we used the MARCM system to generate and label mitotic clones of *Cph* mutant cells in an otherwise wild-type background. Using two independent *Cph* null alleles, we found that *Cph* mutant clones were smaller than wild-type clones (Fig. 4I,J). Under control conditions, medium-sized clones (containing 6–15 cells) typically had several progenitor cells, multiple ECs, and at least one EE (Fig. 4I,K). In contrast, *Cph* mutant clones often consisted of only a single progenitor cell and a few ECs, with EEs rarely observed (Fig. 4I,K). These results suggest that loss of *Cph* reduces clone growth and strongly compromises the generation of the EEs, but does not fully block EC differentiation. To test whether proliferation and differentiation are interdependent, we performed epistasis experiments with a constitutively active *Ras* mutant (*Ras*^*V12*^). Expression of *Ras*^*V12*^ in progenitor cells significantly increased the number of mitotic cells in the gut and reduced the number of Pros^+^ EEs (Fig. EV5G–I). Co-expression of *Ras*^*V12*^ and *Cph*^*RNAi*^ in progenitor cells increased the number of mitotically active cells compared to *Cph*^*RNAi*^ only condition but failed to restore EE differentiation (Fig. EV5G–I). Thus, proliferative stimulation is insufficient to rescue EE fate in the absence of *Cph*, indicating that proliferation and differentiation are not strictly interdependent in this context.

### Scute binds to the *Cph* locus and promotes its expression during low Notch signalling

**Figure 5:**
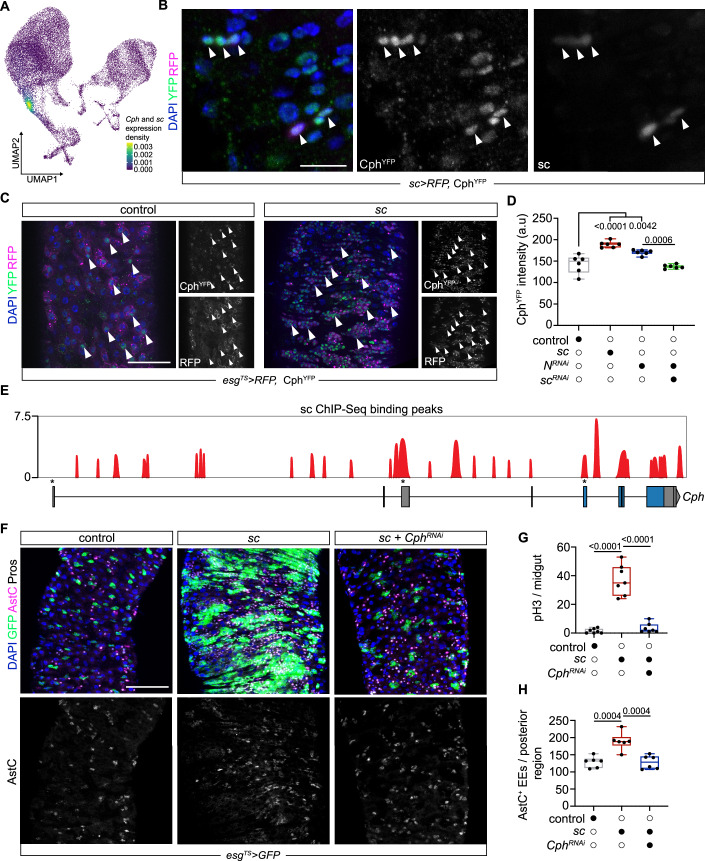
Scute promotes Cph expression. (**A**) UMAP showing joint expression density of *sc* and *Cph*. (**B**) *sc* is co-expressed in a sub-population of *Cph*^+^ cells in vivo (arrowheads). Scale bar 50 μm. (**C**) Confocal images of the posterior midgut in control conditions and sc overexpression in progenitor cells. Note that the number of Cph^YFP^-expressing progenitor cells increases after sc overexpression (arrowheads). Scale bar 100 μm. (**D**) Quantification of Cph^YFP^ intensity in progenitor cells under different genetic conditions. (**E**) ChIP-seq binding peaks of Scute within the *Cph* locus. Asterisks mark transcriptional start sites. (**F**) Overexpression of *sc* in progenitor cells increases the number of progenitors as well as AstC^+^ EEs. Silencing *Cph* in progenitor cells overexpressing *sc* suppresses the phenotypes associated with *sc* overexpression. Scale bar 100 μm. (**G**) Quantification of mitotic cells across the entire midgut. (**H**) Quantification of AstC^+^ EE cells in the posterior region. For (**D**, **G**, **H**), three independent replicates were done with the following sample size from left to right: (**D**) *n* = 6, 6, 6, 6; (**G**) *n* = 7, 7, 7; (**H**) *n* = 6, 6, 6. For all images, nuclei are labelled with DAPI. Box plots: line, median; box, 75th–25th percentiles; whiskers, minimum to maximum. One-way ANOVA test with Tukey post hoc comparison was used for (**D**, **G**, **H**). Source data are available online for this figure.

**Figure EV6:**
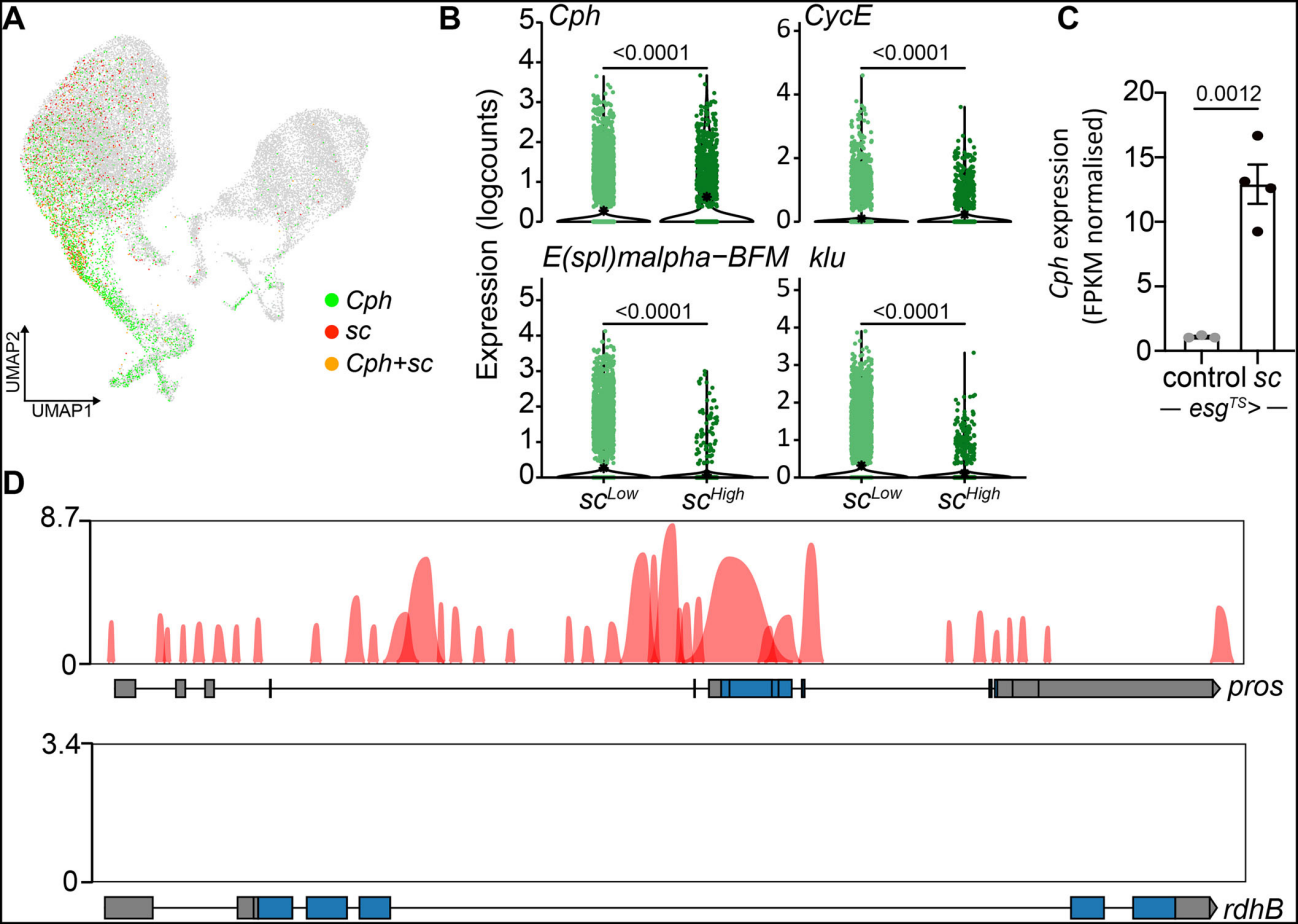
Characteristics of *sc* expressing intestinal stem cells, related to Fig. 4. (**A**) UMAP of *Cph*^+^ (green) and *sc*^+^ (red) cells, with co-expression indicated in orange. (**B**) Expression of *Cph*, *CycE*, *E(spl)mα-BFM* and *klu* in sc^High^ and sc^Low^ expressing intestinal cells. Note that *Cph* is expressed in sc^High^ expressing intestinal cells. (**C**) Re-analysis of Bulk RNA-sequencing of *sc* overexpression in progenitor cells (Chen et al, 2018), demonstrating increased *Cph* expression in progenitor cells. (**D**) ChIP-seq tracks for *sc* binding to *pros* and *rdhB*. For (**B**), two independent replicates of scRNA-seq were performed. Each dot represents a single cell. For (**C**), data represent three independent replicates for control and four for sc overexpression. Statistical test for (**B**, **C**) were done using Seurat’s FindMarkers function with test.use = “MAST“ or PyDESeq2, respectively.

Previous reports demonstrate that *sc* is transiently expressed in a sub-population of ISCs in response to low Notch signalling (Chen et al, 2018). Sc in turn binds to the *pros* locus, a master regulator of EE fate, to increase its expression and commit ISCs to the EE lineage. Using our scRNA-seq dataset, we confirmed the increase of *sc* expression in the *Notch*^*sgRNAx2*^ condition and observed an overlap between *sc* and *Cph* in the ISC and EEP populations (Figs. 5A, EV3C, and EV6A). By categorising ISCs into *sc*^High^ and *sc*^Low^ populations, we found higher *Cph* expression in the *sc*^High^ population (Fig. EV6B). *sc*^High^ ISCs also displayed low Notch signalling activity and increased expression of cell cycle regulators, including *CycE* and *CycA*, when compared to the *sc*^Low^ populations, consistent with their pro-proliferative state (Fig. EV6B). Using a previously validated enhancer trap line that partially recapitulates *sc* expression (Chen et al, 2018), we found sparse co-expression of *sc* and *Cph* in vivo (Fig. 5B).

Given the overlap in *sc* and *Cph* expression, we investigated whether Sc is able to directly regulate *Cph* expression. By re-analysing previously published bulk RNA-sequencing data from intestinal progenitor cells overexpressing *sc* (Chen et al, 2018), we found *Cph* expression significantly upregulated in this context (Fig. EV6C). To validate this, we overexpressed *sc* specifically in progenitor cells and monitored the intensity of Cph^YFP^ within this population. In line with the bulk RNA-seq dataset, we observed a marked increased in Cph^YFP^ levels in progenitor cells overexpressing *sc*, demonstrating that *sc* is sufficient to regulate Cph protein abundance (Fig. 5C,D). To determine if *sc* is required for the upregulation of Cph protein levels, we knocked down *sc* in the context of silencing *Notch*, which by itself increases *Cph* expression. Knockdown of *sc* markedly reduced Cph^YFP^ intensity in progenitor cells expressing *Notch*^*RNAi*^, indicating that *sc* is required for the *Notch*^*RNAi*^-dependent upregulation of *Cph* (Fig. 5C,D). To gain mechanistic insight into how *sc* regulates *Cph* expression, we used publicly available ChIP-seq data obtained for Sc in the progenitor population (Li et al, 2017). We confirmed previous reports that Sc binds to the *pros* locus and further demonstrated the specificity of the ChIP-seq data by showing that Sc does not bind to *rdhB*, which is enriched in the adult eye and not expressed in the intestine (Fig. EV6D). Interestingly, we detected Sc binding peaks at *Cph* transcriptional start sites (TSS), specifically in the TSS region of the transcripts Cph-Rl and Cph-RK/Cph-PL, as well as in exonic and intronic regions (Fig. 5E). These data demonstrate that *sc* directly regulates *Cph* expression.

In light of the findings above, we hypothesised that *Cph* functions as a mediator of *sc* activity in regulating progenitor proliferation and EE generation. We observed that *sc* overexpression resulted in a significant expansion of actively dividing progenitor cells and increased the number of AstC^+^ EEs (Fig. 5F–H). Knockdown of *Cph* in progenitor cells overexpressing *sc* rescued both of these phenotypes, indicating that *Cph* is required for *sc*-induced changes in cell type composition (Fig. 5F–H). In conclusion, our data identifies *Cph* as a direct transcriptional target of *sc* and a key mediator of *sc*-induced proliferation and differentiation in intestinal stem cells.

### Cph is required to remodel the transcriptome of progenitor cells upon loss of Notch signalling

**Figure 6:**
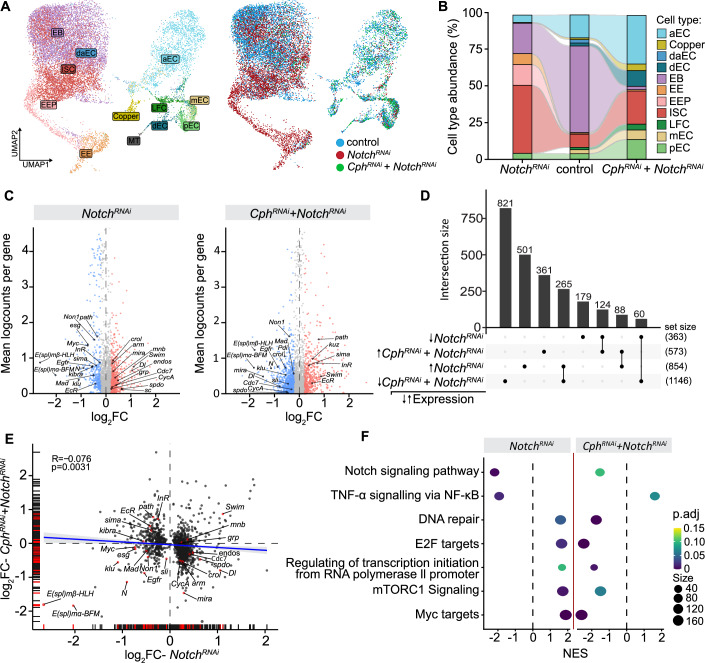
*Cph* is required to remodel the transcriptome of Notch-depleted intestinal stem cells. (**A**) UMAP plot coloured by intestinal cell types and split by perturbation conditions. (**B**) Alluvial plot representing changes in cell type abundance in the respective perturbation condition. (**C**) MA-plot highlighting differentially expressed genes in ISCs+EEPs for the respective perturbation condition. (**D**) UpSet plot illustrating the number of differentially expressed genes and their intersections between groups. Arrows indicate whether gene expression is up or down in the perturbed condition, and set size indicates the total number of genes per group. (**E**) Pearson’s correlation coefficient of all shared genes in *Notch*^*RNAi*^ and *Cph*^*RNAi*^*+Notch*^*RNAi*^. (**F**) Gene set enrichment analysis results for each condition (apart from Notch signalling, only the enriched pathways with opposite effects between the two perturbations are shown). clusterProfiler’s GSEA function was used to calculate adjusted *P* values for (**F**), whereas correlation *P* values were computed using a two-sided *t* test on the correlation coefficient for (**E**).

**Figure EV7:**
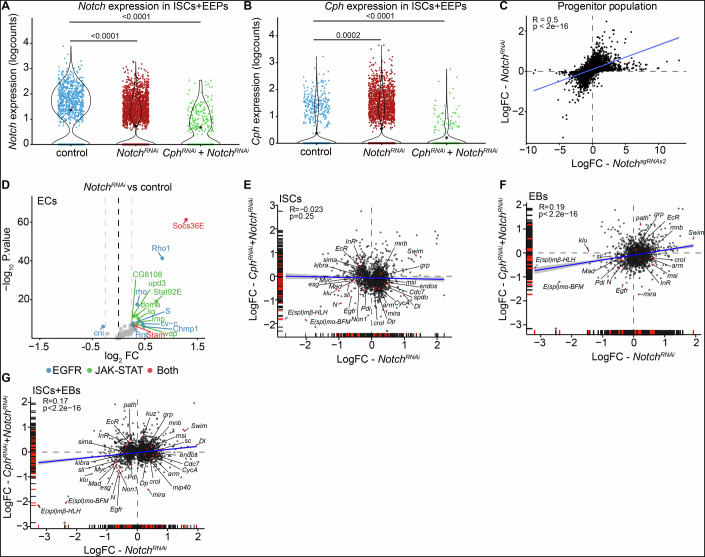
Transcriptional reprogramming of progenitor cells, related to Fig. 5. (**A**, **B**) Expression of *Notch* (**A**) and *Cph* (**B**) in ISCs+EEPs under control, *Notch*^*RNAi*^ and *Cph*^*RNAi*^+*Notch*^*RNAi*^ condition. (**C**) Correlation coefficient comparing differential gene expression results between *Notch*^*RNAi*^ and *Notch*^*sgRNAx2*^. (**D**) Volcano plot of differential gene expression in the EC population in control vs *Notch*^*RNAi*^. Genes belonging to the EGFR, JAK-STAT or both signalling pathways are colour-coded. (**E**) Correlation coefficient of all shared genes in ISCs in *Notch*^*RNAi*^ and *Cph*^*RNAi*^*+Notch*^*RNAi*^ condition. (**F**) Correlation coefficient of all shared genes in EBs in *Notch*^*RNAi*^ and *Cph*^*RNAi*^*+Notch*^*RNAi*^ condition. (**G**) Correlation coefficient of all shared genes in ISCs and EBs in *Notch*^*RNAi*^ and *Cph*^*RNAi*^*+Notch*^*RNAi*^ condition. For (**A**, **B**, **D**, **E**), one independent scRNA-seq replicate was performed per condition. Significance for (**A**, **B**, **D**) were calculated using Seurat’s FindMarkers function with test.use = “MAST“, for (**C**, **E**–**G**) correlation *P* values were computed using a two-sided *t* test on the correlation coefficient.

Thus far, we have shown that when Notch signalling is inactivated *Cph* expression is induced by *sc* and is required for maintaining the progenitor and EE populations. To investigate the transcriptional programmes that *Cph* regulates during this process, we performed scRNA-seq of the midgut while expressing *Notch*^*RNAi*^ or *Cph*^*RNAi*^*+Notch*^*RNAi*^ in the progenitor population. After quality control, we obtained 17,059 cells across control, perturbed and co-perturbed conditions and identified all major cell type clusters (Fig. 6A). We confirm that both *Notch* and *Cph* expression were downregulated in ISC+EEPs when targeted with *RNAi* (Fig. EV7A,B). We also show that the differentially expressed genes between *Notch*^*RNAi*^ and *Notch*^*sgRNAx2*^ conditions were positively correlated, indicating that both perturbations elicit similar transcriptional changes within the progenitor population (Fig. EV7C). Indeed, a notable increase in *Cph* expression was also observed in ISC+EEPs expressing *Notch*^*RNAi*^ (Fig. EV7B), underscoring the strong induction of *Cph* expression when Notch signalling is disrupted, whether through *RNAi* silencing or CRISPR mutagenesis.

To investigate changes in epithelial cell composition, we quantified cell type abundances across all conditions. We observed a notable increase in the number of ISCs, EEPs, EEs and a decrease in EBs in the *Notch*^*RNAi*^ condition (Fig. 6B), consistent with observations made using CRISPR-Cas9 (Fig. 1B–D). Co-expression of *Cph*^*RNAi*^ and *Notch*^*RNAi*^ in progenitors reduced the numbers of ISCs, EEPs, and EEs, while driving an increase in ECs without a corresponding rise in EBs compared to *Notch*^*RNAi*^-only condition (Fig. 6B). This suggests that *Cph* is required for *Notch*^*RNAi*^-induced changes to cell type composition. Previous reports indicate that *Notch*^*RNAi*^-induced tumours invade the basement membrane, resulting in the loss of ECs, which triggers JAK-STAT and EGFR-dependent cytokine signalling to further accelerate tumour growth (Patel et al, 2015). Consistently, we observed that *Upd3* and genes involved in the regulation of JAK/STAT signalling, as well as EGFR signalling components, were significantly upregulated in the EC population when *Notch* was silenced in progenitor cells (Fig. EV7D). This demonstrates that our scRNA-seq data captures expected cell type differences as well as non-autonomous gene expression changes.

To further understand the function of *Cph* in progenitor cells, we identified DEGs within specific progenitor populations, including ISCs, EBs, ISC/EBs and ISC/EEPs. We found multiple Notch target genes, such as *E(spl)mβ-HLH* and *E(spl)mα-BFM*, which were significantly downregulated in ISC/EEPs when *Notch* was silenced in progenitor cells (Fig. 6C). We also observed changes in the expression of key TFs involved in ISC differentiation, including *sc* and *klu* (Fig. 6C). Moreover, genes related to the cell cycle and ISC maintenance were significantly upregulated in the *Notch*^*RNAi*^ condition, including *CycA*, *mira* and *Dl* (Fig. 6C). In contrast, many of these genes were downregulated in ISC/EEPs expressing *Cph*^*RNAi*^*+Notch*^*RNAi*^, suggesting that *Cph* is involved in inducing their expression (Fig. 6C). A quantitative display of the intersection between the number of differentially expressed genes highlighted that many upregulated genes in *Notch*^*RNAi*^ are downregulated in *Cph*^*RNAi*^*+Notch*^*RNAi*^, and vice versa (Fig. 6D).

To more directly understand the requirement of *Cph* to remodel the transcriptome of *Notch*-depleted progenitor cells, we used Pearson’s correlation coefficient to compare the relationship between all shared genes in *Notch*^*RNAi*^ and *Cph*^*RNAi*^*+Notch*^*RNAi*^ conditions. We hypothesised that if *Cph* does not impact the transcriptome of *Notch*-depleted cells, then there would be a positive correlation between the log-fold changes of *Notch*^*RNAi*^ and *Cph*^*RNAi*^*+Notch*^*RNAi*^. However, we found that the transcriptional signatures of *Notch*-depleted ISCs and ISC/EEPs were either blunted or negatively correlated when *Cph* was silenced (Figs. 6E and EV7E). Conversely, we observed a positive correlation between the shared genes when the EB population was included in the analysis, indicating that *Cph* has limited roles in altering the transcriptome of this progenitor cell type when *Notch* is depleted (Fig. EV7F,G), consistent with the lack of *Cph* expression in EBs. Gene set enrichment analysis revealed that genes involved in Myc, mTORC1 signalling, DNA repair and E2F targets were upregulated when *Notch* was silenced, whereas genes involved in NF-κB signalling were downregulated (Fig. 6F). Notably, following co-expression of *Cph*^*RNAi*^*+Notch*^*RNAi*^, the same pathways previously upregulated after silencing *Notch* were downregulated (Fig. 6F). Importantly, this transcriptional rewiring was not due to increasing *Notch* expression or Notch signalling activity, as the expression of *Notch* and Notch pathway target genes, such as *E(spl)mβ-HLH* and *E(spl)mα-BFM*, were significantly downregulated in both the *Notch*^*RNAi*^ and *Cph*^*RNAi*^*+Notch*^*RNAi*^ conditions (Fig. 6C,F). In conclusion, our findings demonstrate that *Cph* is required to reprogramme the transcriptional state of ISCs and EEPs under conditions of reduced Notch signalling.

### Genome-wide chromatin profiling of Cph reveals novel target genes involved in ISC proliferation and EE generation

**Figure EV8:**
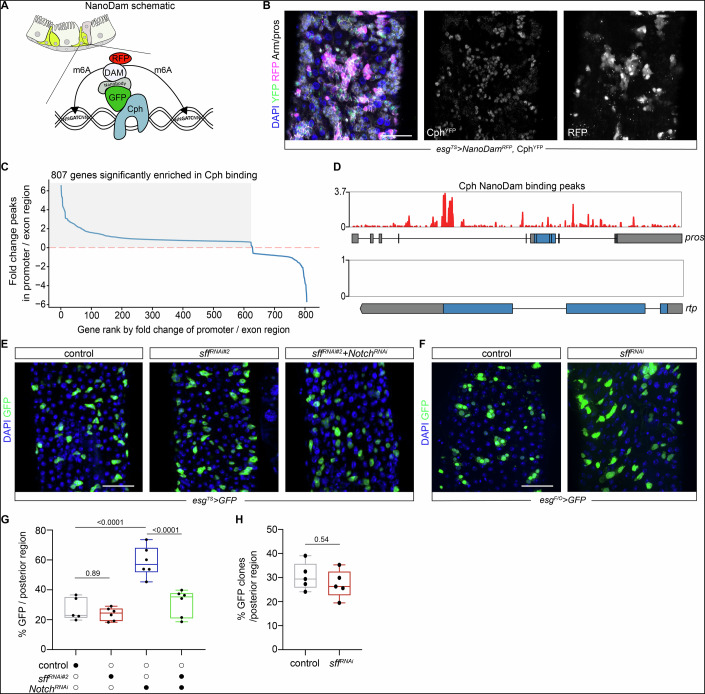
NanoDam profiling of Cph, related to Fig. 6. (**A**) Schematic of NanoDam in progenitor cells. (**B**) Expression of *NanoDam*^*RFP*^ in *GFP*^*+*^ progenitor cells using the *esg*^*TS*^ driver. Scale bar 100 μm. (**C**) Significant Cph target genes within the progenitor population. (**D**) Cph NanoDam binding intensity on the *pros* and *rtp* locus. Binding intensities are shown as log_2_-fold enrichment. (**E**) Confocal images of the posterior midgut under different genetic conditions. (**F**) Confocal images of Flip-out clones within the posterior midgut under different genetic conditions. (**G**) Quantification of the number of GFP^+^ progenitor cells in the posterior region. (**H**) Quantification of the number of GFP^+^ clones within the posterior midgut. For (**G**, **H**), a minimum of two independent replicates were done with the following sample sizes from left to right: (**G**) *n* = 5, 6, 6, 6; (**H**) *n* = 5, 5. For all images, nuclei are labelled with DAPI. Box plots: line, median; box, 75th–25th percentiles; whiskers, minimum to maximum. One-way ANOVA test with Tukey post hoc comparison was used for (**G**). Mann–Whitney test was used for (**H**).

**Figure 7:**
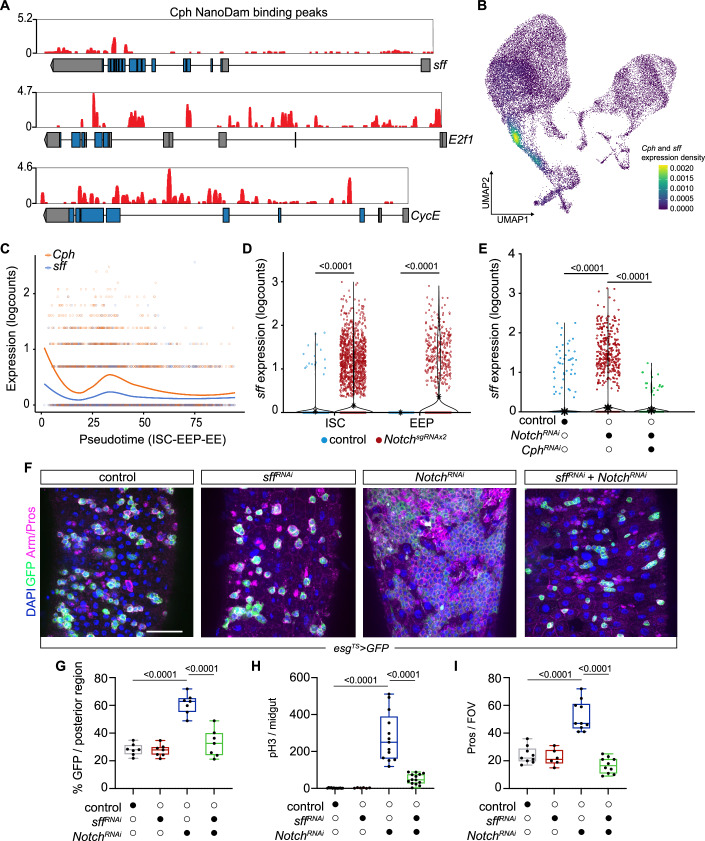
NanoDam identifies Cph target genes that are important for the proliferation and differentiation of ISCs. (**A**) NanoDam binding profile for Cph in various target genes. Binding intensities are shown as log_2_-fold enrichment. (**B**) UMAP showing the joint expression density of *Cph* and *sff*. (**C**) Expression of *Cph* and *sff* along the ISC-EEP-EE lineage. (**D**) Expression of *sff* in ISCs and EEPs during homeostasis and *Notch* mutant condition. (**E**) Expression of *sff* in all progenitors during homeostasis, *Notch*^*RNAi*^ and *Cph*^*RNAi*^*+Notch*^*RNAi*^ conditions. (**F**) Knockdown of *sff* in progenitors depleted of *Notch* significantly reduces GFP+ progenitors and pros+ EEs when compared to *Notch*^*RNAi*^ expressing progenitors. Scale bar 100 μm. (**G**) Quantification of GFP^+^ cells in the posterior midgut. (**H**) Quantification of mitotic cells across the entire midgut. (**I**) Quantification of Pros^+^ EEs in the field of view. For (**D**), two independent replicates of scRNA were performed, whereas for (**E**) one independent replicate was done. Each dot represents a single cell. For (**G**–**I**), a minimum of three independent replicates were done with the following sample sizes from left to right: (**G**) *n* = 7, 7, 6, 7; (**H**) *n* = 13, 6, 13, 14; (**I**) *n* = 9, 6, 10, 10. For all images, nuclei are labelled with DAPI. Box plots: line, median; box, 75th–25th percentiles; whiskers, minimum to maximum. Seurat FindMarkers function with test.use = “MAST“ was used to calculate *P* values for (**D**, **E**). One-way ANOVA test with Tukey post hoc comparison was used for (**G**–**I**). Source data are available online for this figure.

Next, we sought to identify Cph target genes in vivo by performing NanoDam to generate DNA-binding profiles for Cph (Fig. EV8A). NanoDam utilises a GFP/YFP-recognising nanobody which links Dam methylase to an endogenously tagged DNA-binding protein, resulting in m6A methylation of surrounding GATC sites (Tang et al, 2022). This method is advantageous over other chromatin profiling approaches, such as targeted DamID (Southall et al, 2013), as it does not require the overexpression of a DNA-binding protein. We restricted expression of NanoDam specifically to the progenitor population using the *esg*^*TS*^ driver and induced its binding to endogenously tagged Cph^YFP^ for ~17 h (Fig. EV8A,B). After quality control and normalisation to the NanoDam-only control, significant Cph binding sites were found for 807 genes across the entire genome (Fig. EV8C). We observed pronounced peak intensity profiles for cell cycle-related genes, including *CycE* and *E2F1* (Fig. 7A). Moreover, we also identified several peaks in the *pros* locus (Fig. EV8D), suggesting that *Cph* may regulate its expression. Conversely, we did not identify significant peaks in *rtp*, which is not expressed in the intestine during steady state (Fig. EV8D).

One of the top genes identified in our Cph NanoDam dataset, based on differential binding (log2 fold change and FDR), was *sugar-free frosting* (*sff*) (Fig. 7A), which is poorly characterised with respect to its function in the intestine. *sff* was particularly interesting because it was barely detectable during homeostasis but its expression increased in *Notch*^*sgRNAx2*^ mutant flies, coinciding with *Cph* along the ISC-EEP-EE trajectory (Fig. 7B–D). The induction in *sff* expression was prominent in ISCs and EEPs after Notch signalling was disrupted using either *Notch*^*sgRNAx2*^ or *Notch*^*RNAi*^ (Fig. 7D,E). Interestingly, the elevated expression of *sff* returned back to wild-type levels after co-silencing *Cph* with *Notch*, indicating that *Cph* directly regulates the expression of *sff* (Fig. 7E).

Sff has previously been characterised as an SAD-like kinase that plays a key role in regulating vesicle tethering and glycosylation during embryogenesis (Baas et al, 2011). Since *sff* expression is induced during low Notch condition, we asked whether it is functionally required for the proliferation and differentiation of *Notch*-depleted tumours. While flies expressing progenitor-specific *Notch*^*RNAi*^ developed neuroendocrine tumour-like structures characterised by excessive ISC and EE cells, silencing *sff* with two independent *RNAi* lines significantly reduced progenitor proliferation (Figs. 7F,G and EV8E,F). Consistently, we also observed a significant decrease in the fraction of mitotically active cells in flies expressing *sff*^*RNAi*^ +*Notch*^*RNAi*^ compared to *Notch*^*RNAi*^-only (Fig. 7F,H). Moreover, co-silencing of *sff* and *Notch* highlighted a key requirement for *sff* in the formation of EEs (Fig. 7F,I). By contrast, silencing *sff* under homoeostatic conditions had no effect on progenitor cell, EE numbers or epithelial turnover, consistent with its minimal expression during steady state (Figs. 7F–I and EV8E–H). These findings reveal a previously unrecognised role for vesicle tethering and glycosylation in driving hyperproliferation and differentiation of ISCs during low Notch signalling. In conclusion, our in vivo  chromatin binding profiles of Cph highlighted key genes involved in cell cycle regulation, EE differentiation and further revealed a novel role for *sff*, which is required for ISC proliferation and EE generation when Notch signalling is low.

### Auto-repression of Cph protects progenitor cells against autophagy and cell death

**Figure 8:**
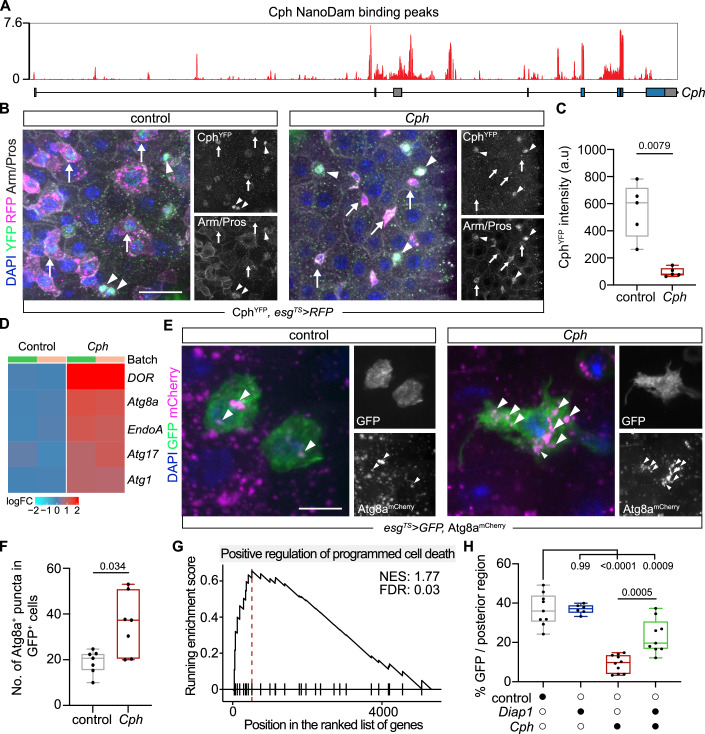
Cph represses its own expression to prevent autophagy and programmed cell death. (**A**) NanoDam binding profile for Cph demonstrating that it binds to its own locus. Binding intensities are shown as log_2_-fold enrichment. (**B**) Overexpression of Cph in the progenitor population (RFP^+^) decreases endogenous Cph^YFP^ intensity in progenitor cells (arrows) but not EEs (arrowheads). Scale bar 20 μm. (**C**) Quantification of Cph^YFP^ intensity in progenitor cells. (**D**) Heatmap of gene expression in control and progenitor-specific Cph overexpression conditions. Log fold change was calculated with respect to the average control expression. (**E**) Confocal images of progenitor cells show an increased number of Atg8a⁺ autophagosomes upon Cph overexpression. Scale bar 10 μm. (**F**) Quantification of the number of Atg8^+^ puncta in GFP^+^ progenitor cells. (**G**) Running enrichment score of positive regulators involved in programmed cell death. (**H**) Quantification of GFP^+^ cells in the posterior midgut. For (**C**), intensity measurements for each condition were taken from one matched experiment, For (**F**, **H**), a minimum two independent replicates were done with the following sample size from left to right: (**C**) *n* = 5, 5; (**F**) *n* = 7, 7; (**H**) *n* = 9, 6, 10, 9. For all images, nuclei are labelled with DAPI. Box plots: line, median; box, 75th–25th percentiles; whiskers, minimum to maximum. Mann–Whitney test was used for (**C**, **F**). One-way ANOVA test with Tukey post hoc comparison was used for (**H**). Source data are available online for this figure.

**Figure EV9:**
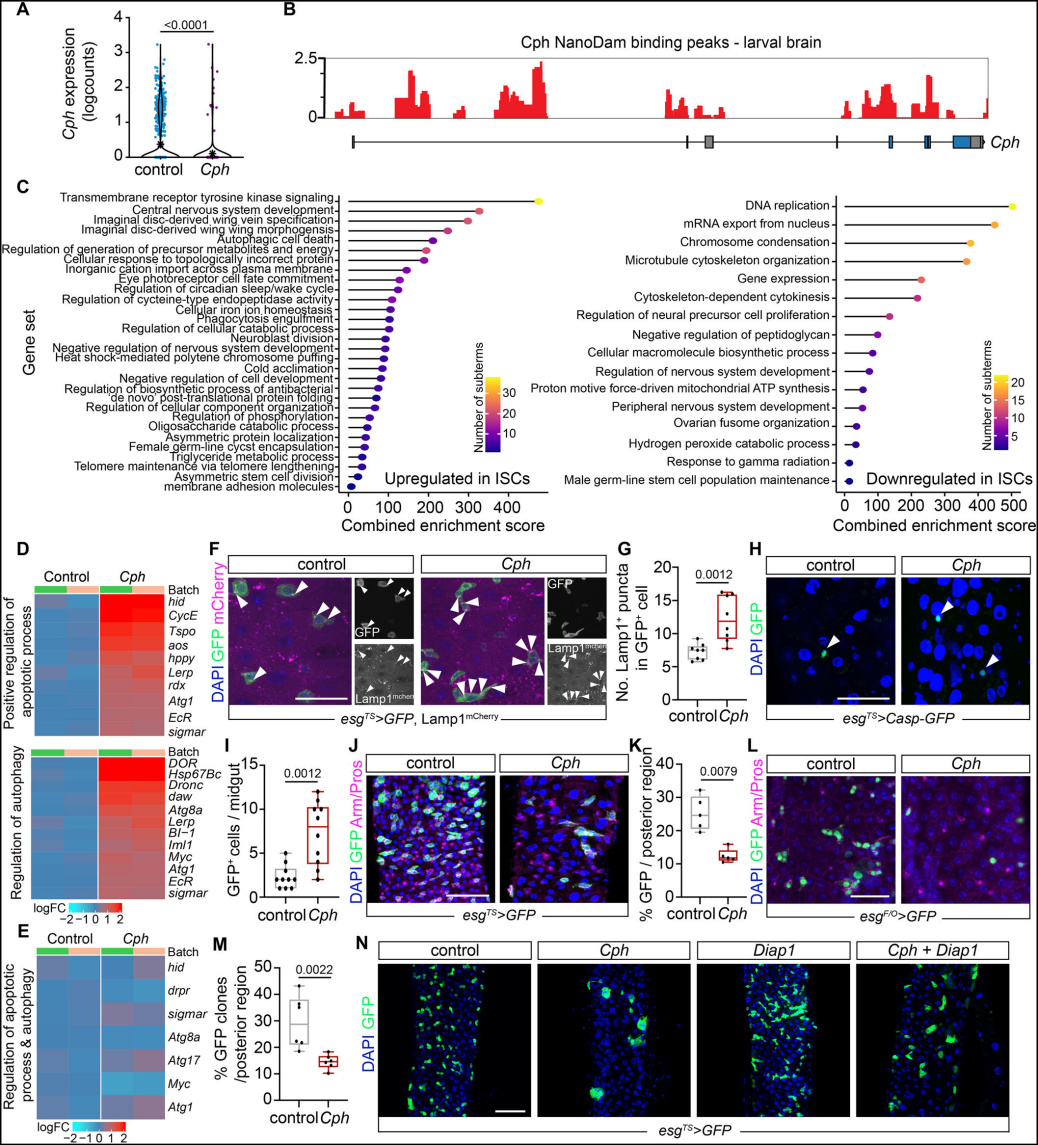
Sustained expression of Cph induces autophagy and cell death, related to Fig. 7. (**A**) Expression of Cph derived from scRNA-seq of control and progenitor-specific Cph overexpression condition. (**B**) NanoDam profiling of Cph in the *Drosophila* larval brain reveals self-binding. Binding intensities are shown as log_2_-fold enrichment. (**C**) Overrepresentation analysis of upregulated and downregulated terms in ISCs overexpressing *Cph*. (**D**) Heatmap of cell death-related genes and autophagy-related genes specifically in ISCs across two independent scRNA-seq replicates in control and *Cph* overexpression conditions. Log fold change was calculated with respect to the average control expression. (**E**) Heatmap of cell death-related genes and autophagy-related genes in EBs. (**F**) Confocal images of endogenously tagged Lamp1^mCherry^ in GFP^+^ progenitor cells within the posterior region. Scale bar 40 μm. (**G**) Quantification of the number of Lamp^+^ puncta in GFP^+^ progenitor cells within the posterior midgut. (**H**) Confocal images of the apoptotic sensor Casp-GFP in the posterior midgut. Scale bar 40 μm. (**I**) Quantification of the number of Casp-GFP^+^ cells in the midgut. (**J**) Confocal images of the posterior midgut showing that overexpression of Cph in progenitor cells decreases the number of progenitor cells. (**K**) Quantification of the number of GFP+ progenitor cells in the posterior midgut. (**L**) Confocal images of Flip-out clones within the posterior midgut under control and progenitor-specific Cph overexpression condition. Scale bar 40 μm. (**M**) Quantification of GFP+ clones within the posterior region. Scale bar 100 μm. (**N**) Confocal images of GFP^+^ progenitor cells in the posterior midgut. Scale bar 100 μm. For (**A**), two independent scRNA-seq replicates were done for each condition. Each dot represents a single cell. For (**G**, **I**, **M**), a minimum of two independent replicates were done, For (**K**), a single matched condition was quantified with the following sample size from left to right: (**G**) *n* = 8, 8; (**I**) *n* = 10, 10; (**K**) *n* = 5, 5; (**M**) *n* = 6, 6. For all images, nuclei are labelled with DAPI. Box plots: line, median; box, 75th–25th percentiles; whiskers, minimum to maximum. Mann–Whitney test was used for all graphs, except for (**A**), where the Seurat’s FindMarkers function with test.use = “MAST“ was used to calculate *P* value.

Given that *sc* promotes *Cph* expression, we next sought to identify how *Cph* expression is downregulated. Our NanoDam profiling of *Cph* revealed that *Cph* binds to its own locus at multiple sites, suggesting that *Cph* may regulate its own expression (Fig. 8A). To recapitulate conditions where *Cph* expression is elevated and determine how endogenous *Cph* expression is altered, we overexpressed *Cph* in progenitor cells and quantified Cph^YFP^. Overexpression of *Cph* significantly reduced Cph^YFP^ in progenitor cells but not in EE cells, where *esg* expression is absent, demonstrating that *Cph* suppresses its own expression (Fig. 8B,C). scRNA-seq of flies overexpressing *Cph* in progenitor cells revealed a marked reduction in *Cph* expression within the ISC population, demonstrating that the auto-suppressive function of *Cph* occurs at the transcriptional level (Fig. EV9A). These findings indicate that the induction of *Cph* during ISC-EEP-EE differentiation is followed by auto-suppressive feedback which limits *Cph* expression. To understand whether autoregulation of *Cph* is a conserved mechanism, we investigated *Cph* NanoDam data from the developing *Drosophila* larval brain^45^. Consistently, we found that *Cph* binds to itself in the larval brain, suggesting that autoregulation may be a general mechanism to control *Cph* expression across different tissues and developmental stages (Fig. EV9B).

To understand the functional consequences of sustained *Cph* expression, we identified DEGs and performed GSEA in ISCs. This revealed 600 upregulated genes and 470 downregulated genes, with upregulated genes being significantly enriched in gene sets related to a variety of different processes, including stem cell function, protein quality control, autophagosome assembly and regulation of autophagy (Fig. EV9C). Consistent with the later signatures, we observed increased expression of *Atg8a*, *Atg1*, and *Atg17* in ISCs and not EBs across both scRNA-seq replicates upon *Cph* overexpression (Figs. 8D and EV9D,E). Because changes in autophagosome dynamics are often accompanied by altered lysosome biogenesis, we examined lysosomal abundance using the fluorescent marker Lamp1–mCherry (Hegedus et al, 2016). *Cph* overexpression increased the number of Lamp1⁺ lysosomes in progenitor cells (Fig. EV9F,G). To assess autophagosome abundance, we quantified the autophagosome marker Atg8a–mCherry (Nagy et al, 2016), which revealed a significant increase in the number of autophagosomes in *Cph*-overexpressing cells, consistent with the transcriptional elevation of autophagy-related genes (Fig. 8E,F). As increased autophagosome number can reflect either autophagy induction or reduced autophagosome clearance, we conclude that uncontrolled *Cph* expression elevates autophagosome abundance.

GSEA also revealed strong enrichment of apoptosis-related terms, including positive regulation of apoptotic process and programmed cell death, suggesting that *Cph*-overexpressing cells trigger apoptosis (Fig. 8G). To validate this in vivo, we used the genetically encoded caspase-3 activity sensor GC3Ai (Schott et al, 2017). This reporter is engineered from GFP in which the protein’s N- and C-termini are joined by a short linker containing the caspase-3 recognition sequence (DEVD). In its intact state, this circularised GFP remains non-fluorescent. When effector caspases become active, they cut the DEVD site within the linker and restore GFP fluorescence. This allows apoptotic activity to be monitored dynamically in vivo (Schott et al, 2017). Using this sensor in combination with the *esg* driver to overexpress *Cph* in ISC/EBs, we observed a marked increase in the number of apoptotic cells following *Cph* overexpression (Fig. EV9H,I). Consistent with this observation, we observed a pronounced decrease in the number of *esg*^*+*^ cells that overexpressed *Cph*, as well as a decrease in epithelial turnover (Figs. 8H and EV9J–M). Because ISCs and EBs differ in their apoptotic sensitivities (Reiff et al, 2019; Sulekh et al, 2024), we utilised our scRNA-seq data to establish that apoptotic signatures are induced specifically in ISCs and not EBs (Figs. 8G and EV9D,E). To assess the functional relevance of apoptosis, we blocked apoptosis by overexpressing the caspase inhibitor, *Diap1*. This intervention partially rescued the *esg*^*+*^ cells, demonstrating that apoptosis is a key driver of *esg*^*+*^ cell depletion when *Cph* expression is unchecked (Figs. 8H and EV9N). Our data demonstrate that failure to restrain *Cph* expression increases autophagosome accumulation and activates a pro-apoptotic programme, leading to depletion of ISCs.

## Discussion

We present a comprehensive multi-omic analysis of the fly midgut combined with single and co-perturbation studies to identify *Cph* as a novel regulator of ISC maintenance and EE differentiation. *Cph* is present in ISCs and EEs during homeostasis, and its expression is induced in ISCs and EEPs when Notch signalling is low, a finding that was recently reported (King et al, 2024). In this condition, we show that *scute* binds to multiple sites within the *Cph* locus to promote its expression. *Cph* reprogrammes the transcriptome of ISCs and EEPs by directly regulating cell cycle and lineage commitment genes, while simultaneously repressing its own expression. This autoinhibitory feedback mechanism prevents progenitor cells from accumulating autophagosomes and undergoing cell death, which ensures ISC maintenance and cell fate commitment are faithfully executed. Our extensive scRNA-seq dataset also provides an inferred spatial atlas of the *Drosophila* midgut. This dataset may serve as a crucial reference map for future research on stem cell maintenance and lineage commitment in the midgut and can be explored by the community through our Shiny App.

By profiling DNA-binding sites of Cph in vivo using NanoDam, we identified hundreds of target genes that are bound by Cph in progenitor cells. Indeed, we identified *sff*, a homologue of SAD kinase, as a crucial Cph target gene that regulates ISC proliferation and EE generation. Very little is known about the function of *sff* outside the context of the embryo where it has been described to be involved in vesicle tethering and glycosylation at the Golgi (Baas et al, 2011), which are key for membrane trafficking. Given that membrane trafficking and endocytic events are crucial for regulating Notch signalling (Yamamoto et al, 2010), our findings raise the possibility that *sff* may also be involved in distinct membrane trafficking of Notch pathway components.

Our in vivo chromatin profiling, together with the finding that *Cph* overexpression lowers endogenous *Cph* mRNA, supports a model in which *Cph* represses its own expression by directly binding its genomic locus. While we cannot exclude the possibility of post-translational regulation of *Cph*, the observed reduction at the mRNA level indicates that at least part of this feedback occurs at the transcriptional level. Tight control of *Cph* expression, e.g. through such a feedback mechanism, is crucial for homeostatic regulation, as failure to limit *Cph* expression results in the accumulation of autophagosomes and cell death in ISCs. Previous reports indicate that autophagy in the *Drosophila* midgut can be both tumour suppressive or oncogenic (Nagy et al, 2016; Zhang et al, 2019). Our observation that upregulation of autophagy-related gene expression coincides with a reduction in the progenitor population suggests that, in this context, autophagy is detrimental to ISCs. This form of autoregulation ensures that ISCs do not accumulate autophagosomes and undergo cell death, thus serving as a protective mechanism. A self-stimulatory mechanism has been described for *sc* (Chen et al, 2018). To our knowledge, our study is the first to report an auto-repressive mechanism that ensures ISC function. Interestingly, the mammalian homologue of *Cph*, *BCL11b*, functions both as a transcriptional activator and repressor in lymphoid cells (Califano et al, 2015), suggesting that the dual regulatory role of *Cph* may be conserved in higher eukaryotes. Another example is the transcription factor *Hes1*, which not only activates the expression of target genes but also represses its own transcription (Hirata et al, 2002; Takebayashi et al, 1994). Such dual-functioning transcription factors likely play a key role in coordinating diverse biological processes while simultaneously maintaining tight control over their own expression levels – a process that may involve other molecular factors.

While our findings demonstrate that *sc* can directly regulate *Cph* expression, it is important to recognise that *sc* is only transiently expressed in ISCs during homeostasis (Chen et al, 2018), whereas *Cph* expression remains stable. This implies that additional factors are involved in regulating *Cph* expression during steady state, which should be a subject of future studies. Beyond transcriptional regulation, post-translational modifications may also play a role in controlling Cph protein levels in ISCs. Previous studies have identified Phyl as an adaptor protein that promotes the degradation of Ttk via the E3 ubiquitin ligase pathway (Yin and Xi, 2018). Notably, we observed a significant upregulation of *phly* following Notch signalling inactivation, however, further research is needed to determine whether Phly also regulates Cph abundance in ISCs.

*Cph* is homologous to mammalian *BCL11a* and *BCL11b*. *BCL11a* is highly expressed across various hematopoietic lineages and is involved in the transition from γ-globin to β-globin expression during the shift from foetal to adult erythropoiesis (Liu et al, 2003; Sankaran et al, 2009). Recently, BMP signalling was shown to downregulate *BCL11b*, which normally sustains Wnt activity by inhibiting the NuRD complex and promoting β-catenin–TCF4 interactions, thereby regulating intestinal homeostasis and regeneration (Li et al, 2024). *BCL11A* and *BCL11B* share 67% and 55% amino acid identity, with particularly strong similarity in the N-terminal region, suggesting that functional parallels may be drawn. Thus, it is conceivable *Cph* represents an evolutionarily ancient module linking transcriptional control, lineage commitment, and progenitor cell maintenance. Future studies aimed at understanding the conservation of the *Cph* self-repressive mechanism may uncover new principles of stem cell regulation.

## Methods

**Table:** Reagents and tools table

Reagent	Source	Identifier
**Antibodies**		
Mouse anti-Armadillo (1:50)	DSHB	N27A1
Mouse anti-Prospero (1:20)	DSHB	MR1A
Rabbit anti-Tachykinin (1:500)	Gift from J Veenstra (Veenstra et al, 2008)	N/A
Rabbit anti-Allatostatin C (1:500)	Gift from J Veenstra (Veenstra et al, 2008)	N/A
Rabbit anti-Phospho-Histone H3 (1:500)	Cell Signaling	9701S
Rabbit anti-PDM1 (1:1000)	Gift from C Yu and X Yang	N/A
Peroxidase IgG Fraction Monoclonal Mouse anti-Digoxin	Jackson ImmunoResearch	200-032-156
Rabbit anti-Beta Galactosidase (1:1000)	ICL, Inc.	RGAL-45A-Z
Rabbit anti-GFP (1:1000)	Invitrogen	A-11122
Goat anti-rabbit conjugated to AlexaFluor488 (1:1000)	Invitrogen	A-11034
Donkey anti-mouse conjugated to AlexaFluor549 (1:1000)	Invitrogen	A-21202
Chicken anti-mouse conjugated to AlexaFluor647 (1:1000)	Invitrogen	A-21201
DpnI	New England Biolabs	R0176S
DpnII	New England Biolabs	R0543S
**Chemicals, peptides, and recombinant proteins**		
Phosphate-buffered saline	Sigma-Aldrich	P3812-10PAK
16% Paraformaldehyde	Thermo Fisher Scientific	043368.9 M
VECTASHIELD with DAPI	Vector Laboratories	H-1200
Triton X-100	Sigma-Aldrich	X100
Elastase	Sigma-Aldrich	E0258
DIG DNA Labelling Kit	Roche	11277073910
Poly-L-Lysin -hydrobromid	Sigma-Aldrich	P1524
**Critical commercial assays**		
10X Chromium 3’ + 5’ Gene Expression Analysis kit	10X Genomics	N/A
**Deposited data**		
scRNA-seq datasets	This study	GEO: GSE276185
NanoDam dataset	This study	GEO: GSE280439
Bulk RNA-seq	Chen et al (2018)	GEO: GSE102569
ChIP-seq	Li et al (2017)	GEO: GSE84283
DamID	Guo et al (2022b)	GEO: GSE211629
**Experimental models: organisms/strains**		
*dmGlut-Gal4*	BDSC	63397
*Npc2f-Gal4*	BDSC	81176
*Path-Gal4*	BDSC	71411
*sc-Gal4*	BDSC	48607
*UAS-Cph* ^*RNAi#2*^	BDSC	26713
*UAS-sc*	BDSC	51672
*tub*-*GAL80*^*ts*^	BDSC	7019
*UAS-sff* ^*RNAi*^	VDRC	100717
*UAS-Notch* ^*RNAi*^	VDRC	27228
*UAS-Notch* ^*sgRNAx2*^	VDRC	341922
*UAS-Sff* ^*RNAi#2*^	BDSC	36656
*UAS-Cph* ^*RNAi*^	VDRC	104402
*MARCAM19A*	Gift from J Korzelius (King et al, 2024)	N/A
*Cph* ^*B32*^	Gift from J Korzelius (King et al, 2024)	N/A
*Cph* ^*A*^	Gift from J Korzelius (King et al, 2024)	N/A
*Delta-lacZ*	Gift from J Korzelius (King et al, 2024)	N/A
*Su(H)-GBE-LacZ*	Gift from J Korzelius (King et al, 2024)/S Bray (Furriols and Bray, 2001)	N/A
*Atg8a* ^*mCherry*^	Gift from G Juhász (Nagy et al, 2016)	N/A
*UAS-Diap1*	Gift from I Lohmann	N/A
*Lamp1* ^*mCherry*^	Gift from G Juhász (Hegedus et al, 2016)	N/A
*UAS-GC3Ai*	BDSC	84343
*Cph-YFP*	Gift from Andrea Brand (Fox et al, 2022), DGGR	115236
*esg* ^*TS*^ *, UAS-GFP, UAS-Cas9* ^*p.2*^	Gift from F Port (Port et al, 2020)	N/A
*esg* ^*TS*^ *, UAS-GFP, Su(H)-Gal80 (ISC* ^*TS*^ *)*	Gift from B Edgar	N/A
*Su(H)-Gbe-Gal4, UAS-GFP; TubGal80ts (EB* ^*TS*^ *)*	Gift from J Korzelius	N/A
*esg* ^*TS*^ *, UAS-GFP*	Gift from B Edgar	N/A
*esg* ^*TS*^ *, UAS-RFP*	Gift from B Edgar	N/A
*esg* ^*TS*^	Gift from B Edgar	N/A
*UAS-H2B-RFP*	Gift from T Reiff (Antonello et al, 2015)	N/A
*esg* ^*F/*O^	Gift from P Patel (Jiang et al, 2009)	N/A
*UAS-NanoDam* ^*RFP*^	Gift from A Brand (Tang et al, 2022)	N/A
*UAS-Cph*	Gift from A Brand (Fox et al, 2022)	N/A
*Pros* ^*TS*^	Boutros Lab	N/A
*w* ^*1118*^	Boutros Lab	N/A
**Oligonucleotides**		
*Vha100-4* F: ggccgcggGAGAGCAACAGCATCTTCCG	This study	N/A
*Vha100-4* R: cccggggcCAGCACTTGGATCATCTCGC	This study	N/A
Universal 3’ T7 primer for antisense probes: AGGGATCCTAATACGACTCACTATAGGGCCCGGGGC	This study	N/A
Universal 5’ T7 primer for sense probes: GAGAATTCTAATACGACTCACTATAGGGCCGCGG	This study	N/A
Sanger forward primer: CTGGCGTTTGCGAATTTGCCAAA	This study	N/A
Sanger reverse primer: GGATCTTACCAGTGCATGTGTCC	This study	N/A
**Software and algorithms**		
GraphPad Prism 8	GraphPad Software	https://www.graphpad.com/
Fiji	Schindelin et al (2012)	https://imagej.net/software/fiji/
Seurat (version 4.1.1)	Butler et al (2018); Stuart et al (2019)	https://cran.r-project.org/src/contrib/Archive/Seurat/
Adobe Illustrator (version 29.0)	Adobe	https://www.adobe.com/products/illustrator.html
CellRanger (version 7.0.1)	10X Genomics	https://support.10xgenomics.com/single-cell-gene-expression/software/downloads/latest
bowtie2 (version 2.5.4)	Langmead and Salzberg (2012)	https://sourceforge.net/projects/bowtie-bio/files/bowtie2/2.3.4/
MACS2	Zhang et al (2008) (Gaspar, 2018)	https://pypi.org/project/MACS2/
MELD	Burkhardt et al (2021)	https://github.com/KrishnaswamyLab/MELD
muscat (version 1.12.1)	Crowell H et al (2020)	https://www.bioconductor.org/packages/release/bioc/html/muscat.html
edgeR (version 3.38.0)	Chen et al (2025)	https://bioconductor.org/packages/release/bioc/html/edgeR.html
enrichR (version 3.1)	Kuleshov et al (2016)	https://www.rdocumentation.org/packages/enrichR/versions/3.2
msigdbr (version 7.5.1)	Dolgalev I (2022)	https://www.rdocumentation.org/packages/msigdbr/versions/7.5.1
tradeSeq (version 1.13.04)	Van den Berge et al (2020) (Van den Berge et al, 2020)	https://www.bioconductor.org/packages/release/bioc/html/tradeSeq.html
slingshot (version 2.4.0)	Street et al (2018)	https://bioconductor.org/packages/devel/bioc/vignettes/slingshot/inst/doc/vignette.html
condiments (version 1.6.0)	Roux de Bezieux et al (2024)	https://bioconductor.org/packages/3.18/bioc/html/condiments.html
clusterProfiler (version 4.4.4)	Yu et al (2012)	https://bioconductor.org/packages/release/bioc/html/clusterProfiler.html
SingleR (version 1.10.0)	Aran et al (2019)	https://bioconductor.org/packages/release/bioc/html/SingleR.html
Scran (version 1.24.0)	Lun et al (2016)	https://bioconductor.org/packages/release/bioc/html/scran.html
DESeq2 (version 1.36.0)	Love et al (2014)	https://bioconductor.org/packages/release/bioc/html/DESeq2.html
Nebulosa (version 1.8.0)	Alquicira-Hernandez and Powell (2021)	https://bioconda.github.io/recipes/bioconductor-nebulosa/README.html
FastQC (version 0.11.5)	Andrews (2010)	https://www.bioinformatics.babraham.ac.uk/projects/fastqc/
Integrative Genomic Viewer (IGV)	Robinson et al (2011)	https://software.broadinstitute.org/software/igv/download
R	R-project	https://cran.ma.imperial.ac.uk/
RStudio	RStudio	https://www.rstudio.com/products/rstudio/download/
Python	Python	https://www.python.org/
**Other**		
scRNA-seq analysis pipeline	This study	https://github.com/boutroslab/Supp_Redhai_Hirschmueller_2024
NanoDam analysis pipeline	This study	https://github.com/boutroslab/Supp_Redhai_Hirschmueller_2024
ChIP-seq analysis pipeline	This study	https://github.com/boutroslab/Supp_Redhai_Hirschmueller_2024
DamID analysis pipeline	This study	https://github.com/boutroslab/Supp_Redhai_Hirschmueller_2024

### Fly husbandry

*Drosophila* stocks were raised on a 12:12 h light:dark cycle and maintained on standard fly food consisting per liter of 44 g syrup, 80 g malt, 80 g corn flour, 10 g soy flour 18 g yeast, 2.4 g methly-4-hydroxybenzoate, 6.6 mL propionic acid, 0.66 mL phosphoric acid and 8 g agar. For CRISPR mutagenesis experiments, newly eclosed flies were shifted to 29 °C for 10 days and subsequently to 18 °C for 30 days before switching back to 29 °C for 1 day prior to imaging as previously reported(Bahuguna et al, 2021; Redhai et al, 2024). For all other experiments requiring temperature shift (*Gal80*^*TS*^) for transgene induction, parental lines were kept at 18 °C, and the progeny were shifted to 29 °C after eclosion for 20 days. For all experiments (unless otherwise stated), mated adult female flies were used and transferred to fresh food every 2 days.

### Escargot-flip-out experiments

Flip-out clones were generated as previously described (Jiang et al, 2009). Briefly, expression of *flippase* by *esg*^*TS*^ at 29 °C activates a constitutive Act>STOP>Gal4 driver by excising the STOP cassette flanked by FRT sites. This system was induced for 15–20 days and results in the expression of *GFP* and *RNAi* in both progenitor cells and their descendant progeny. Flies were evenly housed in control and treatment groups, and their food was changed every 2 days throughout the experiment.

### Dissection and immunohistochemistry

Flies were starved for 3 h prior to dissection to reduce luminal content in the intestine. Adult female intestines were dissected in PBS (Phosphate buffered saline, P3812-10PAK) and transferred to polylysine slides and fixed in 4% Paraformaldehyde (Thermo Scientific - 16% Paraformaldehyde diluted in PBS) for 20–60 min depending on the antibody. Samples were washed with PBST (PBS with 0.1% Triton X-100) for 30 min and then blocked with PBSTB (PBST with 1% Bovine serum albumin) for 30 min at room temperature. The primary antibody was diluted in PBSTB and incubated with samples overnight at 4 °C. Samples were then washed five times in PBST and incubated for 1.5–2.5 h at room temperature with secondary antibody (antibodies coupled to Alexa fluorophores, Invitrogen) in PBSTB. Samples were washed five times in PBST and mounted in mounting medium (VECTASHIELD from Vector Laboratories with DAPI; Vector Labs, H-1200). Immunostainings for both experimental and control conditions were carried out on the same slide to enable direct comparisons.

### Fluorescent in situ hybridisation

The following primer pair was used to generate *Vha100-4* probes; F: GAGAGCAACAGCATCTTCCG, R: CAGCACTTGGATCATCTCGC. Probes were labelled using a DIG RNA labelling mix (Roche) following the manufacturer’s guidelines. Universal 3’ T7 primer for antisense probes: AGGGATCCTAATACGACTCACTATAGGGCCCGGGGC, Universal 5’ T7 primer for sense probes: GAGAATTCTAATACGACTCACTATAGGGCCGCGG. Intestinal samples were prepared and fixed as previously reported (Loza-Coll et al, 2014).

### Image acquisition and processing

Confocal fluorescent images were acquired using either an upright Nikon A1 confocal microscope with a 25× Apo dipping objective (NA 1.1) or a spinning disk microscope (CREST V3) on a Nikon Ti2 inverted microscope equipped with a ×60 NA PlanApo 1.4 oil immersion objective using Nis-Elements 5.3 software. Images shown represent maximal intensity projection. The same acquisition settings (laser power and gain/ camera settings) were applied to both experimental and control groups. All statistical analyses were performed on raw 16 bit images using Fiji 2.0. To quantify GFP-positive progenitor cell, a Fiji macro was developed by Dr. Damir Krunic from the DKFZ imaging facility. This macro was used on z-stacked images of the upper epithelial layer of the midgut. For each channel, background subtraction and Gaussian Blur were applied to smooth the cell/area. The threshold for each signal intensity was determined, and a “Find Maxima” code was applied to define the centre of the area or cell. The GFP signal that overlapped with the DAPI channel was quantified. The percentage of GFP cells was calculated as a measure of total DAPI cells. To quantify midgut mitosis, phospho-Histone H3 puncta that overlapped with DAPI nuclei were counted throughout the entire intestine using a Nikon A1 confocal.

### Cph^YFP^ intensity measurements

To measure Cph^YFP^ intensity, progenitor cell nuclei were outlined using the elliptical selection tool in Fiji. The mean grey value within each selection was quantified, and background fluorescence was subtracted. Three nuclei per midgut were analysed, and their values averaged.

### Quantification of puncta

To quantify the number of Atg8a^mCherry^ and Lamp1^mCherry^ puncta, a z-stacked image was taken of the entire progenitor cell, and the multipoint selection tool was used in Fiji to select all mCherry^+^ puncta. Three cells were quantified and averaged in each midgut.

### MARCM clones

To induce clones, adult female flies (2–4 days post-eclosion) were subjected to heat shocks in a 37 °C water bath for 2 days in a row. On the first day, two heat shocks of 30 min each were performed, separated by a 2-h recovery period at 25 °C. On the second day, flies received a single 30-min heat shock at 37 °C. Following clone induction, flies were maintained at 25 °C for 15 days. Flies were evenly housed in control and treatment groups, and their food was changed every 2 days throughout the experiment.

### Lifespan assay

In total, 10–15 adult female flies and five adult male flies were kept in each vial as described in Fly husbandry at 29 °C. All lifespan experiments started with at least 70 adult female flies for each condition, and the number of dead female flies was recorded every day until all female flies were dead.

### Sanger sequencing

Full-length cDNA obtained from scRNA-seq was used as a template for PCR amplification of the Notch cDNA, using forward (CTGGCGTTTGCGAATTTGCCAAA) and reverse (GGATCTTACCAGTGCATGTGTCC) primers flanking both sgRNA target sites. Amplification was carried out with the KAPA HiFi HotStart ReadyMix PCR Kit (Roche - KK2601) using standard PCR condition (annealing temperature: 67 °C; 25 cycles). For Sanger sequencing, the PCR product was analysed using either the forward or reverse primer, enabling coverage of both sgRNA sites. Mutagenesis was assessed based on the appearance of mixed chromatogram peaks and a loss of high-quality sequence signal downstream of the cleavage sites.

### Single-cell RNA-sequencing and high-throughput sequencing

Flies were starved for 3 h and placed in vials with filter paper containing 5% sucrose for 16 h. 20 midguts were dissected from each genotype in ice-cold PBS, taking care to remove the hindgut, Malpighian tubules and proventriculus. Samples were digested in 1 mg/ml Elastase (Sigma, #E0258) solution at 25 °C for 45 min at 1000 RPM and vortexed every 15 min. Samples were then pelleted and resuspended in PBS, and dissociated cells were then passed through a 40 μm then 20 μm cell strainer and subsequently counted. Approximately 20,000 live cells were used for scRNA-seq with 10X Genomics using either the 3’ kit for CRISPR experiments or 5’ kit for *RNAi* experiments following the manufacturers protocol for library generation. Prior to sequencing, library fragment size was determined using an Agilent Bioanalyzer high-sensitivity chip and quantified using Qubit. Libraries were multiplexed and sequenced using a Nextseq 550 at the CCTP, Deep Sequencing Labor, Heidelberg University.

### Single-cell RNA-sequencing data analysis

A CellRanger (version 7.0.1) index was built using the *Drosophila melanogaster* genome sequence along with the corresponding GTF file (Ensembl release 102). To generate single-cell count matrices, reads were aligned to the reference using cellranger count with the include-introns option. Raw gene expression count matrices were pre-processed and filtered using scran (Lun et al, 2016) (version 1.24.0), following the guidelines of Amezquita et al (Amezquita et al, 2020). In brief, droplets with RNA content below each library’s inflection point were removed, along with cells meeting any of the following criteria: (a) fewer than 250 detected genes, (b) ranking in the top 1 percentile of cells by UMI count, or (c) a high percentage of mitochondrial reads. One replicate from each of the *Notch*^*RNAi*^ and *Cph*^*RNAi*^+*Notch*^*RNAi*^ conditions showed an unusually high content of mitochondrial reads and was removed from subsequent analyses. Seurat (version 4.1.1) (Hao et al, 2021) was used for all downstream analyses unless otherwise stated. These analyses included log normalisation of counts, data scaling, cell cycle inference, and identifying the 3000 most variable genes per replicate. Afterwards, the replicates were integrated, and batch effects were corrected using the IntegrateData approach from Seurat. Dimensionality reduction was performed using RunPCA, and UMAPs were constructed using the first 20 principal components. Clustering of the data was performed using the Louvain algorithm(Blondel et al, 2008). Cell type labels were manually assigned to clusters based on the expression of characteristic marker genes(Dutta et al, 2015; Guo et al, 2019; Hung et al, 2020). Subpopulations of EEs were identified by reclustering EEs and analysing marker gene expression (Guo et al, 2019). For differential cell type abundance, we quantified the number of cells per cell type and replicate and modelled the resulting data as a negative binomial distribution using DESeq2 (version 1.36.0) (Love et al, 2014). The size factors were set to be equal to the total number of cells per replicate. Joint expression densities were calculated by the plot_density function from Nebulosa (version 1.8.0) (Alquicira-Hernandez and Powell, 2021).

### Regional mapping of intestinal cells

We downloaded the summary table from the region-specific bulk RNA sequencing dataset (Dutta et al, 2015) to predict the regional origin of our cells using SingleR (v1.10.0) (Aran et al, 2019) with default settings, individually for all cell types, including ISCs, EBs, ECs, and EEs.

### Trajectory differential expression analysis

To reduce the complexity of the dataset for the trajectory inference, we aggregated large flat cells (LFCs) and copper cells into middle enterocytes (mECs) and differentiating anterior enterocytes (daECs) into differentiating enterocytes (dECs). Using 30 principal components as the input, individual trajectories for the control and *Notch*^*sgRNAx2*^ condition were then inferred using slingshot (version 2.4.0) (Street et al, 2018) and condiments (version 1.6.0) (Roux de Bezieux et al, 2024), with ISCs as the starting cluster. The associationTest function from tradeSeq (version 1.13.04) (Van den Berge et al, 2020) was used to determine gene expression changes along a differentiation trajectory, while the conditionTest function was used to identify condition-specific gene expression changes. Gene expression trends along trajectories were generated by local polynomial regression fitting as implemented by the loess function in R.

### MELD analysis

Knockout efficiency was assessed independently for each replicate using MELD (version 1.0.0) (Burkhardt et al, 2021), which calculates the likelihood that a particular cell is from the control or *Notch*^*sgRNAx2*^ condition. To identify the optimal set of parameters for the MELD algorithm, we performed a parameter search using the Benchmarker class as implemented in the MELD package. Ultimately, the algorithm was run using 20 principal components as the input. Subsequently, for each cell type, the determined perturbation likelihoods were used as inputs for vertex frequency clustering (VFC) (Burkhardt et al, 2021) to identify perturbed and unperturbed cell populations.

### Differential expression and gene set enrichment analysis

Where possible, pseudobulk expression profiles were created by aggregating counts of the same cell type across replicates and perturbation status, as inferred by MELD and VFC. Subsequently, expression was compared using muscat (version 1.12.1) and edgeR (version 3.38.0). For experimental conditions with a single replicate, we used Seurat’s FindMarkers function along with the test.use = ”MAST*”* parameter. Gene set enrichment analysis was performed using clusterProfiler (version 4.4.4) (Yu et al, 2012). Gene sets were taken from the FlyEnrichR website (https://maayanlab.cloud/FlyEnrichr/#stats), and the msigdbr package (version 7.5.1) and overrepresentation analysis was performed using the enrichR package (version 3.1) (Kuleshov et al, 2016). To simplify the redundancy of gene ontology terms, they were grouped based on their semantic similarity using the rrvgo package (version 1.10.0) (Sayols, 2023).

### Categorising expression levels

To classify cells based on gene expression levels, we computed the 25th and 75th percentiles for each gene and condition. Cells at or below the 25th percentile were categorised as having “low” expression, while those at or above the 75th percentile were classified as “high.” Additionally, if the 75th percentile of expression was less than 1, any cell with an expression level equal to or greater than 1 was classified as “high.”

### Bulk RNA-seq data analysis

Processed bulk RNA-seq data were downloaded from GEO (GSE102569) (Chen et al, 2018). We removed genes with less than 10 counts in total. Next, we used PyDESeq2 (v0.4.9) (Muzellec et al, 2023) for differential gene expression testing between control and *sc* overexpressed samples using a Wald test.

### ChIP-seq and DamID data analysis

Raw files were acquired via sratools (v3.0.10) from the respective GEO repositories: ChIP-seq: GSE84283 (Li et al, 2017); DamID: GSE211629 (Guo et al, 2022b), and extracted as fastq files. Reads were trimmed using trim_galore (v0.6.10) with settings for Illumina reads, removing 10 bp from both the 5’ and 3’ ends of all reads, and requiring a minimal read length of 36 bp. Trimmed reads were then aligned to the *Drosophila melanogaster* genome using a precompiled bowtie index (BDGP6) and bowtie2 (v2.5.4) (Langmead and Salzberg, 2012) while allowing for maximum single mismatches. Resulting alignment files were filtered for uniquely mapped reads, converted to bam, sorted, and indexed using samtools (v1.20) (Li et al, 2009). We used MACS2 (Gaspar, 2018) for peak calling while filtering out peaks with FDR > 0.05. Peaks were then annotated with CHIPseeker (v1.38.0) (Yu et al, 2015), and a genome annotation file from Ensembl (index BDGP6.46.110). Tracks were plotted using pyGenomeTracks (Lopez-Delisle et al, 2021; Ramirez et al, 2018). The entire workflow was implemented using snakemake (v8.14.0) (Molder et al, 2021).

### Cph NanoDam

To perform Cph NanoDam in the intestine, we crossed a Cph^YFP^; *UAS*-*NanoDam* line with *esg*^*TS*^. As control, we crossed *UAS*-*NanoDam* line with *esg*^*TS*^. Flies were reared at 18 °C. Adult flies aged between 3 and 5 days were shifted to 29 °C for 17 h. 30 Midguts were dissected in ice-cold PBS, taking care to remove Malpighian tubules, hindgut and proventriculus, and samples were frozen in −80 °C. A total of four biological replicates were collected per condition. NanoDam samples were processed as previously described (Tang et al, 2022). DNA was extracted from dissected midgut, and methylated fragments were isolated with DpnI (NEB: R0176S) and DpnII (NEB: R0543S) digestion. Methylated fragments were then amplified with PCR and sonicated in order to generate libraries suitable for sequencing. Sequencing was performed using single-end 86 bp reads using a Nextseq 550 at the CCTP, Deep Sequencing Labor, Heidelberg University. Data has been deposited at GEO repositories: GSE280439. For Cph NanoDam from larval brain tissue, data were obtained from GEO: GSE190210 (Tang et al, 2022).

### NanoDam data analysis

The reads were processed as described for the DamID data. Additionally, a GATC signal was obtained by binning the genome into consecutive GATC sites using a custom Python script and a GFF of sites obtained from damidseq_pipeline (Marshall and Brand, 2015), then calculating fold changes of the NanoDam Cph signal with respect to the control for each replicate, respectively.

### Quantification and statistical analysis

GraphPad Prism 10 software was used for statistical analyses. The statistical tests used for each experiment are indicated in the figure legends. For statistical tests of scRNA-seq and NanoDam dataset, see the corresponding sections in “Methods”. No sample size calculation was performed; sample sizes are not limiting and were determined based on practical considerations and variation between animals. All codes are available at https://github.com/boutroslab/Supp_Redhai_Hirschmueller_2024.

### Additional resources

scRNA-seq Shiny App - https://shiny-portal.embl.de/shinyapps/app/16_IntestiMap.

## Supplementary information

Peer Review File

Source data Fig. 1

Source data Fig. 2

Source data Fig. 3

Source data Fig. 4

Source data Fig. 5

Source data Fig. 7

Source data Fig. 8

Expanded View Figures

## Data Availability

Source data are provided with this manuscript. The scRNA-seq datasets generated in this study are available from the following databases: scRNA-seq data: Gene Expression Omnibus: GSE276185. A processed version of scRNA-seq data is available at 10.5281/zenodo.17716158. NanoDam data: Gene Expression Omnibus: GSE280439. All codes used in this study are available at: https://github.com/boutroslab/Supp_Redhai_Hirschmueller_2024. The source data of this paper are collected in the following database record: biostudies:S-SCDT-10_1038-S44318-026-00808-x.
