# Supplementary figures and images for: Autoinhibitory feedback preserves intestinal stem cell maintenance and fate commitment
Source: EMBO J. 2026 May 20;45(13):4636–69. doi: 10.1038/s44318-026-00808-x (PMC13324515; doi:10.1038/s44318-026-00808-x)

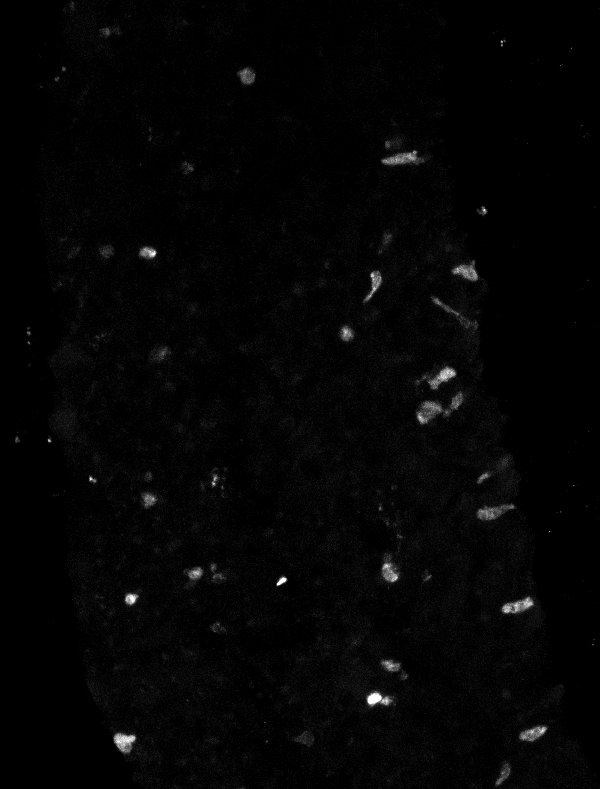

Supplement: Supplementary file 2 — Source data Fig. 1 [file 44318_2026_808_MOESM2_ESM.zip › Fig.1/Panel D/control AstC.jpg]

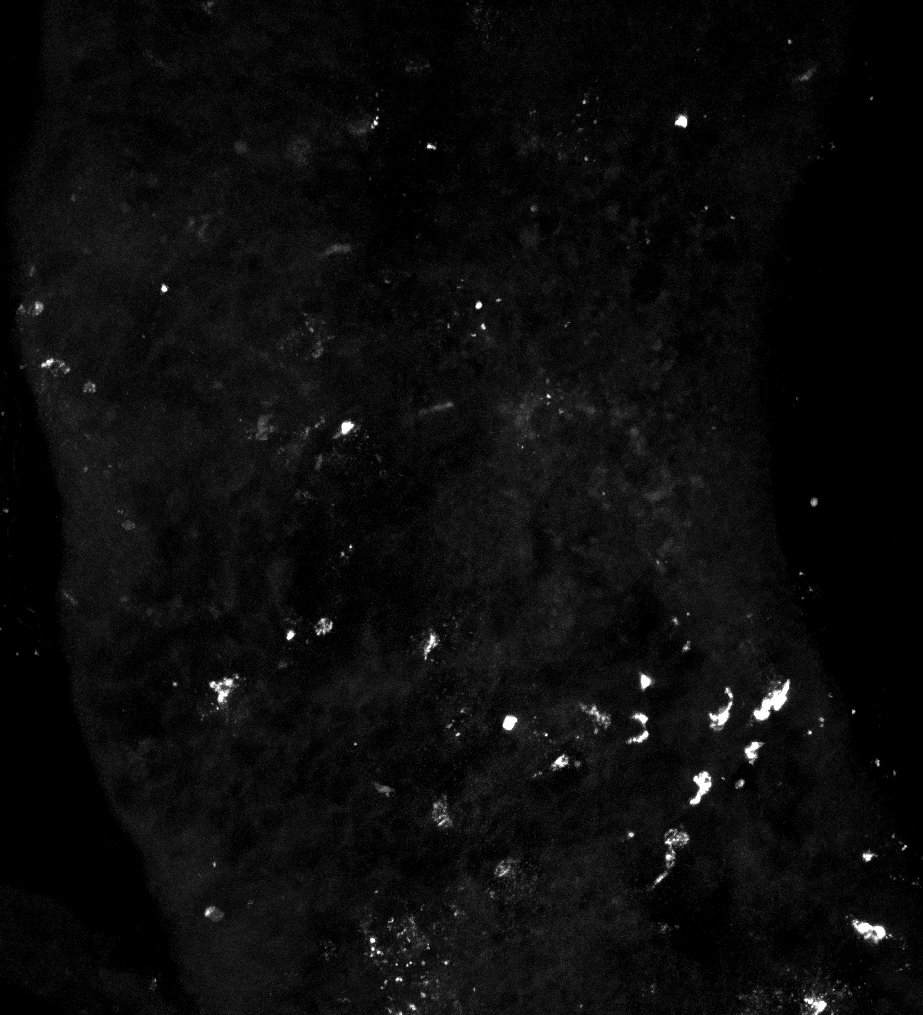

Supplement: Supplementary file 2 — Source data Fig. 1 [file 44318_2026_808_MOESM2_ESM.zip › Fig.1/Panel D/Notch sgRNAx2 TK.jpg]

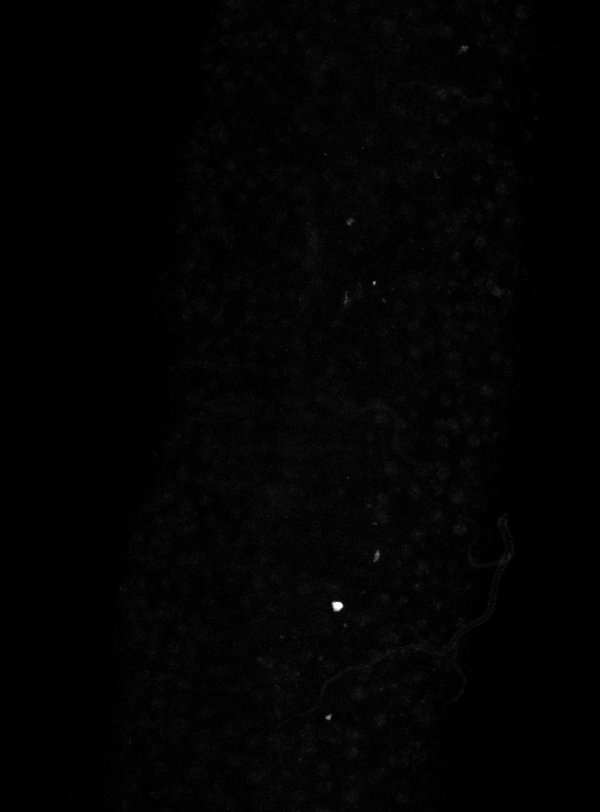

Supplement: Supplementary file 2 — Source data Fig. 1 [file 44318_2026_808_MOESM2_ESM.zip › Fig.1/Panel D/control PH3.jpg]

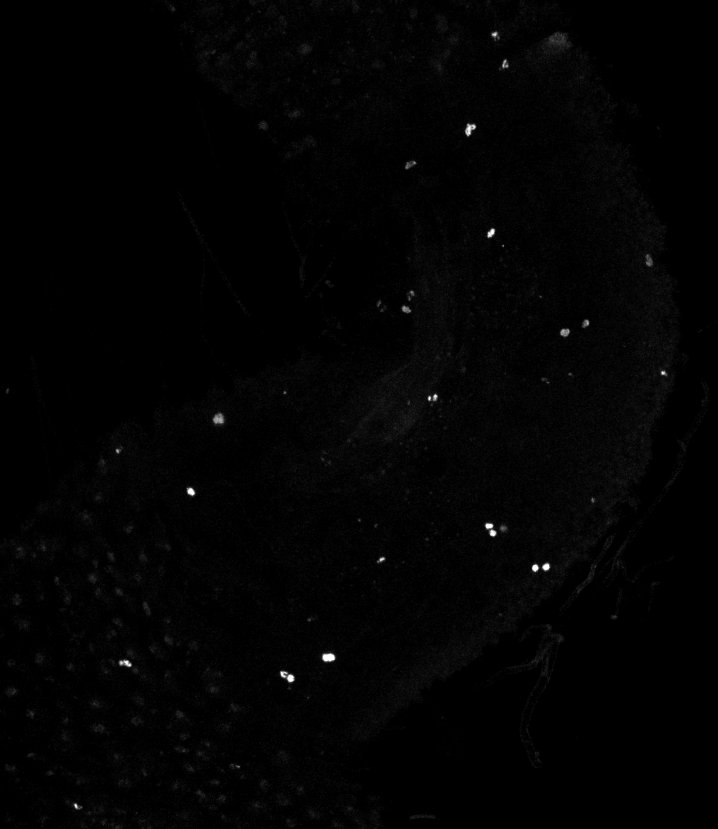

Supplement: Supplementary file 2 — Source data Fig. 1 [file 44318_2026_808_MOESM2_ESM.zip › Fig.1/Panel D/Notch sgRNAx2 PH3.jpg]

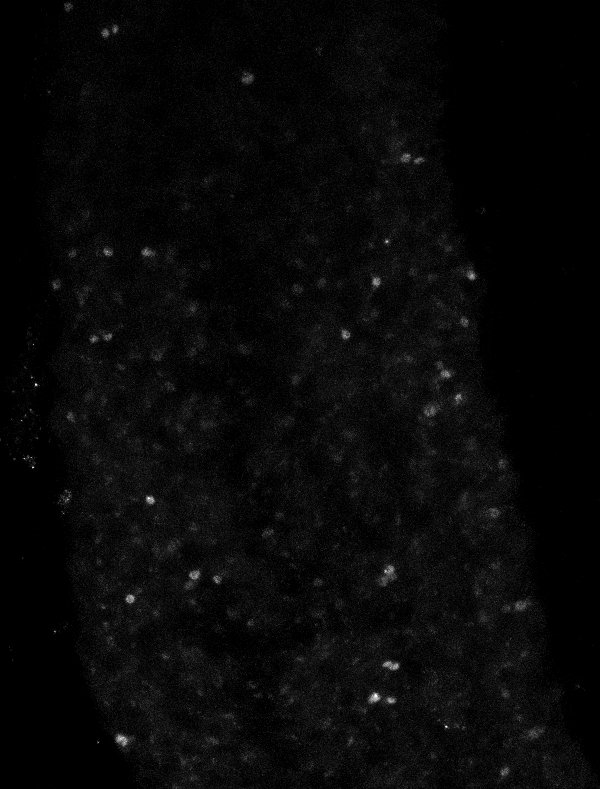

Supplement: Supplementary file 2 — Source data Fig. 1 [file 44318_2026_808_MOESM2_ESM.zip › Fig.1/Panel D/control Pros.jpg]

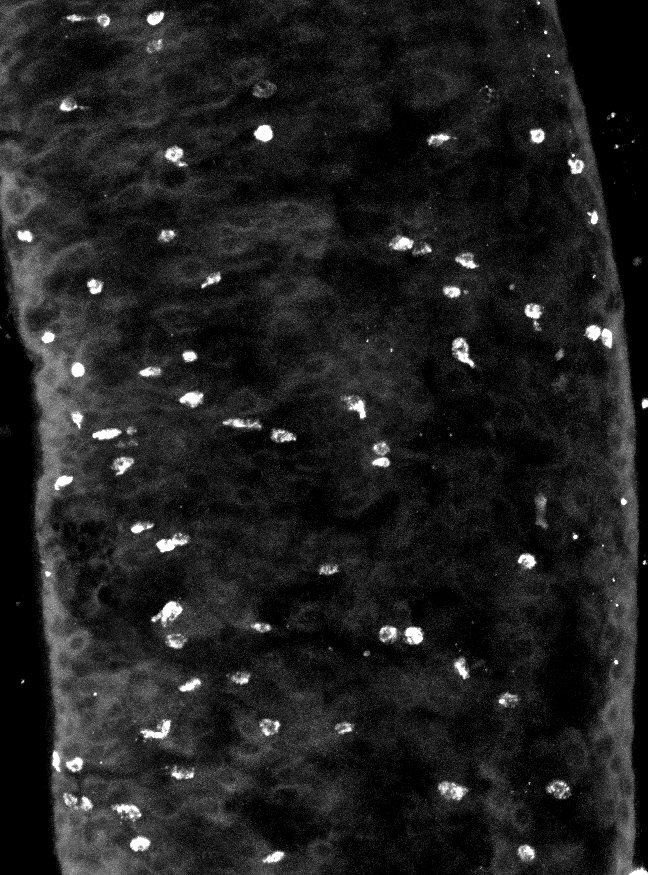

Supplement: Supplementary file 2 — Source data Fig. 1 [file 44318_2026_808_MOESM2_ESM.zip › Fig.1/Panel D/control TK.jpg]

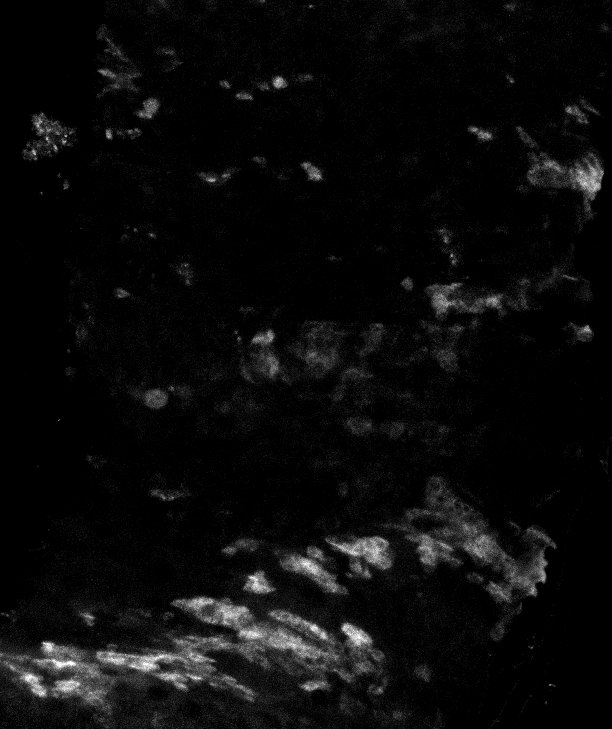

Supplement: Supplementary file 2 — Source data Fig. 1 [file 44318_2026_808_MOESM2_ESM.zip › Fig.1/Panel D/Notch sgRNAx2 AstC.jpg]

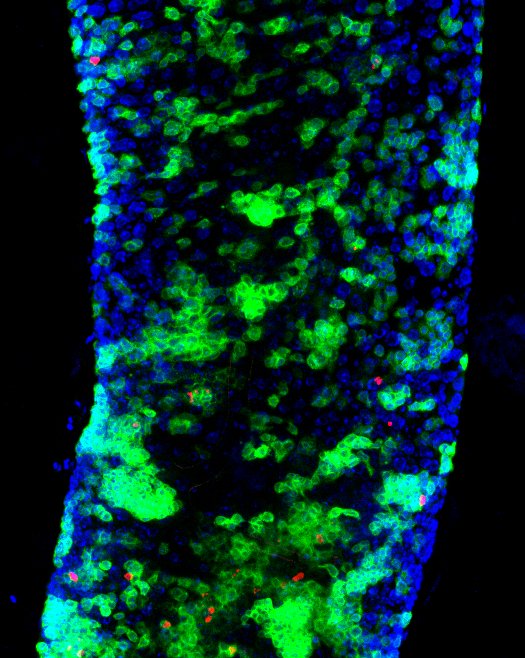

Supplement: Supplementary file 2 — Source data Fig. 1 [file 44318_2026_808_MOESM2_ESM.zip › Fig.1/Panel D/Notch sgRNAx2 merged.jpg]

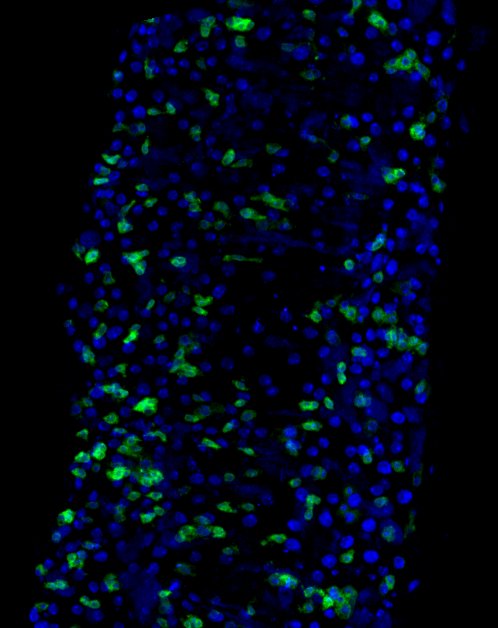

Supplement: Supplementary file 2 — Source data Fig. 1 [file 44318_2026_808_MOESM2_ESM.zip › Fig.1/Panel D/control merged.jpg]

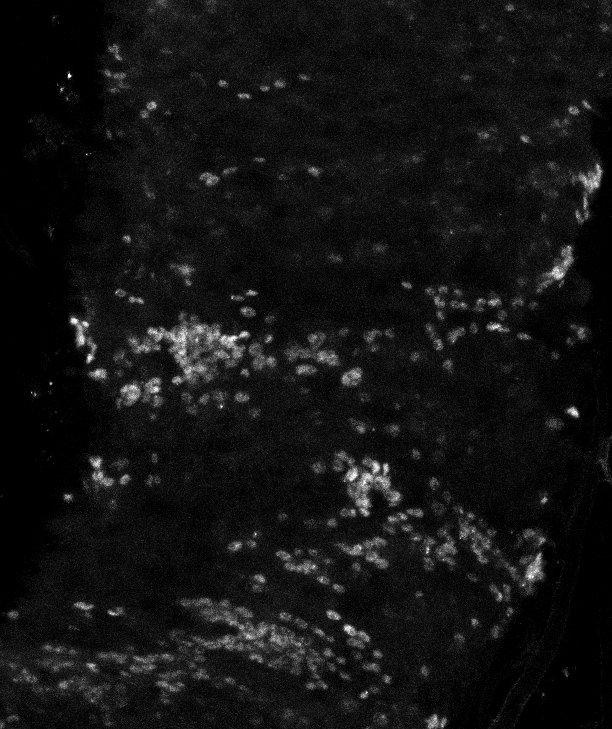

Supplement: Supplementary file 2 — Source data Fig. 1 [file 44318_2026_808_MOESM2_ESM.zip › Fig.1/Panel D/Notch sgRNAx2 Pros.jpg]

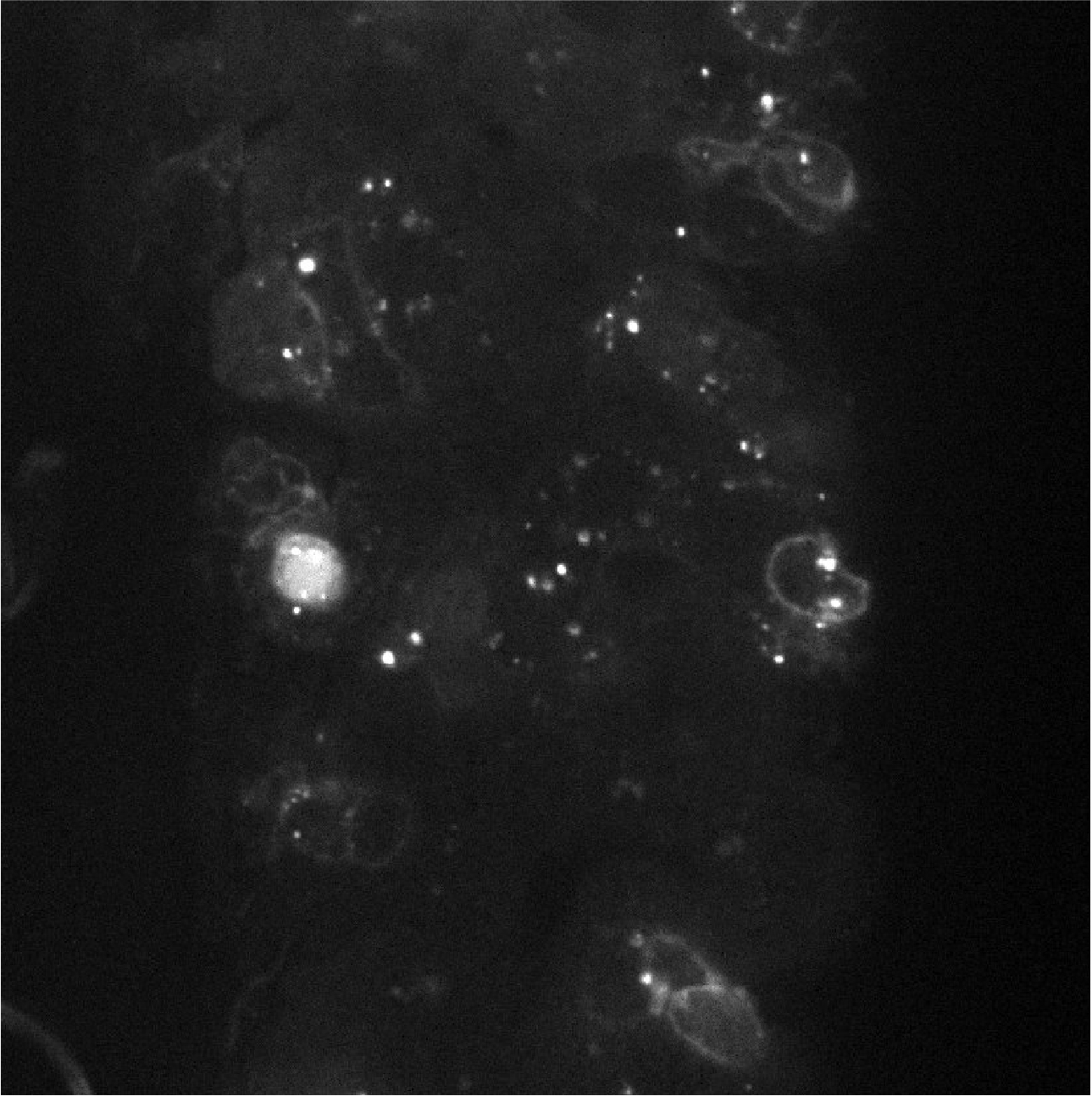

Supplement: Supplementary file 3 — Source data Fig. 2 [file 44318_2026_808_MOESM3_ESM.zip › Fig.2/Panel B/RFP.jpg]

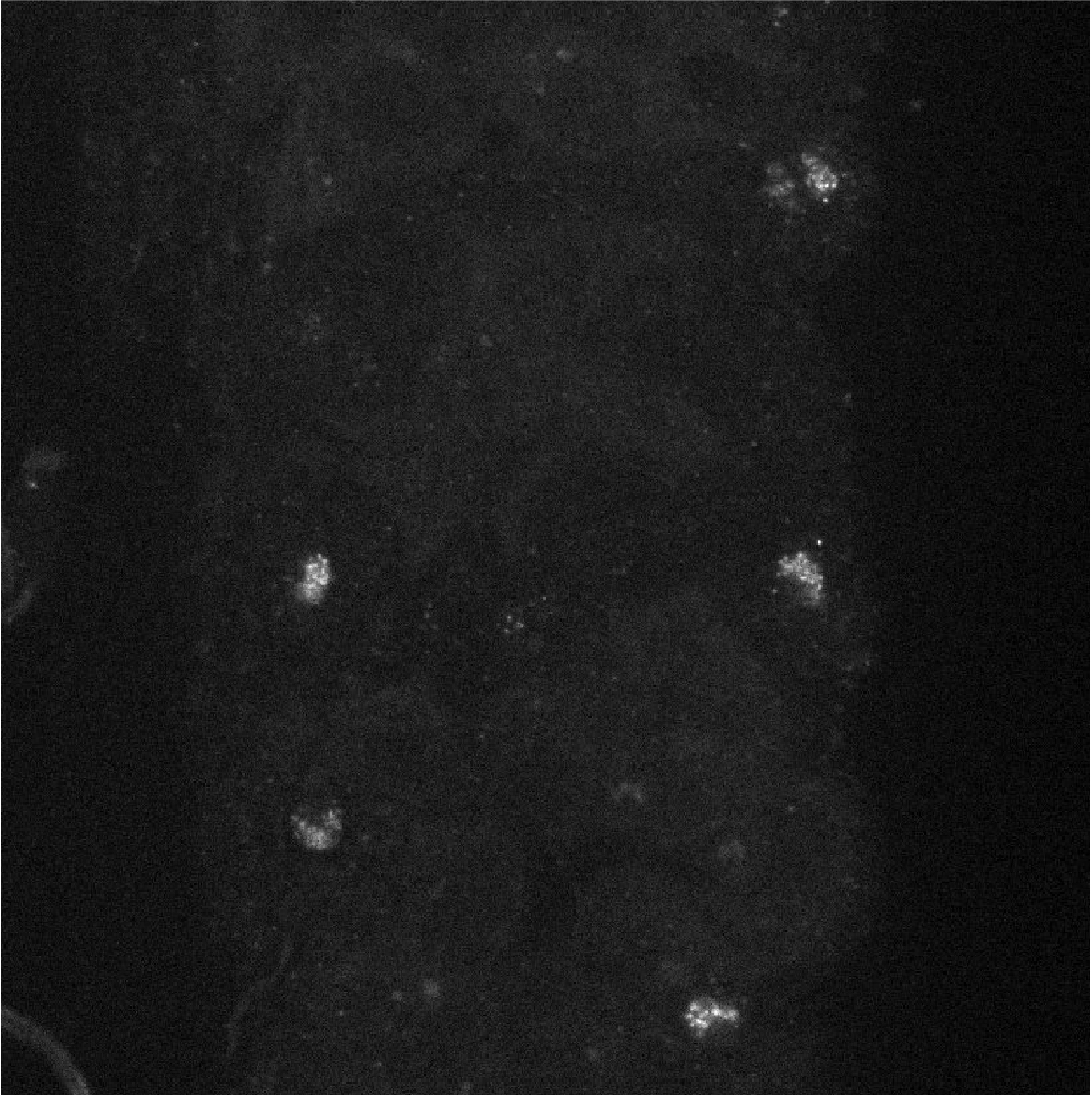

Supplement: Supplementary file 3 — Source data Fig. 2 [file 44318_2026_808_MOESM3_ESM.zip › Fig.2/Panel B/Cph YFP.jpg]

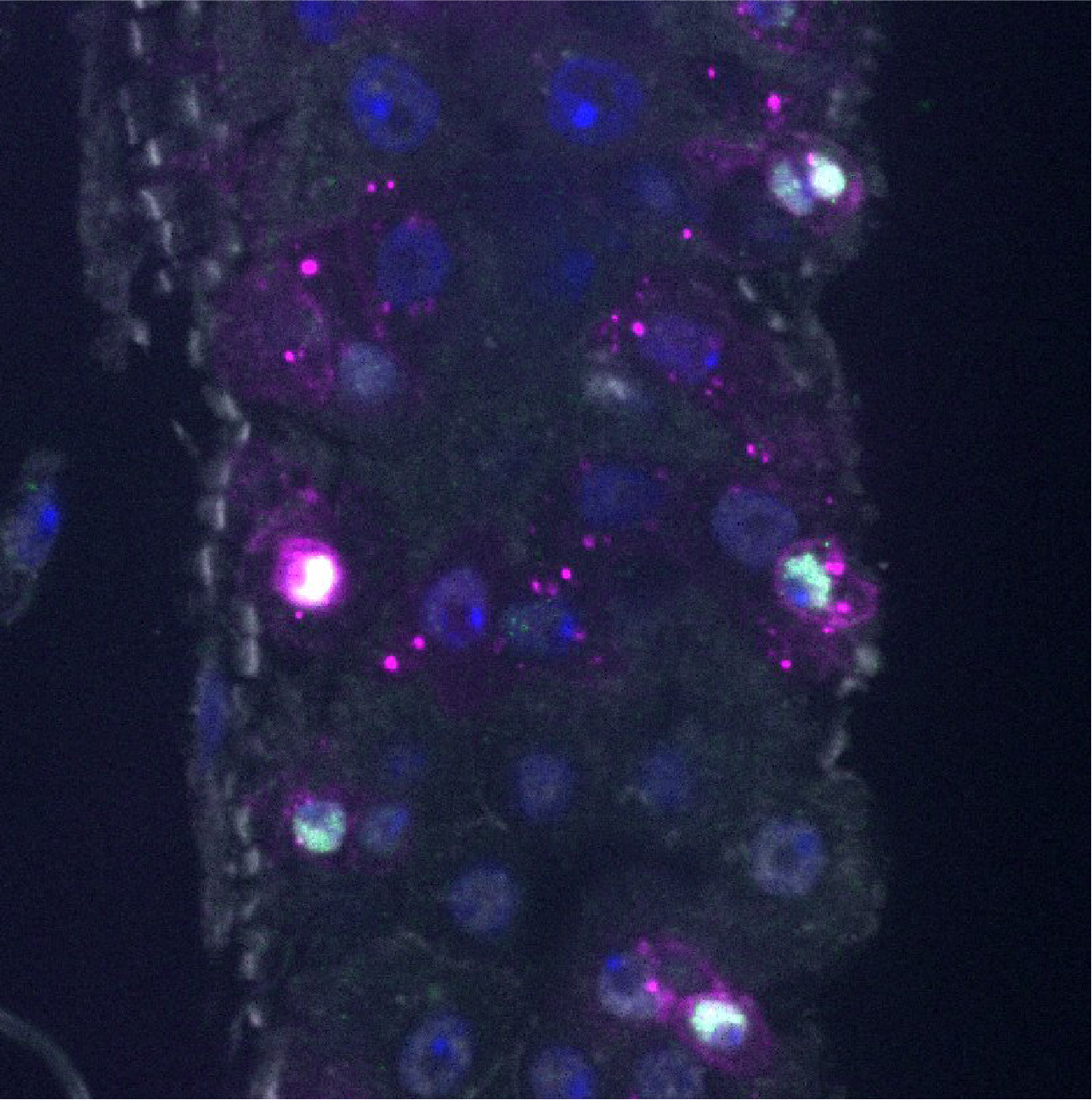

Supplement: Supplementary file 3 — Source data Fig. 2 [file 44318_2026_808_MOESM3_ESM.zip › Fig.2/Panel B/Merged.jpg]

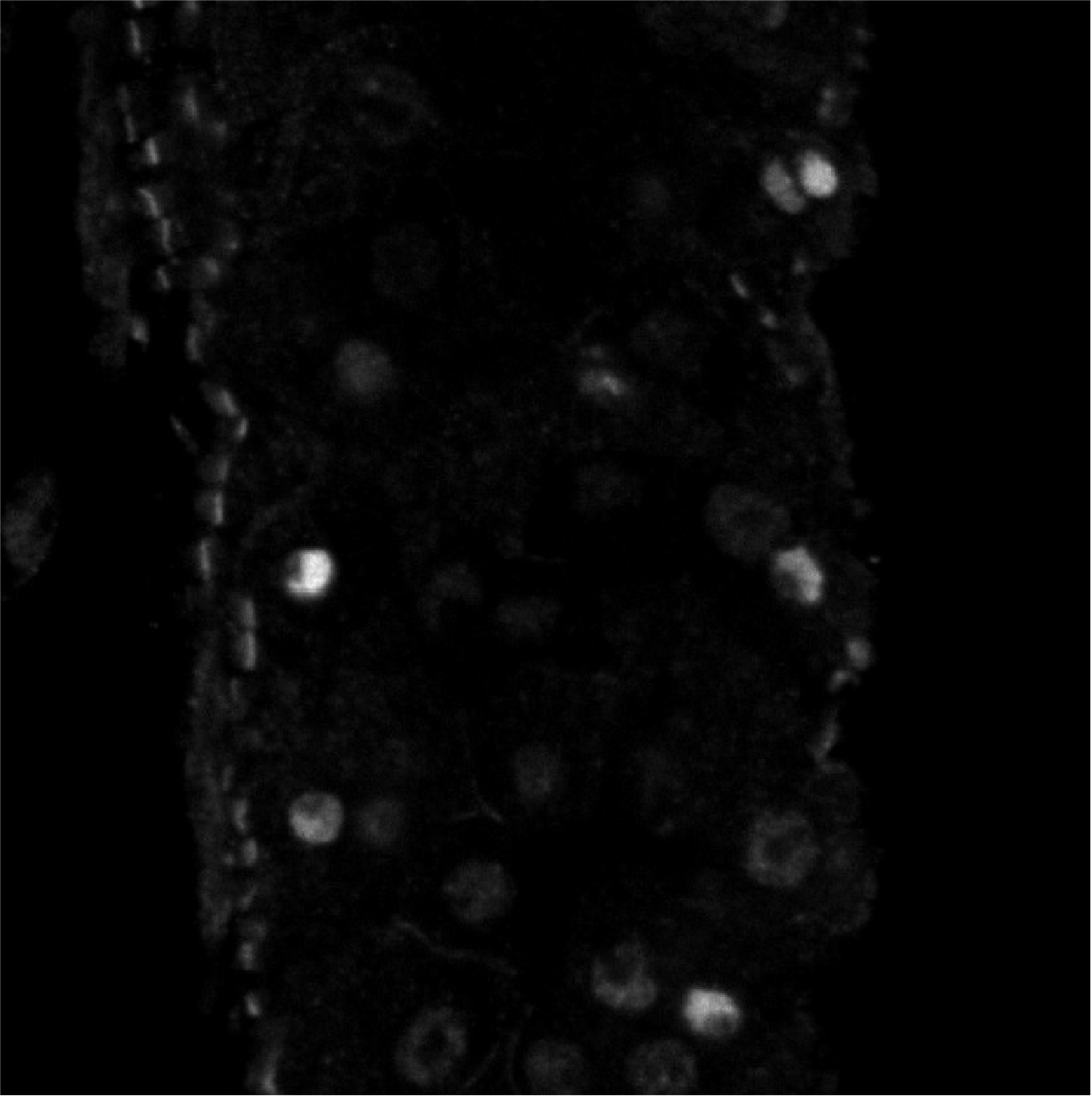

Supplement: Supplementary file 3 — Source data Fig. 2 [file 44318_2026_808_MOESM3_ESM.zip › Fig.2/Panel B/LacZ.jpg]

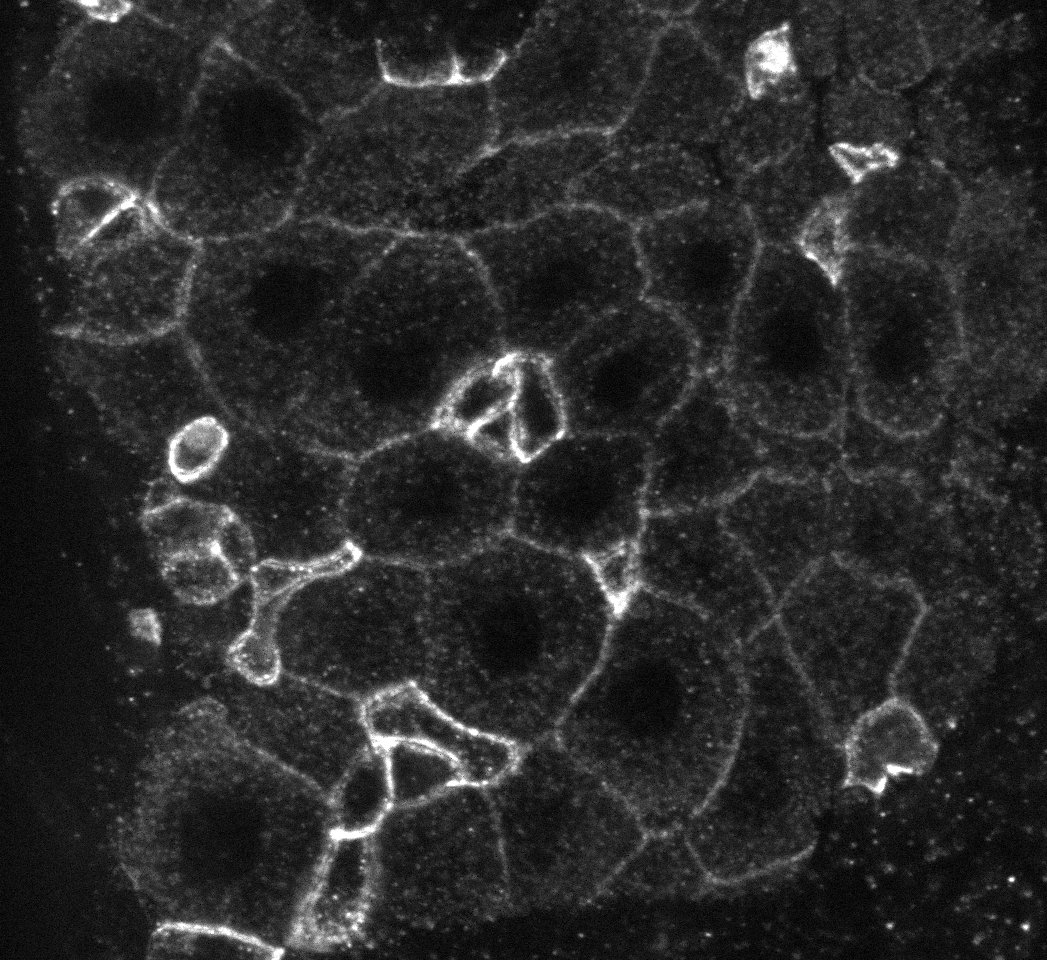

Supplement: Supplementary file 3 — Source data Fig. 2 [file 44318_2026_808_MOESM3_ESM.zip › Fig.2/Panel D/control Arm:Pros.jpg]

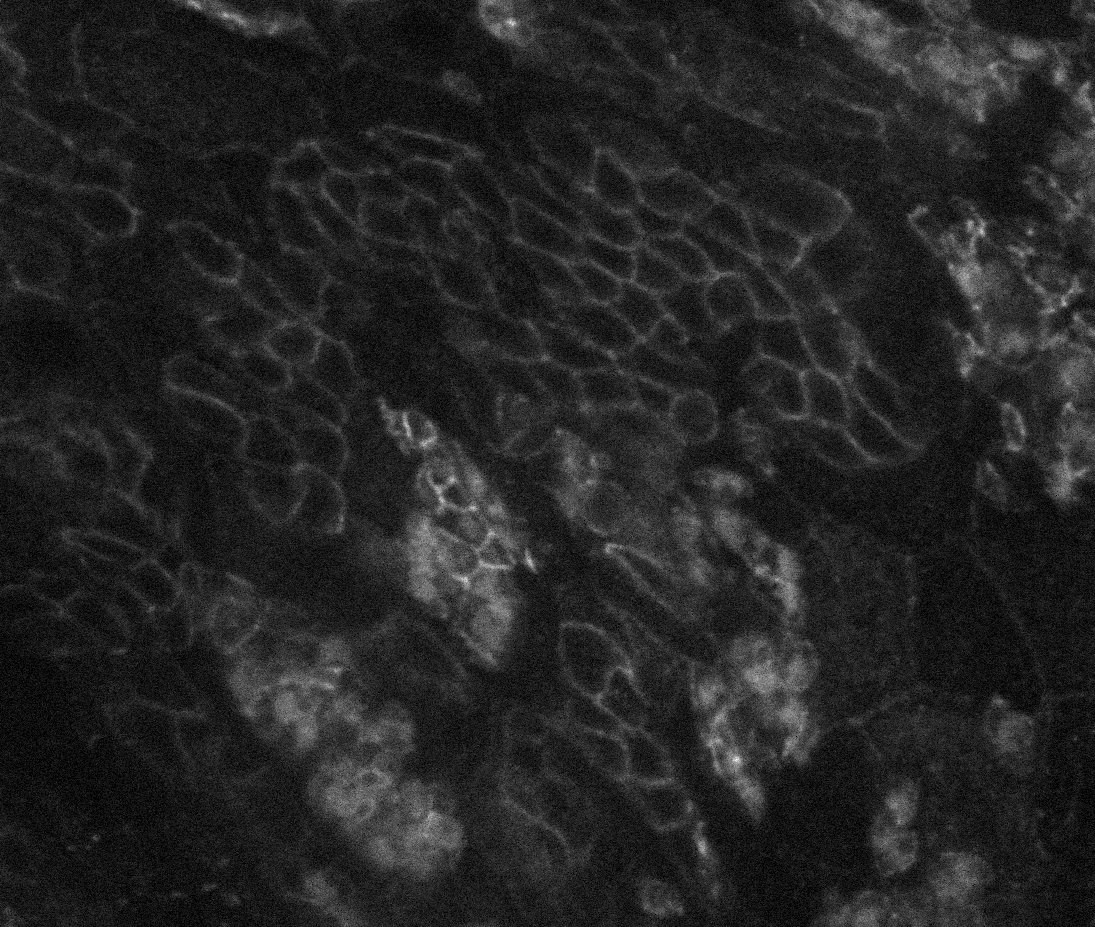

Supplement: Supplementary file 3 — Source data Fig. 2 [file 44318_2026_808_MOESM3_ESM.zip › Fig.2/Panel D/Notch RNAi Arm:Pros.jpg]

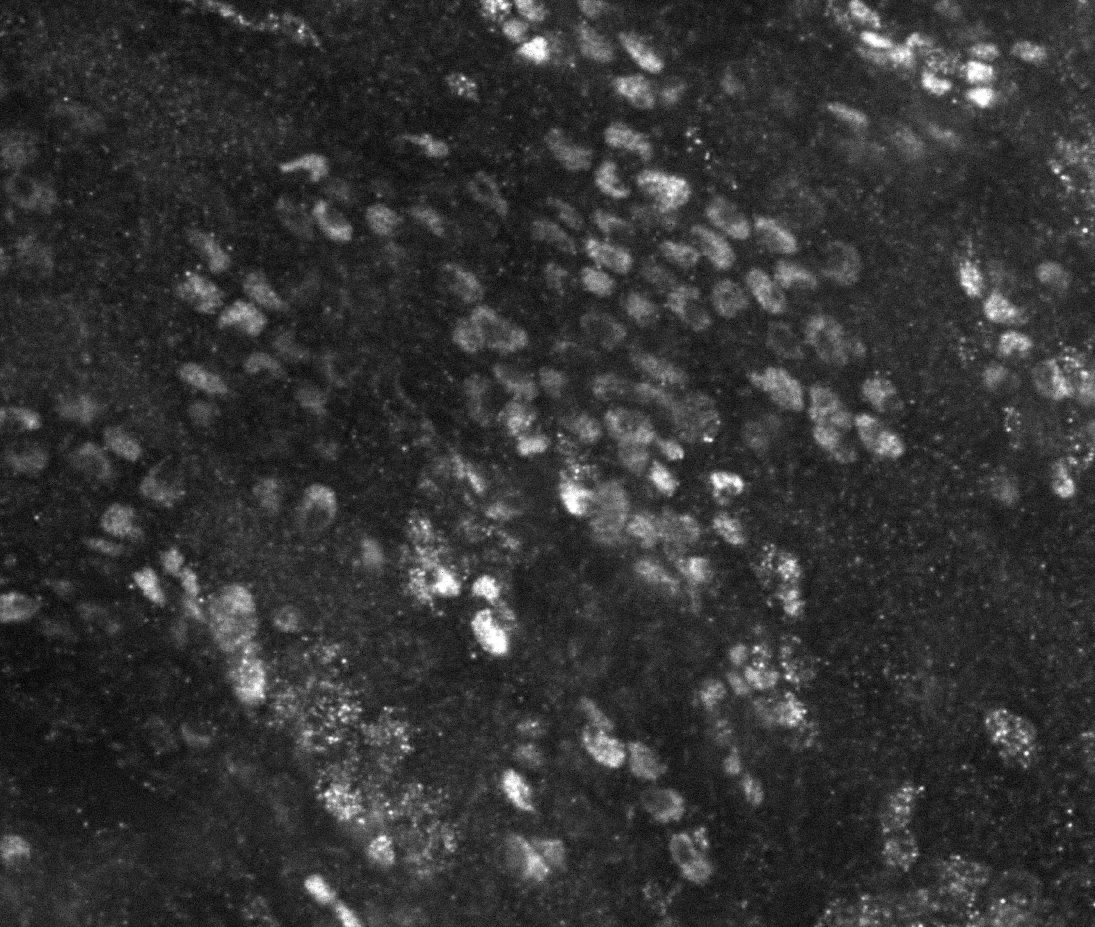

Supplement: Supplementary file 3 — Source data Fig. 2 [file 44318_2026_808_MOESM3_ESM.zip › Fig.2/Panel D/Notch RNAi Cph-YFP.jpg]

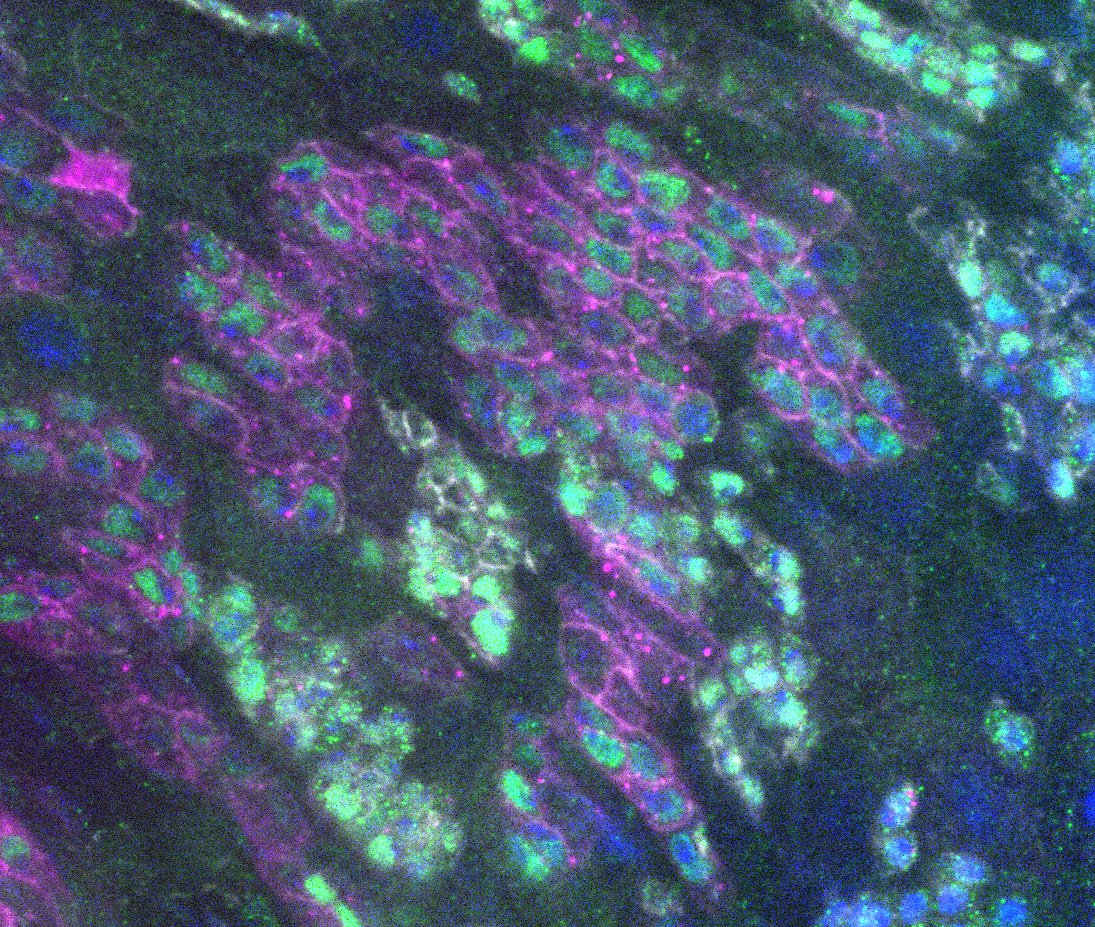

Supplement: Supplementary file 3 — Source data Fig. 2 [file 44318_2026_808_MOESM3_ESM.zip › Fig.2/Panel D/Notch RNAi merged.jpg]

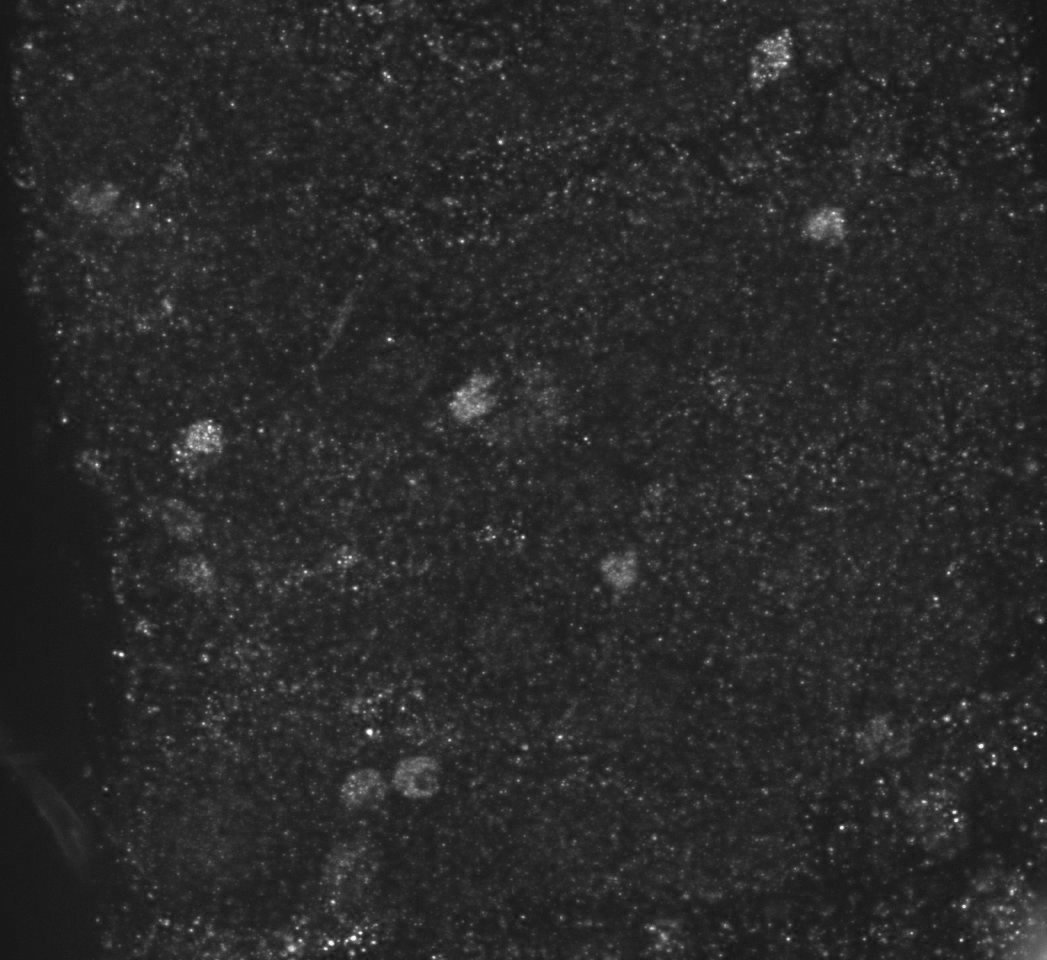

Supplement: Supplementary file 3 — Source data Fig. 2 [file 44318_2026_808_MOESM3_ESM.zip › Fig.2/Panel D/control Cph-YFP.jpg]

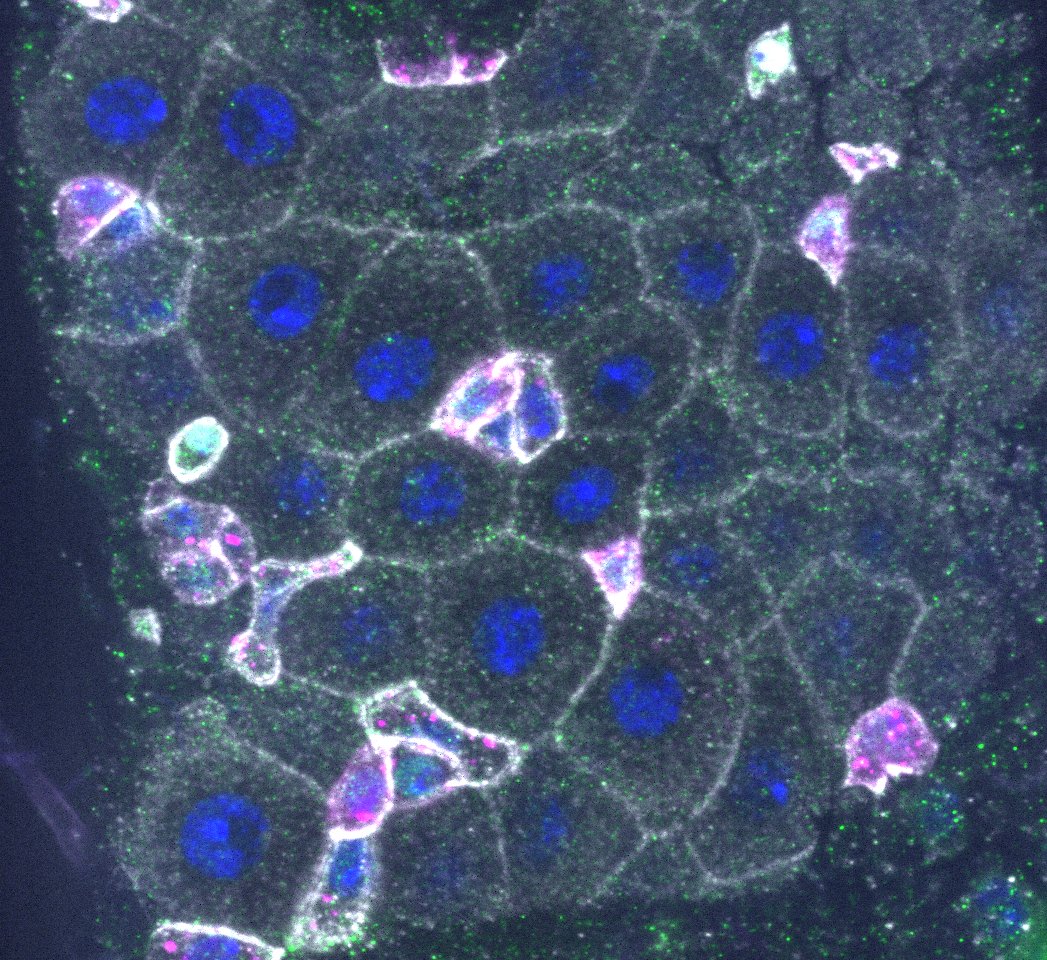

Supplement: Supplementary file 3 — Source data Fig. 2 [file 44318_2026_808_MOESM3_ESM.zip › Fig.2/Panel D/control merged.jpg]

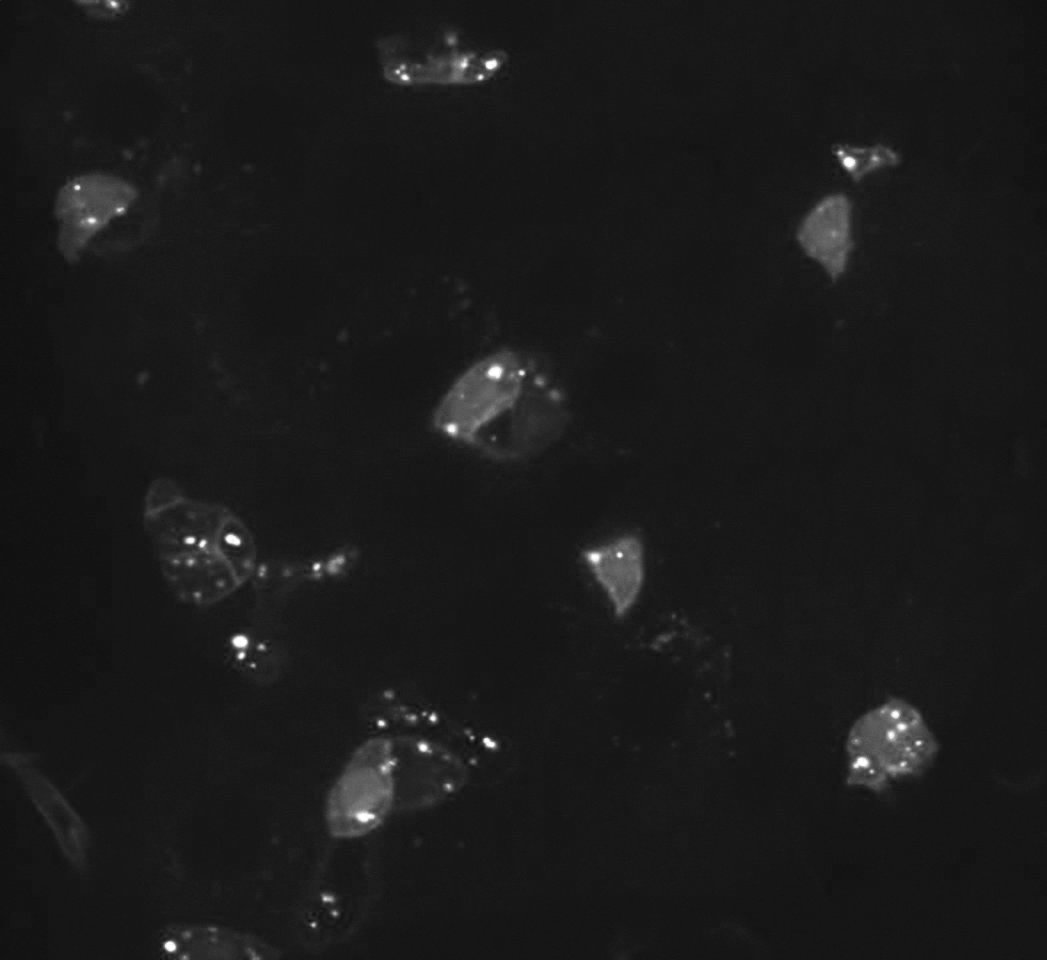

Supplement: Supplementary file 3 — Source data Fig. 2 [file 44318_2026_808_MOESM3_ESM.zip › Fig.2/Panel D/control RFP.jpg]

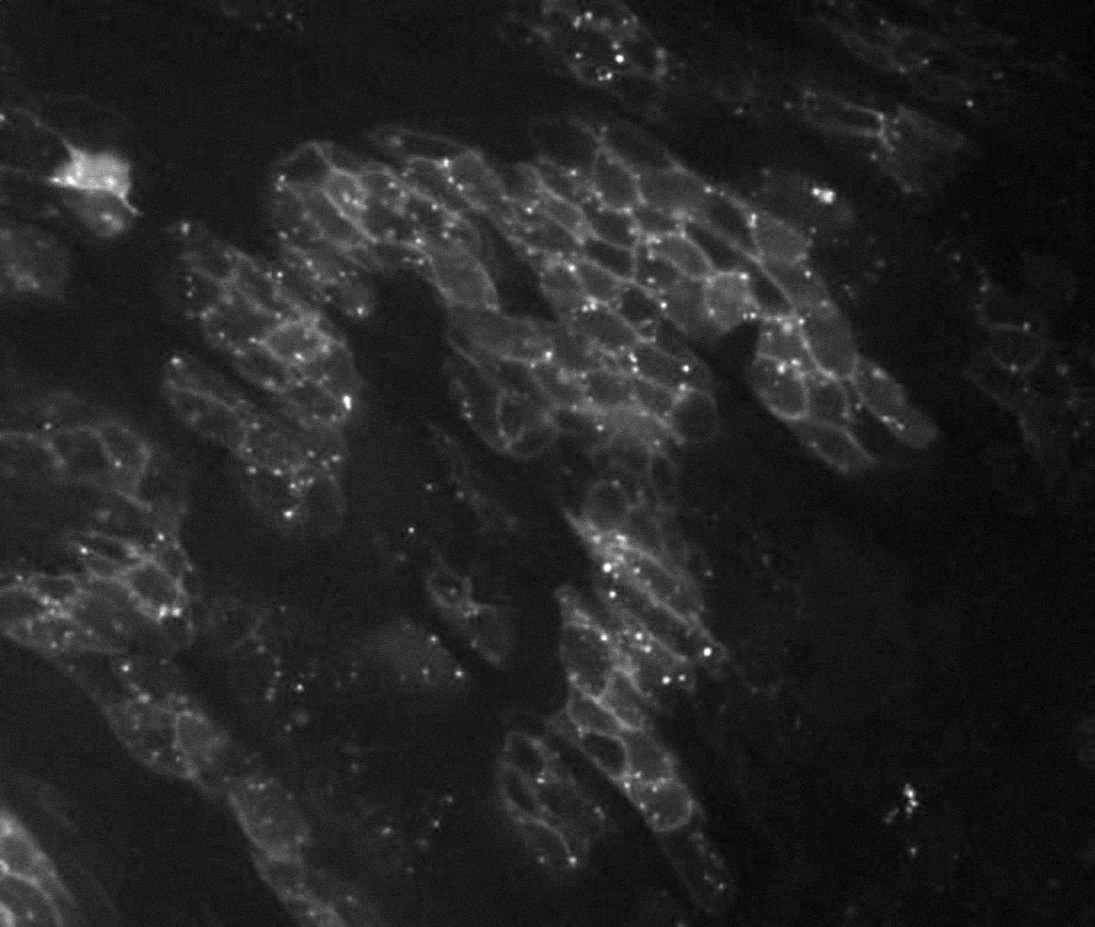

Supplement: Supplementary file 3 — Source data Fig. 2 [file 44318_2026_808_MOESM3_ESM.zip › Fig.2/Panel D/Notch RNAi RFP.jpg]

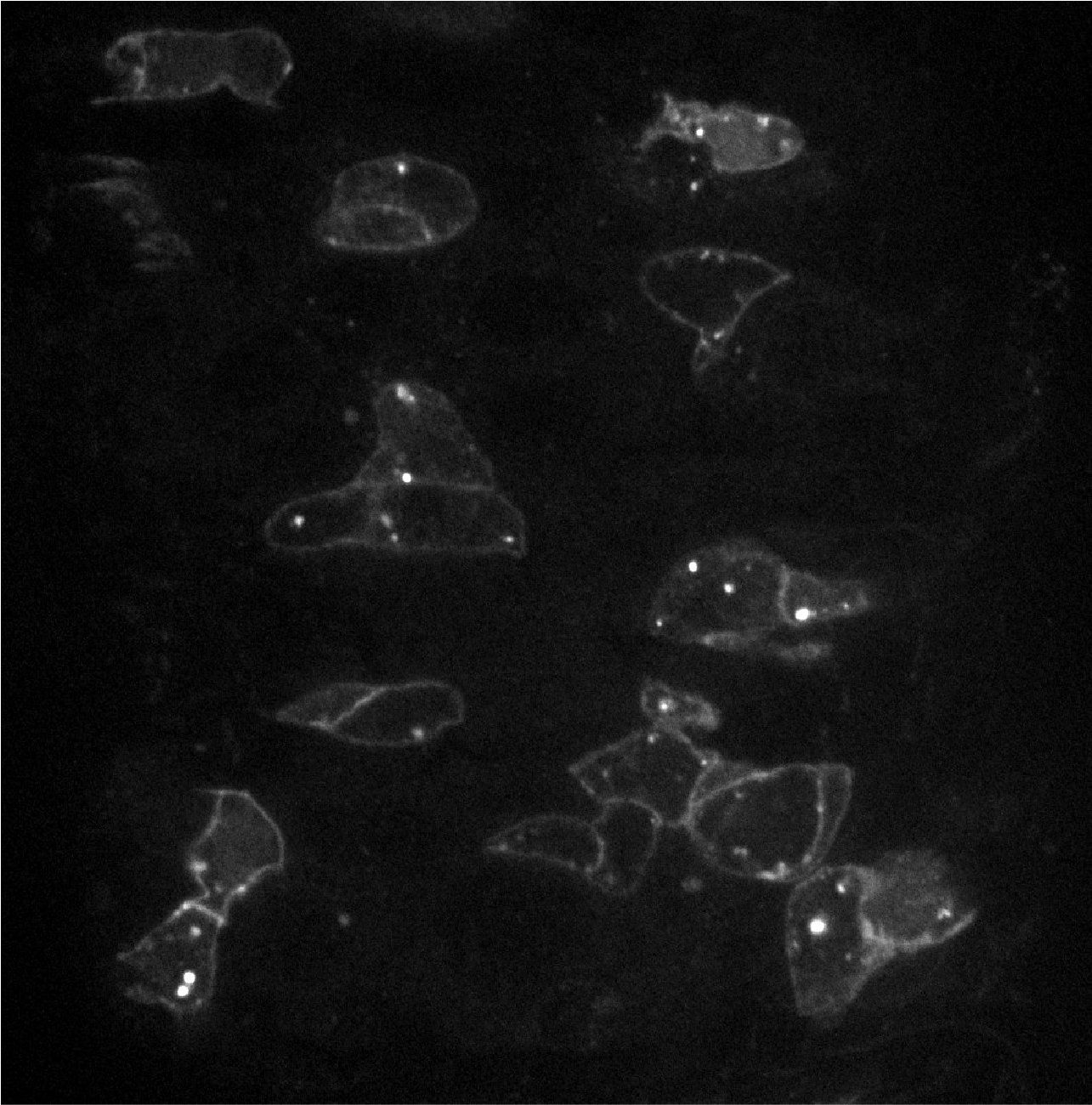

Supplement: Supplementary file 3 — Source data Fig. 2 [file 44318_2026_808_MOESM3_ESM.zip › Fig.2/Panel C/RFP.jpg]

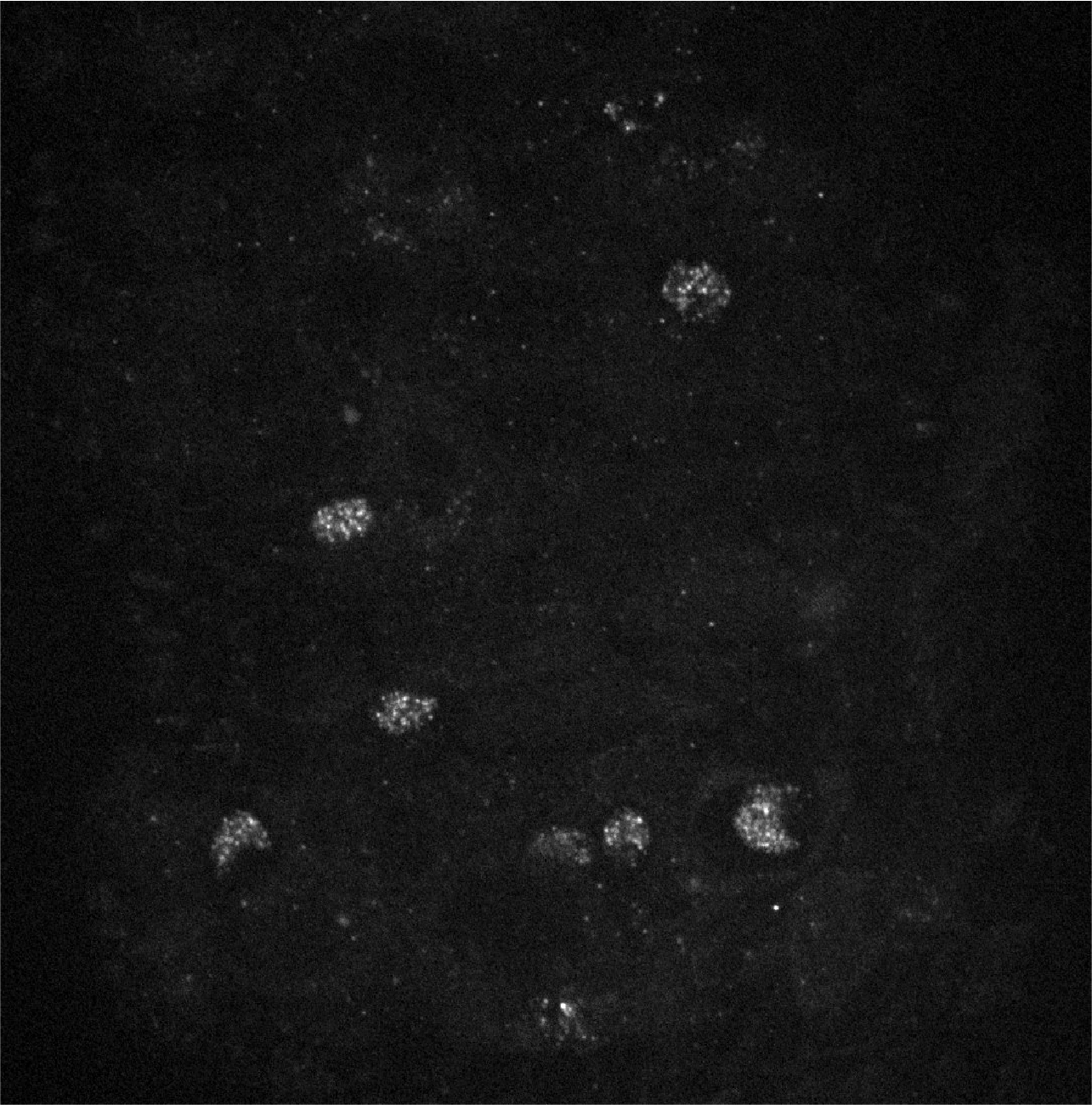

Supplement: Supplementary file 3 — Source data Fig. 2 [file 44318_2026_808_MOESM3_ESM.zip › Fig.2/Panel C/Cph YFP.jpg]

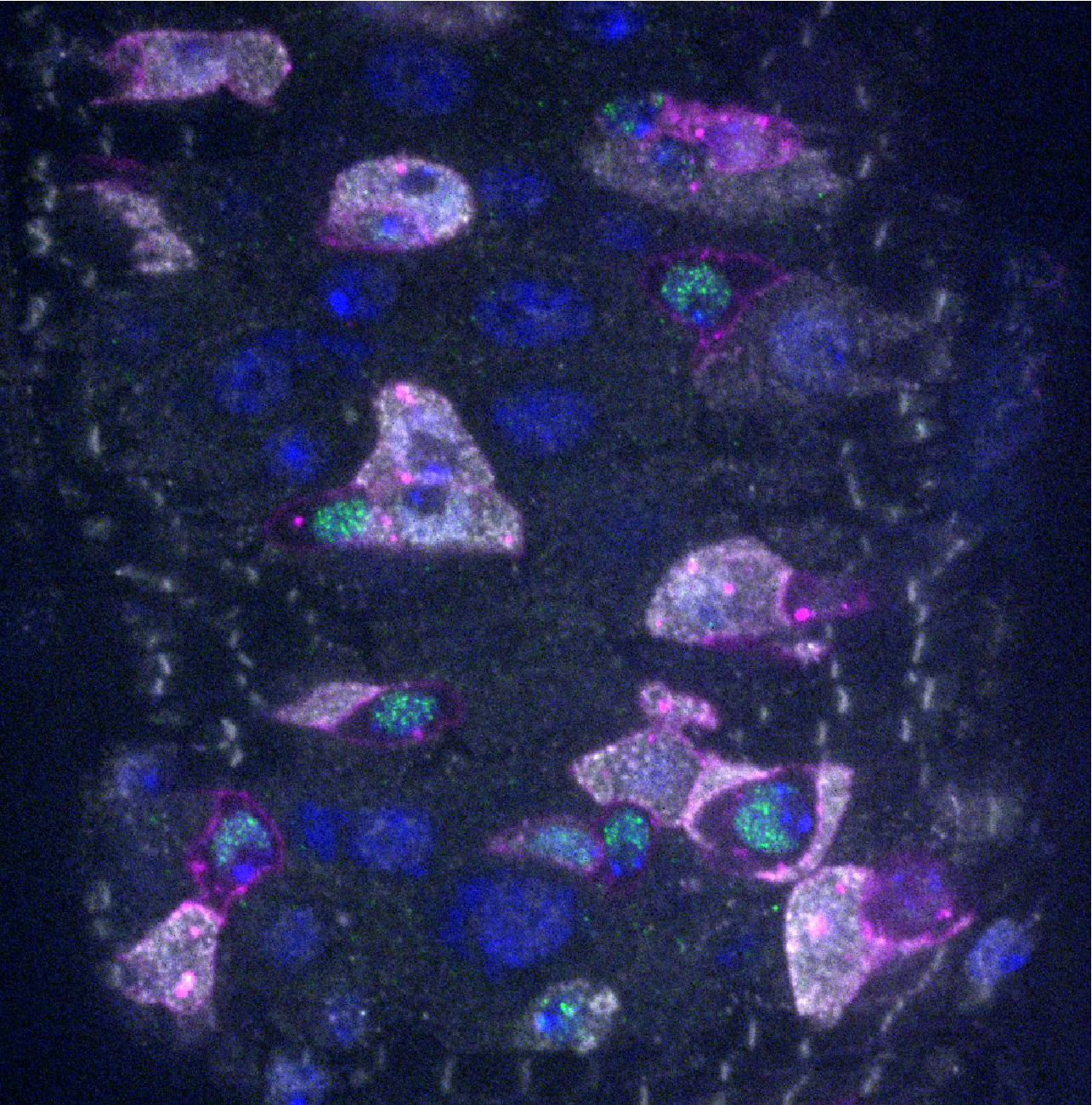

Supplement: Supplementary file 3 — Source data Fig. 2 [file 44318_2026_808_MOESM3_ESM.zip › Fig.2/Panel C/Merged.jpg]

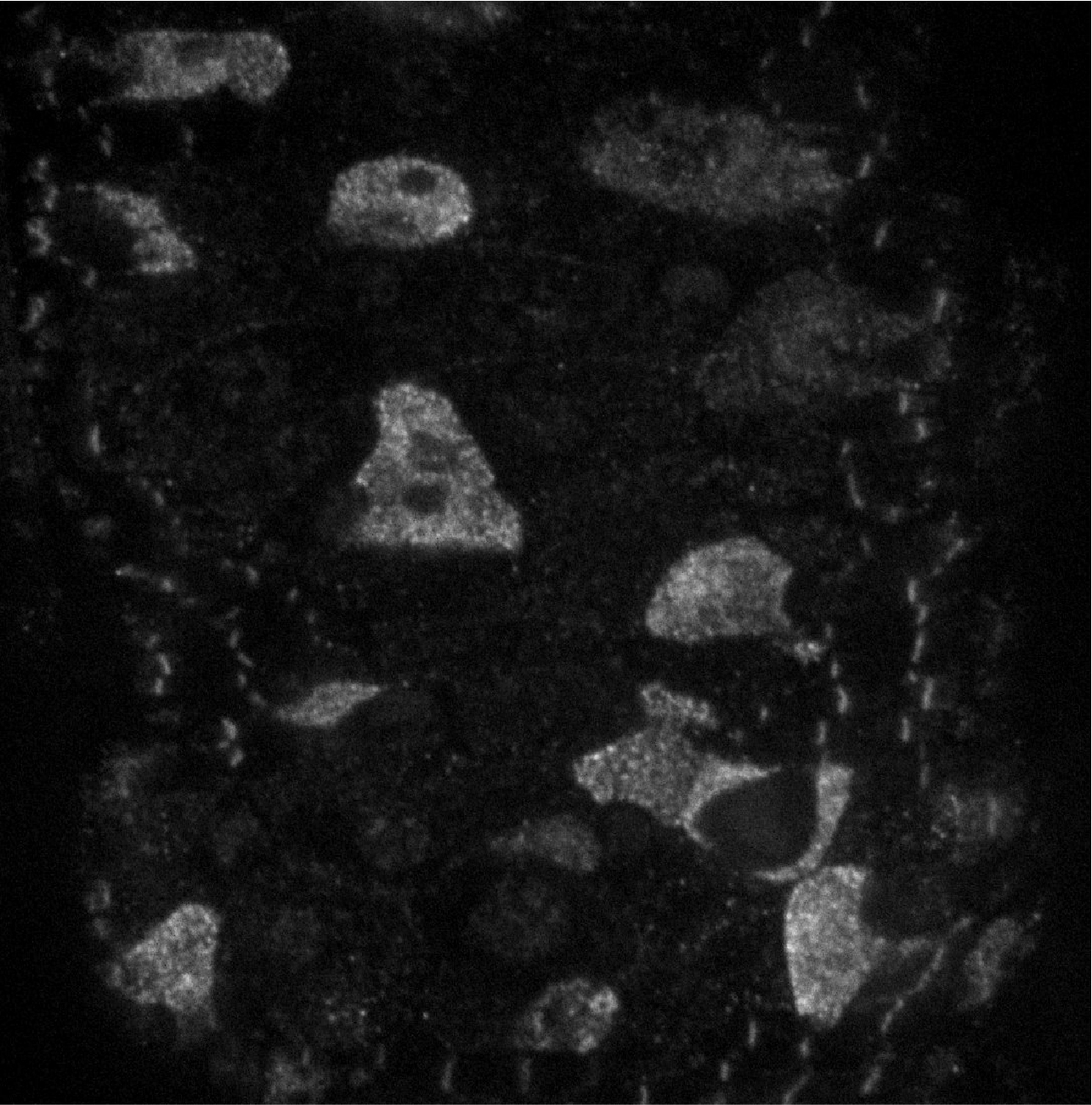

Supplement: Supplementary file 3 — Source data Fig. 2 [file 44318_2026_808_MOESM3_ESM.zip › Fig.2/Panel C/LacZ.jpg]

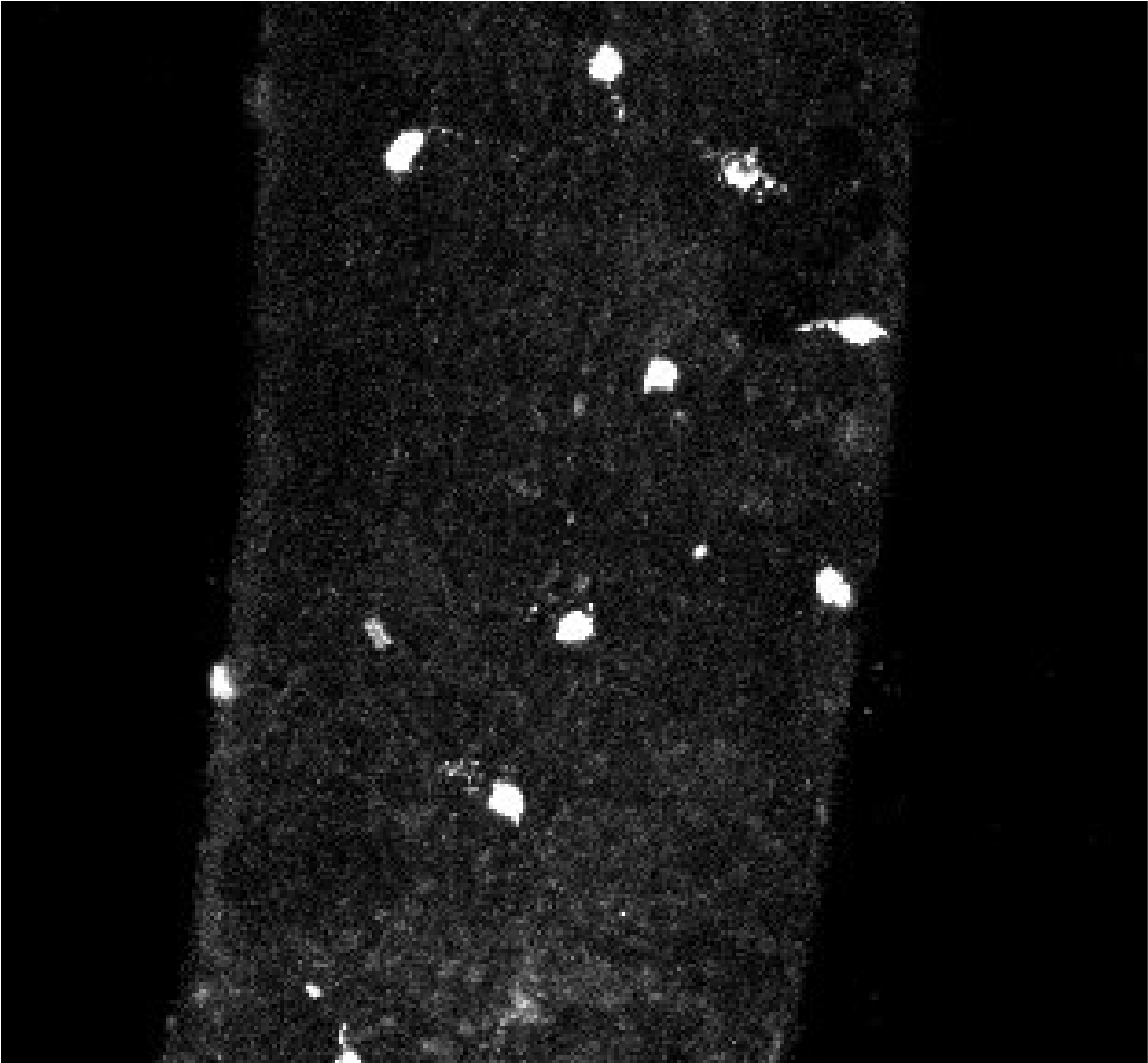

Supplement: Supplementary file 4 — Source data Fig. 3 [file 44318_2026_808_MOESM4_ESM.zip › Fig.3/Panel C/Cph RNAi#1 + Notch RNAi - GFP PH3.jpg]

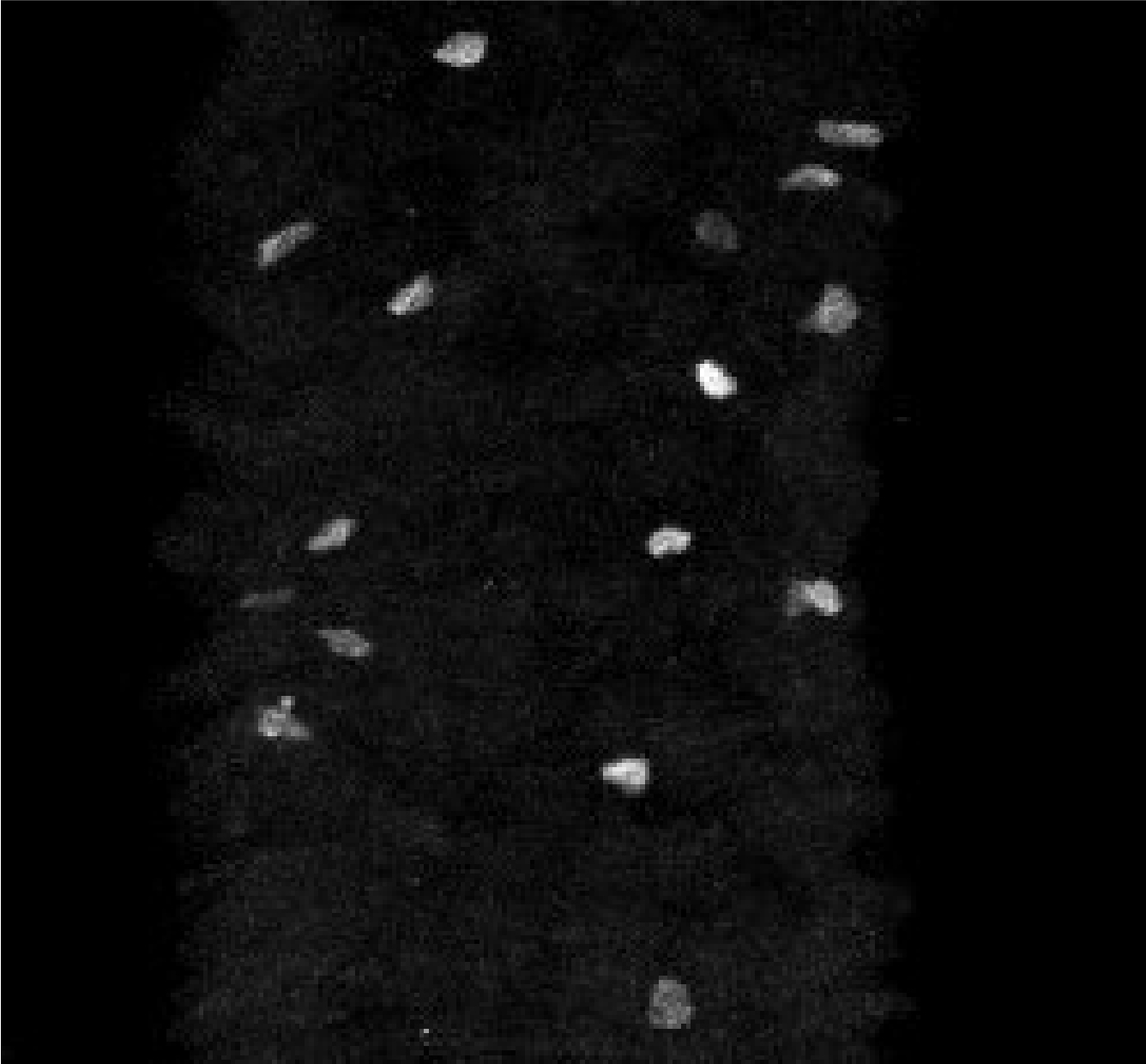

Supplement: Supplementary file 4 — Source data Fig. 3 [file 44318_2026_808_MOESM4_ESM.zip › Fig.3/Panel C/Control - AstC.jpg]

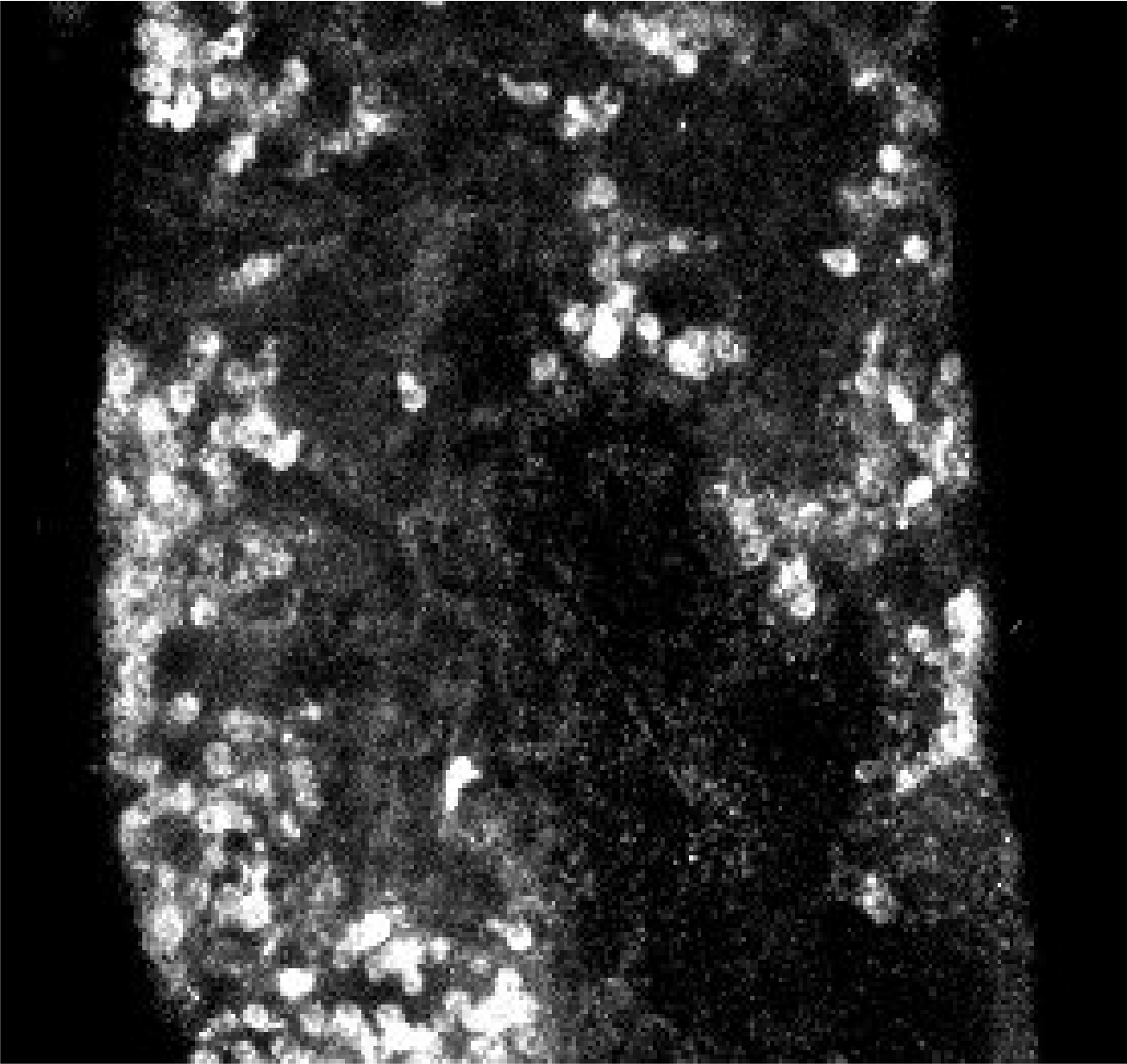

Supplement: Supplementary file 4 — Source data Fig. 3 [file 44318_2026_808_MOESM4_ESM.zip › Fig.3/Panel C/Notch RNAi - AstC.jpg]

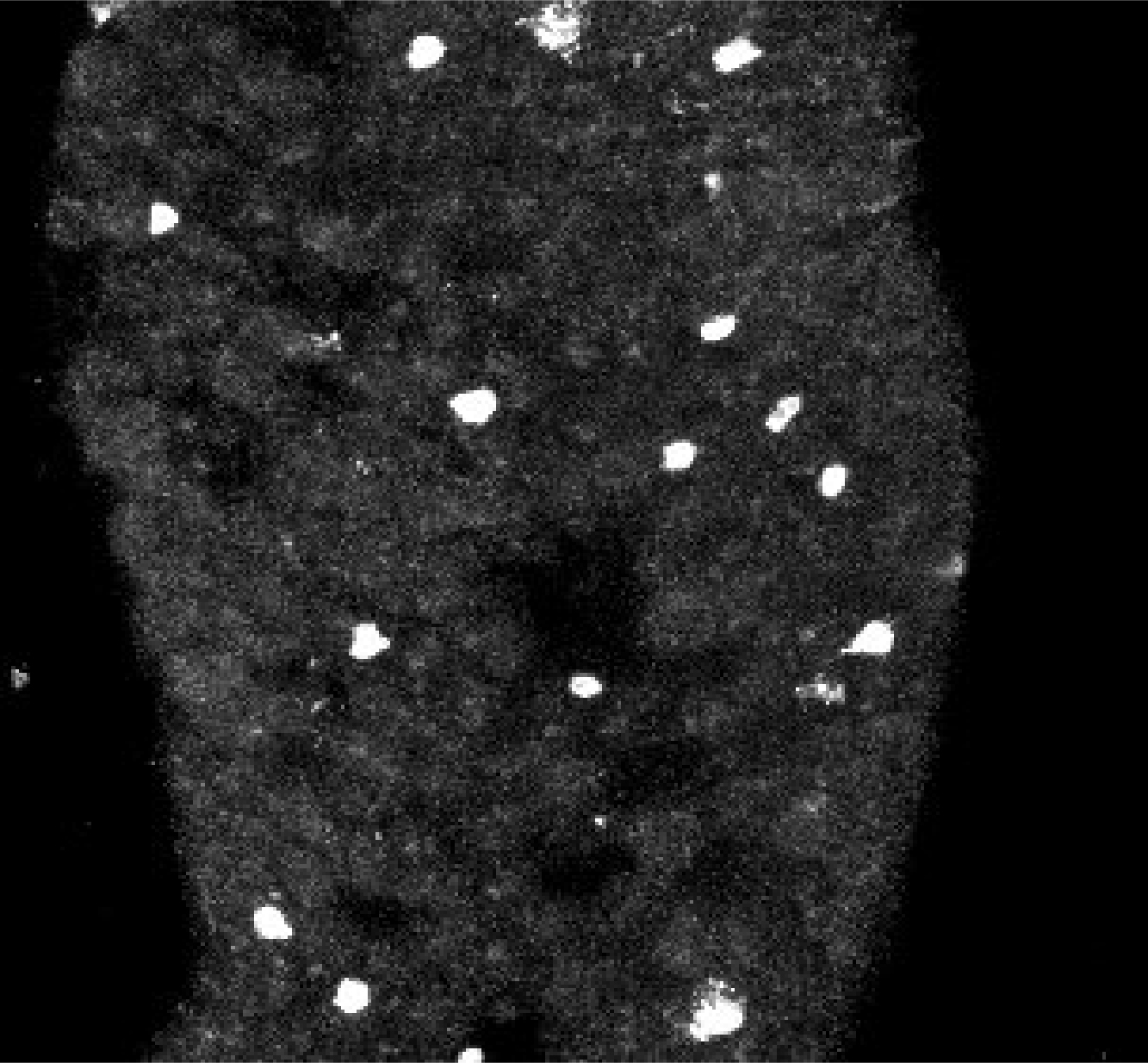

Supplement: Supplementary file 4 — Source data Fig. 3 [file 44318_2026_808_MOESM4_ESM.zip › Fig.3/Panel C/Control - TK.jpg]

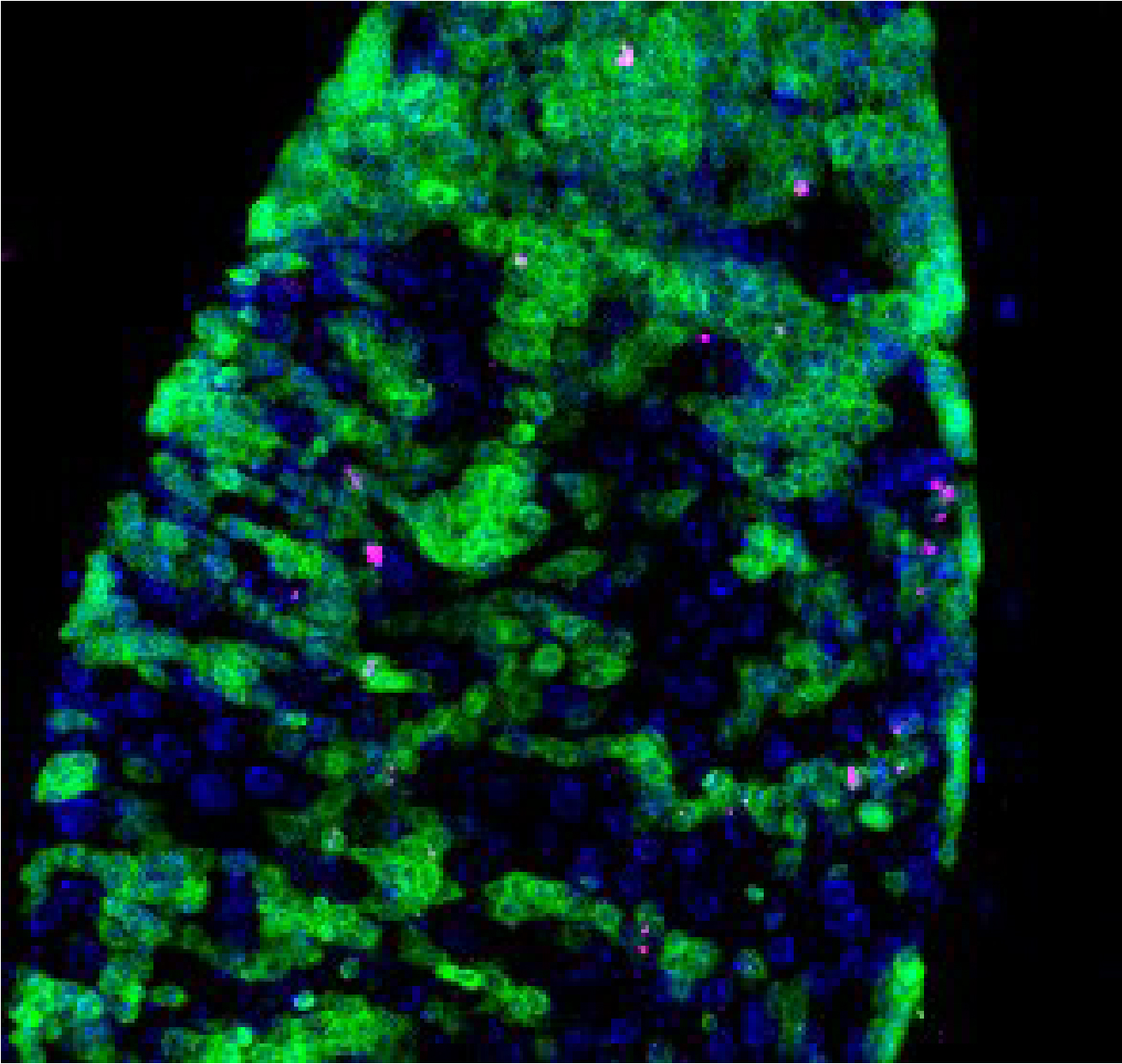

Supplement: Supplementary file 4 — Source data Fig. 3 [file 44318_2026_808_MOESM4_ESM.zip › Fig.3/Panel C/Notch RNAi - GFP PH3.jpg]

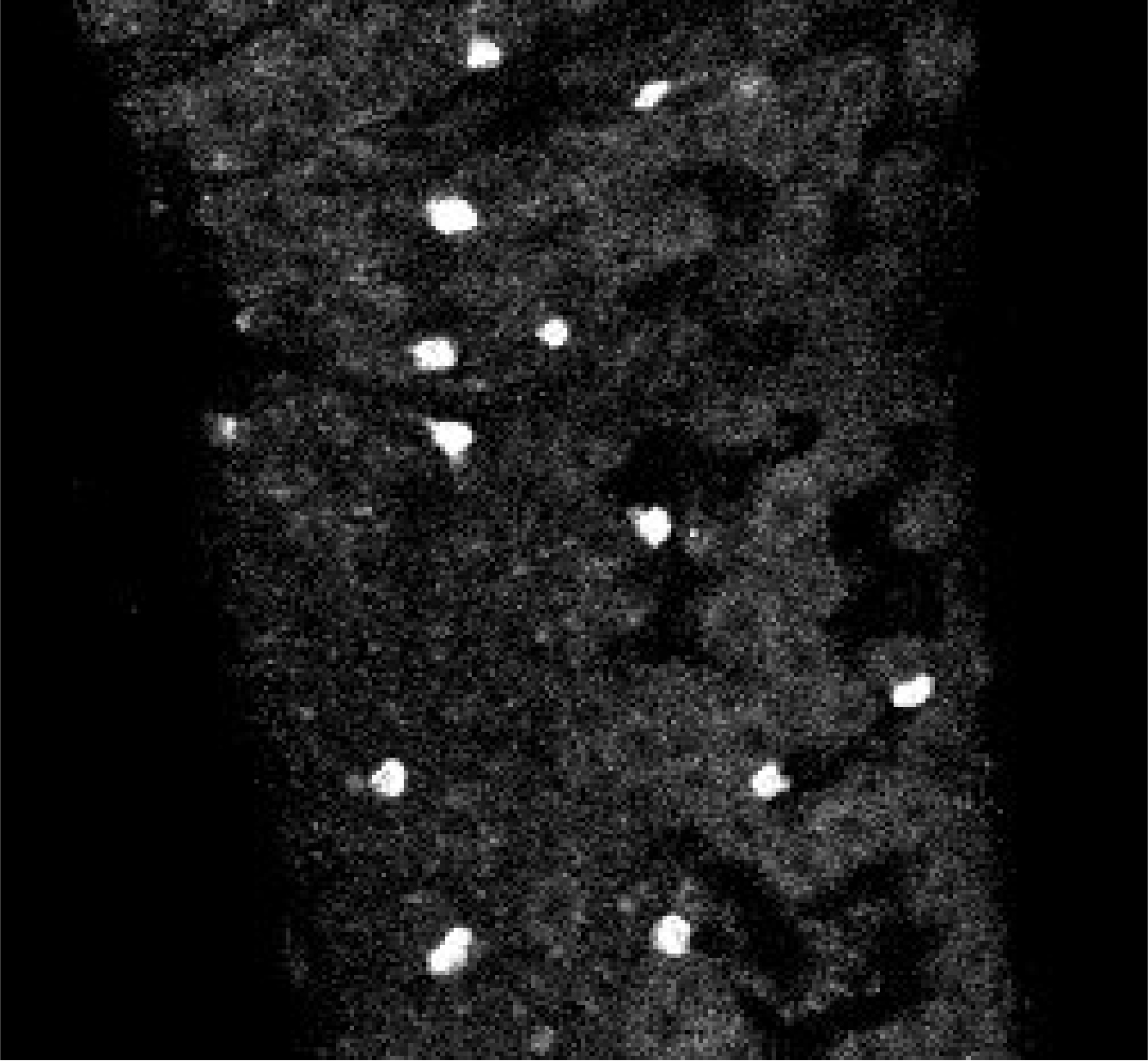

Supplement: Supplementary file 4 — Source data Fig. 3 [file 44318_2026_808_MOESM4_ESM.zip › Fig.3/Panel C/Cph RNAi#1 + Notch RNAi - AstC.jpg]

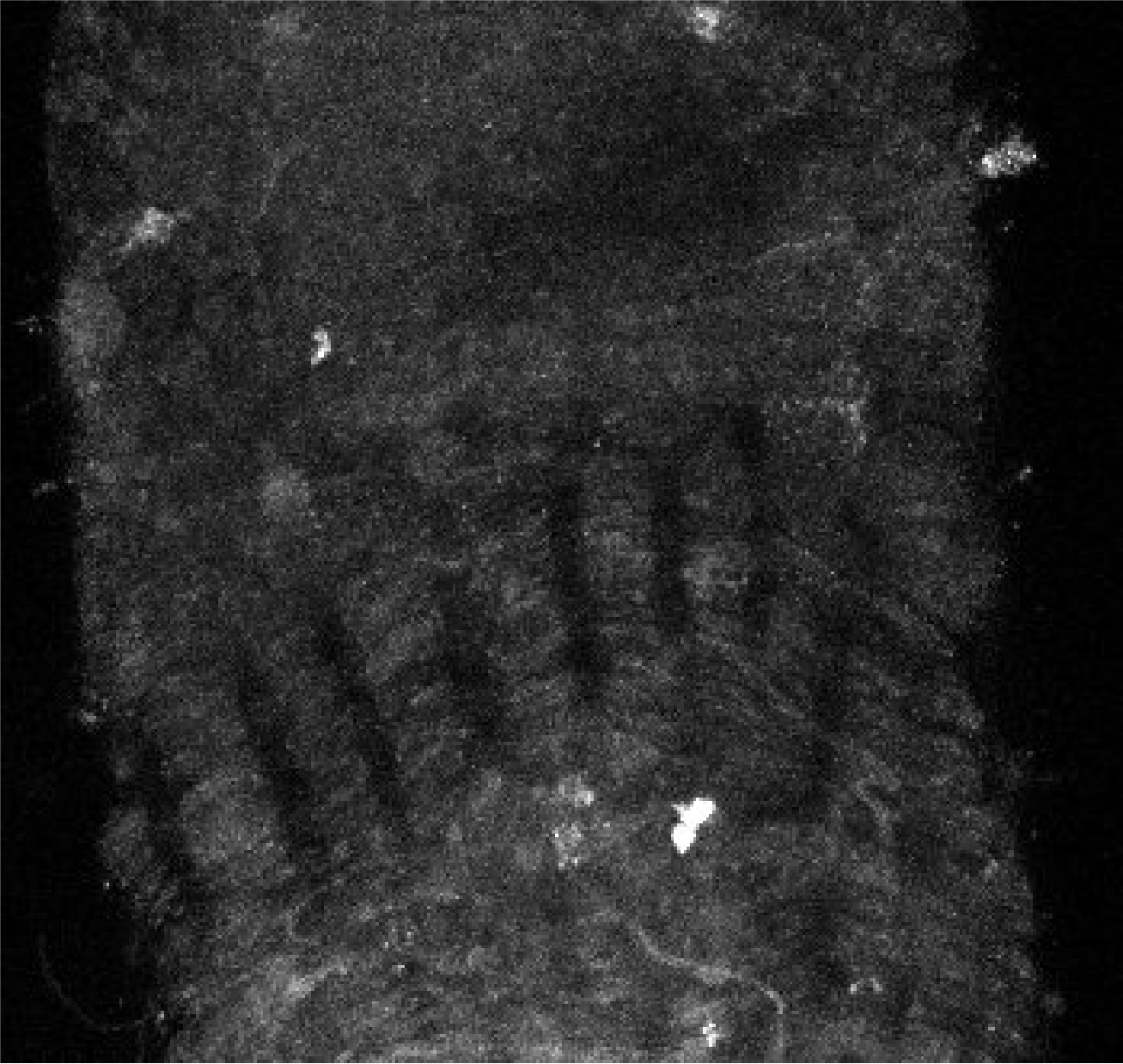

Supplement: Supplementary file 4 — Source data Fig. 3 [file 44318_2026_808_MOESM4_ESM.zip › Fig.3/Panel C/Notch RNAi - TK.jpg]

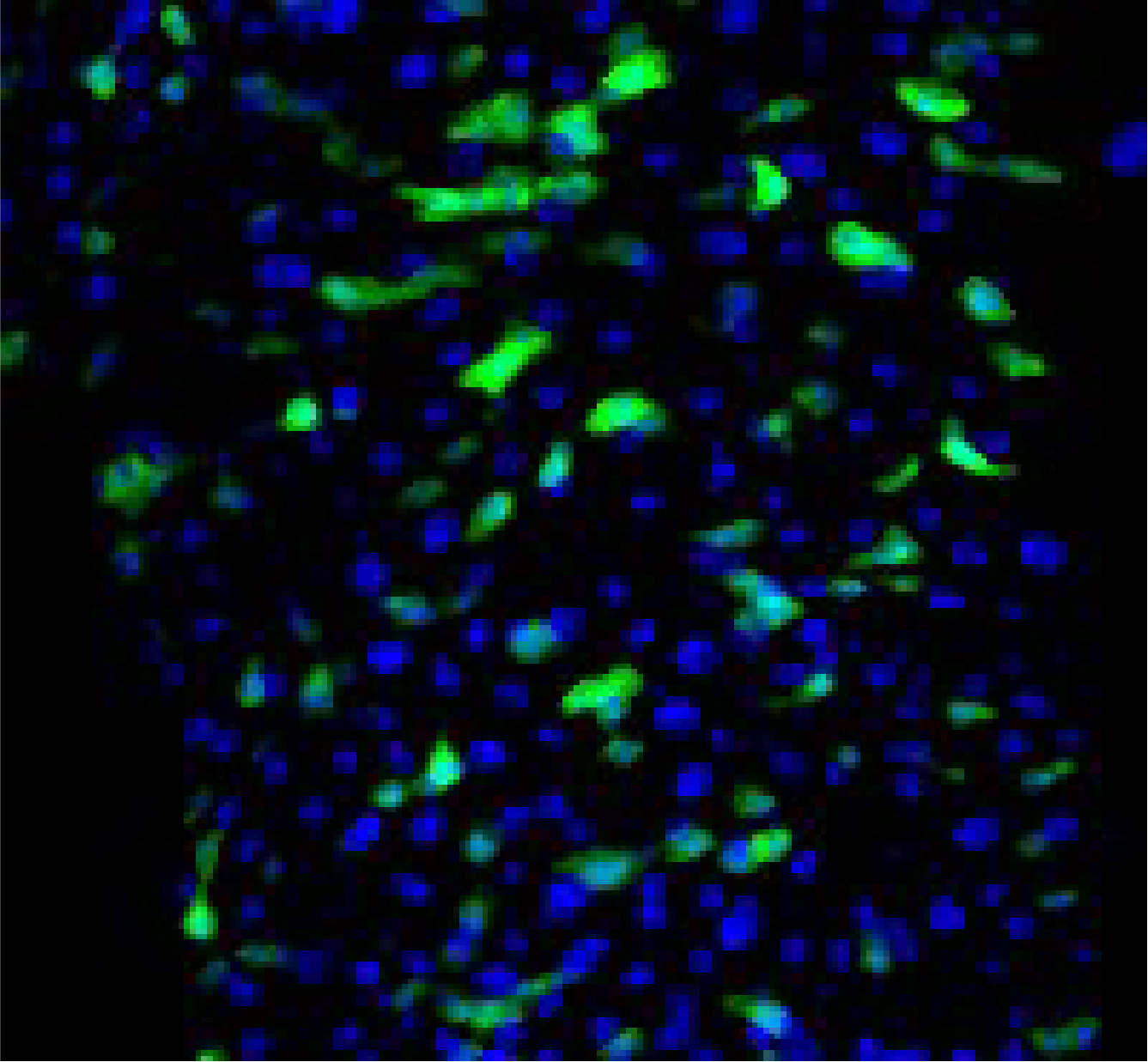

Supplement: Supplementary file 4 — Source data Fig. 3 [file 44318_2026_808_MOESM4_ESM.zip › Fig.3/Panel C/Control - GFP PH3.jpg]

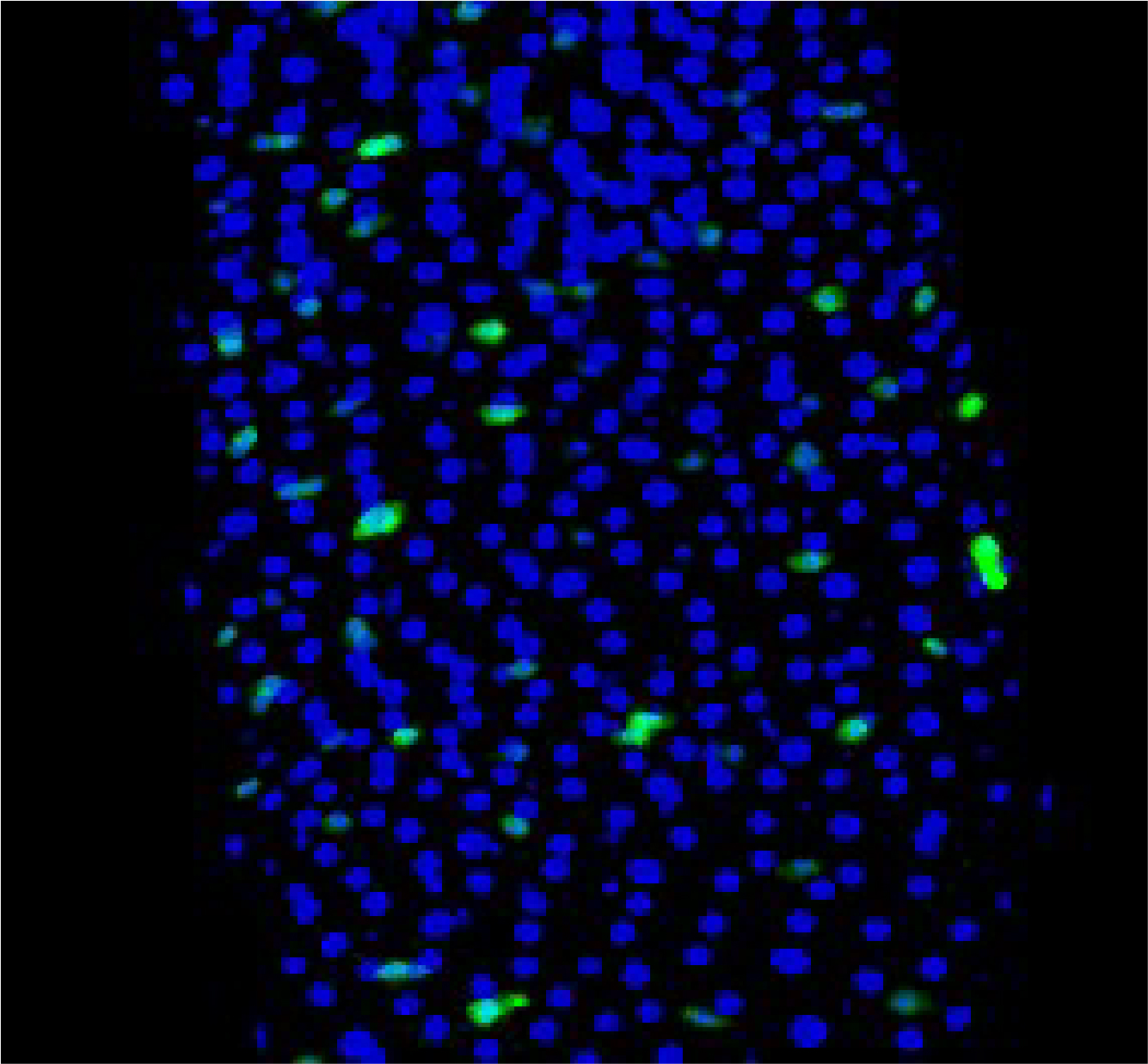

Supplement: Supplementary file 4 — Source data Fig. 3 [file 44318_2026_808_MOESM4_ESM.zip › Fig.3/Panel C/Cph RNAi#1 + Notch RNAi - TK.jpg]

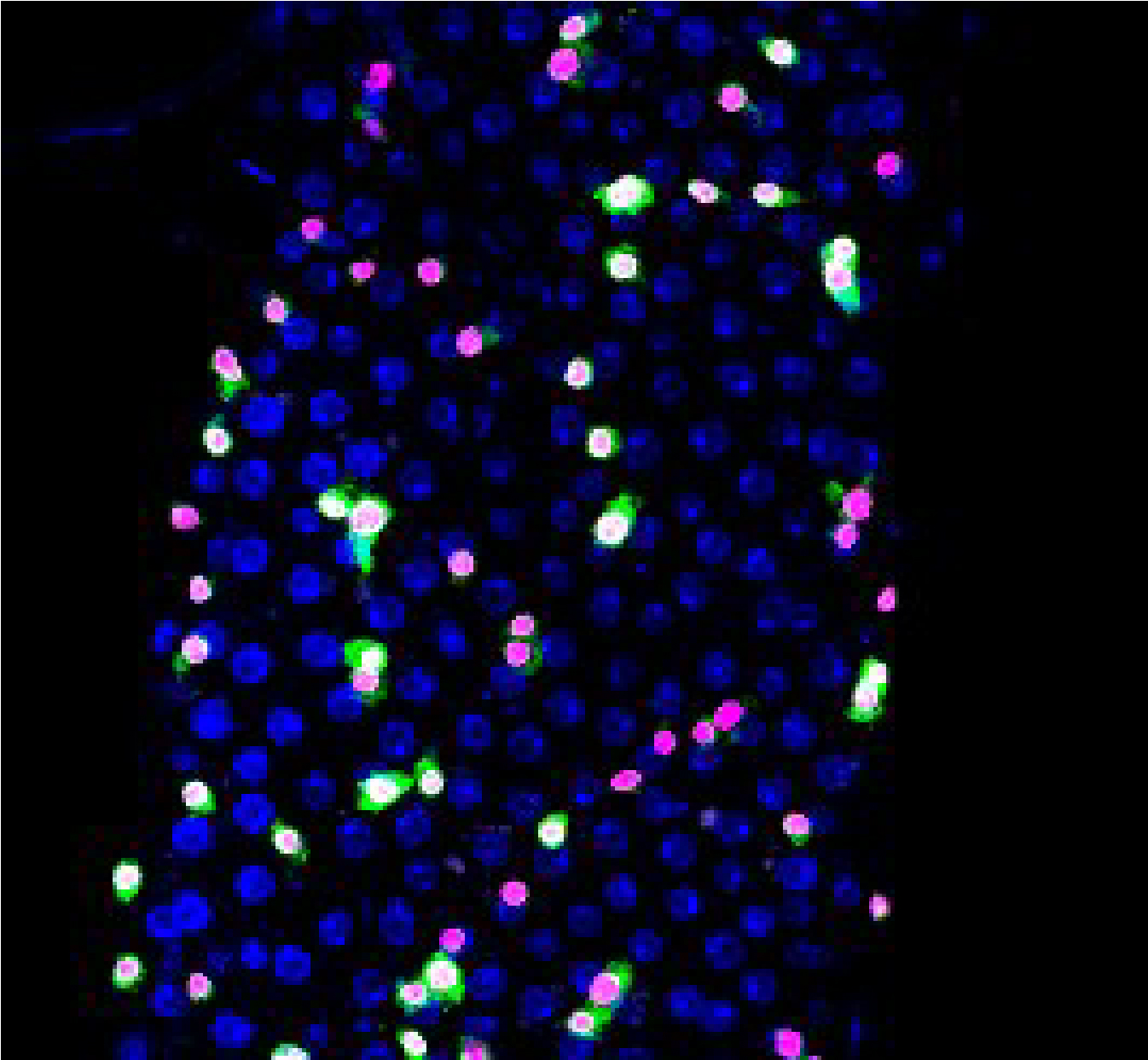

Supplement: Supplementary file 4 — Source data Fig. 3 [file 44318_2026_808_MOESM4_ESM.zip › Fig.3/Panel A/control.jpg]

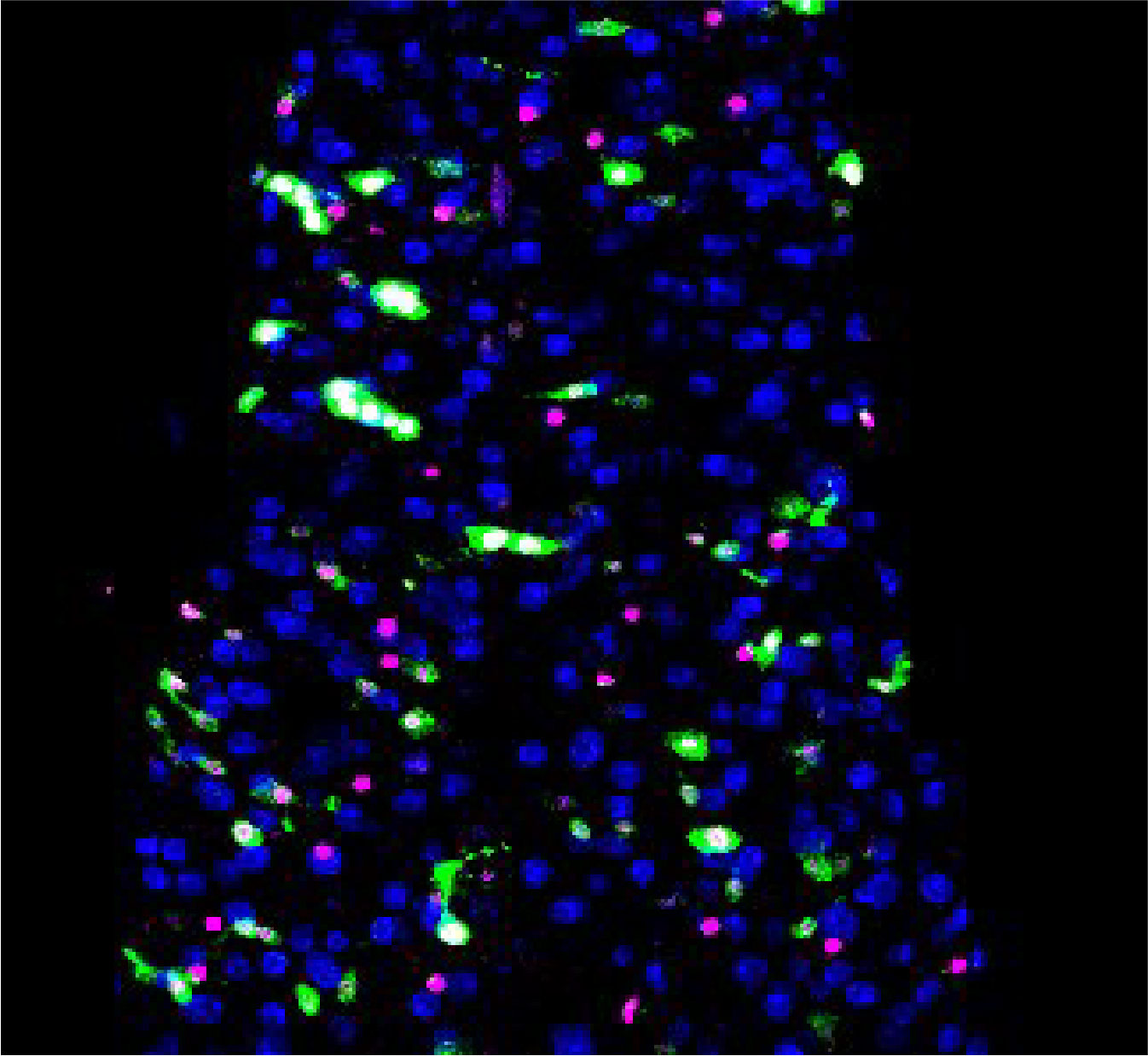

Supplement: Supplementary file 4 — Source data Fig. 3 [file 44318_2026_808_MOESM4_ESM.zip › Fig.3/Panel A/Cph RNAi#1 + Notch RNAi.jpg]

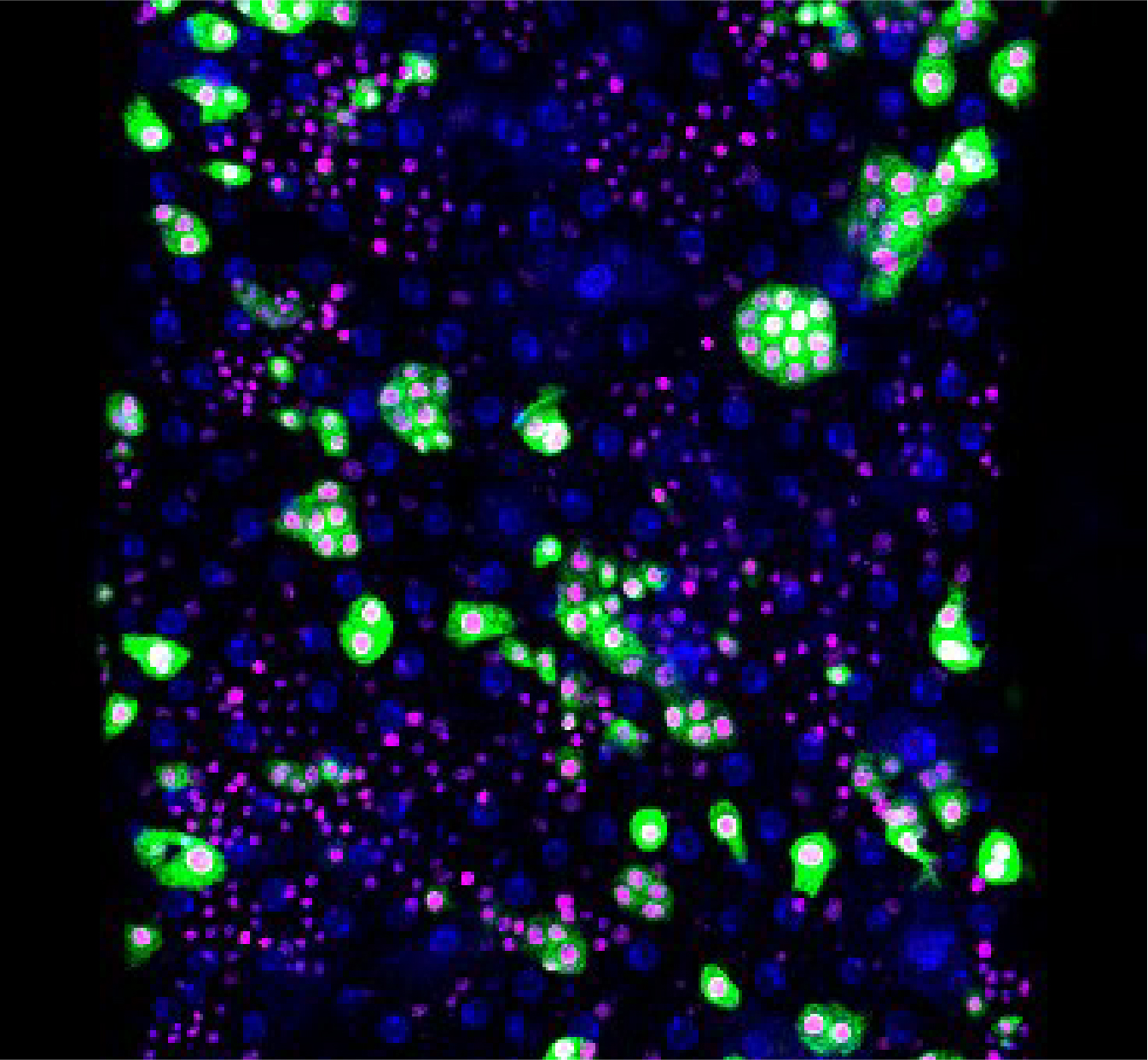

Supplement: Supplementary file 4 — Source data Fig. 3 [file 44318_2026_808_MOESM4_ESM.zip › Fig.3/Panel A/Notch RNAi.jpg]

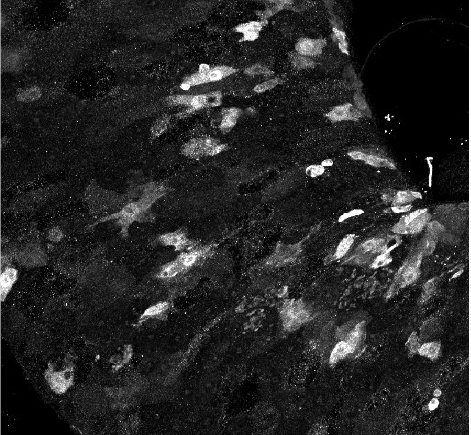

Supplement: Supplementary file 5 — Source data Fig. 4 [file 44318_2026_808_MOESM5_ESM.zip › Fig.4/Panel D/control - LacZ.jpg]

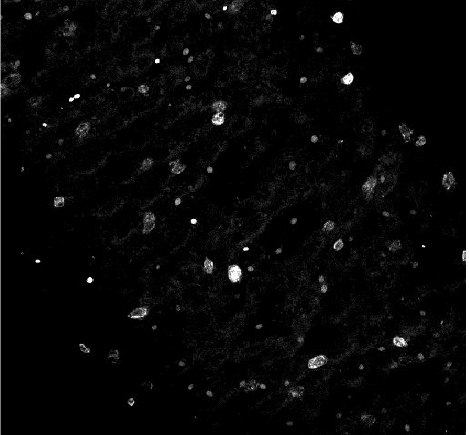

Supplement: Supplementary file 5 — Source data Fig. 4 [file 44318_2026_808_MOESM5_ESM.zip › Fig.4/Panel D/Cph RNAi #2 - GFP.jpg]

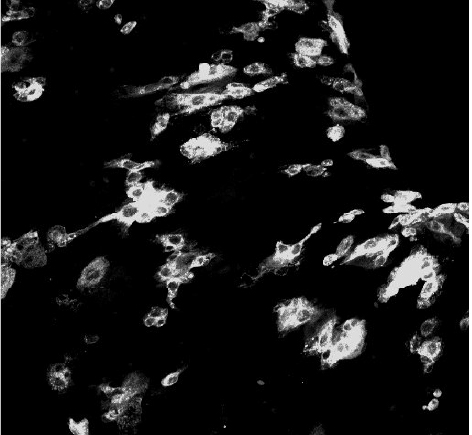

Supplement: Supplementary file 5 — Source data Fig. 4 [file 44318_2026_808_MOESM5_ESM.zip › Fig.4/Panel D/control - GFP.jpg]

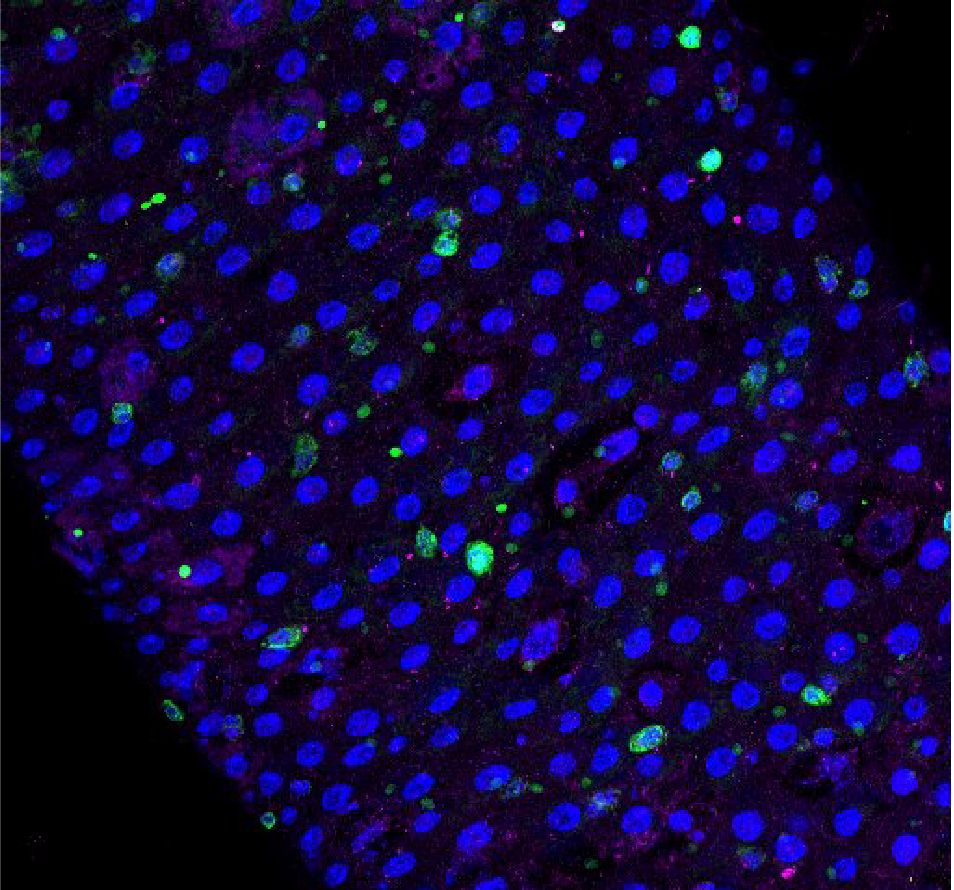

Supplement: Supplementary file 5 — Source data Fig. 4 [file 44318_2026_808_MOESM5_ESM.zip › Fig.4/Panel D/Cph RNAi #2 - merged.jpg]

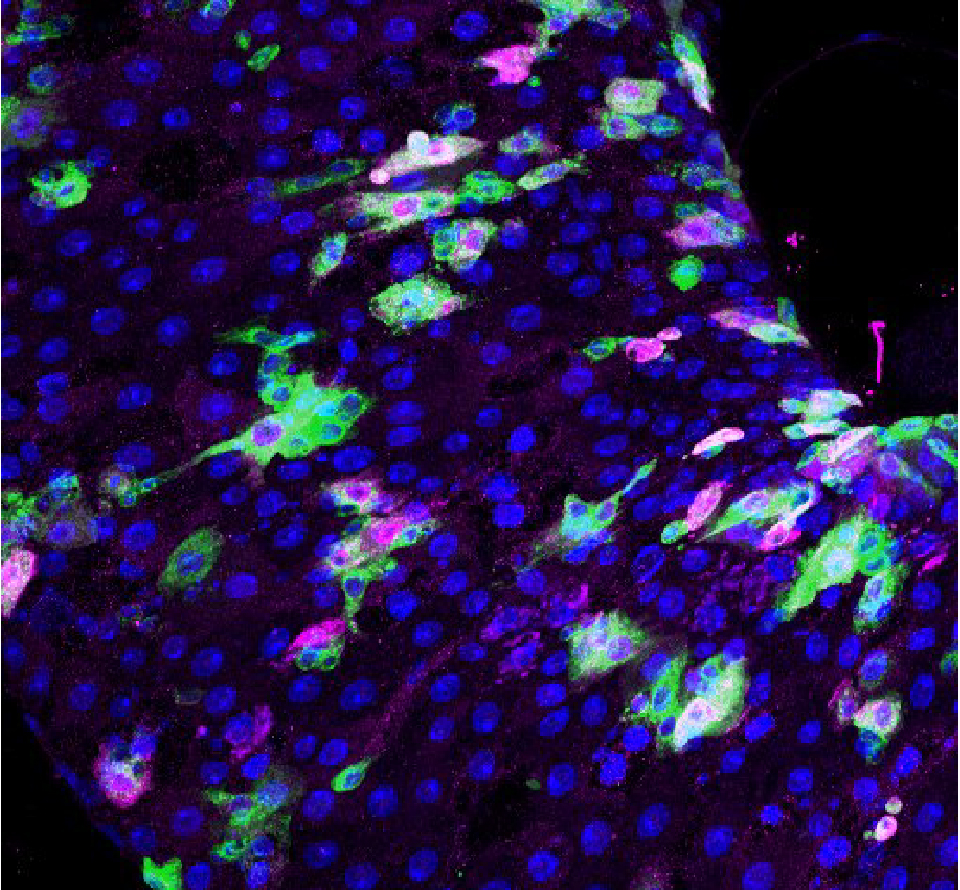

Supplement: Supplementary file 5 — Source data Fig. 4 [file 44318_2026_808_MOESM5_ESM.zip › Fig.4/Panel D/control - merged.jpg]

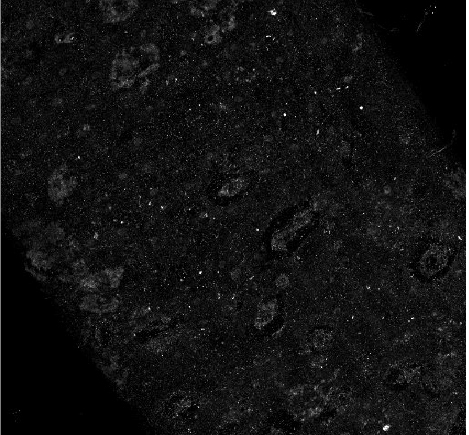

Supplement: Supplementary file 5 — Source data Fig. 4 [file 44318_2026_808_MOESM5_ESM.zip › Fig.4/Panel D/Cph RNAi #2 - LacZ.jpg]

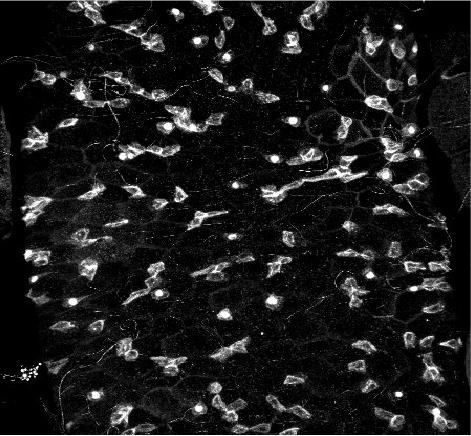

Supplement: Supplementary file 5 — Source data Fig. 4 [file 44318_2026_808_MOESM5_ESM.zip › Fig.4/Panel F/control - Arm:Pros.jpg]

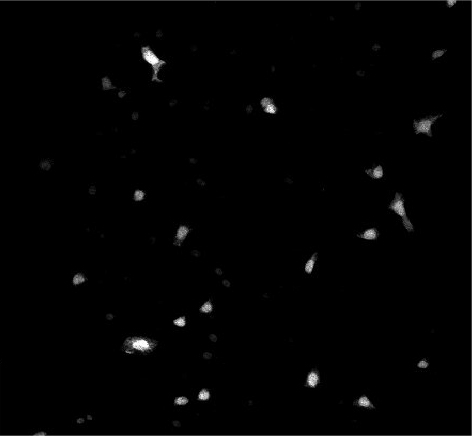

Supplement: Supplementary file 5 — Source data Fig. 4 [file 44318_2026_808_MOESM5_ESM.zip › Fig.4/Panel F/Cph RNAi#2 - GFP.jpg]

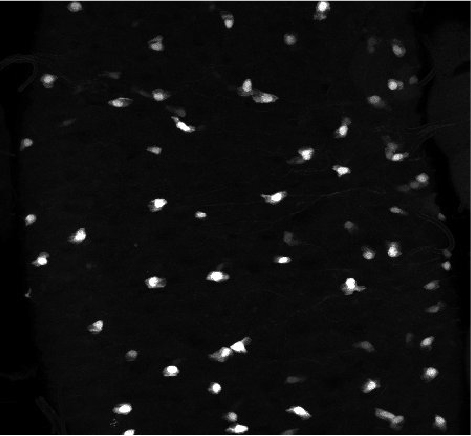

Supplement: Supplementary file 5 — Source data Fig. 4 [file 44318_2026_808_MOESM5_ESM.zip › Fig.4/Panel F/control - GFP.jpg]

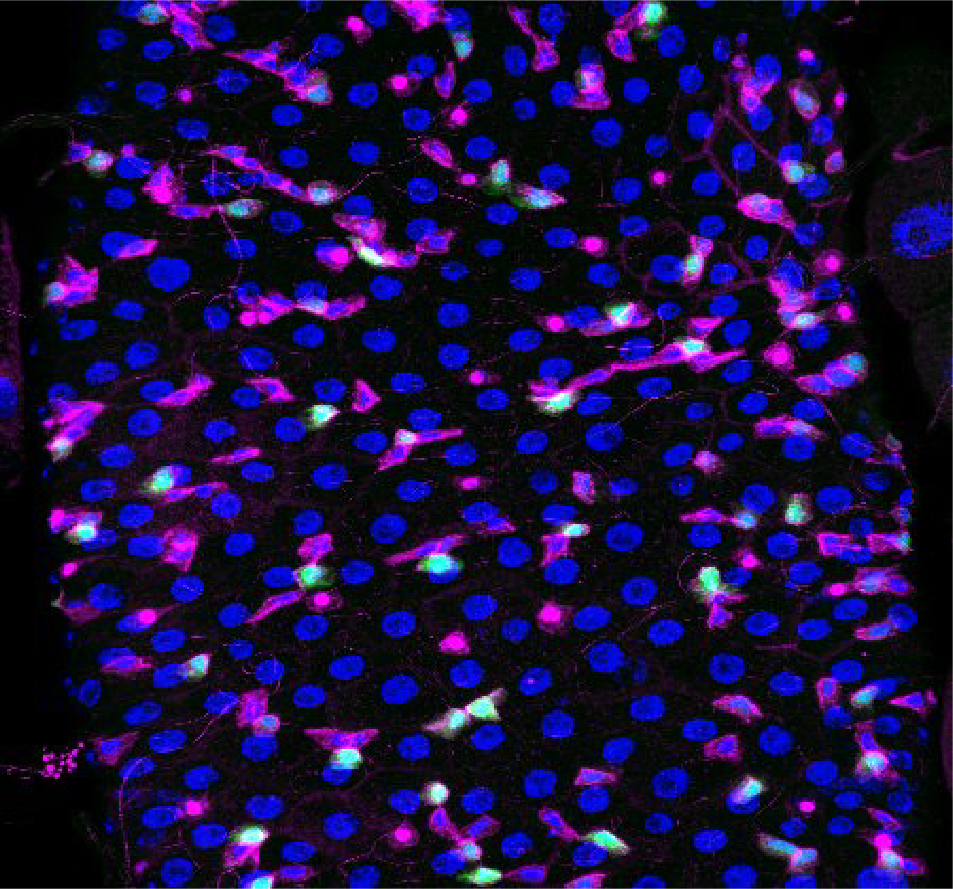

Supplement: Supplementary file 5 — Source data Fig. 4 [file 44318_2026_808_MOESM5_ESM.zip › Fig.4/Panel F/control - merged.jpg]

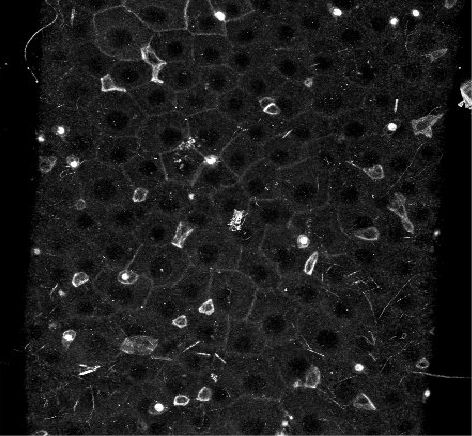

Supplement: Supplementary file 5 — Source data Fig. 4 [file 44318_2026_808_MOESM5_ESM.zip › Fig.4/Panel F/Cph RNAi#2 - Arm:Pros.jpg]

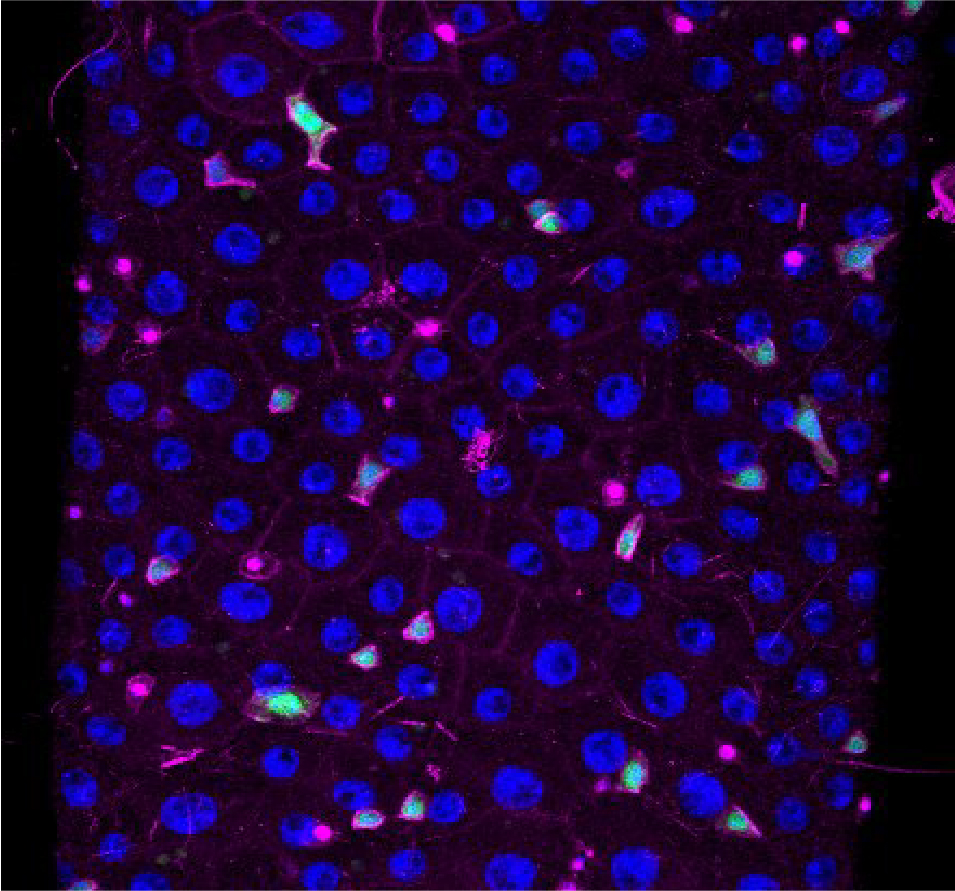

Supplement: Supplementary file 5 — Source data Fig. 4 [file 44318_2026_808_MOESM5_ESM.zip › Fig.4/Panel F/Cph RNAi#2 - merged.jpg]

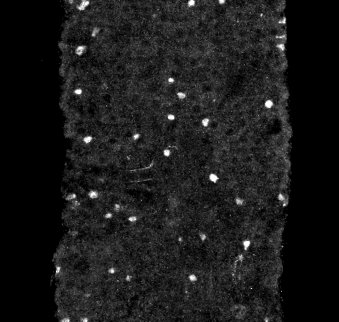

Supplement: Supplementary file 5 — Source data Fig. 4 [file 44318_2026_808_MOESM5_ESM.zip › Fig.4/Panel A/control - AstC.jpg]

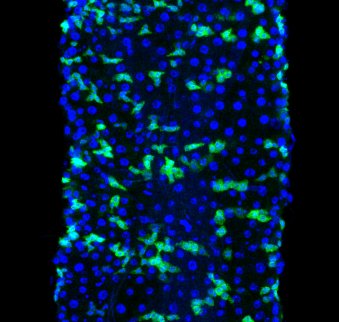

Supplement: Supplementary file 5 — Source data Fig. 4 [file 44318_2026_808_MOESM5_ESM.zip › Fig.4/Panel A/control - GFP.jpg]

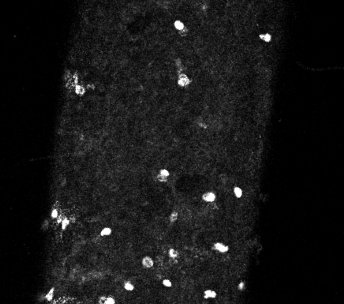

Supplement: Supplementary file 5 — Source data Fig. 4 [file 44318_2026_808_MOESM5_ESM.zip › Fig.4/Panel A/control - TK.jpg]

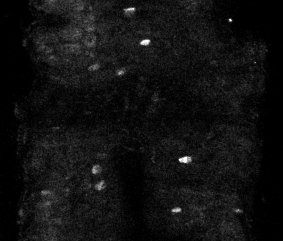

Supplement: Supplementary file 5 — Source data Fig. 4 [file 44318_2026_808_MOESM5_ESM.zip › Fig.4/Panel A/Cph RNAi#1 - AstC.jpg]

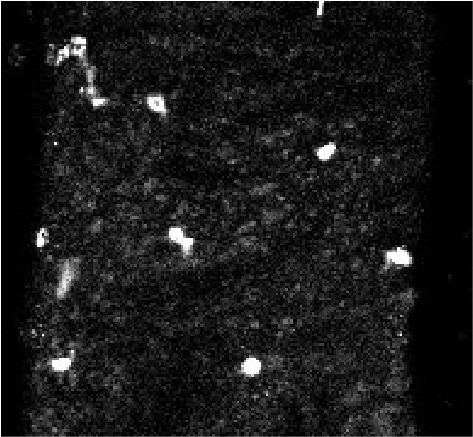

Supplement: Supplementary file 5 — Source data Fig. 4 [file 44318_2026_808_MOESM5_ESM.zip › Fig.4/Panel A/Cph RNAi#1 - TK.jpg]

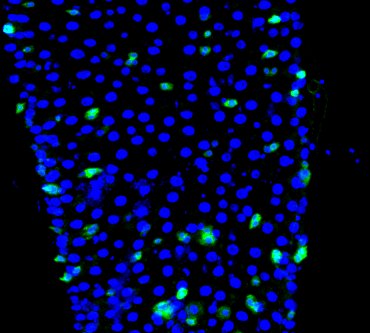

Supplement: Supplementary file 5 — Source data Fig. 4 [file 44318_2026_808_MOESM5_ESM.zip › Fig.4/Panel A/Cph RNAi#1 - GFP.jpg]

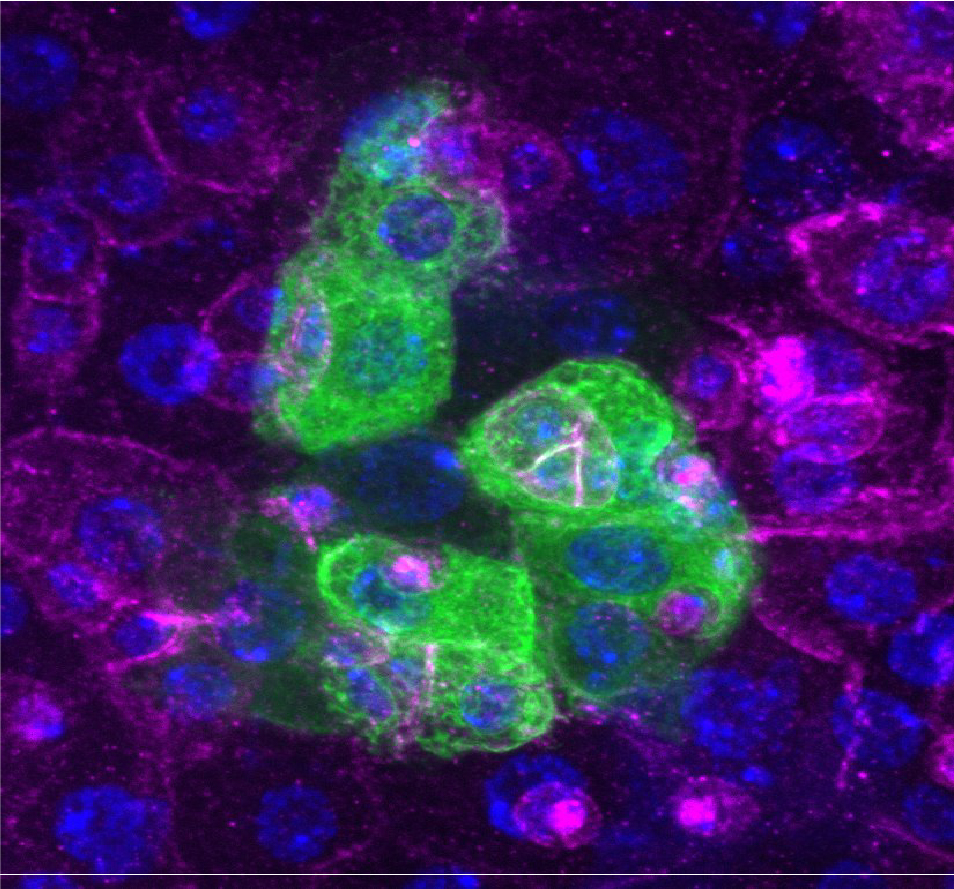

Supplement: Supplementary file 5 — Source data Fig. 4 [file 44318_2026_808_MOESM5_ESM.zip › Fig.4/Panel I/control.jpg]

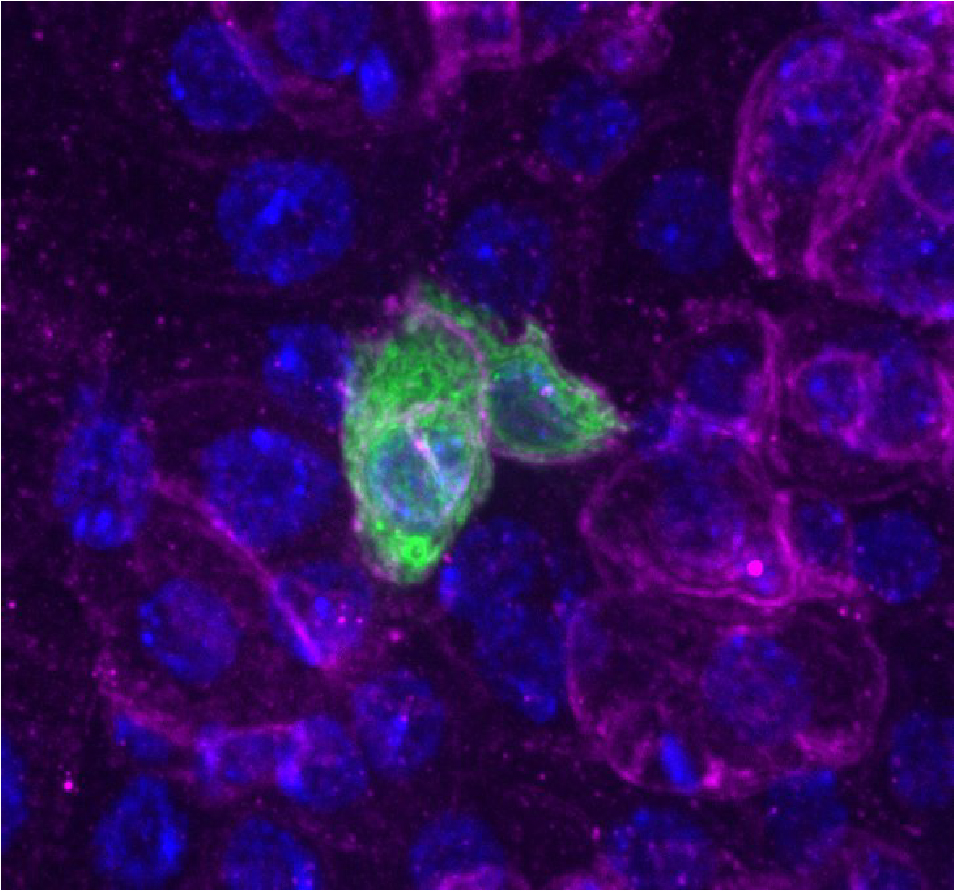

Supplement: Supplementary file 5 — Source data Fig. 4 [file 44318_2026_808_MOESM5_ESM.zip › Fig.4/Panel I/Cph B32.jpg]

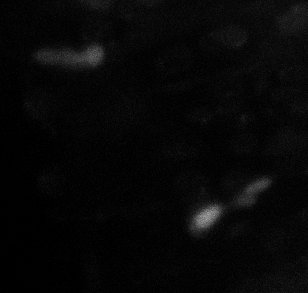

Supplement: Supplementary file 6 — Source data Fig. 5 [file 44318_2026_808_MOESM6_ESM.zip › Fig.5/Panel B/Sc.jpg]

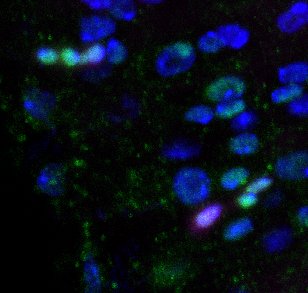

Supplement: Supplementary file 6 — Source data Fig. 5 [file 44318_2026_808_MOESM6_ESM.zip › Fig.5/Panel B/Merged.jpg]

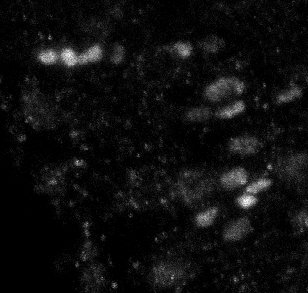

Supplement: Supplementary file 6 — Source data Fig. 5 [file 44318_2026_808_MOESM6_ESM.zip › Fig.5/Panel B/Cph-YFP.jpg]

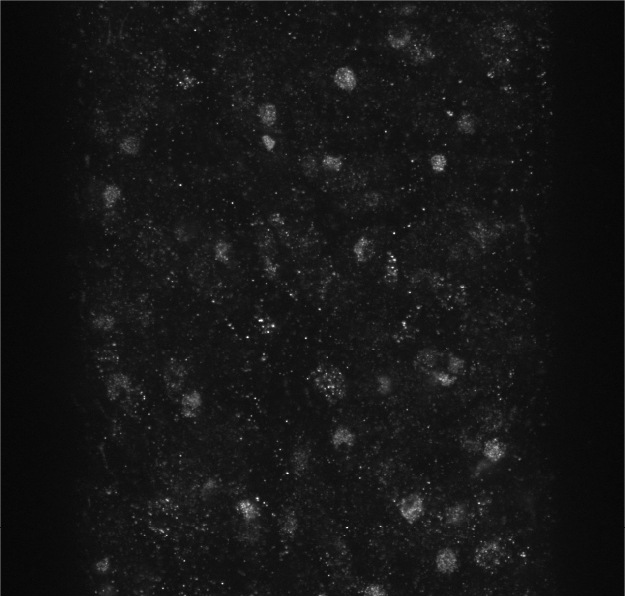

Supplement: Supplementary file 6 — Source data Fig. 5 [file 44318_2026_808_MOESM6_ESM.zip › Fig.5/Panel C/control - Cph YFP.jpg]

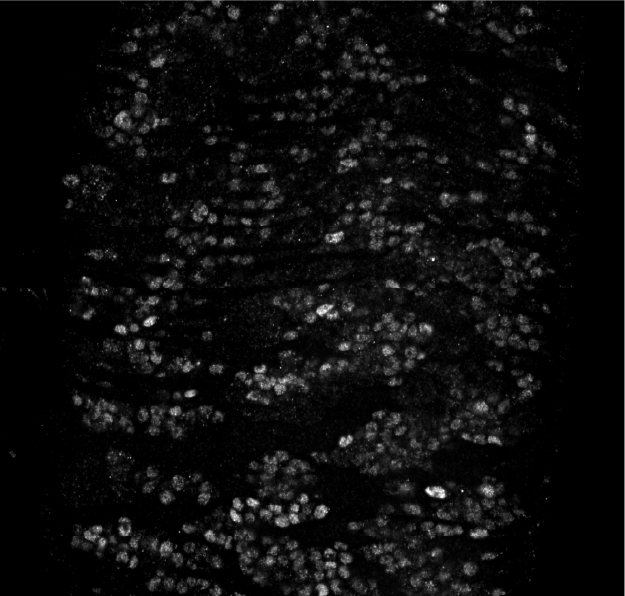

Supplement: Supplementary file 6 — Source data Fig. 5 [file 44318_2026_808_MOESM6_ESM.zip › Fig.5/Panel C/sc - Cph YFP.jpg]

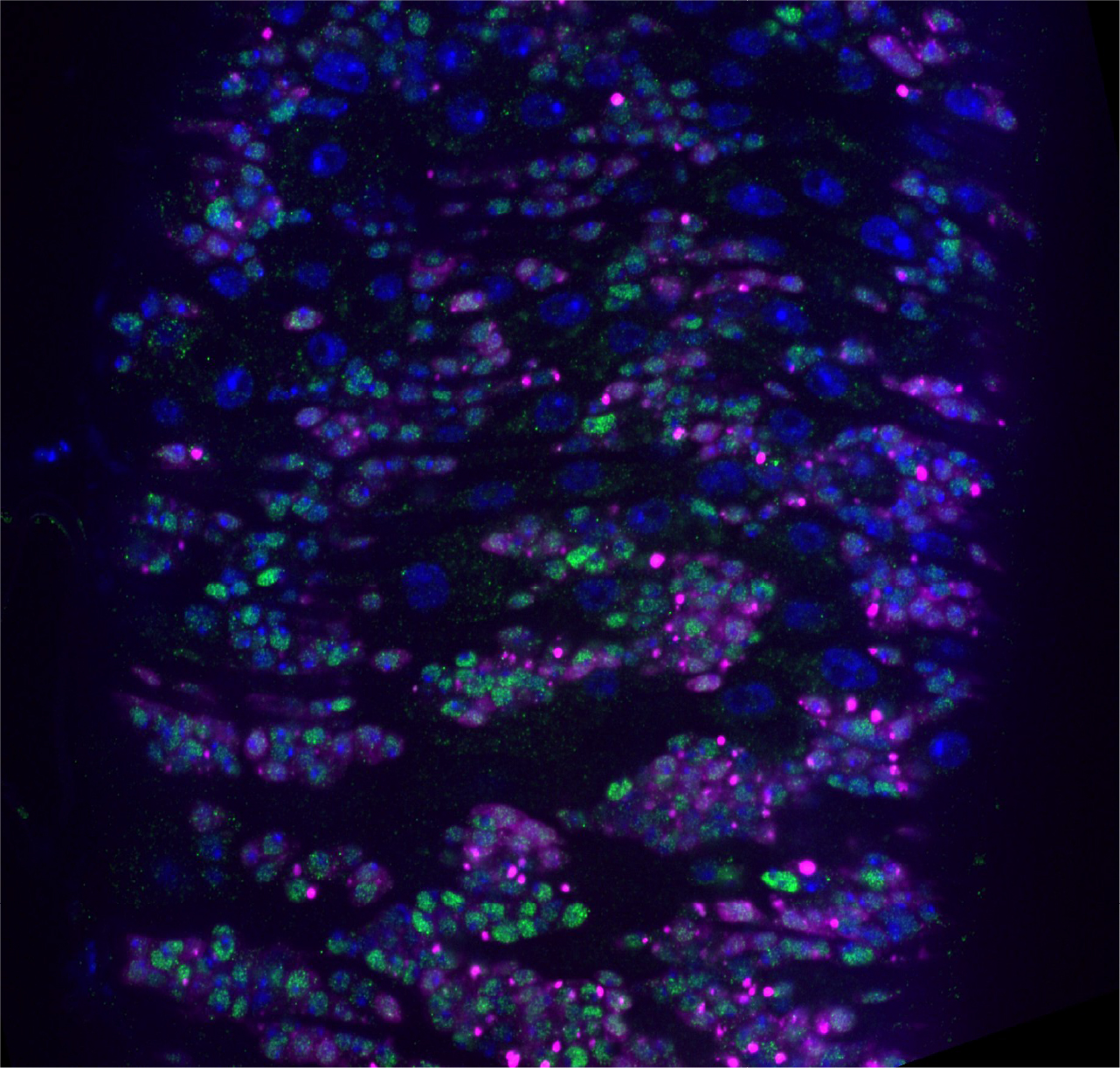

Supplement: Supplementary file 6 — Source data Fig. 5 [file 44318_2026_808_MOESM6_ESM.zip › Fig.5/Panel C/sc - merged.jpg]

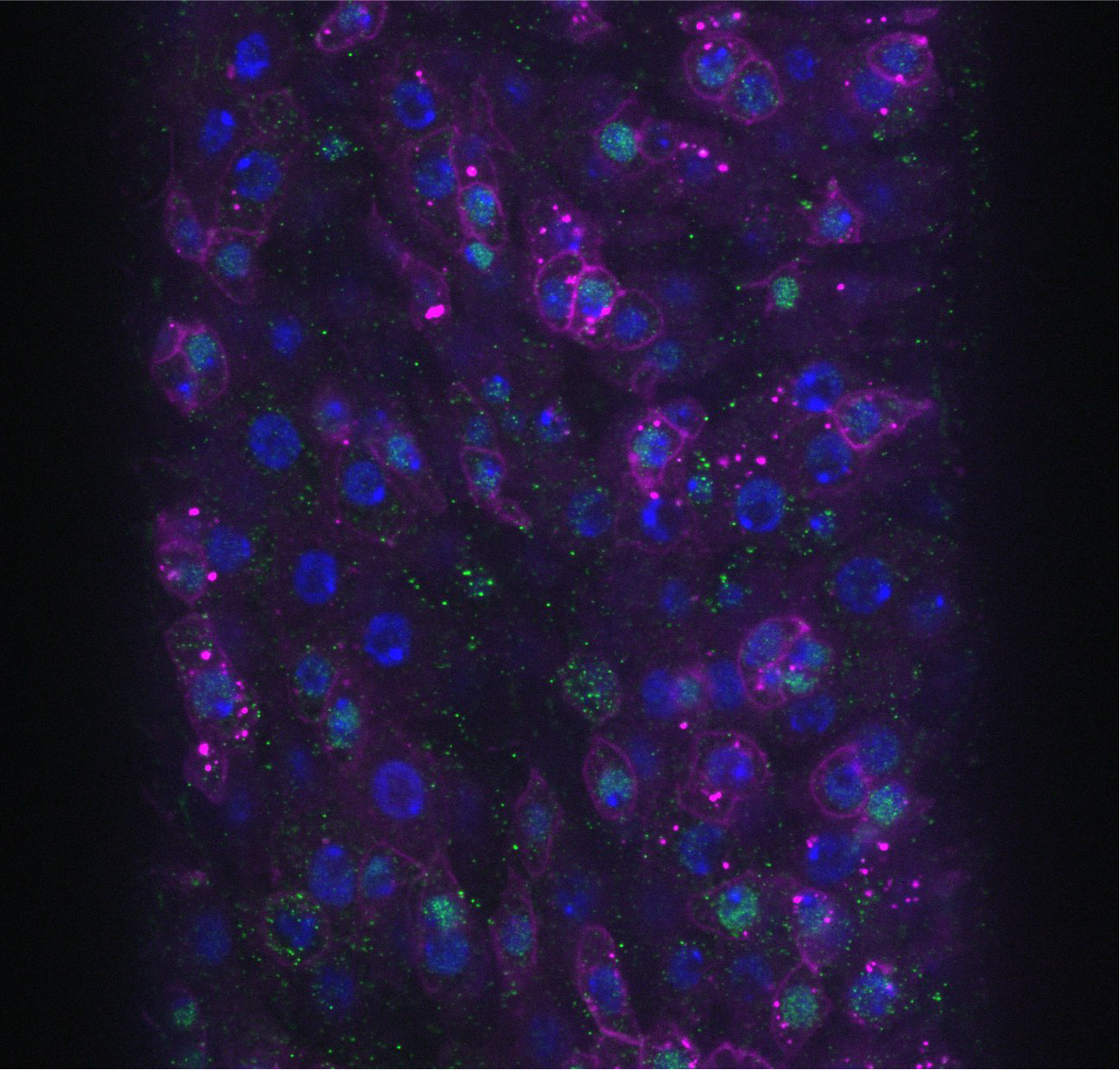

Supplement: Supplementary file 6 — Source data Fig. 5 [file 44318_2026_808_MOESM6_ESM.zip › Fig.5/Panel C/control - merged.jpg]

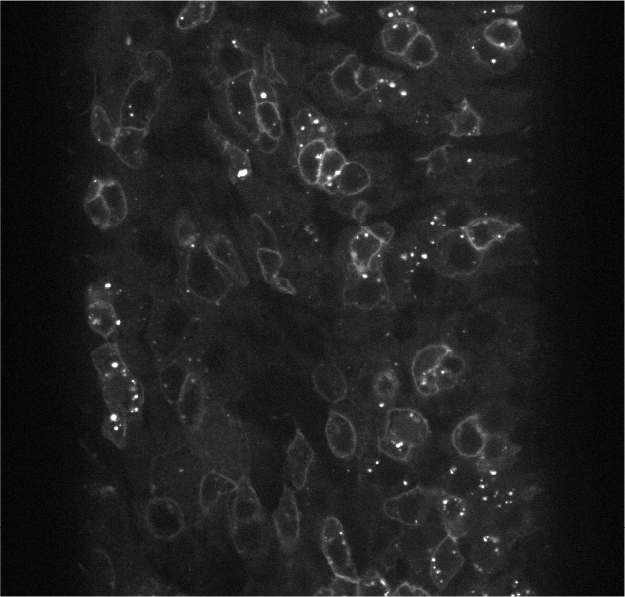

Supplement: Supplementary file 6 — Source data Fig. 5 [file 44318_2026_808_MOESM6_ESM.zip › Fig.5/Panel C/control - RFP.jpg]

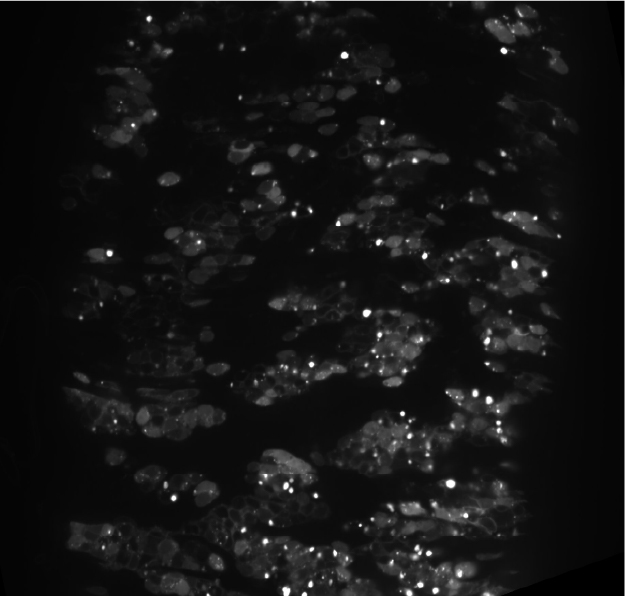

Supplement: Supplementary file 6 — Source data Fig. 5 [file 44318_2026_808_MOESM6_ESM.zip › Fig.5/Panel C/sc - RFP.jpg]

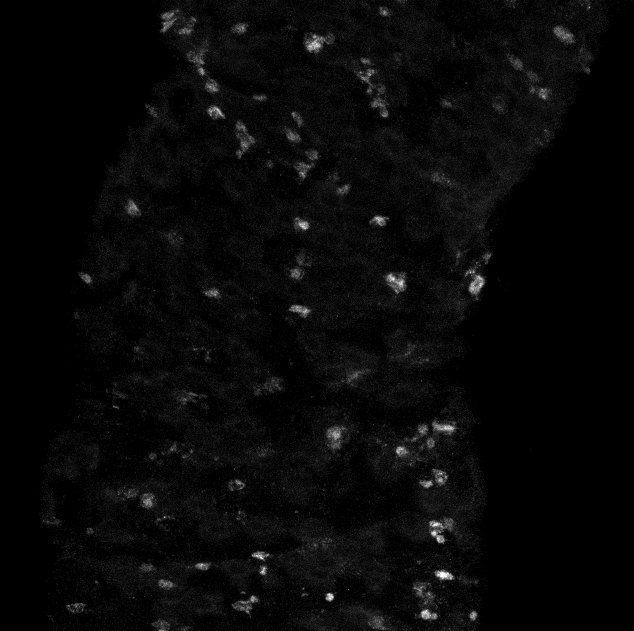

Supplement: Supplementary file 6 — Source data Fig. 5 [file 44318_2026_808_MOESM6_ESM.zip › Fig.5/Panel F/control - AstC.jpg]

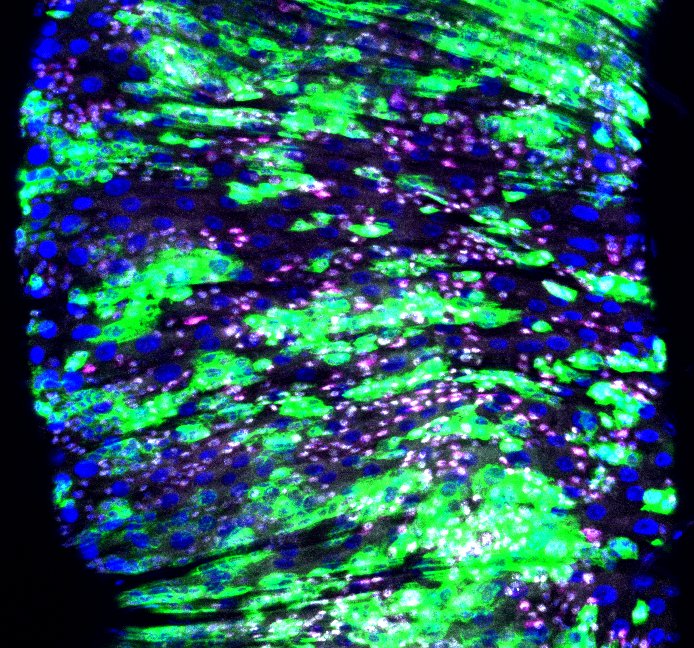

Supplement: Supplementary file 6 — Source data Fig. 5 [file 44318_2026_808_MOESM6_ESM.zip › Fig.5/Panel F/sc - merged.jpg]

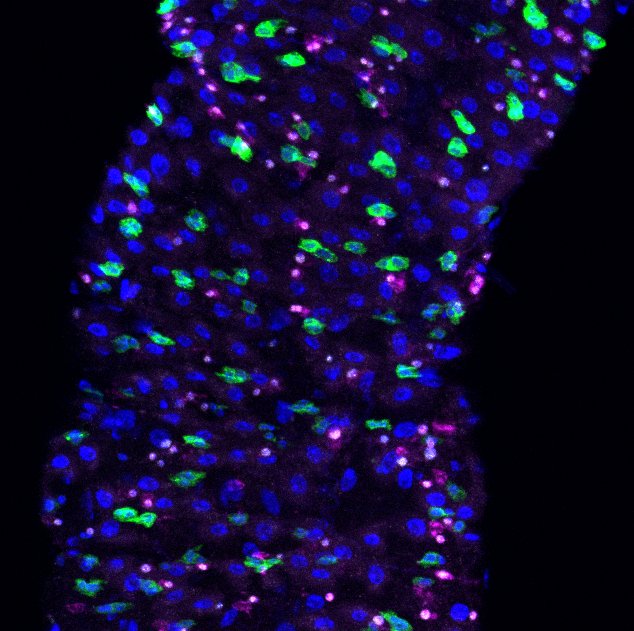

Supplement: Supplementary file 6 — Source data Fig. 5 [file 44318_2026_808_MOESM6_ESM.zip › Fig.5/Panel F/control - merged.jpg]

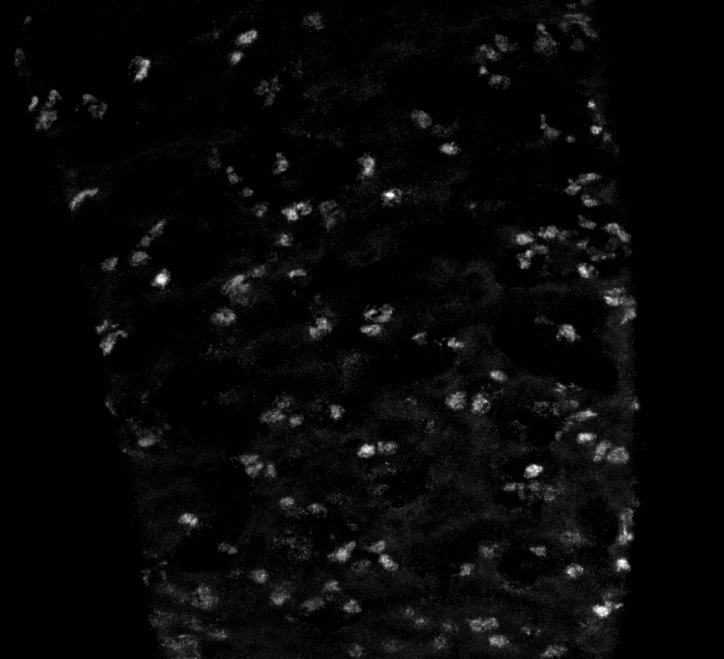

Supplement: Supplementary file 6 — Source data Fig. 5 [file 44318_2026_808_MOESM6_ESM.zip › Fig.5/Panel F/sc + Cph RNAi - AstC1.jpg]

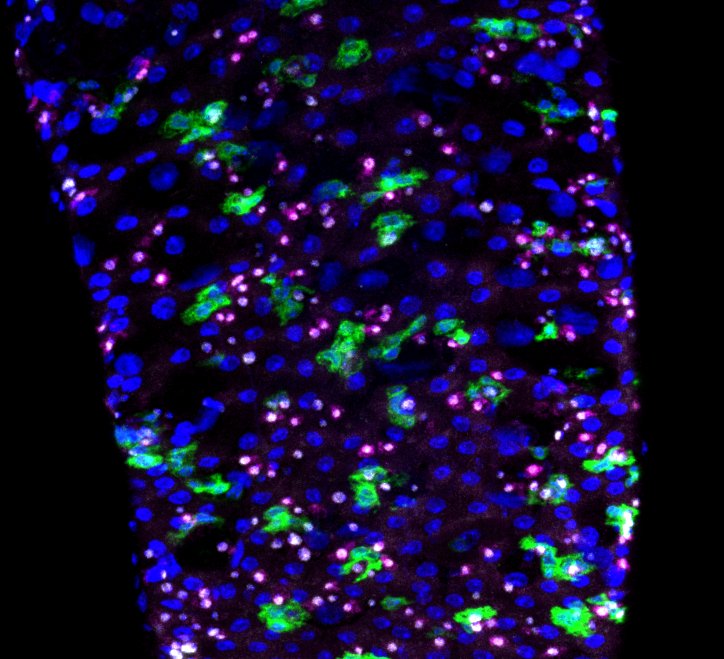

Supplement: Supplementary file 6 — Source data Fig. 5 [file 44318_2026_808_MOESM6_ESM.zip › Fig.5/Panel F/sc + Cph RNAi - merged.jpg]

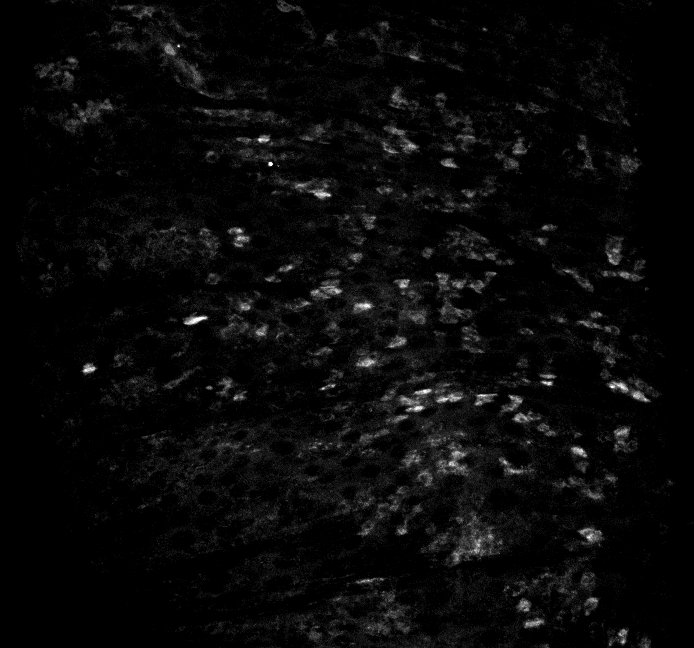

Supplement: Supplementary file 6 — Source data Fig. 5 [file 44318_2026_808_MOESM6_ESM.zip › Fig.5/Panel F/sc - AstC.jpg]

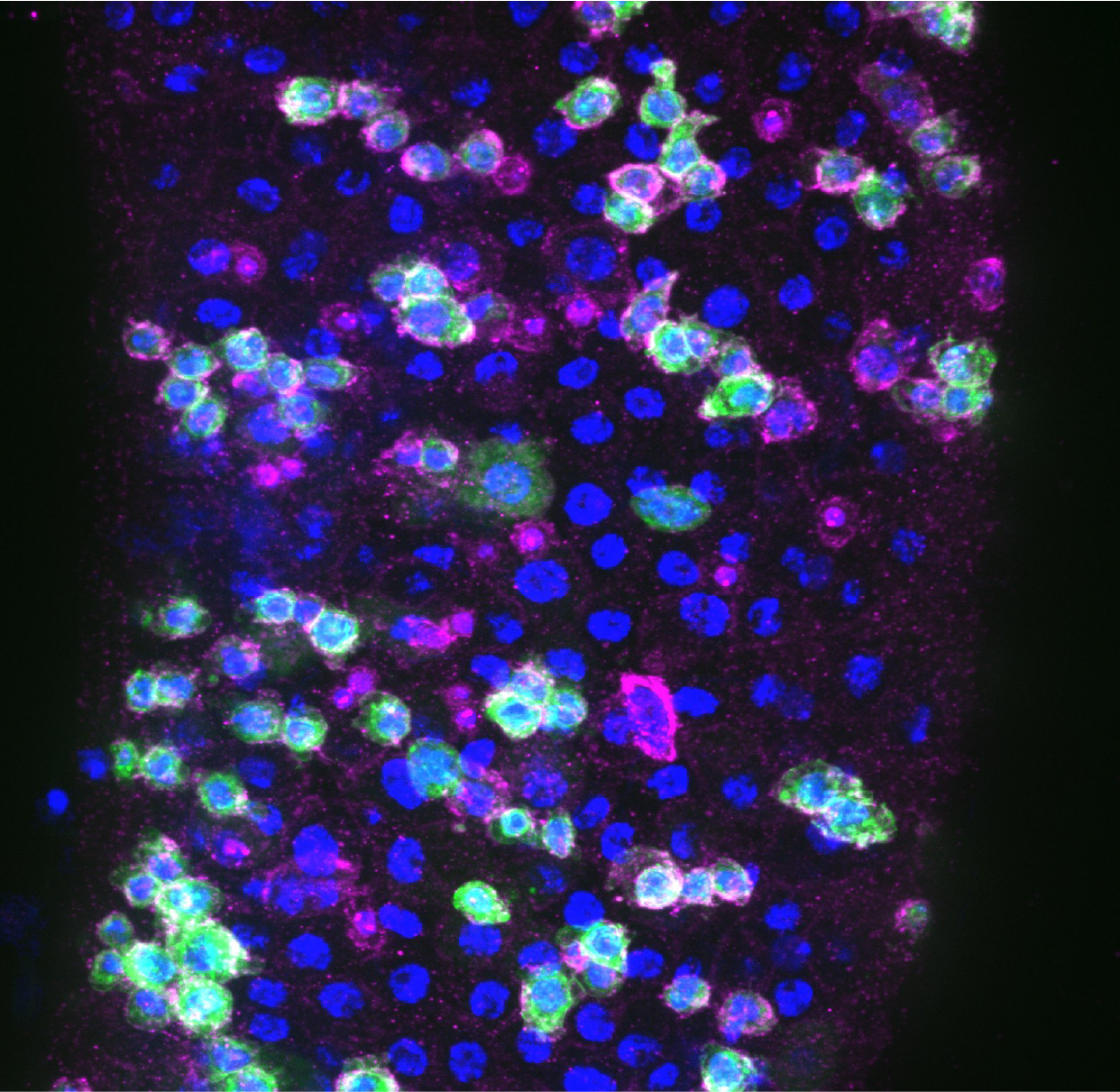

Supplement: Supplementary file 7 — Source data Fig. 7 [file 44318_2026_808_MOESM7_ESM.zip › Fig.7/Panel F/control.jpg]

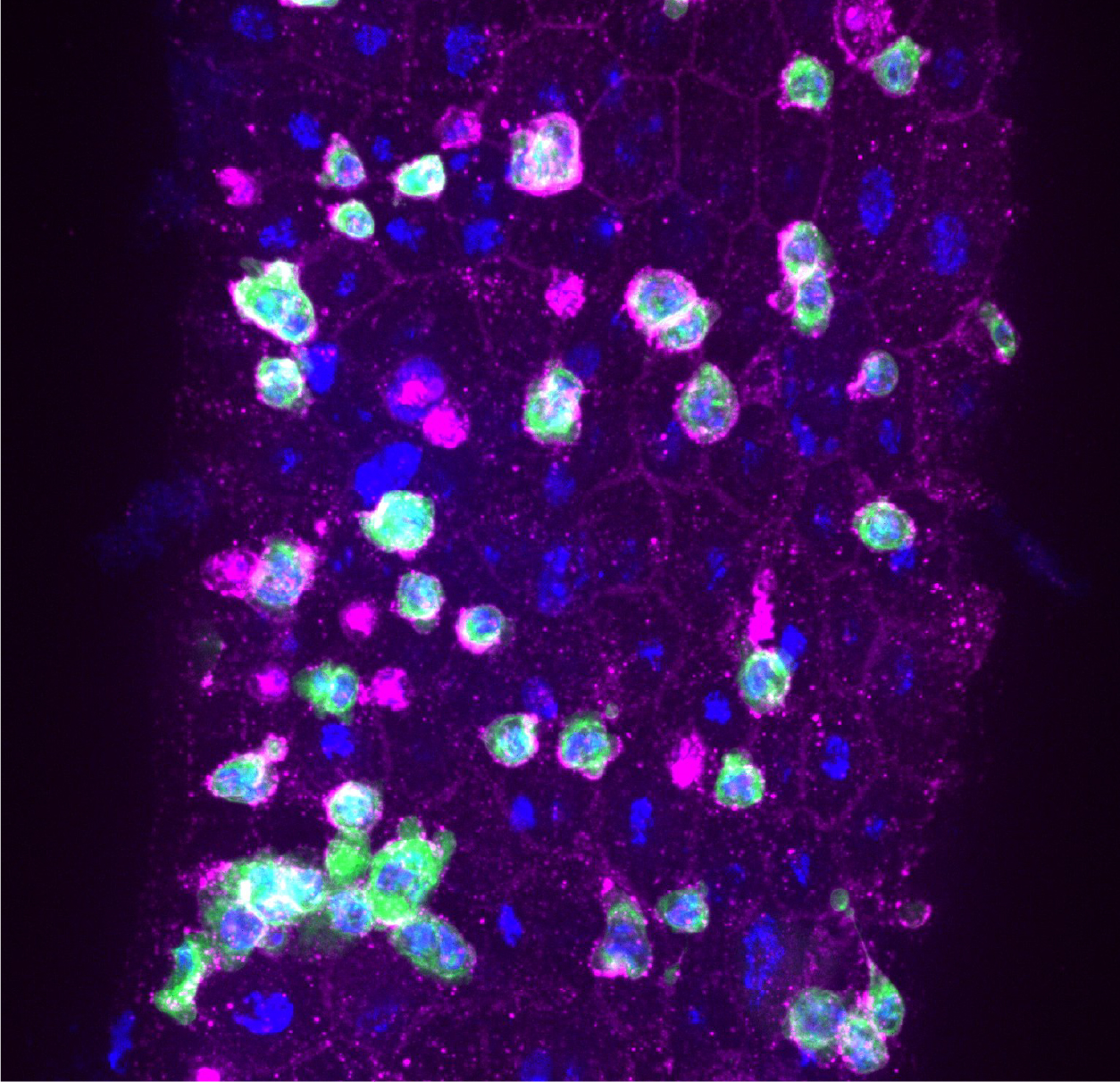

Supplement: Supplementary file 7 — Source data Fig. 7 [file 44318_2026_808_MOESM7_ESM.zip › Fig.7/Panel F/Sff RNAi.jpg]

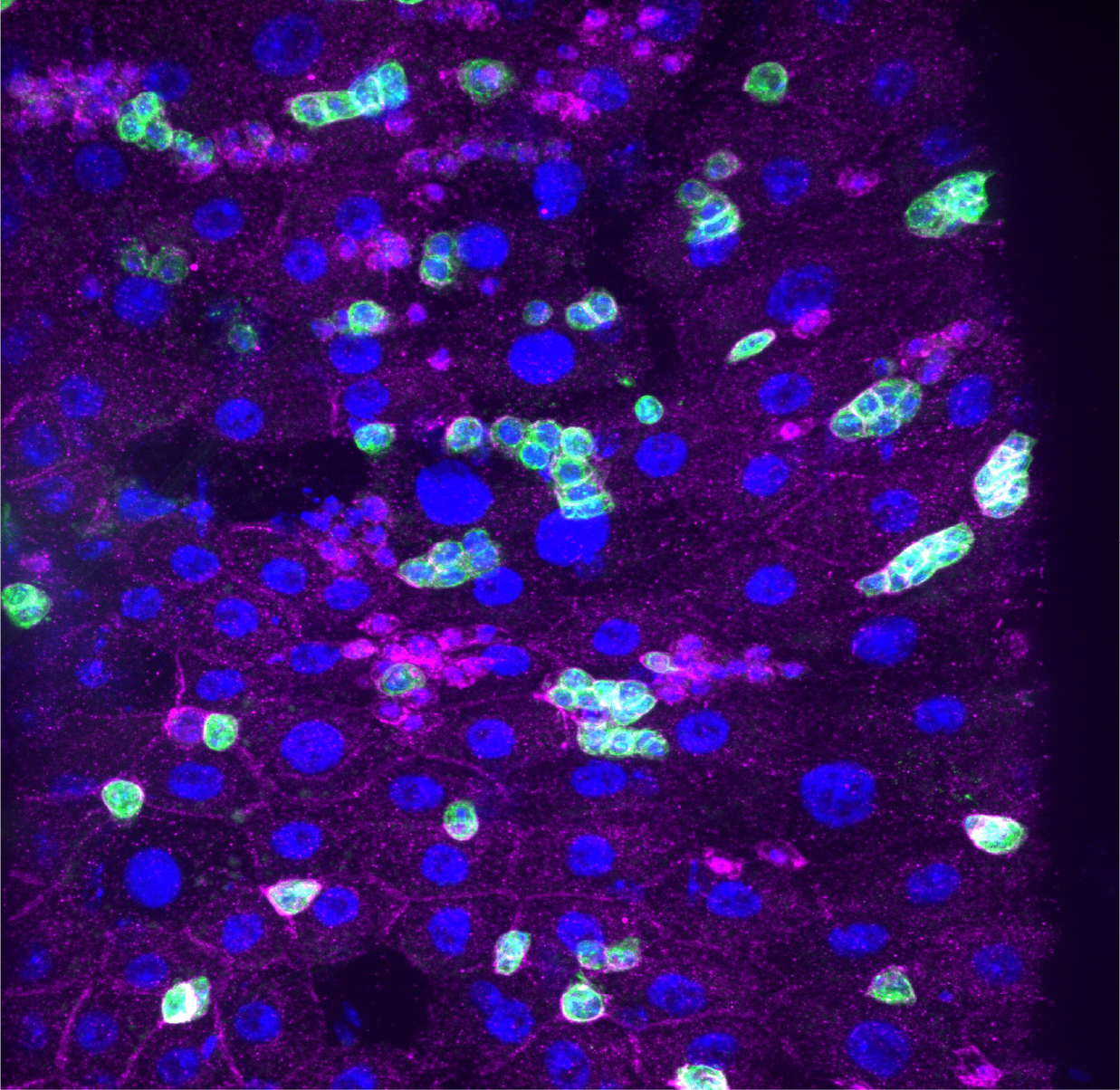

Supplement: Supplementary file 7 — Source data Fig. 7 [file 44318_2026_808_MOESM7_ESM.zip › Fig.7/Panel F/Sff RNAi + Notch RNAi.jpg]

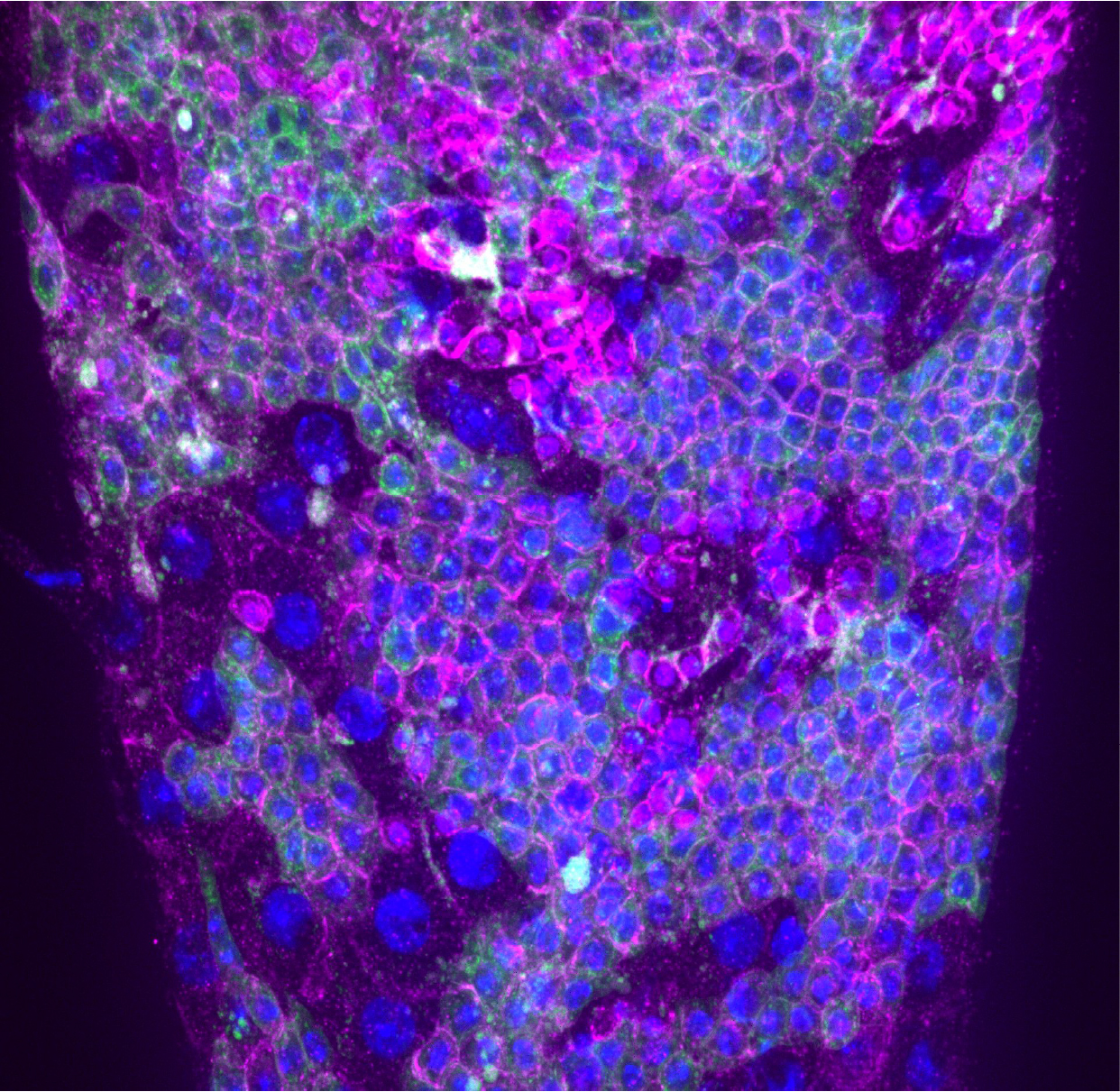

Supplement: Supplementary file 7 — Source data Fig. 7 [file 44318_2026_808_MOESM7_ESM.zip › Fig.7/Panel F/Notch RNAi.jpg]

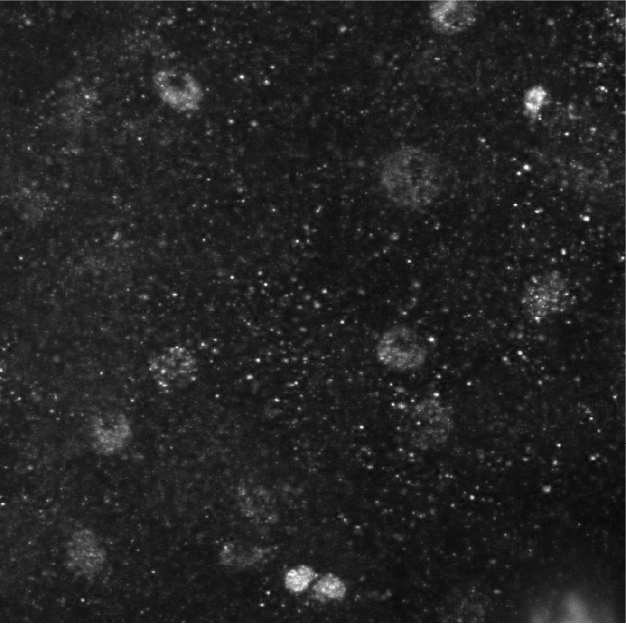

Supplement: Supplementary file 8 — Source data Fig. 8 [file 44318_2026_808_MOESM8_ESM.zip › Fig.8/Panel B/control - Cph YFP.jpg]

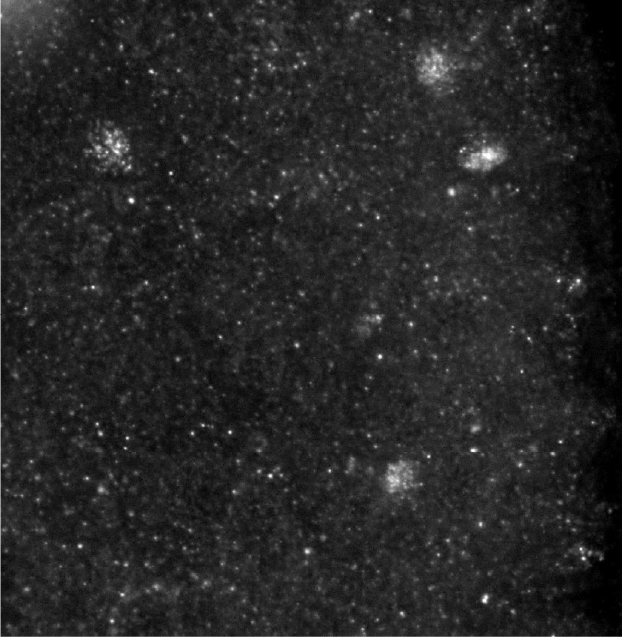

Supplement: Supplementary file 8 — Source data Fig. 8 [file 44318_2026_808_MOESM8_ESM.zip › Fig.8/Panel B/Cph - Cph YFP.jpg]

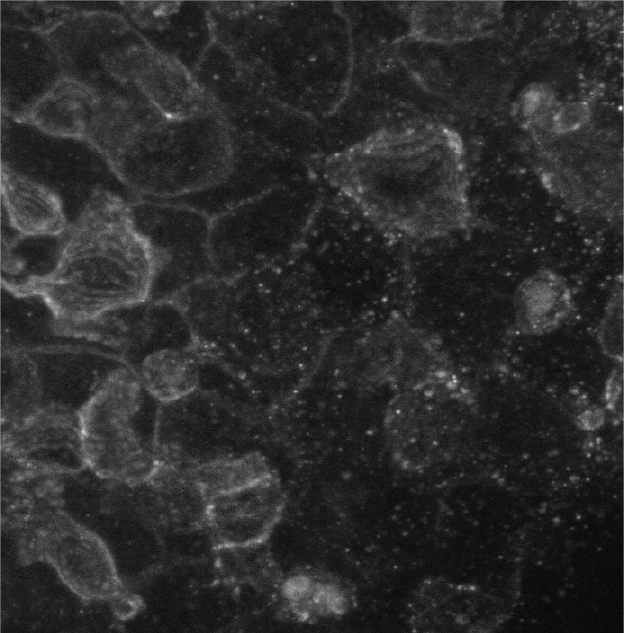

Supplement: Supplementary file 8 — Source data Fig. 8 [file 44318_2026_808_MOESM8_ESM.zip › Fig.8/Panel B/control - Arm:Pros.jpg]

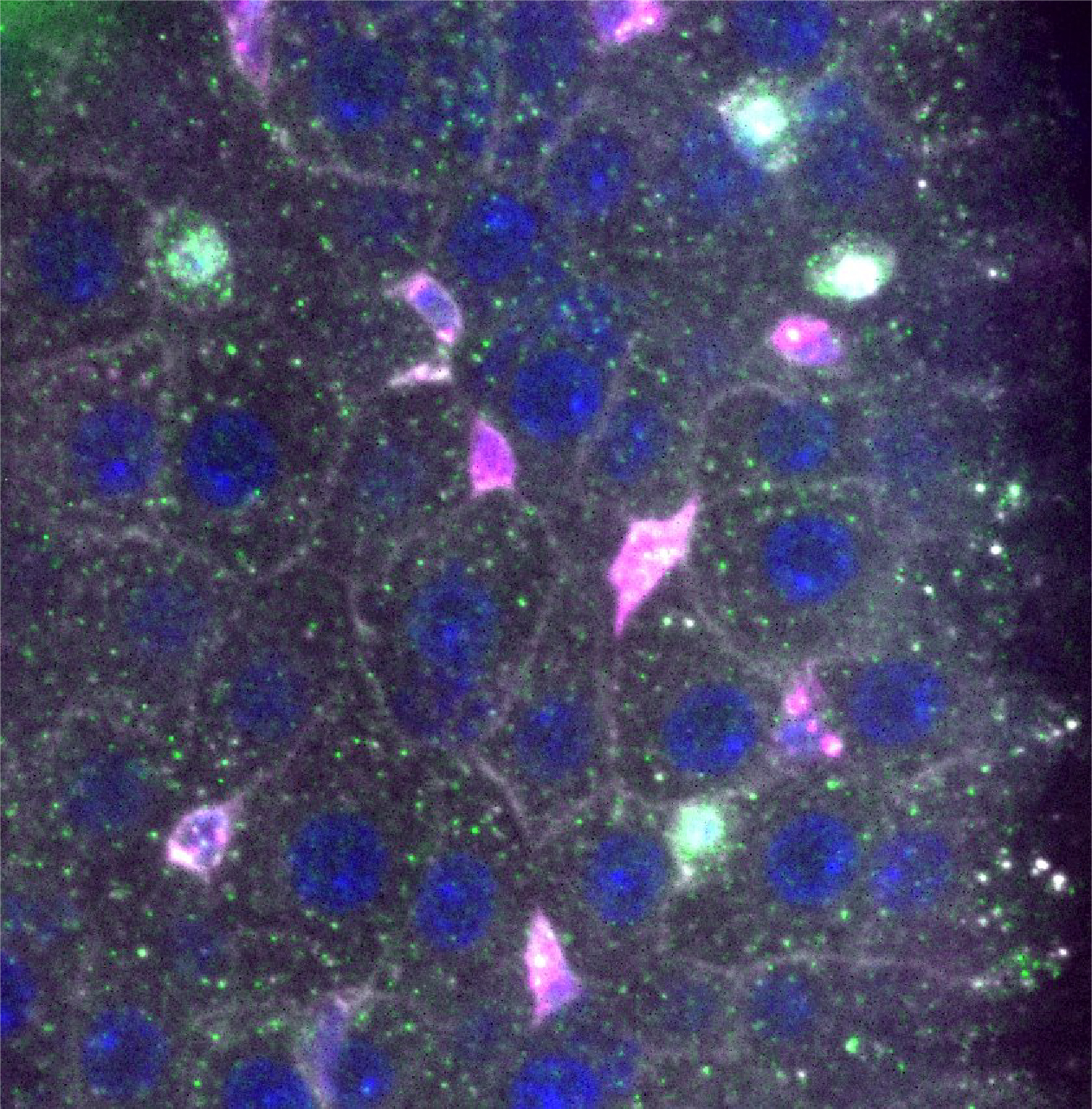

Supplement: Supplementary file 8 — Source data Fig. 8 [file 44318_2026_808_MOESM8_ESM.zip › Fig.8/Panel B/Cph - Merged.jpg]

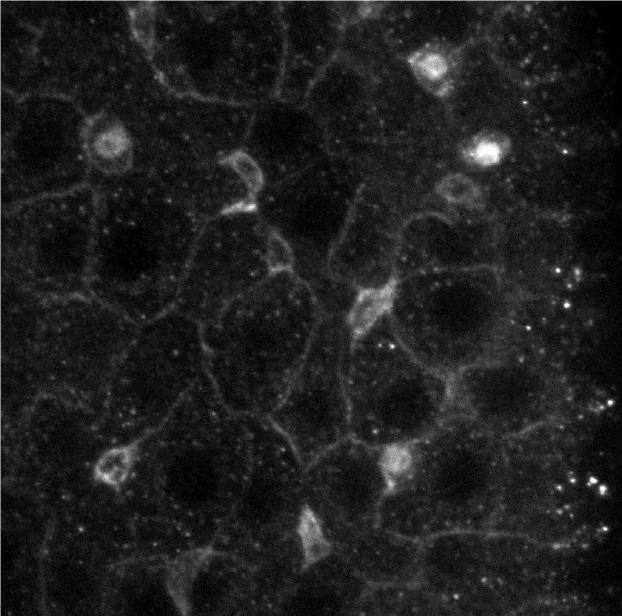

Supplement: Supplementary file 8 — Source data Fig. 8 [file 44318_2026_808_MOESM8_ESM.zip › Fig.8/Panel B/Cph - Arm:Pros.jpg]

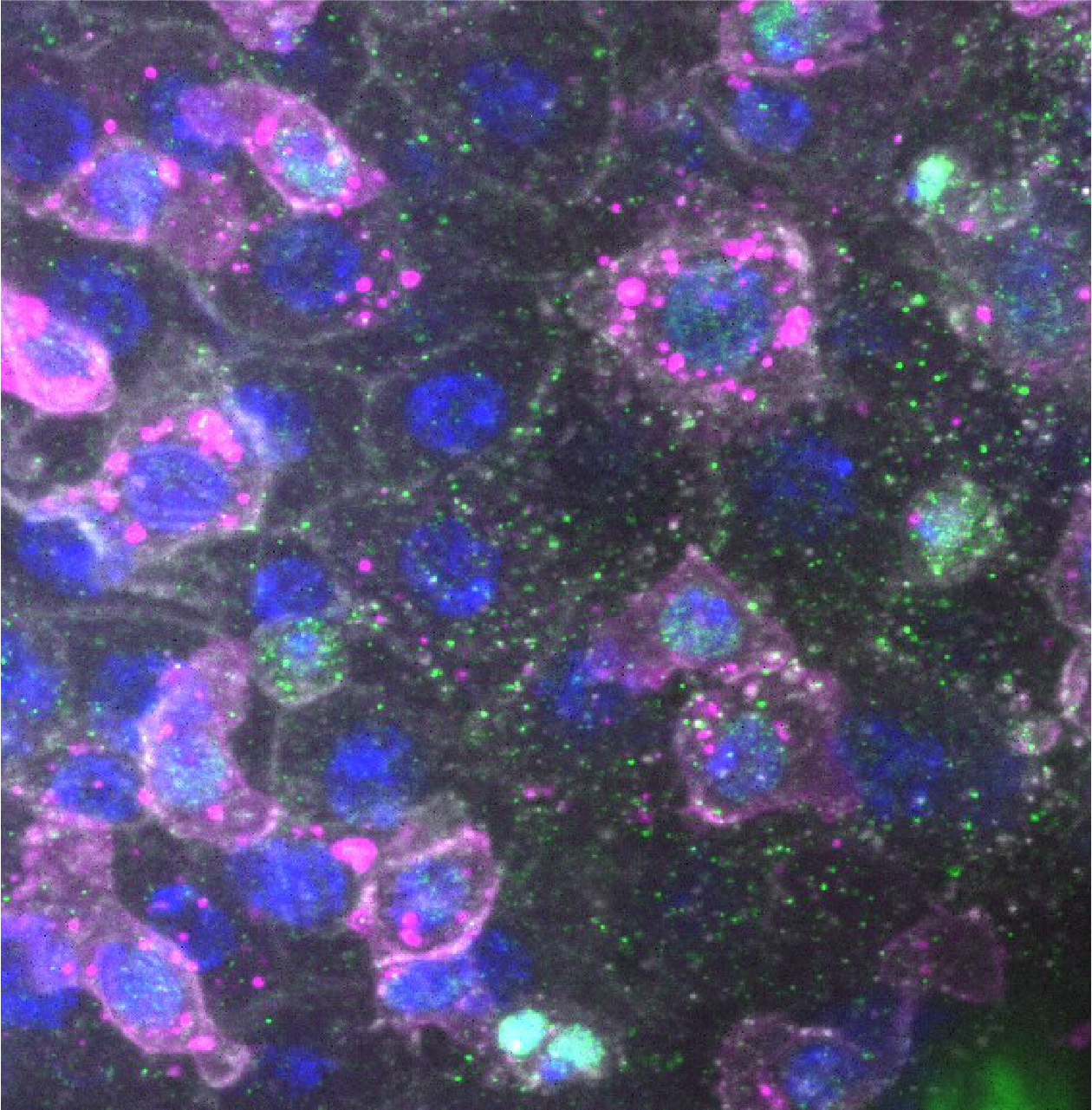

Supplement: Supplementary file 8 — Source data Fig. 8 [file 44318_2026_808_MOESM8_ESM.zip › Fig.8/Panel B/control - Merged.jpg]

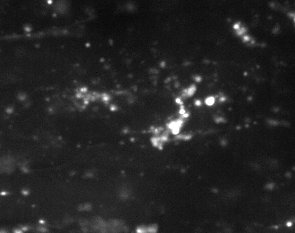

Supplement: Supplementary file 8 — Source data Fig. 8 [file 44318_2026_808_MOESM8_ESM.zip › Fig.8/Panel E/Cph- Atg8a mCherry.jpg]

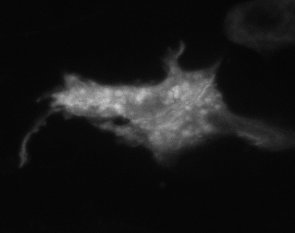

Supplement: Supplementary file 8 — Source data Fig. 8 [file 44318_2026_808_MOESM8_ESM.zip › Fig.8/Panel E/Cph- GFP.jpg]

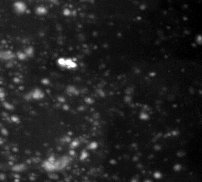

Supplement: Supplementary file 8 — Source data Fig. 8 [file 44318_2026_808_MOESM8_ESM.zip › Fig.8/Panel E/control- Atg8a mCherry.jpg]

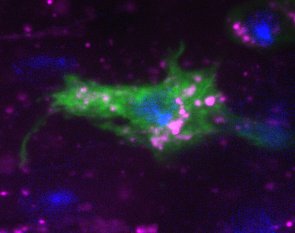

Supplement: Supplementary file 8 — Source data Fig. 8 [file 44318_2026_808_MOESM8_ESM.zip › Fig.8/Panel E/Cph- merged.jpg]

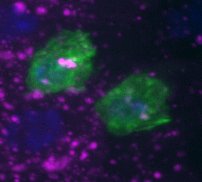

Supplement: Supplementary file 8 — Source data Fig. 8 [file 44318_2026_808_MOESM8_ESM.zip › Fig.8/Panel E/control- merged.jpg]

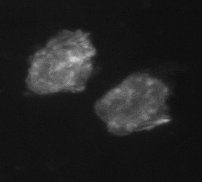

Supplement: Supplementary file 8 — Source data Fig. 8 [file 44318_2026_808_MOESM8_ESM.zip › Fig.8/Panel E/control- GFP.jpg]
