# Supplementary material for: Autoinhibitory feedback preserves intestinal stem cell maintenance and fate commitment
Source: EMBO J. 2026 May 20;45(13):4636–69. doi: 10.1038/s44318-026-00808-x (PMC13324515; doi:10.1038/s44318-026-00808-x)
Supplement: Supplementary file 9 — Expanded View Figures [file 44318_2026_808_MOESM9_ESM.pdf]

Expanded View Figures

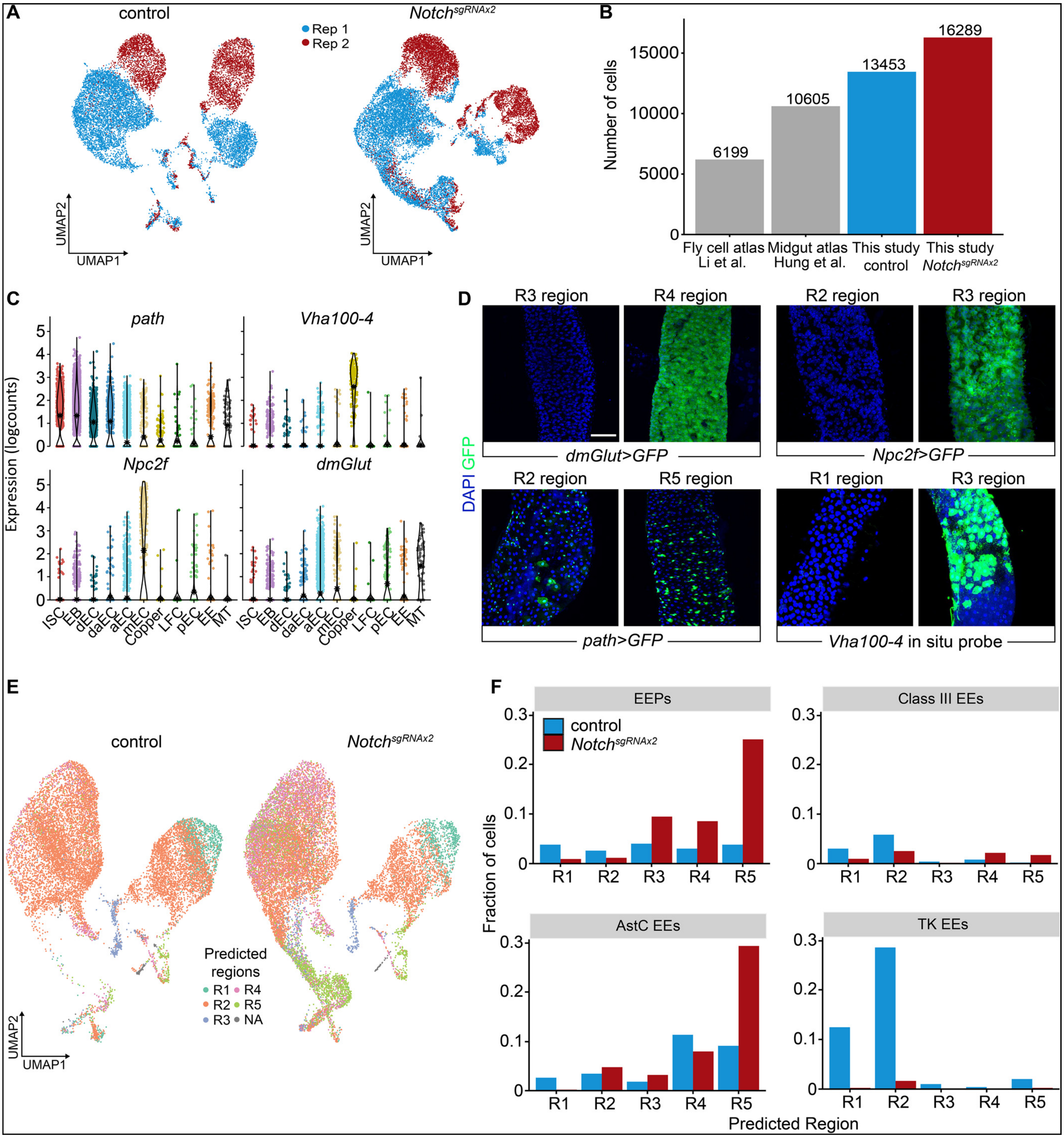

Figure EV1. Quality control, marker gene expression and regional scRNA-seq dataset, related to Fig. 1.

(**A**) UMAP uncorrected for batch effects for each scRNA-seq replicate. (**B**) The number of cells recovered after QC from this study compared to two other studies. (**C**) Violin plot of genes with interesting expression profiles in different intestinal cell types. (**D**) Validation of marker genes in different intestinal regions in vivo, related to (**C**). Scale bar 100 µm. (**E**) UMAP of the regional prediction of cell types from the control and *Notch* mutant conditions. (**F**) Regional predictions for EEs and their subclasses in control and *Notch* mutant conditions. For all images, nuclei are labelled with DAPI. For (**C**), expression profiles are shown from two independent replicates of scRNA-seq with a sample size of 20 flies.

 © The Author(s)

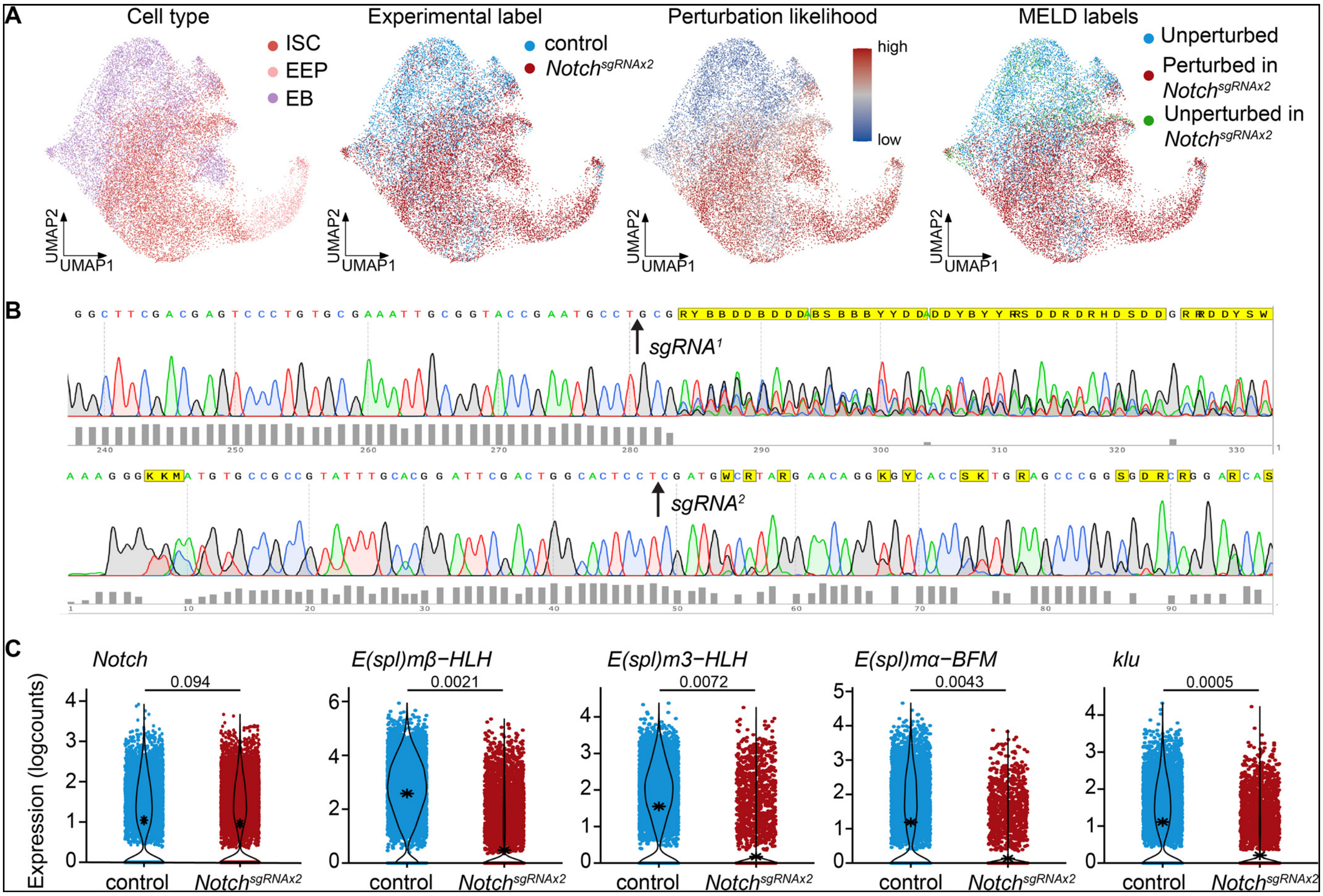

Figure EV2. Identification of perturbed cells using MELD, related to Fig. 2.

(**A**) UMAPs of progenitor cells arranged by cell type, experimental labels and perturbation likelihood, which were used to determine MELD labels. (**B**) Sanger sequencing traces of progenitor-specific *Notch* mutant intestine, highlighting where *sgRNA* starts. (**C**) Expression of Notch and various *Notch* target genes in control and *Notch* mutant conditions. For all comparisons, an asterisk denotes the mean. For (**C**), two independent replicates of scRNA-seq were performed for each condition with a sample size of 20 flies per condition. Statistical test for (**C**) was calculated using the pseudobulk approach and edgeR.

© The Author(s)

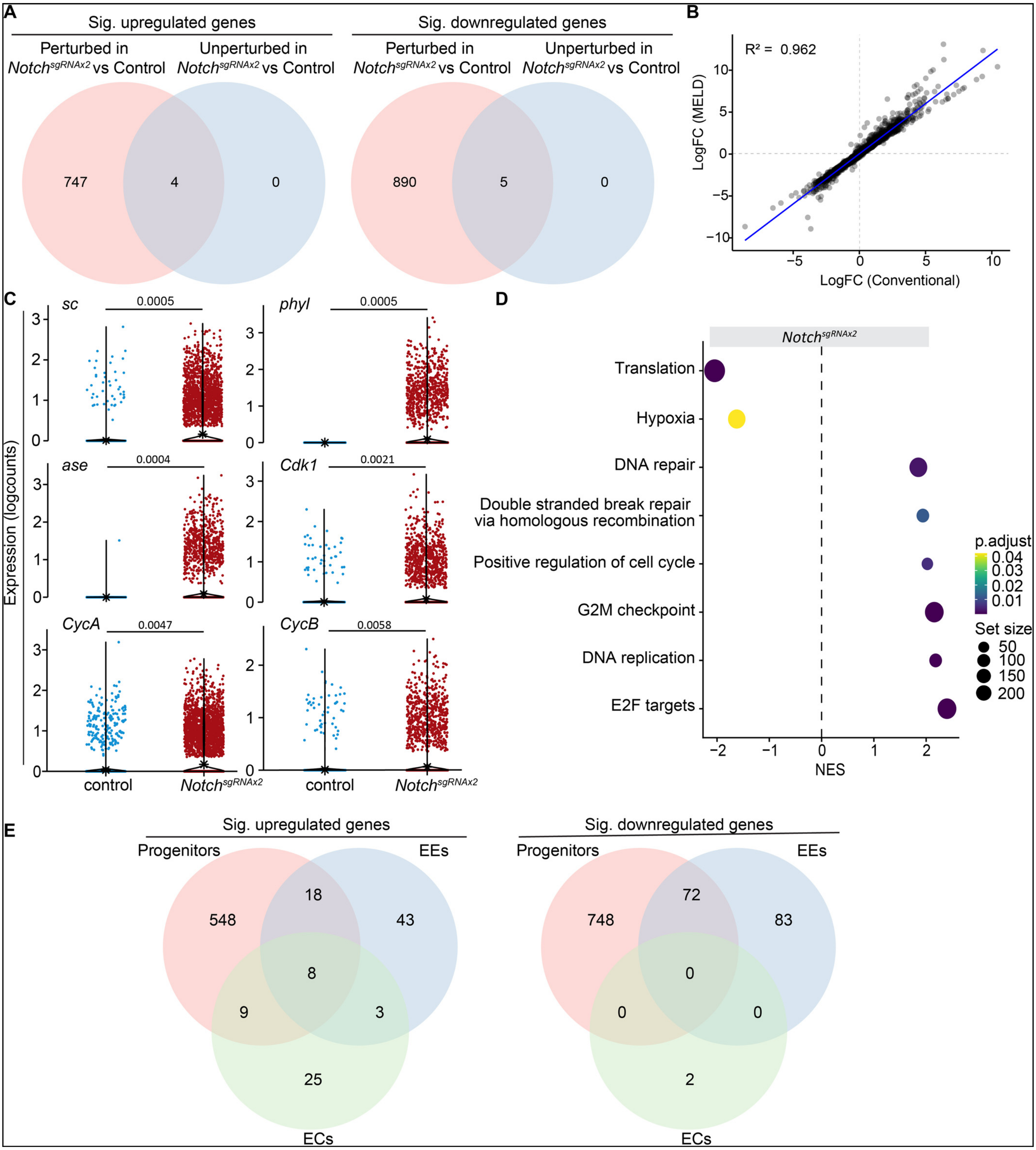

Figure EV3. Identification of DEGs and gene set enrichment analysis, related to Fig. 2.

(**A**) Number of up- and downregulated DEGs in perturbed and unperturbed progenitor cells compared to the control condition. (**B**) Correlation coefficient of DEGs identified by MELD and the conventional method (see "Methods"). (**C**) Expression of various genes in control and perturbed *Notch* mutant progenitor cells. (**D**) Gene set enrichment analysis for *Notch*^{sgRNAx2} condition. (**E**) Number of DEGs that are up- and downregulated in different intestinal cell types. For (**C**), two independent replicates of scRNA-seq were performed for each condition, with a sample size of 20 flies per condition. Significance for (**C**) was calculated using the pseudobulk approach and edgeR, whereas for (**D**) clusterProfiler's GSEA function was used to calculate adjusted significance.

 © The Author(s)

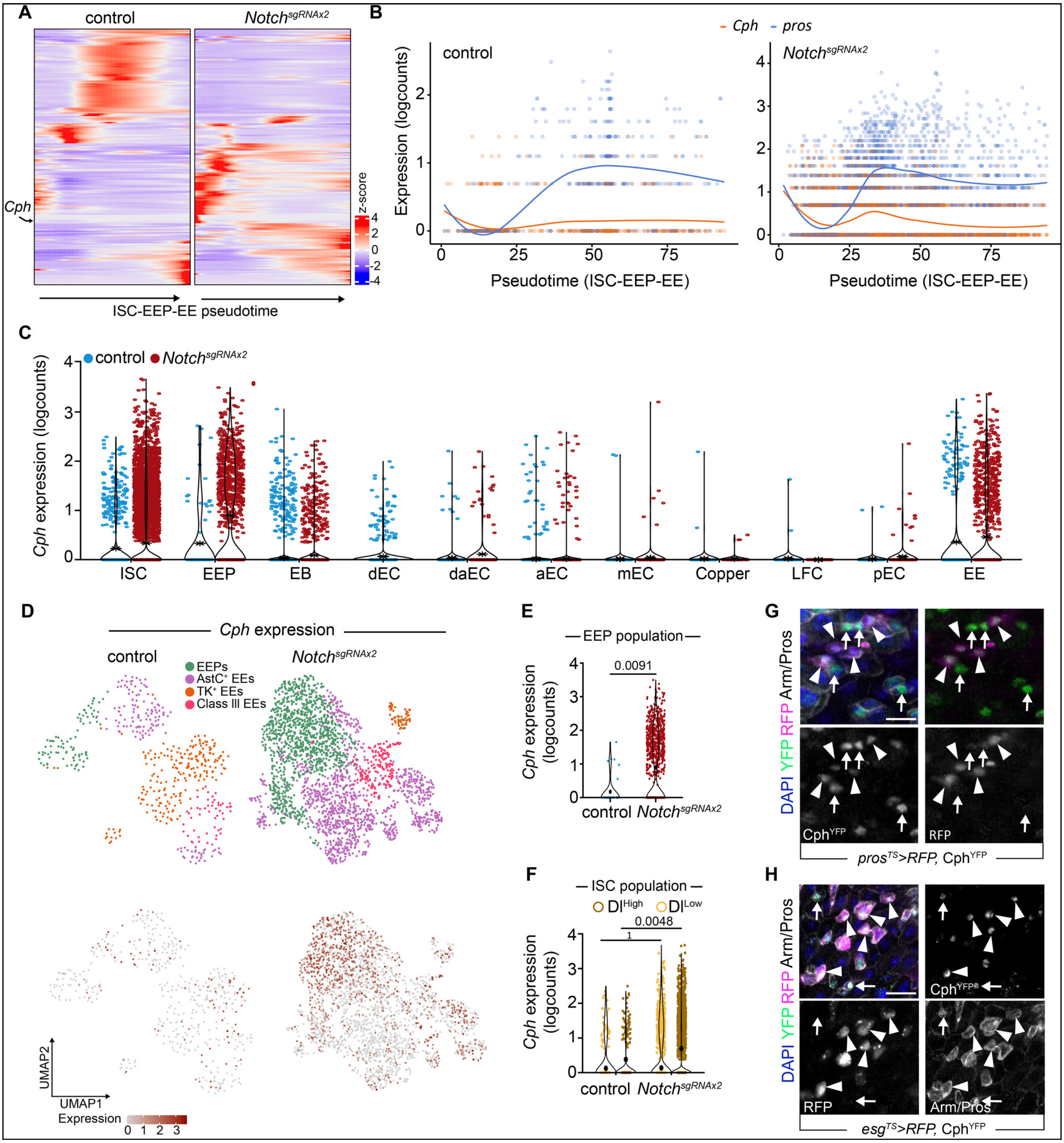
A
control
Notch sgRNAx2
Cph
z-score
ISC-EEP-EE pseudotime
B
Cph
pros
control
Notch sgRNAx2
Expression (logcounts)
Pseudotime (ISC-EEP-EE)
C
control
Notch sgRNAx2
Cph expression (logcounts)
ISC
EEP
EB
dEC
daEC
aEC
mEC
Copper
LFC
pEC
EE
D
Cph expression
control
Notch sgRNAx2
EEPs
AstC+ EEs
TK+ EEs
Class III EEs
UMAP2
UMAP1
Expression
E
EEP population
0.0091
Cph expression (logcounts)
control
Notch sgRNAx2
F
ISC population
Dl High
Dl Low
0.0048
1
Cph expression (logcounts)
control
Notch sgRNAx2
G
DAPI YFP RFP Arm/Pros
Cph YFP
RFP
pros TS>RFP, Cph YFP
H
DAPI YFP RFP Arm/Pros
Cph YFP
RFP
Arm/Pros
esg TS>RFP, Cph YFP

◀ **Figure EV4. *Cph* expression across intestinal cell types and lineages, related to Fig. 2.**

(**A**) Pseudotime expression analysis of all differentially expressed genes along the ISC-EEP-EE lineage under control and *Notch* mutant conditions. (**B**) Expression of *Cph* and *pros* during ISC-EEP-EE differentiation in the homoeostatic condition and *Notch* mutant condition. (**C**) Expression of *Cph* in all intestinal cell types during homeostasis or *Notch* mutant conditions. (**D**) *Cph* expression in the EEP and EE population in control and *Notch* mutant conditions. *Cph* is present in some EEs during homeostasis, and its expression increases mainly in the EEP population. (**E**) Quantification of *Cph* expression specifically within the EEP population under control and *Notch* mutant condition. (**F**) Log counts for *Cph* expression within ISCs that are sub-clustered based on Dl^{High} and Dl^{Low} expression, highlighting that *Cph* expression increases in Dl^{High} ISCs when Notch signalling is perturbed. (**G**) *RFP* expression using the $Pros^{TS}$ driver overlaps with endogenously tagged Cph^{YFP} (arrowheads). Cph^{YFP} is also expressed in non-RFP cells that represent ISCs (arrows). Scale bar 20 μm. (**H**) Cph^{YFP} is found in some progenitor cells (arrowheads) and EEs (arrows) in the male midgut. For all images, nuclei are labelled with DAPI. For (**C–E**), two independent replicates of scRNA-seq were performed for each condition, with a sample size of 20 flies per condition. Scale bar 20 μm. Seurat's FindMarkers function with test.use = "MAST" was used to calculate *P* value for (**E**, **F**).

 © The Author(s)

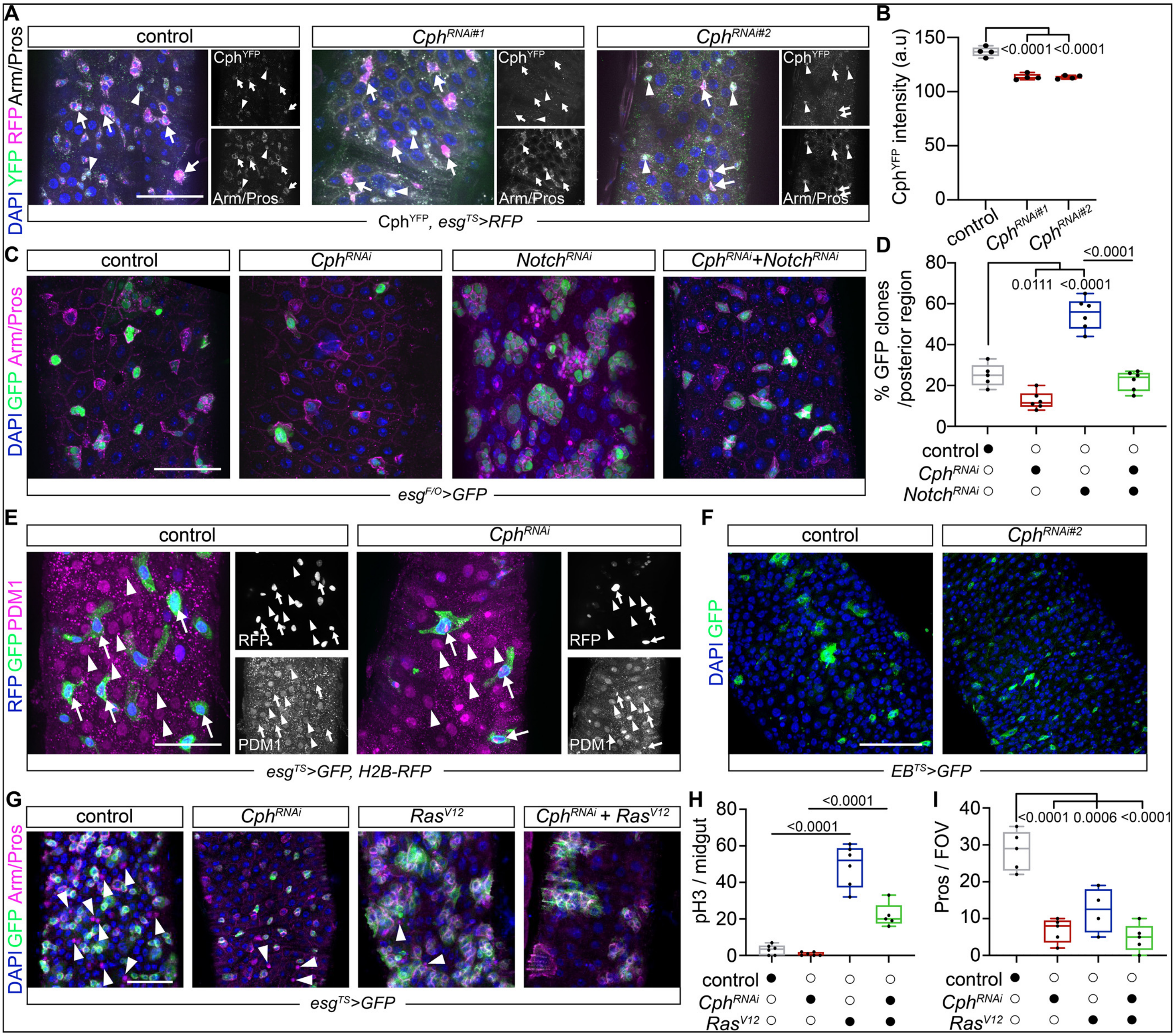

Figure EV5. Functional characterisation of Cph, related to Figs. 3 and 4.

(**A**) Silencing Cph in progenitor cells decreases the intensity of Cph^{YFP} in progenitor cells (arrows) and not in EE (arrowheads). Scale bar 50 µm. (**B**) Quantification of Cph^{YFP} in RFP^{+} progenitor cells. (**C**) Confocal images of Flip-out clones within the posterior midgut under different genetic conditions. Scale bar 100 µm. (**D**) Quantification of the number of GFP clones within the posterior midgut. (**E**) REDDM lineage tracing highlighted that *Cph* depletion causes some progenitor cells to become abnormally large and contain nuclear PDM1. Scale bar 100 µm. (**F**) Confocal images of the posterior midgut when Cph is silenced within EBs. Scale bar 100 µm. (**G**) Confocal images of the posterior midgut under different genetic conditions. Arrowheads point to $Pros^{+}$ enteroendocrine cells. Scale bar 100 µm. (**H**) Quantification of the number of pH3+ mitotically active cells in the midgut. Scale bar 100 µm. (**I**) Quantification of the number of Pros+ enteroendocrine cells in the field of view (FOV). For (**B**), intensity measurements for each condition were taken from one matched experiment, For (**D**), clones for each condition were quantified from one matched experiment, For (**H**, **I**), two independent replicates were done with the following sample size from left to right: (**B**) $n = 4, 4, 4$; (**D**) $n = 5, 6, 6, 6$; (**H**) $n = 6, 6, 6, 5$; (**I**) $n = 5, 5, 4, 5$. For all images, nuclei are labelled with DAPI. Box plots: line, median; box, 75th–25th percentiles; whiskers, minimum to maximum. One-way ANOVA test with Tukey post hoc comparison was used for statistical analysis of all graphs.

© The Author(s)

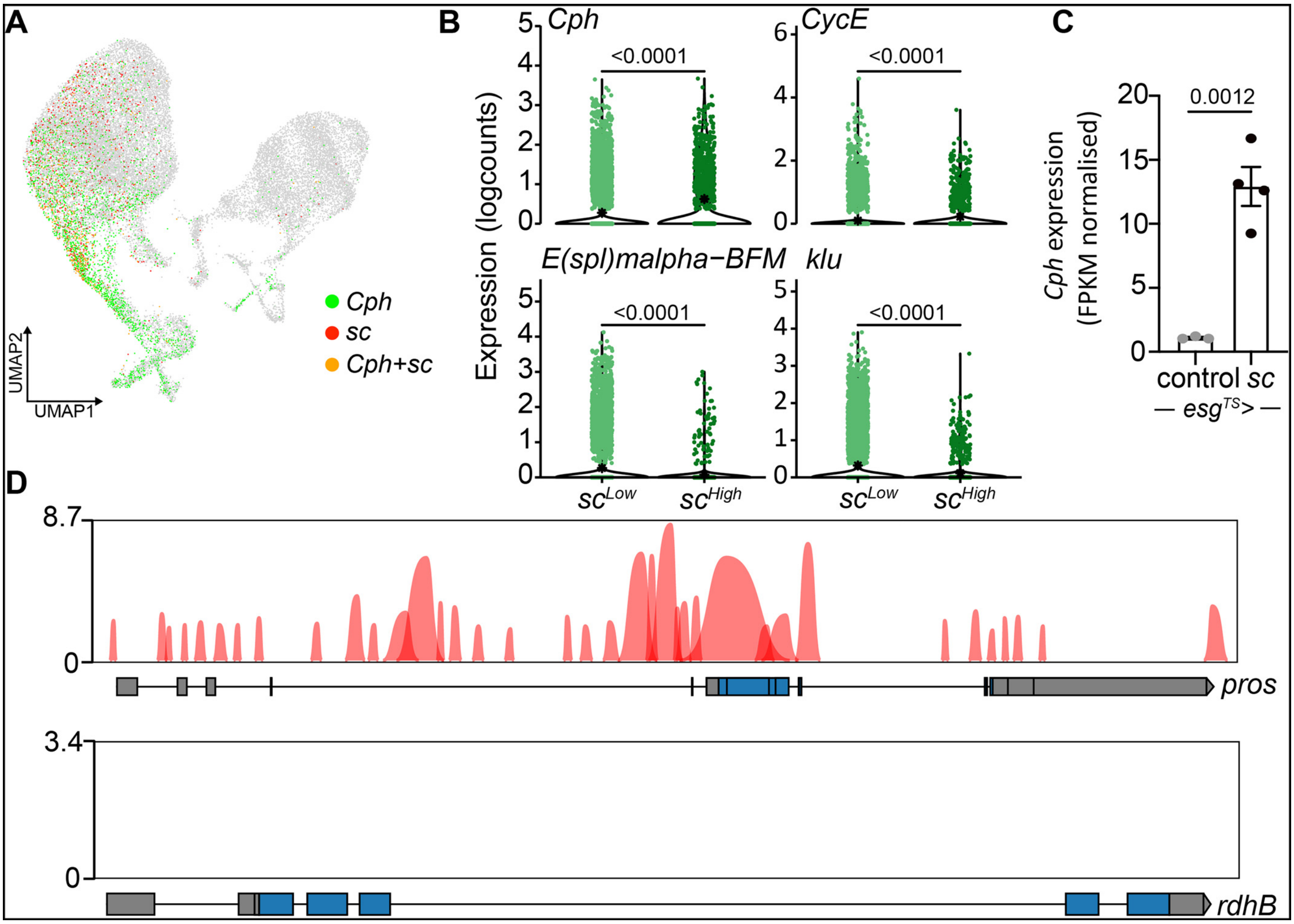

Figure EV6. Characteristics of *sc* expressing intestinal stem cells, related to Fig. 4.

(**A**) UMAP of *Cph*$^{+}$ (green) and *sc*$^{+}$ (red) cells, with co-expression indicated in orange. (**B**) Expression of *Cph*, *CycE*, *E(spl)ma-BFM* and *klu* in sc^{High} and sc^{Low} expressing intestinal cells. Note that *Cph* is expressed in sc^{High} expressing intestinal cells. (**C**) Re-analysis of Bulk RNA-sequencing of *sc* overexpression in progenitor cells (Chen et al, 2018), demonstrating increased *Cph* expression in progenitor cells. (**D**) ChIP-seq tracks for *sc* binding to *pros* and *rdhB*. For (**B**), two independent replicates of scRNA-seq were performed. Each dot represents a single cell. For (**C**), data represent three independent replicates for control and four for sc overexpression. Statistical test for (**B**, **C**) were done using Seurat's FindMarkers function with test.use = "MAST" or PyDESeq2, respectively.

 © The Author(s)

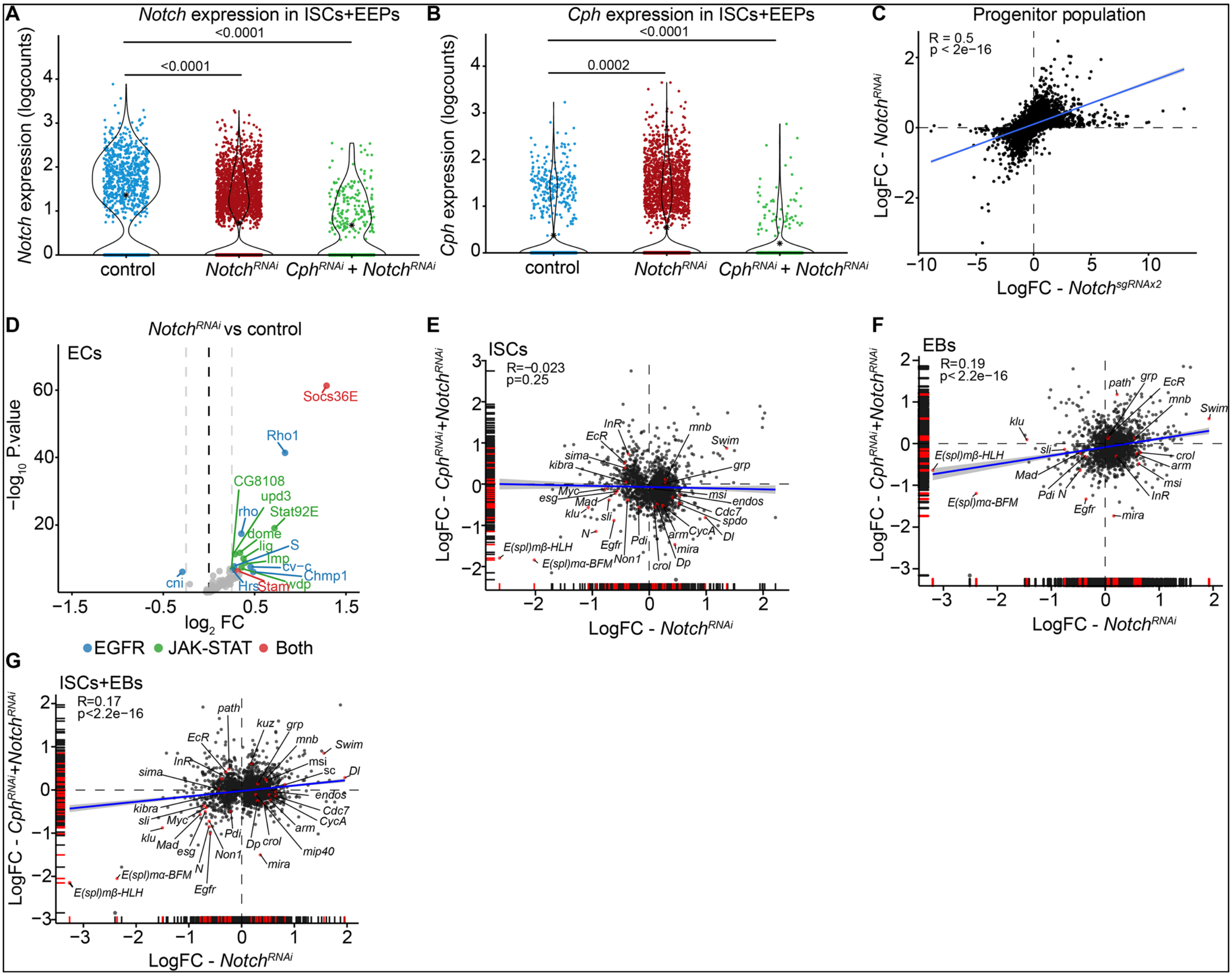

Figure EV7. Transcriptional reprogramming of progenitor cells, related to Fig. 5.

(**A**, **B**) Expression of *Notch* (**A**) and *Cph* (**B**) in ISCs+EEPs under control, *Notch*^{RNAi} and *Cph*^{RNAi}+*Notch*^{RNAi} condition. (**C**) Correlation coefficient comparing differential gene expression results between *Notch*^{RNAi} and *Notch*^{sgRNAx2}. (**D**) Volcano plot of differential gene expression in the EC population in control vs *Notch*^{RNAi}. Genes belonging to the EGFR, JAK-STAT or both signalling pathways are colour-coded. (**E**) Correlation coefficient of all shared genes in ISCs in *Notch*^{RNAi} and *Cph*^{RNAi}+*Notch*^{RNAi} condition. (**F**) Correlation coefficient of all shared genes in EBs in *Notch*^{RNAi} and *Cph*^{RNAi}+*Notch*^{RNAi} condition. (**G**) Correlation coefficient of all shared genes in ISCs and EBs in *Notch*^{RNAi} and *Cph*^{RNAi}+*Notch*^{RNAi} condition. For (**A**, **B**, **D**, **E**), one independent scRNA-seq replicate was performed per condition. Significance for (**A**, **B**, **D**) were calculated using Seurat's FindMarkers function with test.use = "MAST", for (**C**, **E**–**G**) correlation *P* values were computed using a two-sided *t* test on the correlation coefficient.

© The Author(s)

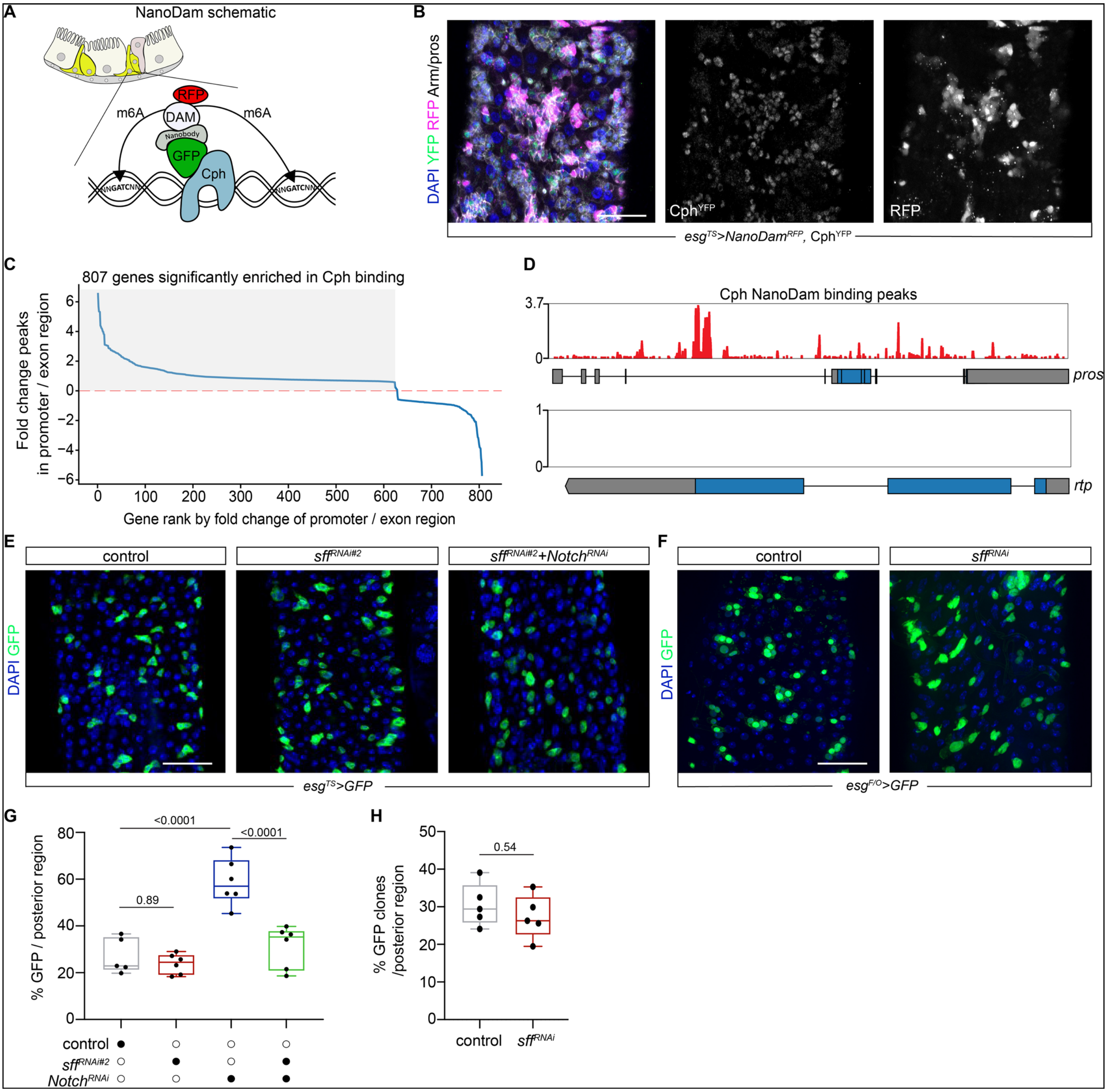

Figure EV8. NanoDam profiling of Cph, related to Fig. 6.

(**A**) Schematic of NanoDam in progenitor cells. (**B**) Expression of *NanoDam*[RFP] in *GFP*[+] progenitor cells using the *esg*[TS] driver. Scale bar 100 µm. (**C**) Significant Cph target genes within the progenitor population. (**D**) Cph NanoDam binding intensity on the *pros* and *rtp* locus. Binding intensities are shown as log_2-fold enrichment. (**E**) Confocal images of the posterior midgut under different genetic conditions. (**F**) Confocal images of Flip-out clones within the posterior midgut under different genetic conditions. (**G**) Quantification of the number of GFP[+] progenitor cells in the posterior region. (**H**) Quantification of the number of GFP[+] clones within the posterior midgut. For (**G**, **H**), a minimum of two independent replicates were done with the following sample sizes from left to right: (**G**) $n = 5, 6, 6, 6$; (**H**) $n = 5, 5$. For all images, nuclei are labelled with DAPI. Box plots: line, median; box, 75th–25th percentiles; whiskers, minimum to maximum. One-way ANOVA test with Tukey post hoc comparison was used for (**G**). Mann–Whitney test was used for (**H**).

 © The Author(s)

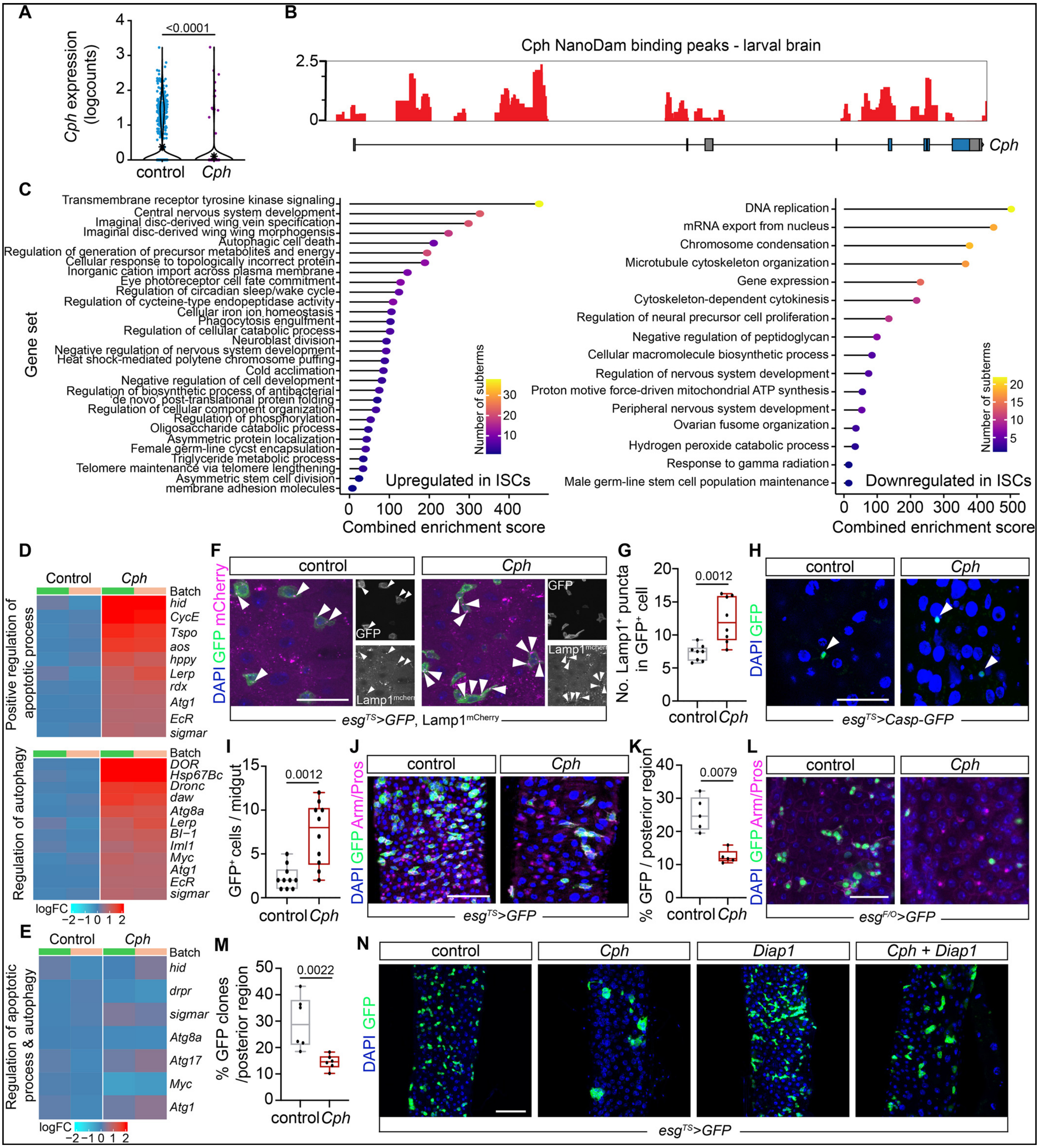
A
Cph expression (logcounts)
<0.0001
control Cph
B
Cph NanoDam binding peaks - larval brain
2.5
0
Cph
C
Gene set
Transmembrane receptor tyrosine kinase signaling
Central nervous system development
Imaginal disc-derived wing vein specification
Imaginal disc-derived wing wing morphogenesis
Autophagic cell death
Regulation of generation of precursor metabolites and energy
Cellular response to topologically incorrect protein
Inorganic cation import across plasma membrane
Eye photoreceptor cell fate commitment
Regulation of circadian sleep/wake cycle
Regulation of cycteine-type endopeptidase activity
Cellular iron ion homeostasis
Phagocytosis engulfment
Regulation of cellular catabolic process
Neuroblast division
Negative regulation of nervous system development
Heat shock-mediated polytene chromosome puffing
Cold acclimation
Negative regulation of cell development
Regulation of biosynthetic process of antibacterial
'de novo' post-translational protein folding
Regulation of cellular component organization
Regulation of phosphorylation
Oligosaccharide catabolic process
Asymmetric protein localization
Female germ-line cycst encapsulation
Triglyceride metabolic process
Telomere maintenance via telomere lengthening
Asymmetric stem cell division
membrane adhesion molecules
Number of subterms
Upregulated in ISCs
Combined enrichment score
DNA replication
mRNA export from nucleus
Chromosome condensation
Microtubule cytoskeleton organization
Gene expression
Cytoskeleton-dependent cytokinesis
Regulation of neural precursor cell proliferation
Negative regulation of peptidoglycan
Cellular macromolecule biosynthetic process
Regulation of nervous system development
Proton motive force-driven mitochondrial ATP synthesis
Peripheral nervous system development
Ovarian fusome organization
Hydrogen peroxide catabolic process
Response to gamma radiation
Male germ-line stem cell population maintenance
Downregulated in ISCs
Combined enrichment score
D
Control Cph
Positive regulation of apoptotic process
Batch
hid
CycE
Tspo
aos
hppy
Lerp
rdx
Atg1
EcR
sigmar
Regulation of autophagy
Batch
DOR
Hsp67Bc
Dronc
daw
Atg8a
Lerp
Bl-1
Iml1
Myc
Atg1
EcR
sigmar
logFC
F
control Cph
DAPI GFP mCherry
GFP
Lamp1mcherry
esgTS>GFP, Lamp1mCherry
G
No. Lamp1+ puncta in GFP+ cell
0.0012
control Cph
H
control Cph
DAPI GFP
esgTS>Casp-GFP
I
GFP+ cells / midgut
0.0012
control Cph
J
control Cph
DAPI GFP Arm/Pros
esgTS>GFP
K
% GFP / posterior region
0.0079
control Cph
L
control Cph
DAPI GFP Arm/Pros
esgF/O>GFP
E
Control Cph
Regulation of apoptotic process & autophagy
Batch
hid
drpr
sigmar
Atg8a
Atg17
Myc
Atg1
logFC
M
% GFP clones /posterior region
0.0022
control Cph
N
control Cph Diap1 Cph + Diap1
DAPI GFP
esgTS>GFP

Figure EV9. Sustained expression of Cph induces autophagy and cell death, related to Fig. 7.

(**A**) Expression of Cph derived from scRNA-seq of control and progenitor-specific Cph overexpression condition. (**B**) NanoDam profiling of Cph in the *Drosophila* larval brain reveals self-binding. Binding intensities are shown as log_2-fold enrichment. (**C**) Overrepresentation analysis of upregulated and downregulated terms in ISCs overexpressing *Cph*. (**D**) Heatmap of cell death-related genes and autophagy-related genes specifically in ISCs across two independent scRNA-seq replicates in control and *Cph* overexpression conditions. Log fold change was calculated with respect to the average control expression. (**E**) Heatmap of cell death-related genes and autophagy-related genes in EBs. (**F**) Confocal images of endogenously tagged $Lamp1^{mCherry}$ in GFP^+ progenitor cells within the posterior region. Scale bar 40 µm. (**G**) Quantification of the number of $Lamp^+$ puncta in GFP^+ progenitor cells within the posterior midgut. (**H**) Confocal images of the apoptotic sensor Casp-GFP in the posterior midgut. Scale bar 40 µm. (**I**) Quantification of the number of Casp-GFP^+ cells in the midgut. (**J**) Confocal images of the posterior midgut showing that overexpression of Cph in progenitor cells decreases the number of progenitor cells. (**K**) Quantification of the number of GFP+ progenitor cells in the posterior midgut. (**L**) Confocal images of Flip-out clones within the posterior midgut under control and progenitor-specific Cph overexpression condition. Scale bar 40 µm. (**M**) Quantification of GFP+ clones within the posterior region. Scale bar 100 µm. (**N**) Confocal images of GFP^+ progenitor cells in the posterior midgut. Scale bar 100 µm. For (**A**), two independent scRNA-seq replicates were done for each condition. Each dot represents a single cell. For (**G**, **I**, **M**), a minimum of two independent replicates were done, For (**K**), a single matched condition was quantified with the following sample size from left to right: (**G**) $n = 8, 8$; (**I**) $n = 10, 10$; (**K**) $n = 5, 5$; (**M**) $n = 6, 6$. For all images, nuclei are labelled with DAPI. Box plots: line, median; box, 75th–25th percentiles; whiskers, minimum to maximum. Mann–Whitney test was used for all graphs, except for (**A**), where the Seurat's FindMarkers function with test.use = "MAST" was used to calculate *P* value.

 © The Author(s)
