# Supplementary material for: Autoinhibitory feedback preserves intestinal stem cell maintenance and fate commitment
Source: EMBO J. 2026 May 20;45(13):4636–69. doi: 10.1038/s44318-026-00808-x (PMC13324515; doi:10.1038/s44318-026-00808-x)
Supplement: Supplementary file 1 — Peer Review File [file 44318_2026_808_MOESM1_ESM.pdf]

THE EMBO JOURNAL

Autoinhibitory feedback preserves intestinal stem cell maintenance and fate commitment

Siamak Redhai, Nick Hirschmüller, Tianyu Wang, Tyler Jackson, Stefan Peidli, Erica Valentini, Shivohum Bahuguna, Svenja Leible, Sarina Möller, Christina Ko, Michaela Holzem, Sviatoslav Kharuk, Lea Braeckow, Fillip Port, David Ibberson, Hongjie Li, Wolfgang Huber, and Michael Boutros

Corresponding author(s): Michael Boutros (m.boutros@dkfz.de) , Siamak Redhai (Siamak.Redhai@dkfz-heidelberg.de), Wolfgang Huber (wolfgang.huber@embl.de)

Review Timeline:

Submission Date:	10th Nov 24
Editorial Decision:	18th Dec 24
Appeal Received:	22nd Oct 25
Editorial Decision:	23rd Nov 25
Revision Received:	8th Dec 25
Accepted:	28th Apr 26

Editor: Ieva Gailite

Transaction Report:

(Note: With the exception of the correction of typographical or spelling errors that could be a source of ambiguity, letters and reports are not edited. Depending on transfer agreements, referee reports obtained elsewhere may or may not be included in this compilation. Referee reports are anonymous unless the Referee chooses to sign their reports.)

1st Editorial Decision 18th Dec 2024

Dear Dr. Boutros,

Thank you for submitting your manuscript for consideration by The EMBO Journal. We have now received two reviewer reports on your manuscript, which are included below for your information. Since these assessments are in a good agreement, I am taking the decision on their basis to avoid further delays. Based on the provided comments, we unfortunately had to conclude that the study is not a sufficiently strong candidate for publication in The EMBO Journal.

As you can see, both reviewers appreciate the presented single cell transcriptomics analysis and find the identification of Cph as a regulator of ISC proliferation and differentiation interesting. However, they also point out that the specific functional role of Cph in the ISC proliferation/maintenance vs enteroendocrine cell differentiation is insufficiently clarified. They also have concerns regarding the proposed positioning of Cph under transcriptional control by Sc. Finally, in addition to various technical concerns, such as a need to use multiple RNAi lines, they find that further exploration is needed into the role of Cph in a setting where Notch signalling is unperturbed. Since these experiments would require substantial revision work with an unpredictable outcome, I am afraid that I cannot explicitly offer to invite a revised version of the manuscript at The EMBO Journal.

Nevertheless, if you find that you can address all or most of the reviewers' concerns, I would be willing to reconsider the revised manuscript. In such a case, I would send it back to the same reviewers, if possible, but would allow them to make new comments on the added data, which might then have to be further addressed if the reviewers are more positive in this round of assessment.

Thank you in any case for the opportunity to consider this manuscript. I regret that I could not communicate more positive news, but I nevertheless hope that you will find our reviewers' comments helpful for further improvement of the manuscript.

With kind regards,

Ieva Gailite

Ieva Gailite, PhD
Senior Scientific Editor
The EMBO Journal
Meyerhofstrasse 1
D-69117 Heidelberg
Tel: +4962218891309
i.gailite@embojournal.org

Referee #1:

In this manuscript, Redhai et al. found that Cph expression was significantly increased in Notch inactivation-induced tumors by comparing single-cell sequencing results after Notch knockdown. Their results showed that Cph is essential for ISC maintenance and division, and when Cph is knocked down along with Notch knockdown, the Notch mutant tumor fails to grow. Chromatin profiling showed that Cph has direct binding to genes related to EEC differentiation, suggesting that Cph is involved in EEC differentiation. They also found that overexpression of Cph resulted in decreased expression of the Cph-YFP reporter, suggesting that overexpression of Cph has a repressive effect on its own transcription.

The large amount of single cell transcriptomic data in this manuscript constructively contributes to the study of Drosophila midgut homeostasis. It has significant reference value for Notch signaling in ISC maintenance and differentiation. The discovery of the cph gene by single cell sequencing sheds new light on understanding the phenotypes caused by Notch mutations. Unfortunately, in this study, mutation of cph resulted in arrest of ISC division and differentiation of ISCs into polyploid cells; and overexpression of cph led to ISC apoptosis. Therefore, it is difficult to phenotypically clarify the role of cph in the differentiation of ISCs into EECs. The authors require further experiments to support their conclusions.

Major points:

1. On line 104-105, line 112-123, the authors wrote "When Notch signaling is high/When Notch signaling is low", citing an article published by the Spradling lab in 2007. In this literature, high/low Notch activation refers to the daughter cell of the ISC being in a high or low state while the ISC is in the state of Notch inactivation (Ohlstein, B. & Spradling, 2007). However, in this manuscript the authors found that high/low Notch signaling occurs in ISCs and that Notch knockdown results in increased

expression of Sc and cpf in ISCs. Therefore, the authors should specifically write "When Notch signaling is high in ISCs/When Notch signaling is low in ISCs". The data in this manuscript are reminiscent of bidirectional Notch activation, in which EMC/EEPs expressing Dl activate Notch signaling in ISCs (Guo and Ohlstein 2015).

2. Figure 1E is misleading. I thought the small black and white panels were the separate channels of the RGB images. I later realized that the author was showing different midguts with different antibody staining. The author should remake this figure to show the GFP signal, Pros and Tk (and AstC) staining in the same midgut.

3. In line 252, the authors claim that Cpf-YFP is expressed in ISCs. However, in Figure 2F, the Cph-YFP signal cannot be distinguished in EBs or in ISCs. I noticed that there are pairs of Cph-YFP+ cells in the control image of Figure 2F, indicating that Cpf is expressed in both ISC and EB. The author should use NRE-lacZ; esg>RFP stock to distinguish Cpf expression in ISC or EB. Again, it is very clear that pairs of Cpf-GFP cells are present in Figure EV4.

4. Line 274, are the large cells in Figure 3B ECs? The author should stain Pdm1 to check the cell fate. And the author should use NRE-lacZ, the Notch reporter, to check Notch activation in the esgts>cpf RNAi midgut. Also, in Figure EV6A, the shape of the esgts>cpf RNAi GFP+ polyploid cells reminds me of the typical mTOR activation phenotype in ISCs (Amcheslavsky, Ito et al. 2011, Kapuria, Karpac et al. 2012). The genetic interaction between cpf and mTOR signaling should be considered to understand the molecular functions of cpf in ISCs during tissue homeostasis.

5. line 287-290, "......suggesting that Cph is involved in maintaining the progenitor and EE populations (Figure 3C-E)". However, the reduction in the number of EECs does not prove that cpf is necessary for EEC differentiation. Since knockdown of cpf results in the loss of ISC, the absence of ISC naturally results in fewer EECs. Not only is the number of EECs reduced, but the number of newly generated ECs is also reduced. In my opinion, the cpf RNAi phenotype is mainly related to ISC division and maintenance. The authors need to demonstrate with more genetic experiments that knockdown of cpf leads to reduced differentiation of EEC. For example, counting the percentage of ISC daughter cells that differentiate into EEC and the percentage that differentiate into EB-EC in the esgts F/O or ReDDM >cph RNAi lineage.

6. Figure 3C is misleading. The Astc and Tk staining panels are not in the same gut as the first row of esgts>GFP panels. The author should show the GFP and AstC/Tk staining in the same midgut regions. The PH3 staining is not necessary, so the author can put the AstC/Tk staining in the red channel.

7. In lines 294-296 the authors claim that cpf RNAi reversed EEC differentiation. However, cpf RNAi inhibited the proliferative capacity of Notch RNAi ISCs, so there was no production of new EECs. Therefore, AstC and Tk were stained for residual EECs in Figure 3C. If the authors insist that cpf RNAi reversed Notch RNAi EEC differentiation, then the authors should perform staining for AstC and Tk in ReDDM>cpf RNAi+Notch RNAi lineage tracing experiments to demonstrate that the cells staining for AstC and Tk are H2B-RFP+ cells.

8. Figure 4A analyses the expression of cpf in the case of Notch knockdown. They show that Cpf is expressed in Pros+ Sc+ cells. However, Figure 2F and 4B shows that Cpf is expressed in most ISCs and EBs that do not express Sc, suggesting that Cpf expression is not dependent on Sc. Furthermore, Figure 4D does not show that Sc binds to the TSS site of cph, and the binding site shown is in the exon of cph. Further experiments are required to determine whether Sc binds directly to cph DNA to regulate cph transcription. For example, knock down Sc in the context of Notch knockdown and observe the expression of Cpf-YFP.

9. Figure 4E-G does not demonstrate that cpf is downstream of sc. Knockdown of any of the housekeeping genes or knockdown of cell cycle related genes may result in fewer cell proliferation and fewer AstC+ cells from Sc overexpression. More rigorous experiments are needed to support the conclusions drawn by the authors in lines 334-336. For example, using Crispr-Cas9 to mutate the E-box binding motif GCAGGTGT within the cph locus to test for reduced transcript levels in cpf.

10. In Figure 5B and lines 355-363, the author observed that CphRNAi+NotchRNAi leads to an increase in the number of ECs. However, the conclusion that cpf knockdown leads to a shift in the direction of ISC differentiation towards EB-ECs is not appropriate. According to the literature published by Edgar's group (Patel, P.H., Dutta, D. & Edgar, B.A., 2015), Notch RNAi leads to tumors that squeeze the neighboring EC, resulting in the loss of ECs, which in turn promotes further tumor growth. cpf RNAi leads to a drastic reduction in the cell's ability to proliferate (Figure 3D and 4F), therefore Notch RNAi is unable to form tumors that invade the basement membrane occupied by ECs, resulting in no reduction in the number of EC cells.

To support the conclusion in line 362-363, the author should perform lineage tracing experiments. Make Notch mutant/RNAi MARCM clones with cpf RNAi expressed inside the clone cells to show that differentiation is restored as in the WT control MARCM clones.

11. For the transcriptional analysis of NotchRNAi and CphRNAi+NotchRNAi mdiguts (Fig. 5C-F), a key argument is: Is the alteration of the Notch RNAi ISC transcriptome by cpf RNAi direct or indirect? Due to the squeezing effect of Notch RNAi tumors on ECs and the proliferative effect of EC on Notch RNAi tumors via upd3 and EGFR ligands, if cpf RNAi affects the proliferation of Notch RNAi tumors, it will indirectly affect the expression of a number of genes.

The ideal analysis (not mandatory) would be to compare single cell sequencing of CphRNAi+NotchRNAi with sequencing of single cells prior to tumor generation by Notch RNAi.

12. In Figure 6F, the GFP signal is abnormal in sff RNAi+Notch RNAi. To me, it looks like sff knockdown resulted in ISC cell death. I am curious about the phenotype of ssf knockdown. Since the authors claimed that sff is not expressed in ISCs during homeostasis, then esgts and esgF/O>sff RNAi should not induce ISC loss phenotype.

Referee #2:

Overall Assessment
The bioinformatics analysis in this study is thorough and robust. However, the subsequent cell biological investigations aimed at elucidating causality are notably weak, often lacking quantitative rigor and comprehensive exploration.

General Comments
This study exhibits three critical weaknesses that should be experimentally addressed:
1. The authors heavily rely on Notch inhibition as a contextual framework, which is both artificial and non-physiological. A fundamental, more biologically relevant question is the role of Cph under normal physiological conditions. By extensively employing an artificial Notch inhibition model and then extrapolating their findings to physiological conditions, the authors risk drawing overly general and potentially misleading conclusions. It is imperative that they clearly delineate the artificial context from the physiological one. Moreover, they should explore the function of Cph under normal, homeostatic conditions to enhance the biological relevance of their findings.
2. The simultaneous regulation of proliferation and differentiation by Cph is insufficiently clarified. Are these two processes interdependent? Investigating potential crosstalk between these pathways could yield deeper biological insights.
3. In the experimental parts, the data often lack quantitative rigor and thorough investigation. Relying solely on single RNAi experiments is problematic. The authors must employ multiple RNAi constructs (for both Cph and sff) to validate their findings. The authors also must specify the region of the gut analyzed in their data, as it significantly influences interpretation. Although the study focuses on female flies, recent evidence highlights the influence of both sex and genetic background on intestinal stem cell (ISC) proliferation. Including data from males and varied genetic backgrounds is essential for robust conclusions.
Specific Comments

Figure 1:
The authors show that Notch inhibition alters the balance of type 1 and type 2 enteroendocrine (EE) cells while leaving class 3 unaffected. Why, then, does this manipulation affect the total EE cell number? This inconsistency is confusing and warrants clarification.

EV2b:
The Sanger sequence should display several patterns of CRISPR-induced mutations, not just one. Is this data derived from a single cell?

Figure 2F:
The expression of Cph in Prospero-positive cells is challenging to appreciate. Improved presentation or clarification is needed.

Figures 3B and 3C:
These data require quantification. Additionally, the reduction in progenitor cell numbers shown in Figure EV6 could result from decreased proliferation or increased differentiation rather than cell death. Experimental clarification is necessary. The authors must employ multiple RNAi or gRNA constructs for Cph knockdown. Describing the effect of Cph RNAi under conditions without Notch inhibition would provide more biologically meaningful insights.

Figures 4C-D:
The figure itself should be explicitly stated that this represents a re-analysis of previously published data. Relying on existing RNA-seq datasets to infer Scute overexpression's impact on Cph expression, rather than directly demonstrating this, is an unusual choice. Ideally, the authors should validate this finding using their NanoDam system, gel shift assays, or other direct approaches.

Figure 6F:
Multiple RNAi constructs for sff are necessary. Demonstrating the normal function of sff during homeostatic conditions would also be more relevant. Regarding NanoDam, does the GFP-recognizing nanobody also recognize YFP?

Figure 7:
The proposed negative feedback loop lacks experimental support and may involve post-translational regulation. In Figure 6, Cph is suggested to enhance gene expression, yet here it is proposed to suppress it. If the authors are positing such a dual role, they

must provide a mechanistic explanation for this discrepancy.

The claims regarding autophagy and cell death are unsubstantiated. The authors fail to provide evidence that autophagy or apoptosis occurs. They should be aware of the relevant literature on autophagy in ISCs (e.g., Nagy et al., 2018; Zhang et al., 2019) and acknowledge that ISCs are generally highly resistant to cell death. Furthermore, autophagic cell death is a rare phenomenon requiring substantial experimental validation, none of which is provided here.

** As a service to authors, EMBO Press provides authors with the possibility to transfer a manuscript that one journal cannot offer to publish to another EMBO publication or the open access journal Life Science Alliance launched in partnership between EMBO Press, Rockefeller University Press and Cold Spring Harbor Laboratory Press. The full manuscript and if applicable, reviewers' reports, are automatically sent to the receiving journal to allow for fast handling and a prompt decision on your manuscript. For more details of this service, and to transfer your manuscript please click on Link Not Available. **

Please do not share this URL as it will give anyone who clicks it access to your account.

Manuscript: EMBOJ-2024-119585.

Response to reviewers

We sincerely thank the reviewers for their insightful comments and constructive suggestions, which have helped us significantly improve the clarity and rigor of our study. In response, we have conducted a series of additional experiments that address the key concerns raised. These new data, together with our original findings, reinforce the robustness of our proposed model - *Cph* regulates intestinal stem cell maintenance, proliferation and enteroendocrine cell differentiation, and its autoregulation guards against autophagy and cell death.

We have summarized the key revisions below, which include the addition of a new main figure (Fig. 4), as well as new data panels in Figures 2, 4, 5, 7, 8 and Figures EV4, EV5, EV8, and EV9.

On the role of Cph during steady state:

- Through clonal analysis and use of cell type–specific drivers, we now show that *Cph* intrinsically regulates both intestinal stem cell maintenance and EE differentiation, and that these processes are functionally separable (response to Reviewer 1, point 5, 11; Reviewer 2, general point 1, 2, specific point 4).
- We further demonstrate that *Cph* activity in intestinal stem cells is essential to sustain normal lifespan, underscoring its physiological significance (Reviewer 2, general point 1).

On the regulatory relationship between sc and Cph:

- By analyzing sc ChIP-seq data and leveraging an endogenously tagged Cph protein trap line, we establish that sc binds to transcriptional start sites within the *Cph* locus (Reviewer 1, point 8; Reviewer 2, point 4).
- Our experiments show that sc is both necessary and sufficient to regulate Cph protein levels (Reviewer 1, point 8; Reviewer 2, point 4).

On the autoregulation of Cph

- We demonstrate that *Cph* represses its own expression.
- Overexpression of *Cph* increases the expression of autophagy and apoptotic-related genes in intestinal stem cells (Reviewer 2, specific point 7).
- Using autophagy and cell death reporters, we show that unrestrained *Cph* expression induces autophagy and apoptosis in *vivo* (Reviewer 2, specific point 7).
- Importantly, blocking apoptosis partially rescues the stem cell population, identifying apoptosis as a key driver of stem cell loss when *Cph* regulation fails (Reviewer 2, specific point 7).

Strengthening experimental findings

- We have repeated assays with two independent *RNAi* lines for *Cph* and *Sff*, as well as using two *Cph* null alleles (Reviewer 2, general point 3, specific point 6).

- We have validated that *Cph* knockdown results in a decrease in *Cph* expression and protein level (Reviewer 2, specific point 4).
- Together with enhanced quantitative analyses, these controls provide robust support for our conclusions.

In this letter, figures associated with our responses are labeled as R-Fig, while those included in the revised manuscript are referred to as MS-Fig. We have provided a detailed point by point response to the reviewer's comments below.

Referee #1:

In this manuscript, Redhai et al. found that Cph expression was significantly increased in Notch inactivation-induced tumors by comparing single-cell sequencing results after Notch knockdown. Their results showed that Cph is essential for ISC maintenance and division, and when Cph is knocked down along with Notch knockdown, the Notch mutant tumor fails to grow. Chromatin profiling showed that Cph has direct binding to genes related to EEC differentiation, suggesting that Cph is involved in EEC differentiation. They also found that overexpression of Cph resulted in decreased expression of the Cph-YFP reporter, suggesting that overexpression of Cph has a repressive effect on its own transcription.

The large amount of single cell transcriptomic data in this manuscript constructively contributes to the study of Drosophila midgut homeostasis. It has significant reference value for Notch signaling in ISC maintenance and differentiation. The discovery of the Cph gene by single cell sequencing sheds new light on understanding the phenotypes caused by Notch mutations. Unfortunately, in this study, mutation of Cph resulted in arrest of ISC division and differentiation of ISCs into polyploid cells; and overexpression of Cph led to ISC apoptosis. Therefore, it is difficult to phenotypically clarify the role of Cph in the differentiation of ISCs into EECs. The authors require further experiments to support their conclusions.

We thank the reviewer for their positive feedback on our study, particularly with regard to our single-cell genomic data. We greatly appreciate their recognition of the strengths of our approach and their constructive suggestions for improvement.

We fully agree that a more comprehensive characterization of *Cph* under homeostatic conditions would enhance the depth and impact of our findings. In response to the reviewer's suggestions, we have undertaken a series of additional experiments aimed specifically at addressing their key concerns. These new analyses have allowed us to gain deeper insights into the function of *Cph* under baseline conditions and its regulation.

We believe that the results from these experiments substantially strengthen our conclusions and provide a more robust foundation for our interpretations. We have incorporated these findings into the revised manuscript, and we hope the reviewer will find that these additions enhance the quality and clarity of the study.

Major points:

1. On line 104-105, line 112-123, the authors wrote "When Notch signaling is high/When Notch signaling is low", citing an article published by the Spradling lab in 2007. In this literature, high/low Notch activation refers to the daughter cell of the ISC being in a high or low state while the ISC is in the state of Notch inactivation (Ohlstein, B. & Spradling, 2007). However, in this manuscript the authors found that high/low Notch signaling occurs in ISCs and that Notch knockdown results in increased expression of Sc and cpf in ISCs. Therefore, the authors should specifically write "When Notch signaling is high in ISCs/When Notch signaling is low in ISCs". The data in this manuscript are reminiscent of bidirectional Notch activation, in which EMC/EEPs expressing Dl activate Notch signaling in ISCs (Guo and Ohlstein 2015).

We thank the reviewer for highlighting this distinction. We have now revised our statement to refer to ISCs when mentioning high/low Notch signaling and have cited Guo and Ohlstein 2015 (lines 84-86)

2. Figure 1E is misleading. I thought the small black and white panels were the separate channels of the RGB images. I later realized that the author was showing different midguts with different antibody staining. The author should remake this figure to show the GFP signal, Pros and Tk (and AstC) staining in the same midgut.

We apologies for the lack for clarity. Unfortunately, we cannot co-stain AstC and Tk on the same midgut as both antibodies are derived from rabbit. Moreover, PH3 is also a rabbit antibody, which is why we have taken the approach to stain each of these antibodies separately.

To avoid confusion, have re-arranged the black and white panels (Fig. 1D) and have also stated in the figure legend that the immunostaining panels were obtained from separate fly midguts.

3. In line 252, the authors claim that Cpf-YFP is expressed in ISCs. However, in Figure 2F, the Cph-YFP signal cannot be distinguished in EBs or in ISCs. I noticed that there are pairs of Cph-YFP+ cells in the control image of Figure 2F, indicating that Cpf is expressed in both ISC and EB. The author should use NRE-lacZ; esg>RFP stock to distinguish Cpf expression in ISC or EB. Again, it is very clear that pairs of Cpf-GFP cells are present in Figure EV4.

Following the reviewer's suggestion, we used Su(H)-GBE-LacZ (NRE-LacZ), which specifically marks enteroblasts (EBs). As shown in R-Fig. 1B/MS-Fig. 2C, Cph^{YFP} (*Cph* tagged at its endogenous locus) signal is absent in NRE-$LacZ^{+}$ cells, indicating that *Cph* is not expressed in EBs. To confirm the identity of *Cph* expressing cells, we used the stem cell–specific marker Delta-LacZ, and showed that *Cph* is expressed ISCs (R-Fig. 1A/MS-Fig. 2B). We are unsure which pairs of Cph-YFP cells the reviewer is referring to but we have now separated individual channels to avoid confusion. Together with our existing data, we have now revised our text to state that *Cph* is expressed only in ISCs, EEPs and EEs (lines 233-235) and have annotated the panels to highlight Cph^{YFP} expressing cell types (R-Fig. 1/MS-Fig. 2B,C).

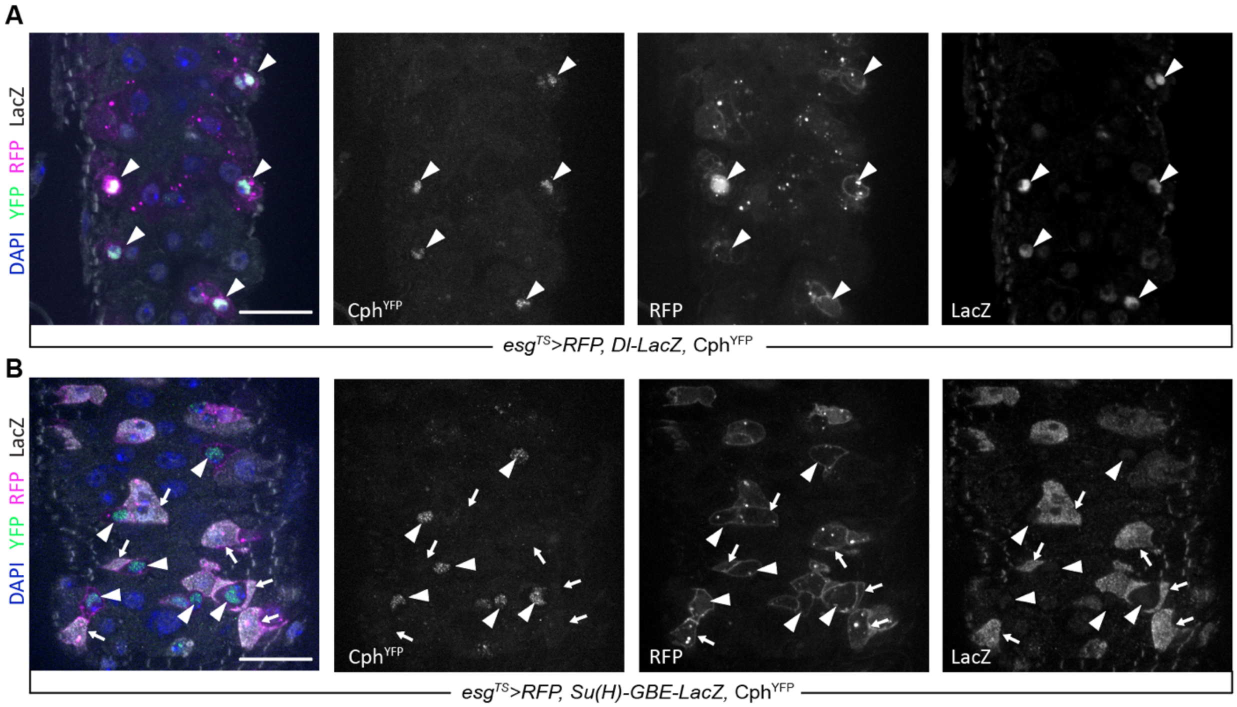

R-Fig.1 – **(A, B)** β-gal staining of midgut highlights that Cph is found specifically in Delta-LacZ^{+} ISCs (A, arrowheads) and not Su(H)-GBE-LacZ^{+} EBs (B, arrows). Confocal images taken from the posterior midgut. Scale bar for 100 μm.

4. Line 274, are the large cells in Figure 3B ECs? The author should stain Pdm1 to check the cell fate. And the author should use NRE-lacZ, the Notch reporter, to check Notch activation in the esgts>cpf RNAi midgut. Also, in Figure EV6A, the shape of the esgts>cpf RNAi GFP+ polyploid cells reminds me of the typical mTOR activation phenotype in ISCs (Amcheslavsky, Ito et al. 2011, Kapuria, Karpac et al. 2012). The genetic interaction between cpf and mTOR signaling should be considered to understand the molecular functions of cpf in ISCs during tissue homeostasis.

We have performed the following experiment as suggested by the reviewer:

1) We have stained the midgut with PDM1 antibody when *Cph* is silenced using the ReDDM system (*esg^{TS}, H2B-RFP*). Interestingly, the large *esg^{+}/H2B^{+}* cells that appear when *Cph* is silenced are positive for PDM1. However, unlike ECs which contain PDM1 throughout the nuclei during steady state, large *esg^{+}/H2B^{+} Cph^{RNAi}* expressing cells displayed intense foci of PDM1 staining in the nucleus (R-Fig.2A/MS-Fig. EV5E). We think this is a result of a disruption to the ISC differentiation program, which results in *esg^{+}/H2B^{+} Cph^{RNAi}* expressing cells becoming large, with large nuclei, but still expressing progenitor markers such as *esg*. We have now incorporated this data into MS-Fig. EV5E, lines 286-289.
2) We have analyzed the pattern of Su(H)-GBE-LacZ (NRE-LacZ) during homeostasis and when *Cph* is silenced in progenitor cells using *esg^{TS}*. As shown in R-Fig. 2B,C/ MS.Fig4 D,E, while NRE-LacZ marked a sub-population of *esg^{+}* cells during steady state, when *Cph* is silenced, NRE-LacZ expression is drastically reduced, indicating that progenitor cells are arrested at the ISC stage. These data are now discussed in lines 282-284.

3) We also investigated the interaction of *Cph* with mTOR signaling following the reviewer's suggestion. *Amcheslavsky, Ito et al. 2011, Kapuria, Karpac et al. 2012* demonstrated that overactivation of mTOR signaling in progenitor cells increases their size. The reviewer has suggested that this is reminiscent of the large *esg*$^{+}$ cells that we observe when *Cph* is silenced in progenitor cells, indicating that there may be an overactivation of mTOR. Therefore, we have blocked mTOR signaling using a dominant negative mutant of Tor (*Tor*^{TED}) when *Cph* is silenced in progenitor cells. This demonstrated that downregulating mTOR signaling by itself decreases the number of progenitor cells. Co-expression of *Tor*^{TED}+ *Cph*^{RNAi} did not rescue the depleted progenitor population that we observed when silencing *Cph* only (R-Fig. 2D). Since these experiments demonstrates that mTOR does not have a significant role in the *Cph*^{RNAi} -induced depletion of progenitor cells, we have not included it in our revised manuscript so that the paper remains streamlined and focused.

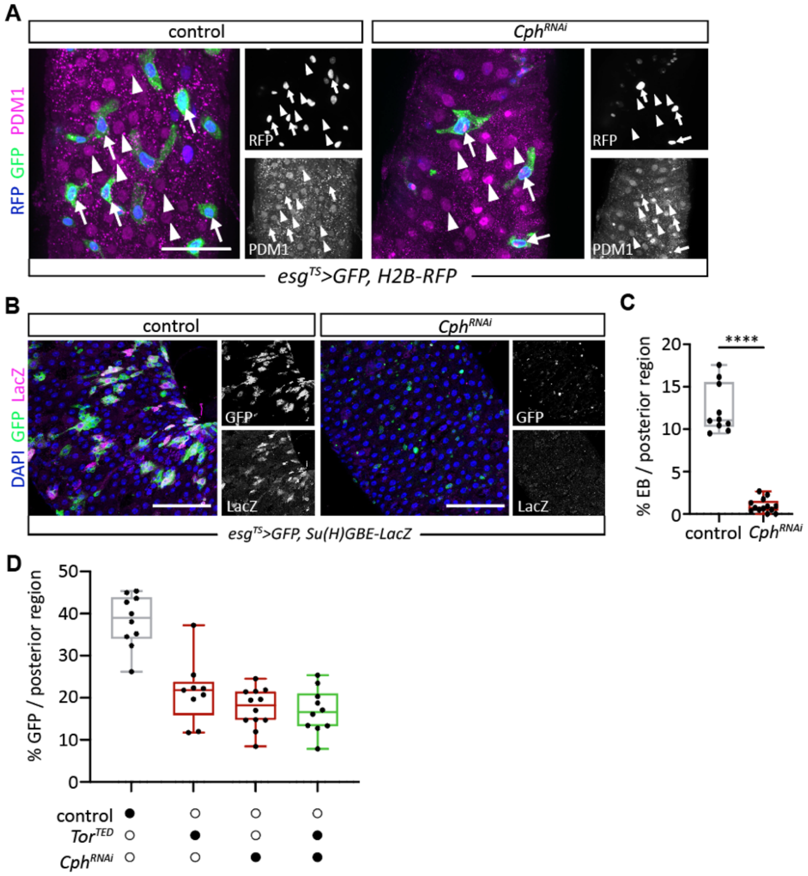

R-Fig.2 – **(A)** REDDM lineage tracing highlighted that *Cph* depletion causes some progenitor cells to become abnormally large and contain nuclear PDM1. Scale bar 100 µm. **(B)** β-gal staining of the midgut demonstrated that loss of Cph reduces the number of Su(H)-GBE^{+} EBs, indicating that cell cycle is arrested at the ISC stage. Scale bar 100 µm. **(C)** Quantification of the number of Su(H)-GBE^{+} EBs in the posterior midgut. Each dot represents a biological replicate. **(D)** Progenitor-specific expression of *Tor*^{TED} (inhibition of mTOR signaling) or *Cph*^{RNAi} reduce the number of progenitor cells in the posterior midgut. Blocking mTOR signaling (*Tor*^{TED}) in *Cph*^{RNAi} expressing progenitor cells does not modulate the number of progenitor cells compared to *Cph*^{RNAi} only condition. Mann-Whitney test was used to calculate the p value for (C).

5. line 287-290, "......suggesting that Cph is involved in maintaining the progenitor and EE populations (Figure 3C-E)". However, the reduction in the number of EECs does not prove that cpf is necessary for EEC differentiation. Since knockdown of cpf results in the loss of ISC, the absence of ISC naturally results in fewer EECs. Not only is the number of EECs reduced, but the number of newly generated ECs is also reduced. In my opinion, the cpf RNAi phenotype is mainly related to ISC division and maintenance. The authors need to demonstrate with more genetic experiments that knockdown of cpf leads to reduced differentiation of EEC. For example, counting the percentage of ISC daughter cells that differentiate into EEC and the percentage that differentiate into EB-EC in the esgts F/O or ReDDM >Cph RNAi lineage.

We fully agree with the reviewer that a more detailed assessment of Cph's role in EE differentiation will strengthen our conclusions. To more directly assess the role of *Cph* in EE differentiation, we performed additional genetic experiments:

First, we generated MARCM clones using two independent *Cph* null alleles (Cph^{A} and Cph^{B32}), allowing us to study mutant clones in a wild-type background. Under control conditions, medium-sized clones (containing 6–15 cells) typically had several Armadillo-positive diploid progenitor cells, multiple polyploid ECs, and at least one Pros^{+} EE (R-Fig. 3A-C/MS-Fig. 4I-K). In contrast, Cph^{A} and Cph^{B32} clones were consistently smaller (2-5 cells per clone) and showed a marked depletion of Pros^{+} EE cells, despite containing some ECs and progenitors (R-Fig. 3A-C/MS-Fig. 4I-K). These results suggest that *Cph* is required not only for clonal expansion but also for EE cell differentiation.

To complement this finding, and as the reviewer suggested, we employed the $esg^{F/O}$ lineage tracing system to assess epithelial turnover. Upon *Cph* knockdown, we observed a pronounced reduction in GFP-positive lineage-traced cells, consistent with impaired in tissue turnover (R-Fig. 3D,E/ MS-Fig. EV5C,D). In the context of low notch signaling, *Cph* is required to generate enteroendocrine cells and for ISC proliferation (R-Fig. 3D,E/ MS-Fig. EV5C,D). This supports our conclusion that *Cph* is required for ISC maintenance, proliferation and differentiation.

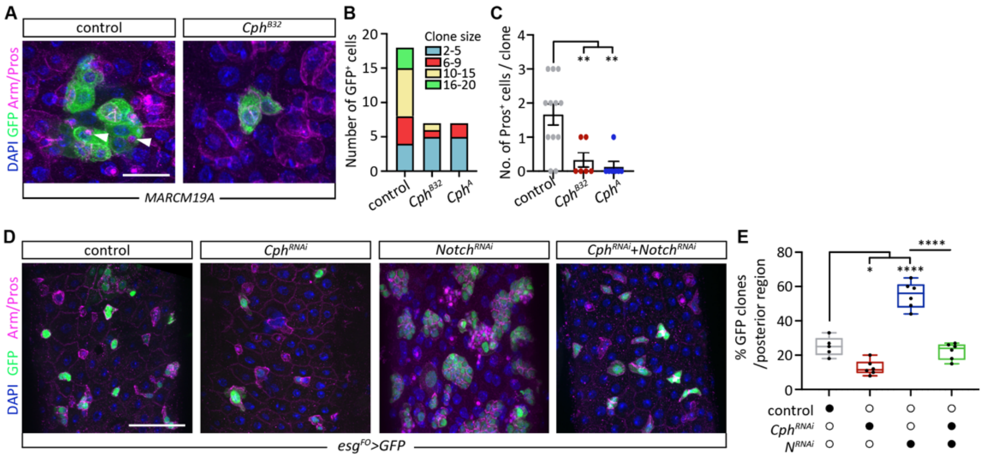

R-Fig.3 – **(A)** MARCM clones of Cph^{B32} null mutant are markedly smaller than control clones and contain few, if any, EEs. Scale bar 50 µm. **(B)** Quantification of the number of cells per clone. **(C)** Quantification of the number of Pros^{+} EEs in GFP^{+} clones. **(D)** Confocal images of Flip out clones

within the posterior midgut under different genetic conditions. Scale bar 100 µm. **(E)** Quantification of the number of GFP clones within the posterior midgut. One-way ANOVA test with Tukey post hoc comparison was used for statistical analysis (C,D).

6. Figure 3C is misleading. The Astc and Tk staining panels are not in the same gut as the first row of esgts>GFP panels. The author should show the GFP and AstC/Tk staining in the same midgut regions. The PH3 staining is not necessary, so the author can put the AstC/Tk staining in the red channel.

Please see our response to point 2 with regards to co-staining AstC and TK. We think that having pH3 is important as it indicates that *Cph* is involved in regulating mitosis under *Notch* depleted condition, which is a novel finding. Due to the space restrictions within this figure, it was difficult to re-arrange panels, however we have now separated the composite images from the black and white panels with a thick black line to indicate they are not from the same midgut. Additionally, we have now stated in the legend of figure 3 that the images were obtained from different midguts to avoid confusion.

7. In lines 294-296 the authors claim that cpf RNAi reversed EEC differentiation. However, cpf RNAi inhibited the proliferative capacity of Notch RNAi ISCs, so there was no production of new EECs. Therefore, AstC and Tk were stained for residual EECs in Figure 3C. If the authors insist that cpf RNAi reversed Notch RNAi EEC differentiation, then the authors should perform staining for AstC and Tk in ReDDM>cpf RNAi+Notch RNAi lineage tracing experiments to demonstrate that the cells staining for AstC and Tk are H2B-RFP+ cells.

We thank the reviewer for this thoughtful comment and apologize for the confusion in our original text. Our data indicates that *Cph* is required for both the proliferation and EE differentiation phenotypes induced by *Notch* knockdown in progenitor cells. To clarify, in the Cph^{RNAi} + $Notch^{RNAi}$ condition, reduced Pros staining likely reflects a suppression of EE differentiation due to impaired proliferation and lineage progression, rather than a reversal of differentiation in pre-existing EE cells.

We have revised the manuscript text (lines 267-270) to read: "conditional knockdown of both *Cph* and *Notch* decreased the number of progenitors that were mitotically active and restored both $AstC^+$ EEs and Tk^+ EE numbers back to wild type levels "

8. Figure 4A analyses the expression of cpf in the case of Notch knockdown. They show that Cpf is expressed in Pros+ Sc+ cells. However, Figure 2F and 4B shows that Cpf is expressed in most ISCs and EBs that do not express Sc, suggesting that Cpf expression is not dependent on Sc. Furthermore, Figure 4D does not show that Sc binds to the TSS site of Cph, and the binding site shown is in the exon of Cph. Further experiments are required to determine whether Sc binds directly to Cph DNA to regulate Cph transcription. For example, knock down Sc in the context of Notch knockdown and observe the expression of Cpf-YFP.

We appreciate the suggestion to better understand the role of *scute* (*sc*) in regulating *Cph* expression. In response, we have conducted a number of additional experiments and clarified our analyses in the revised manuscript.

1. We now show that overexpression of *sc* using the *esg*^{TS} system leads to increased levels of Cph^{YFP} within progenitor cells (R-Fig.4/ MS-Fig. 5C,D). These data demonstrate that *sc* overexpression is sufficient to elevate Cph protein levels in progenitor cells.

2. Consistent with our scRNA-seq results, we show that knockdown of Notch leads to increased Cph^{YFP} intensity in progenitor cells (R-Fig.4/ MS-Fig. 5D). Importantly, when *sc* is simultaneously silenced in the context of *Notch*^{RNAi}, Cph^{YFP} levels are significantly reduced compared to *Notch*^{RNAi} alone (R-Fig.4/ MS-Fig. 5D). This data demonstrate that *sc* is required—at least in part—for the *Notch*^{RNAi} -induced upregulation of *Cph*.

3. We have now placed asterisks on the scute Chip-seq plot to annotate the location of transcription start site (TSS). As shown in MS-Fig. 5E, significant binding peaks are detected at some of these TSS sites, supporting our conclusion that *sc* directly binds to the *Cph* locus.

We would like to refer the reviewer to our response to point 2, where we show that *Cph* is expressed in ISCs and not EBs. Together with our additional experiments, we now show that *sc* is sufficient to promote *Cph* expression and is required for *Notch*^{RNAi}-induced increase in Cph^{YFP}. However, we acknowledge that *sc* is transiently expressed in ISCs, whereas *Cph* is present in all ISCs, including those lacking detectable *sc*. Therefore, we think that additional regulators likely contribute to *Cph* expression and finding these factors is important in future studies. We have included this in the discussion, 563-567.

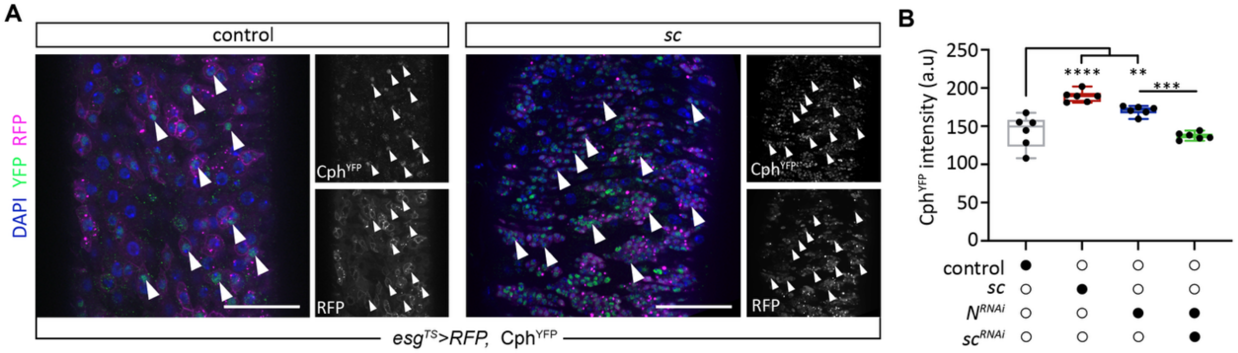

R-Fig. 4 – **(A)** Confocal images of the posterior midgut, highlighting Cph^{YFP} in RFP^{+} progenitor cells. Scale bar 100 µm. **(B)** quantification of Cph^{YFP} in progenitor cells under different genetic backgrounds. Notice that overexpression of *sc* increases Cph^{YFP} intensity in progenitor cells, while knockdown of *sc* in *Notch*^{RNAi} expressing progenitor cells restores Cph^{YFP} intensity in progenitor cells back to wild type levels. One-way ANOVA test with Tukey post hoc comparison was used for statistical analysis.

9. Figure 4E-G does not demonstrate that cpf is downstream of sc. Knockdown of any of the housekeeping genes or knockdown of cell cycle related genes may result in fewer cell proliferation and fewer AstC+ cells from Sc overexpression. More rigorous experiments are needed to support the conclusions drawn by the authors in lines 334-336. For example, using Crispr-Cas9 to mutate

the E-box binding motif GCAGGTGT within the Cph locus to test for reduced transcript levels in cpf.

We agree that Figure 4E-G alone does not establish *Cph* as a direct downstream target of *sc*. However, our conclusion is supported by a body of complementary evidence throughout our revised manuscript, not solely based on this figure. Specifically, we show:

- **Direct binding**: ChIP-seq shows that Scute binds to the TSS of *Cph*, supporting the idea that *Cph* is a direct transcriptional target (MS-Fig. 5E).
- **Regulation**: Overexpression of Scute increases *Cph* transcript and protein levels, demonstrating functional regulation (R-Fig 4/MS-Fig. 5D, EV. 6C).
- **Necessity:** Silencing *sc* in the context of $Notch^{RNAi}$ reduces Cph-YFP levels compared to $Notch^{RNAi}$ only, demonstrating that *sc* is required for $Notch^{RNAi}$-induced upregulation of Cph^{YFP} (R-Fig 4/MS-Fig. 5C,D).
- **Co-expression**: The fact that Scute and Cph are present in the same cell type strengthens the biological plausibility of this regulatory relationship (i.e., Scute is in the right place to activate *Cph*).
- **Functional dependency**: we show that *Cph* is required for Scute-induced progenitor proliferation and EE differentiation.

Together, the combination of binding evidence, regulatory expression, cellular colocalization, and genetic epistasis provide a multi-layered body of evidence that *sc* regulates *Cph* to drive proliferation and EE differentiation. To avoid overinterpretation, we have revised our text to state "In conclusion, our data identifies *Cph* as a direct transcriptional target of *sc* and a key mediator of *sc*-induced proliferation and differentiation in intestinal stem cells, see lines 351-353.

We appreciate the reviewer's suggestion to mutate the E-box binding motif in *Cph* using CRISPR-Cas9 to test whether these sites are required for Scute to drive *Cph* expression. While we agree this would be informative some technical factors prevented us from doing so. For example, the *Cph* locus contains multiple E-box motifs, some of which lack appropriate PAM sites, making targeted mutagenesis by CRISPR mutagenesis challenging in this context. In addition, even if we mutated these sites, Scute may also regulate *Cph* from distant enhancer elements. As a result, we now omit mentioning the E-box motif in our revised manuscript, and focus more on the functional *in vivo* data that we have which demonstrate that scute promotes *Cph* expression, and is required for the $Notch^{RNAi}$-induce increase in *Cph*. We hope that the new data stated above and in our revised manuscript, now sufficiently support our conclusions.

10. In Figure 5B and lines 355-363, the author observed that CphRNAi+NotchRNAi leads to an increase in the number of ECs. However, the conclusion that cpf knockdown leads to a shift in the direction of ISC differentiation towards EB-ECs is not appropriate. According to the literature published by Edgar's group (Patel, P.H., Dutta, D. & Edgar, B.A., 2015), Notch RNAi leads to tumors that squeeze the neighboring EC, resulting in the loss of ECs, which in turn promotes further tumor growth. cpf RNAi leads to a drastic reduction in the cell's ability to proliferate (Figure 3D and 4F), therefore Notch RNAi is unable to form tumors that invade the basement membrane occupied by ECs, resulting in no reduction in the number of EC cells. To support the conclusion in line 362-363, the author should perform lineage tracing experiments. Make Notch

mutant/RNAi MARCM clones with cpf RNAi expressed inside the clone cells to show that differentiation is restored as in the WT control MARCM clones.

We thank the reviewer for highlighting the Patel *et al.* (2015) study and for their thoughtful comments regarding the interpretation of our findings in Figure 5B.

We would like to clarify that our conclusions were not intended to give the impression that *Cph* knockdown leads to a shift in ISC differentiation toward the EB-EC lineage. As stated in the original manuscript, lines 354–358:

"When progenitor cells expressed *Cph*RNAi + *Notch*RNAi, the number of ISCs, EEPs, and EEs decreased in comparison to *Notch* silencing alone (Fig. 5B). Furthermore, while the number of ECs was lower when *Notch* was depleted, co-silencing *Cph*RNAi + *Notch*RNAi resulted in a significant increase in ECs, although this was not accompanied by an increase in the EB population."

In line 358–359 of the original manuscript:

"These data suggest that Cph is required when Notch signalling is low to maintain different intestinal cell types."

To avoid confusion and overinterpretation of our results, we have now re-written this section as follows (lines 374-379):

"We observed a notable increase in the number of ISCs, EEPs, EEs and a decrease in EBs in the *Notch*^{RNAi} condition, consistent with observations made using CRISPR-Cas9. Co-expression of *Cph*^{RNAi} and *Notch*^{RNAi} in progenitors reduced the numbers of ISCs, EEPs, and EEs, while driving an increase in ECs without a corresponding rise in EBs compared to *Notch*^{RNAi} only condition. This suggests that *Cph* is required for *Notch*^{RNA}-induced changes to cell type composition.

Unfortunately, we were not able to generate MARCM fly lines that allowed us to perform double perturbations between *Cph* and *Notch*. However, as the reviewer suggested to use lineage tracing approaches, we have used two alternative lineage tracing systems that support our conclusions. Using the ReDDM system, we see that progenitor-specific silencing of *Notch* increases the number of GFP/RFP^{+} progenitor cells, while increasing RFP^{+} EEs (MS-Fig. 3A,B). Co-silencing *Cph* and *Notch* restored the number of progenitors and EE cells back to WT levels EEs (MS-Fig. 3A,B). Similarly using the *esg*$^{F/O}$ system, we showed that *Cph* is required for *Notch*^{RNAi}-induced increase in progenitor and EE cells (MS-Fig. EV5C,D).

11. For the transcriptional analysis of NotchRNAi and CphRNAi+NotchRNAi mdiguts (Fig. 5C-F), a key argument is: Is the alteration of the Notch RNAi ISC transcriptome by cpf RNAi direct or indirect? Due to the squeezing effect of Notch RNAi tumors on ECs and the proliferative effect of EC on Notch RNAi tumors via upd3 and EGFR ligands, if cpf RNAi affects the proliferation of Notch RNAi tumors, it will indirectly affect the expression of a number of genes. The ideal analysis (not mandatory) would be to compare single cell sequencing of CphRNAi + NotchRNAi with sequencing of single cells prior to tumor generation by Notch RNAi.

We agree that dissecting apart the cell autonomous vs non-autonomous contribution of *Cph* during proliferation is important. Unfortunately, we do don’t have the timepoint that the reviewer requested. However, we have performed additional analyses on our scRNA-seq dataset to investigate their question.

Specifically, we found that Upd3 along with genes involved in the regulation of JAK/STAT signaling as well as EGFR signaling components were significantly upregulated in the EC population when *Notch* was silenced in progenitor cells, consistent with the Patel *et al.* (2015) study (R-Fig. 5/MS-Fig. EV7D,E). Crucially, in co-perturbed condition (*Cph*^{RNAi}+*Notch*^{RNAi}), expression of JAK-STAT and EGFR signaling components remained elevated in ECs, despite a significant decrease in progenitor proliferation (R-Fig. 5/MS-Fig. EV7D,E). This suggests that *Cph* functions autonomously within ISCs to restrain excessive proliferation in low Notch signaling conditions, even though cytokine signaling in neighboring ECs remained elevated.

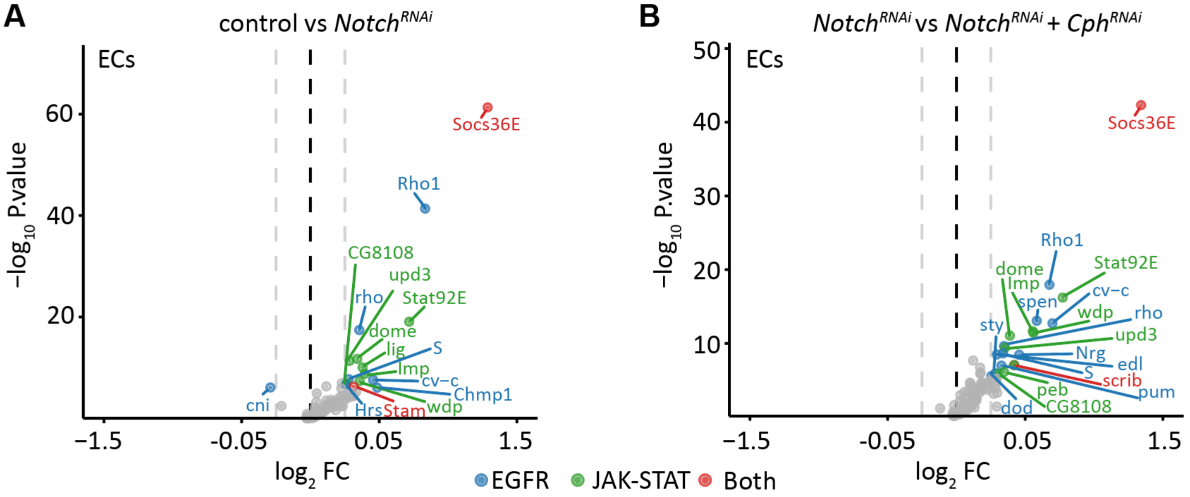

R-Fig.5 – **(A, B)** Volcano plot of differential gene expression in the EC population in either control vs *Notch*^{RNAi} (A) or *Notch*^{RNAi} vs *Cph*^{RNAi}+*Notch*^{RNAi} (B). Genes belonging to the EGFR, JAK-STAT or both signaling pathways are colour coded. Note that many genes that are upregulated for the JAK-STAT and EGFR signaling pathways in control vs *Notch*^{RNAi} remain upregulated in *Notch*^{RNAi} vs *Cph*^{RNAi}+*Notch*^{RNAi}.

12. In Figure 6F, the GFP signal is abnormal in sff RNAi+Notch RNAi. To me, it looks like sff knockdown resulted in ISC cell death. I am curious about the phenotype of ssf knockdown. Since the authors claimed that sff is not expressed in ISCs during homeostasis, then esgts and esgF/O>sff RNAi should not induce ISC loss phenotype.

As suggested by the reviewer, we examined the effect of *Sff* knockdown under homeostatic conditions using both *esg*^{TS} and *esg*$^{F/O}$ drivers. In both experimental conditions, we observed no significant differences between *Sff* knockdown and control midguts—indicating that loss of *Sff* does not alter progenitor cell numbers (*esg*^{TS} experiments) or tissue turnover (*esg*$^{F/O}$ experiments), see MS-Fig.7F-I/ Fig. EV8E-H. These results are in line with *Sff* not being expressed during steady

state. We have also included improved representative images of the *Sff*^{RNAi} + *Notch*^{RNAi} phenotype (MS-Fig. 7F). We have now discussed these results in our revised manuscript, lines 460-462.

Referee #2:

Overall Assessment
The bioinformatics analysis in this study is thorough and robust. However, the subsequent cell biological investigations aimed at elucidating causality are notably weak, often lacking quantitative rigor and comprehensive exploration.

We thank the reviewer for recognizing the robustness of our scRNA-seq dataset. To improve our quantitative rigor and conclusions, we have we used two independent *RNAi* lines for *Cph* and *Sff*. Additionally, we have used two *Cph* null alleles, which support our findings that *Cph* is involved in stem cell maintenance and enteroendocrine cell differentiation. We have also thoroughly investigated the role of *Cph* during steady-state conditions, using MARCM clones and cell type specific perturbations and studied its impact on physiology (e.g lifespan). Moreover, using various reporters, we now show that autoregulation of *Cph* is crucial for preventing autophagy and cell death, and rescue experiments demonstrated that apoptosis is a crucial driver of stem cell loss when *Cph* is expressed beyond physiological levels. We believe these additions have substantially strengthened our conclusions and further improved our manuscript.

General Comments
This study exhibits three critical weaknesses that should be experimentally addressed:

1. The authors heavily rely on Notch inhibition as a contextual framework, which is both artificial and non-physiological. A fundamental, more biologically relevant question is the role of Cph under normal physiological conditions. By extensively employing an artificial Notch inhibition model and then extrapolating their findings to physiological conditions, the authors risk drawing overly general and potentially misleading conclusions. It is imperative that they clearly delineate the artificial context from the physiological one. Moreover, they should explore the function of Cph under normal, homeostatic conditions to enhance the biological relevance of their findings.

We thank the reviewer for their valuable suggestion to better characterize the molecular function of *Cph* under homeostatic conditions. In response, we have performed a number of additional experiments (see below) that further support our conclusion that *Cph* is critical for ISC self-renewal, proliferation and EE differentiation. These new data, along with results from our original manuscript, collectively build a more comprehensive understanding of *Cph* function:

1. We generated MARCM clones with two *Cph* null alleles (Cph^{A} and Cph^{B32}), which revealed that *Cph* is required for clonal expansion and EE generation (R-Fig. 3A-C/MS-Fig. 4I-K).
2. Using the $esg^{F/O}$ system, we found that loss of *Cph* significantly reduces epithelial turnover in the adult midgut (R-Fig. 3D,E / MS-Fig. EV5C,D).
3. Silencing *Cph* in progenitor cells under homeostatic condition predominantly decreases the number of AstC+ EEs, highlighting its role in EE fate specification (MS-Fig. 4A,C).
4. Through cell type–specific knockdown approaches, we demonstrate that *Cph* plays a central role in ISCs and not EBs to regulate proliferation and EE differentiation (MS-Fig. 4F,G/ EV5F).

5. Lifespan assays showed that silencing *Cph* using the ISC-specific driver leads to a reduction in longevity, suggesting a broader physiological role for *Cph* (MS-Fig. 4H).
6. Overexpression of *Cph* leads to its own downregulation (suggesting an autoregulatory feedback mechanism) and induces expression of genes associated with autophagy and cell death, resulting in a reduced number of progenitor cells (MS-Fig. 8B-D/ EV9C,D).
7. Consistent with the findings above, *Cph* overexpression increases the number of $Lamp1^{+}$ lysosomes and $Atg8a^{+}$ autophagosomes, indicating a role in promoting autophagy (MS-Fig. 8E,F/ EV9E,F).
8. Using an apoptotic sensor, we show that *Cph* overexpression increases cell death in progenitor cells, consistent with the upregulation of cell death associated genes (MS-Fig. 8G/ EV. 9G,H). Importantly, blocking apoptosis in *Cph* overexpression progenitor cells partially rescues the number of progenitor cells, suggesting that the apoptotic pathway is a key driver of intestinal stem cell loss when *Cph* is overexpressed (MS-Fig. 8H/ EV9L).

Taken together, these experiments now provide a robust dataset supporting a key role for *Cph* in maintaining intestinal homeostasis and EE differentiation. Specifically, we show that *Cph* is required for ISC maintenance, proliferation, and EE differentiation, and its autoregulation guards ISCs against autophagy and cell death. We believe these findings significantly strengthen the understanding of *Cph* function in the adult midgut during steady state.

2. The simultaneous regulation of proliferation and differentiation by Cph is insufficiently clarified. Are these two processes interdependent? Investigating potential crosstalk between these pathways could yield deeper biological insights.

We thank the reviewer for this thoughtful comment regarding the relationship between proliferation and differentiation in the context of *Cph* function. We have included new data that directly addresses their question, including:

1) We now include MARCM clone analysis using two *Cph* null alleles (Cph^{A} and Cph^{B32}), which demonstrate that *Cph* is required for both clonal expansion and EE cell generation (R-Fig.3A-C/MS-Fig.4I-K). Loss of *Cph* leads to smaller clones and a near absent number of Pros+ EE cells within clones, indicating that proliferation and differentiation are impaired in *Cph*-deficient clones (R-Fig.3A-C/MS-Fig.4I-K).
2) To understand if proliferation and differentiation are interdependent, we performed epistasis experiments using a constitutively active *Ras* allele (Ras^{V12}). Driving Ras^{V12} in midgut progenitors markedly elevated the number of mitotic cells while concomitantly decreasing $Pros^{+}$ enteroendocrine cells (R-Fig.6A-C/ MS-Fig. EV5G-I). When Ras^{V12} was co-expressed with Cph^{RNAi} in progenitors, progenitor abundance and mitotic activity increased compared to Cph^{RNAi} only conditions, yet EE differentiation was not restored (R-Fig.6A-C/ MS-Fig. EV5G-I). Together, these results show that a proliferative stimulation alone cannot rescue EE fate in the absence of *Cph*, indicating that, in this context, proliferation and differentiation are separable.

We believe that these new data, together with the existing NanoDam dataset, demonstrate that *Cph* regulates both proliferation and differentiation, but that these processes are likely independent of one another and occur sequentially.

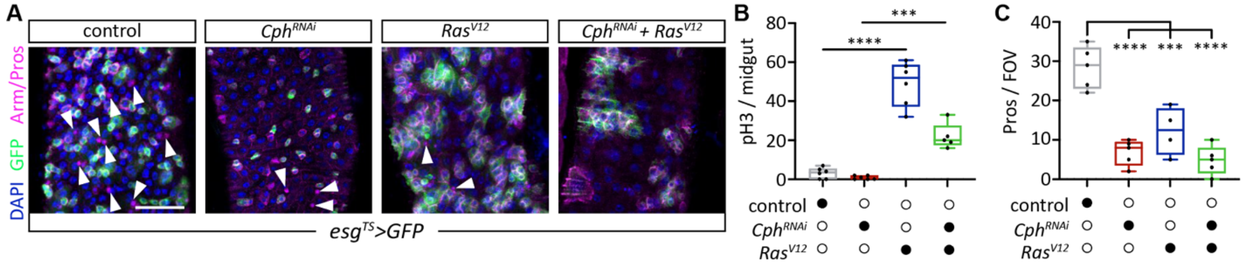

R-Fig.6 – **(A)** Confocal images of the posterior midgut under different genetic conditions. Arrowheads point toward Pros+ EEs. Scale bar 100 µm. **(B, C)** Quantification of the number of mitotically active cells in the midgut (B) or Pros+ EE in the field of view (C). Notice that expression of Ras^{V12} increases proliferation in Cph^{RNAi} expression progenitor cells (B) but does not increase the number of Pros+ EEs (C). One-way ANOVA test with Tukey post hoc comparison was used for statistical analysis of (B,C).

3. In the experimental parts, the data often lack quantitative rigor and thorough investigation. Relying solely on single RNAi experiments is problematic. The authors must employ multiple RNAi constructs (for both Cph and sff) to validate their findings. The authors also must specify the region of the gut analyzed in their data, as it significantly influences interpretation. Although the study focuses on female flies, recent evidence highlights the influence of both sex and genetic background on intestinal stem cell (ISC) proliferation. Including data from males and varied genetic backgrounds is essential for robust conclusions.

We thank the reviewer for raising important concerns regarding experimental rigor, validation, and biological variables such as sex and genetic background.

In response, we have now included two independent *RNAi* lines for both *Cph* (MS-Fig. 4B,C) and *Sff* (MS-Fig. 7F-I/ EV8E-G) for key experiments. Additionally, we utilized two independent *Cph* null alleles (Cph^{A} and Cph^{B32}) to further confirm that *Cph* is required for enteroendocrine (EE) generation and ISC proliferation (R-Fig.3A-C/MS-Fig.4I-K).

We also apologize for the omission of the specific gut regions analyzed. All quantifications—except for phospho-histone H3 (pH3) staining—were performed in the posterior midgut (regions R4/R5). pH3 analysis was conducted across the entire midgut to capture the full range of mitotic activity. In the revised manuscript, we have now explicitly stated the gut region analyzed for each dataset in the figure legends to ensure clarity.

The reviewer raises an important point about sex-specific differences in ISC behavior. It is well-established that ISCs in females are more proliferative than those in males. As noted in the literature (PMID: 26887495), knockdown of *Notch* leads to excessive ISC proliferation in females, but not in males, unless specific genetic manipulations are used to feminize the male gut. For this reason, we used females for all our experiments. In our revised manuscript, we now include data showing that *Cph* is expressed in progenitor cells of both male and female flies (R-Fig.7/ MS-Fig. EV4H). However, due to the limited proliferative response in males, we believe that repeating our experiments in male flies would not be informative for addressing the specific mechanisms we

aimed to investigate and would instead shift the focus toward sex-specific differences, which is beyond the scope of this work

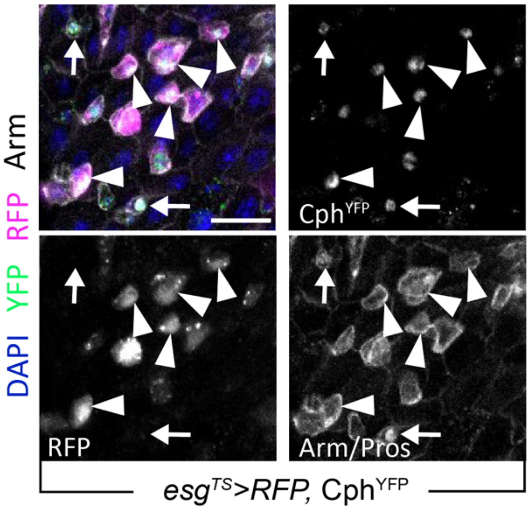

R-Fig. 7 - Cph^{YFP} is found in *esg>RFP*$^{+}$ progenitor cells (arrowheads) and Pros+ enteroendocrine cells (arrows) within the male midgut. Scale bar 20 µm.

Regarding genetic background, we agree that this is an important variable that can influence phenotype penetrance. As a result, to demonstrate the robustness of our phenotypes, we silenced *Cph* in either a w/yv background (*Cph RNAi*#1) or a w/yw background (*Cph RNAi* #2), both of which led to a similar reduction in progenitor cell proliferation and generation of EEs (MS-Fig. 4B,C). This demonstrates that the observed *Cph* phenotype is not driven by differences in genetic background.

Specific Comments

1) Figure 1:
The authors show that Notch inhibition alters the balance of type 1 and type 2 enteroendocrine (EE) cells while leaving class 3 unaffected. Why, then, does this manipulation affect the total EE cell number? This inconsistency is confusing and warrants clarification.

Under homeostatic conditions, ISCs generate EE subtypes, predominantly *AstC*+ (class I) and *Tk*+ (class II) cells, with class III EEs being less abundant. Upon *Notch* depletion, two distinct effects are observed:

1. A shift in subtype identity: EE differentiation becomes biased toward *AstC*+ (class I) cells, with a marked reduction in *Tk*+ (class II) cells.
2. An overall increase in EE number: Loss of Notch drives excessive ISC-to-EE differentiation, leading to a net expansion of the EE population.

Thus, the change in total EE number is not solely due to altered subtype balance, but also reflects an increase in the rate of EE production from ISCs. This effect is consistent with previously

published data showing that Notch inhibition promotes EE hyperplasia (e.g., Micchelli and Perrimon, 2006; Ohlstein and Spradling, 2007). We have now clarified this point in the revised manuscript (lines 157-165).

2) EV2b: The Sanger sequence should display several patterns of CRISPR-induced mutations, not just one. Is this data derived from a single cell?

The original Sanger sequencing was performed on cDNA derived from our scRNA-seq experiment, which was based on a pool of 20 midguts. In the initial analysis, we sequenced only one of the two sgRNA target sites (sgRNA2). We have now sequenced the second site as well, which clearly shows stronger mutagenesis (sgRNA1), as indicated by the loss of high-quality sequencing traces downstream of the sgRNA1 site (R-Fig.8/MS-Fig. EV2B)

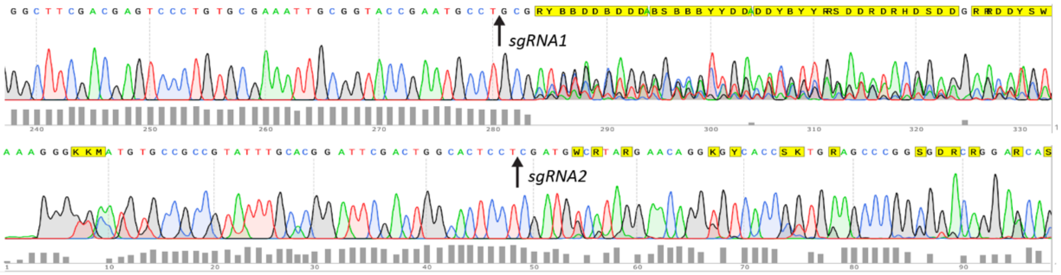

R-Fig.8 – Sanger sequencing traces for both Notch sgRNA target sites (see *Sanger Sequencing* section in Methods.

3) Figure 2F: The expression of Cph in Prospero-positive cells is challenging to appreciate. Improved presentation or clarification is needed.

To improve clarity, we have updated MS-Fig.2D to include separate grayscale panels for Cph and Pros, which better highlight their expression patterns. We also added arrows to indicate Pros^{+} EE cells and arrowheads to mark progenitor cells, making it easier to distinguish co-localization of Cph with Pros.

In addition, we would like to draw the reviewer's attention to MS-Fig.EV4G, where Cph^{YFP} is co-expressed with Pros^{+} RFP-labeled cells, further supporting the presence of Cph in Pros^{+} EE cells.

4) Figures 3B and 3C: These data require quantification. Additionally, the reduction in progenitor cell numbers shown in Figure EV6 could result from decreased proliferation or increased differentiation rather than cell death. Experimental clarification is necessary. The authors must employ multiple RNAi or gRNA constructs for Cph knockdown. Describing the effect of Cph RNAi under conditions without Notch inhibition would provide more biologically meaningful insights.

In our original manuscript we performed quantitative analysis of Figure 3C and displayed mitotic divisions (pH3) and the number of EEs positive for AstC and Tk are quantified in Figure 3D and E, respectively. In our revised manuscript, we have now quantified Figure 3A. Specifically, we

have quantified the number of RFP^+ only cells which is an indicator of epithelial turnover rate in the midgut, and is now displayed in Figure 3B.

We would like to clarify that in Figure EV6, we do not conclude that the reduction in the number of progenitor cells is due to cell death. In our original manuscript, we demonstrate that *Cph* is required to maintain ISC proliferation and maintenance. Since Cph binds to genes involved in the cell cycle, we think that Cph directly impacts proliferation and maintenance. We have now performed MARCM clones using two different *Cph* null alleles (Cph^A and Cph^{B32}). These experiments demonstrated that the clone size of Cph^A and Cph^{B32} are smaller, containing fewer cells per clone compared to control conditions (R-Fig. 3A-C/MS-Fig. 4I-K). This indicates that *Cph* is required for clonal growth, ruling out that an increase in differentiation is the reason for the lower number of progenitor cells. Importantly, although fewer in number, we still observe large polyploid ECs, but hardly any EEs in *Cph* mutant clones, demonstrating that *Cph* is required for EE differentiation (R-Fig. 3A-C/MS-Fig. 4I-K).

We have also used two independent *RNAi* lines to demonstrate that *Cph* is required for maintaining progenitor cell numbers and generation of EEs (MS-Fig. 4B,C). Additionally, we have validated that the *RNAi* constructs that we used for *Cph* results in a decrease in Cph^{YFP} intensity within progenitor cells, demonstrating the effectiveness of *Cph* knockdown (R-Fig. 9/MS-Fig. EV5A,B).

With regards to the function of *Cph* during homeostasis, please see our response to the reviewer's point 1.

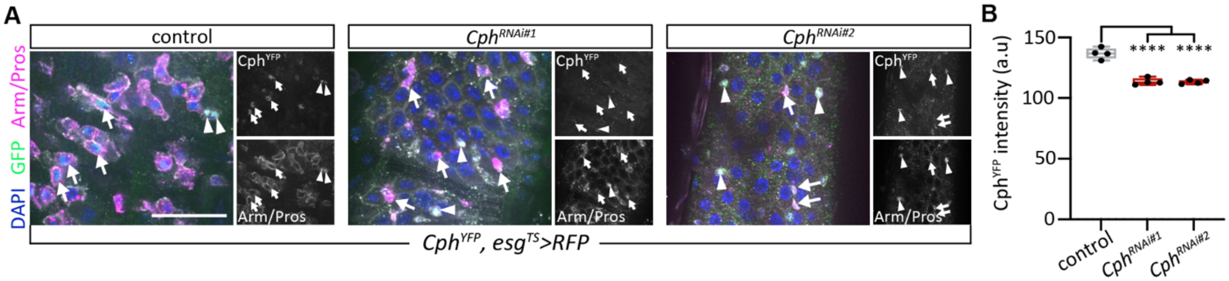

R-Fig. 9 - **(A)** Silencing *Cph* in progenitor cells decreases the intensity of Cph^{YFP} in progneior cells (arrows) and not in EE (arrowheads). Scale bar 50 µm. **(B)** Quantification of Cph^{YFP} in RFP^+ progenitor cells. Each dot represents a biological replicate. One-way ANOVA test with Tukey post hoc comparison was used for statistical analysis.

4) Figures 4C-D: The figure itself should be explicitly stated that this represents a re-analysis of previously published data. Relying on existing RNA-seq datasets to infer Scute overexpression's impact on Cph expression, rather than directly demonstrating this, is an unusual choice. Ideally, the authors should validate this finding using their NanoDam system, gel shift assays, or other direct approaches.

We have now moved this data to EV6C. As suggested by the reviewer, we have stated in the figure legend of Fig.EV6 that panel EV6C is a re-analysis of previously published RNA-seq data (with the appropriate citation included).

We have also performed additional experiments to directly assess the relationship between *sc* and *Cph*. Specifically, we now show that overexpression of *sc* in progenitor cells increases the levels of Cph^{YFP}, demonstrating that *sc* is sufficient to increase Cph protein levels (R-Fig. 10A,B/MS-Fig. 5C,D). Additionally, we show that *sc* is required for the increase in Cph^{YFP} expression observed upon *Notch* knockdown, as co-silencing *sc* together with *Notch* reduced the level of Cph^{YFP} compared to *Notch*^{RNAi} only (R-Fig. 10A,B/MS-Fig. 5C,D). Moreover, in Figure MS-Fig. 5E, Chip-seq data shows that scute binds directly to the *Cph* locus, specifically within transcriptional start sites.

Together, these new data establish both the sufficiency and requirement of *sc* in regulating *Cph* expression and strengthen our conclusion that *sc* plays a direct role in this regulatory axis. We believe these experiments address the reviewer's concern regarding reliance on previously published transcriptomic data and reinforce our conclusions.

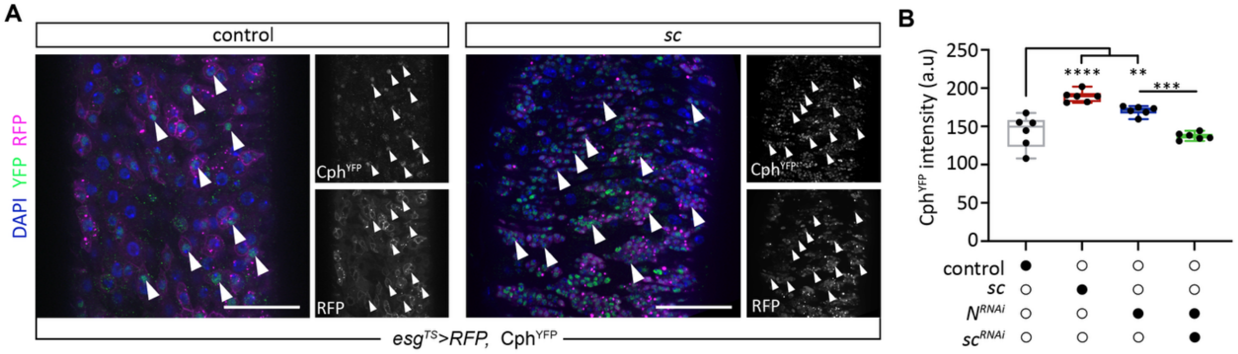

R-Fig. 10 – (A) Confocal images of the posterior midgut under steady state or progenitor-specific overexpression of *sc*. Scale bar 100 μm. (B) Quantification of Cph^{YFP} intensity in progenitor cells. Notice that overexpression of *sc* is sufficient to increase Cph^{YFP}, and its silencing in progenitor cell expressing *Notch*^{RNAi} reduces Cph^{YFP} intensity compared to *Notch*^{RNAi} only condition. Each dot represents a biological replicate.

6) Figure 6F: Multiple RNAi constructs for sff are necessary. Demonstrating the normal function of sff during homeostatic conditions would also be more relevant. Regarding NanoDam, does the GFP-recognizing nanobody also recognize YFP?

To address the role of *sff* under homeostatic conditions, we performed additional experiments using both the *esg*^{TS} and *esg*$^{F/O}$ systems to knock down *sff* during steady-state. These experiments revealed that *sff* knockdown (using two *RNAi* lines) does not alter the number of progenitor cells (*esg*^{TS}) or affect epithelial turnover (*esg*$^{F/O}$) (MS-Fig.7F-I/ Fig. EV8E-H). This result is consistent with our observations that *sff* is not expressed under homeostatic conditions, but becomes induced in progenitor cells upon Notch knockdown. Together, these findings support our conclusion that *sff* functions primarily in a Notch-deficient context, rather than in baseline homeostatic condition.

Regarding the reviewer's question about NanoDam specificity: yes, the nanobody used in the NanoDam system recognizes both GFP and YFP fusion proteins. This is well-documented in the

original NanoDam publication (PMID: 35483359), where YFP-tagged proteins were effectively used for Dam-Nanobody recruitment.

We would like to note that we have updated the display of our NanoDam track plots to show log2 fold change (Log2FC), rather than subtracting control NanoDam from Cph NanoDam. Following the approach described by Marshall & Brand (2015, PMID: 26112292), we recognized this as a more appropriate representation of the data. This adjustment does not alter our original findings, but only affects the scaling of the track plots.

7) Figure 7: The proposed negative feedback loop lacks experimental support and may involve post-translational regulation. In Figure 6, Cph is suggested to enhance gene expression, yet here it is proposed to suppress it. If the authors are positing such a dual role, they must provide a mechanistic explanation for this discrepancy. The claims regarding autophagy and cell death are unsubstantiated. The authors fail to provide evidence that autophagy or apoptosis occurs. They should be aware of the relevant literature on autophagy in ISCs (e.g., Nagy et al., 2018; Zhang et al., 2019) and acknowledge that ISCs are generally highly resistant to cell death. Furthermore, autophagic cell death is a rare phenomenon requiring substantial experimental validation, none of which is provided here.

We thank the reviewer for these valuable comments and for highlighting key literature related to autophagy and ISC biology. We agree that the proposed negative feedback loop and the roles of autophagy and cell death require clearer explanation and stronger experimental support.

1. On the proposed negative feedback loop of Cph:
In our manuscript, we observed that overexpression of *Cph* in progenitor cells leads to a reduction in both endogenous *Cph* transcript levels and Cph^{YFP} protein, suggesting a negative feedback mechanism (MS-Fig. 8B,C/EV9A). In support of this, our NanoDam data show that Cph binds to regions within its own gene locus, consistent with direct autoinhibition (MS-Fig.8A). While we cannot exclude the possibility of post-translational regulation, the observed reduction at the mRNA level indicates that at least part of this feedback occurs at the transcriptional level. We have now clarified this point in the revised manuscript, lines 545-549.

2. On Cph's dual regulatory roles (activation vs. repression):
We appreciate the reviewer's concern about the dual roles of *Cph* in gene regulation. As noted, we observed that *Cph* promotes the expression of many genes, including those involved in the cell cycle, while repressing others—including its own. In our original manuscript, we also show that Cph binds to its own locus in the brain during larval development, suggesting that autoregulation may span across development and tissues (MS-Fig. EV9B). This dual functionality is not without precedent. For example, the mammalian homolog of *Cph*, *BCL11b*, has been shown to act as both a transcriptional activator and repressor in lymphoid cells (PMID: 26231117). Specifically, it activates genes like Gata3, while repressing others, such as Ahr. The dual activating and repressing function of *Cph*-related transcription factors seem to be evolutionary conserved, allowing it to coordinate multiple processes. Another transcription factor, Hes1, is a well-known example of a transcriptional activator that also directly represses its own gene expression, while activating the expressing of other genes (PMIDs: 7906273, 1340473, 12399594). These findings are now discussed in our revised manuscript, lines 545-565.

3. On autophagy and cell death:

We performed additional experiments to investigate both autophagy and cell death more thoroughly, and have cited Nagy et al., 2018 and Zhang et al., 2019.

- **Autophagy:** Overexpression of *Cph* using the esg^{TS} system led to a significant increase in $Atg8a^+$ autophagosomes and $Lamp1^+$ lysosomes in progenitor cells, indicating upregulation of autophagy (R-Fig. 11/MS-Fig. 8E,F/ EV9E,F).

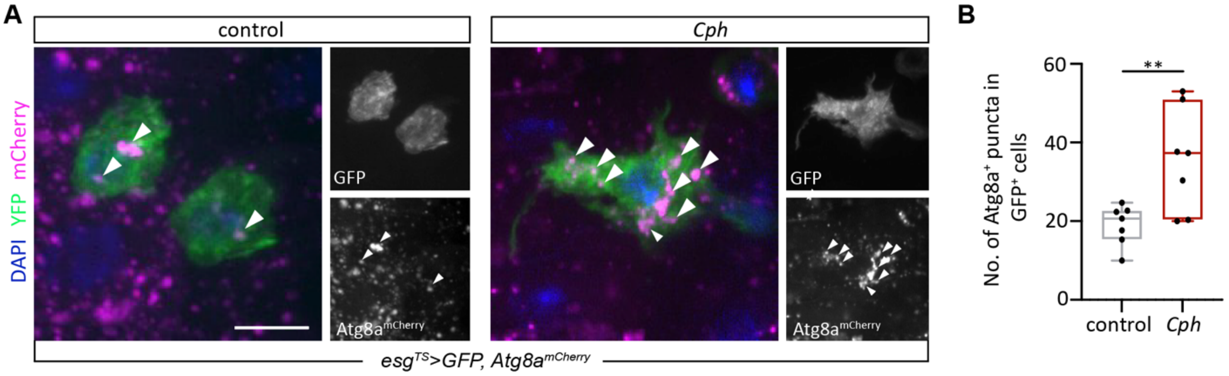

R-Fig. 11– **(A)** Confocal images of progenitor cells within the posterior midgut highlighting Atg8a+ autophagosomes during steady state and progenitor-specific *Cph* overexpression. Scale bar 10 µm. **(B)** Quantification of Atg8a+ puncta within GFP+ progenitor cells. Mann-Whitney test was used for statistical analysis.

- **Cell death:** Overexpression of *Cph* using the esg^{TS} system increased the number of apoptotic progenitor cells, as detected with the GC3Ai apoptotic sensor, indicating activation of apoptotic pathways (R-Fig. 12/MS-Fig. EV9G,H).

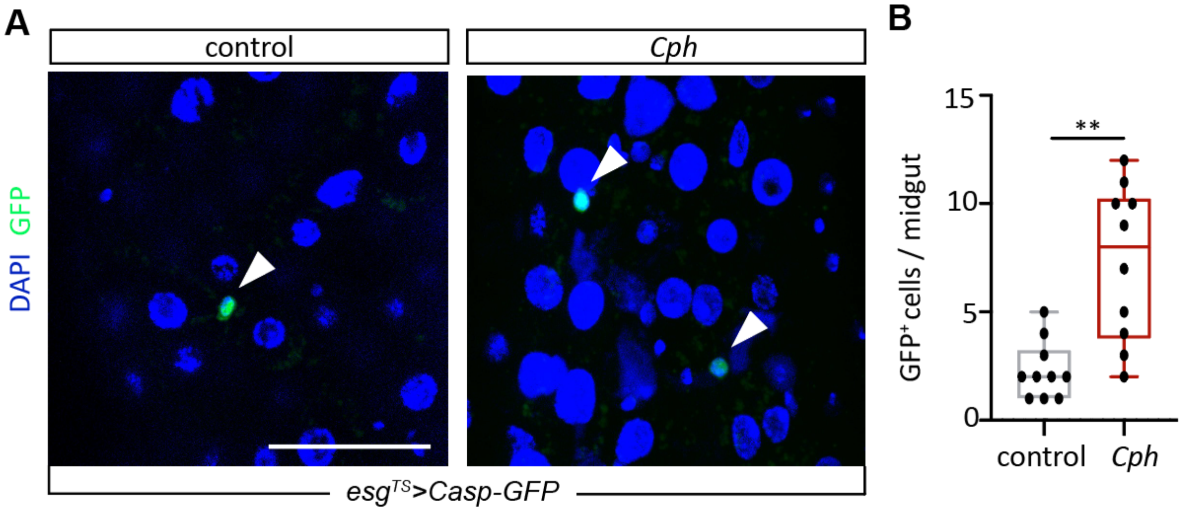

R-Fig.12 – **(A)** Visualisation of the apoptotic sensor (GC3Ai – Casp-GFP) expressed in progenitor cells. Images were taken from the posterior midgut. GFP marks apoptotic cells. Scale bar 10 µm. **(B)** Quantification of the number of GFP^+ apoptotic cells in the entire midgut. Mann-Whitney test was used for statistical analysis.

- **Functional relevance:** Blocking apoptosis in progenitor cells overexpressing *Cph* (e.g., through genetic inhibition of cell death by expression of the caspase inhibitor, *Diap1*) partially restored progenitor cell number, indicating that cell death contributes to the observed reduction in the progenitor populations overexpressing *Cph* (R-Fig. 13/MS-Fig. 8H/ EV9M).

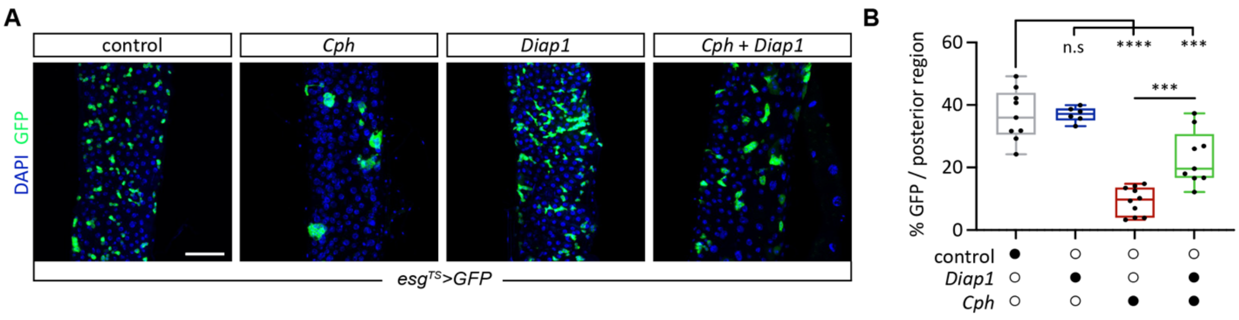

R-Fig.13 – **(A)** Confocal images of the posterior midgut under different genetic conditions. Progenitor cells are labelled with GFP. Scale bar 100 µm. **(B)** Quantification of the number of GFP^{+} progenitor cells. Notice that co-expression of *Cph* with *Diap1* partially restores the progenitor population compared to overexpression of only *Cph*. One-way ANOVA test with Tukey post hoc comparison was used for statistical analysis

Taken together, these findings demonstrate that *Cph* overexpression induces autophagy and triggers cell death, leading to a depletion of progenitor cells. These *in vivo* experiments complement our single cell RNA-sequencing analyses, which demonstrated that overexpression of *Cph* increases the expression of genes associated with autophagy and cell death. However, we agree with the reviewer that our data does not conclusively show autophagy-dependent cell death. In response, we have toned down our interpretation of our dataset in the revised manuscript and now discuss autophagy and apoptosis as co-occurring, see section - Auto-repression of Cph protects progenitor cells against autophagy and cell death.

Appeal - Editorial Decision 23rd Nov 2025

Dear Michael,

Thank you for submitting a revised version of your manuscript. I apologise for the slow process on our side due to the quality checks that we perform on revised manuscripts.

We have now received input from both original reviewers, who are broadly satisfied with the performed revisions and now request only textual revisions for precise and balanced interpretation of the results. There additionally remain only a few editorial points that need to be addressed before I can extend official acceptance of the manuscript:

1. Please submit a complete author checklist, which you can download from our author guidelines (https://media.springernature.com/original/springer-cms/rest/v1/content/27825796/data/v1). Please insert information in the checklist that is also reflected in the manuscript. The completed author checklist will also be part of the Review Process File.
2. Please upload the main figures as individual production quality figure files in the .eps, .tif,or .jpg format (one file per figure). Their legends should stay in the manuscript text, after the References.
3. The email message to the author Svenja Leible (svenja.leible@dkfz.de)was returned to the sender. Please check that this information is correct.
4. Please merge "Funding" section with "Acknowledgements". Please also enter the complete name of funder and project numbers and make sure that this Information is correct and identical both in the manuscript and our online system so that it can be directly linked in PubMed when the article is published.
5. CRediT has replaced the traditional author contributions section because it offers a systematic, machine-readable author contributions format that allows for more effective research assessment. Please remove the Authors Contributions from the manuscript and use the free text boxes beneath each contributing author's name in our online submission system to add specific details on the author's contribution. More information is available in our guide to authors.
6. Please rename "Declaration of interests" section into "Disclosure and competing interests statement".
7. Please remove the Reagents and Tools Table from the manuscript text file and leave as the separately uploaded file in our system.
8. Please update references according to The EMBO Journal style - where there are more than 10 authors on a paper, the first 10 should be listed, followed by 'et al.' Please remove DOIs from the reference list.
9. Please rename supplementary table into Table eV1 and update the callouts accordingly. Please add a short legend to the table.
10. Our data editors have flagged the following issues in figure legends that need correcting:
- Please note that the figure EV-8f appears to be mislabelled as figure EV-8g in the manuscript. Please check and correct as needed.
- Please provide the exact p values in the legends of figures 1c,e; 2e; 3b,d,e,f; 4a,c,e,g,h,k; 5d,g,h; 7d,e,g,h,l; 8c,f,h; EV-2c; EV-3c; EV-4e,f; EV-5b,d,h,i; EV-6b,c; EV-7a,b; EV-8g,h; EV-9a,f,h,j,l.
- Please indicate the statistical test used for data analysis in the legends of figures 4b; 6f; EV-2c; EV-3c,d; EV-7f,g,h.
- Please provide information on the number and nature of replicates in the legends of 1c,e; 2e,f; 3b,d,e; 4b,c,e,g,k; 5d,g,h; 7d,e,g-l; 8c,f,h; EV-1c; EV-2c; EV-3c; EV-4c,e,f; EV-5b,d,h,i; EV-6b,c; EV-7a,b,d,e; EV-8g,h; EV-9a,f,h,j,l.
- Please define the scale bar for figures 7f; EV-1d; EV-2c; EV-5f,g.
- Please define the white arrows and white arrow heads in the legend of figure 2d.
11. At EMBO Press we ask authors to provide source data for the main manuscript figures. You will receive a separate email with instructions for providing source data with your revised manuscript, including how to upload and organize the files.
12. In our standard image integrity check, we have noticed image reuse between figure panels 1D and 5F, as well as 5A and 7B. Please check - if this is intentional, please indicate the reuse in the figure legends.
13. Papers published in The EMBO Journal are accompanied online by a 'Synopsis' to enhance discoverability of the manuscript. It consists of A) a short (1-2 sentences) summary of the findings and their significance, B) 3-4 bullet points highlighting key results (the highlights can be repurposed for this) and C) a synopsis image that is 550x300-600 pixels large (width x height, jpeg or png format). You can either show a model or key data in the synopsis image. Please note that the image size is rather small and that text needs to be readable at the final size.
14. As part of the EMBO Press transparent editorial process, The EMBO Journal will publish online a Peer Review File to accompany accepted manuscripts. This file will be published in conjunction with your paper and will include the anonymous referee reports, your point-by-point response and all pertinent correspondence relating to the manuscript, including decision letters. Please note that the Author Checklist will be published at the end of the Peer Review File.
Please let us know if you want to remove or not any figures or data from the Peer Review File prior to publication. Please note that retaining unpublished data in the Peer Review File means that these count as published and that the Peer Review File would need to be referenced in future publications.

Please let me know if you have any questions regarding any of these points. You can use the link below to upload the revised files.

Thank you again for giving us the chance to consider your manuscript for The EMBO Journal. I look forward to receiving the final version.

With best wishes,

Ieva

Ieva Gailite, PhD
Senior Scientific Editor
The EMBO Journal
Meyerhofstrasse 1
D-69117 Heidelberg
Tel: +4962218891309
i.gailite@embojournal.org

Instructions for preparing your revised manuscript:

Read our guidance for manuscript revisions and related editorial policies: https://link.springer.com/journal/44318/submission-guidelines#cms-Revised-submissions

Please also check that the title and abstract of the manuscript are brief, yet explicit, even to non-specialists.

When assembling figures, please refer to our figure preparation guideline in order to ensure proper formatting and readability in print as well as on screen:
https://media.springernature.com/original/springer-cms/rest/v1/content/27825798/data/v1

IMPORTANT: When you send the revision we will require
- a point-by-point response to the referees' comments, with a detailed description of the changes made (as a word file).
- a word file of the manuscript text.
- individual production quality figure files (one file per figure)
- a complete author checklist
- Expanded View files (replacing Supplementary Information)
- a Reagents and Tools Table as part of the Methods section

Please remember: Digital image enhancement is acceptable practice, as long as it accurately represents the original data and conforms to community standards. If a figure has been subjected to significant electronic manipulation, this must be noted in the figure legend or in the 'Methods' section. The editors reserve the right to request original versions of figures and the original images that were used to assemble the figure.

We realize that it is difficult to revise to a specific deadline. In the interest of protecting the conceptual advance provided by the work, we recommend a revision within 3 months (21st Feb 2026). Please discuss the revision progress ahead of this time with the editor if you require more time to complete the revisions. Use the link below to submit your revision:

https://emboj.msubmit.net/cgi-bin/main.plex

Referee #1:

I thank the authors for their detailed responses and the considerable work undertaken in this revision. The new data have strengthened the manuscript, and I am satisfied with the responses to the majority of my points.

Regarding point #11 on the transcriptional analysis, I appreciate the authors' additional analysis of their scRNA-seq data. However, I find the interpretation of the EC data in R-Fig. 5 / MS-Fig. EV7D,E problematic. The conclusion that JAK-STAT/EGFR signaling "remained elevated" in ECs in the CphRNAi+NotchRNAi condition, and its use to argue for cell autonomy, seems to conflict with the established model from Patel et al. (2015). If CphRNAi suppresses the NotchRNAi-induced ISC hyperplasia, the consequent relief of EC stress and turnover should logically lead to a decrease, not a sustained increase, in the expression of damage-induced cytokines like Upd3. The presented data, showing minimal log2FC differences, do not convincingly resolve this issue.

I recommend removing this specific EC signaling analysis and the associated conclusion. This would prevent potential confusion and strengthen the manuscript's narrative flow.

The manuscript is otherwise much improved and suitable for publication.

Referee #2:

I only suggest changes that the authors can address through textual modification, but these revisions must be made with greater rigour and accuracy. Although I raised this issue previously, the section concerning autophagy and cell death remains misleading.

First, the authors should revise the part referring to autophagic cell death. Autophagic cell death is defined as cell death that does not involve caspase activation (see Bialik et al., Kimchi lab, J. Cell Sci., 2018).

Second, they should acknowledge that autophagosome accumulation does not necessarily indicate activation of autophagy; in fact, it often reflects inhibition of autophagic flux. For this reason, researchers in the autophagy field routinely use markers such as p62 or other assays to assess autophagy activity.

Third, regarding cell death, enteroblasts are generally prone to apoptosis (Reiff et al., EMBO J., 2019), whereas ISCs are relatively resistant (Sulekh et al., Life Sci Alliance, 2024). The authors use the term "progenitors" rather loosely, without clearly distinguishing between enteroblasts and ISCs. They should either define the term precisely or use it more cautiously.

General comment: We thank the reviewers for their positive feedback on our revised manuscript. Their thoughtful suggestions have substantially strengthened our study. We have provided a response to their remaining comments below.

Referee #1:

I thank the authors for their detailed responses and the considerable work undertaken in this revision. The new data have strengthened the manuscript, and I am satisfied with the responses to the majority of my points.

Regarding point #11 on the transcriptional analysis, I appreciate the authors' additional analysis of their scRNA-seq data. However, I find the interpretation of the EC data in R-Fig. 5 / MS-Fig. EV7D,E problematic. The conclusion that JAK-STAT/EGFR signaling "remained elevated" in ECs in the CphRNAi+NotchRNAi condition, and its use to argue for cell autonomy, seems to conflict with the established model from Patel et al. (2015). If CphRNAi suppresses the NotchRNAi-induced ISC hyperplasia, the consequent relief of EC stress and turnover should logically lead to a decrease, not a sustained increase, in the expression of damage-induced cytokines like Upd3. The presented data, showing minimal log2FC differences, do not convincingly resolve this issue.

I recommend removing this specific EC signaling analysis and the associated conclusion. This would prevent potential confusion and strengthen the manuscript's narrative flow.

The manuscript is otherwise much improved and suitable for publication.

Response: As the reviewer suggested, we have removed this data to avoid any potential confusion.

Referee #2:

I only suggest changes that the authors can address through textual modification, but these revisions must be made with greater rigour and accuracy. Although I raised this issue previously, the section concerning autophagy and cell death remains misleading.

First, the authors should revise the part referring to autophagic cell death. Autophagic cell death is defined as cell death that does not involve caspase activation (see Bialik et al., Kimchi lab, J. Cell Sci., 2018).

Response: All references to autophagic cell death have been removed from the text and Figure EV9D.

Second, they should acknowledge that autophagosome accumulation does not necessarily indicate activation of autophagy; in fact, it often reflects inhibition of autophagic flux. For this reason, researchers in the autophagy field routinely use markers such as p62 or other assays to assess autophagy activity.

Response: We thank the reviewer for highlighting this distinction. We have now changed the text accordingly (lines 495-500): “To assess autophagosome abundance, we quantified the

autophagosome marker Atg8a–mCherry (Nagy *et al*, 2016), which revealed a significant increase in the number of autophagosomes in *Cph*-overexpressing cells, consistent with the transcriptional elevation of autophagy-related genes (Fig. 8E,F). As increased autophagosome number can reflect either autophagy induction or reduced autophagosome clearance, we conclude that uncontrolled *Cph* expression elevates autophagosome abundance."

Third, regarding cell death, enteroblasts are generally prone to apoptosis (Reiff et al., EMBO J., 2019), whereas ISCs are relatively resistant (Sulekh et al., Life Sci Alliance, 2024). The authors use the term "progenitors" rather loosely, without clearly distinguishing between enteroblasts and ISCs. They should either define the term precisely or use it more cautiously.

Response: We thank the reviewer for highlighting these studies. Using our scRNA-seq we have confirmed that apoptotic and autophagy-related genes are upregulated in ISCs and not EBs when Cph is overexpressed using the *esg*^{TS} driver (Reviewer Figure 1 and Fig. EV9D). Consequently, we now made changes to the text and cited both studies, please see lines 501-520.

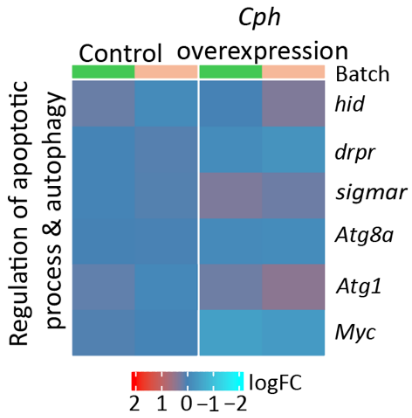

Reviewer Figure 1. Heatmap of cell death and autophagy related genes specifically in EBs across two independent scRNA-seq replicates in control and *Cph* overexpression conditions.

1st Revision - Editorial Decision 28th Apr 2026

Dear Michael,

Thank you for incorporating the final editorial and formatting requests in the manuscript. I apologise for the slow process from our side due to the large numbers of revised manuscripts that were recently submitted to our office. I am now pleased to inform you that your manuscript has been accepted for publication. Congratulations with a nice study!

Before we forward your manuscript to our publishers, we would like to propose some edits in the manuscript title, abstract and synopsis. I have also written a short blurb that will accompany the title of your manuscript in our online table of contents. Please take a look at the proposed text changes in the attached text file and let me know if any corrections are needed.

Your manuscript will be processed for publication by EMBO Press. It will be copy edited and you will receive page proofs prior to publication. Please note that you will be contacted by Springer Nature Author Services to complete licensing and payment information.

You may qualify for financial assistance for your publication charges - either via a Springer Nature fully open access agreement or an EMBO initiative. Check your eligibility: https://link.springer.com/journal/44318/how-to-publish-with-us

Should you be planning a Press Release on your article, please get in contact with embo_production@springernature.com as early as possible in order to coordinate publication and release dates.

I look forward to hearing from you about the final edits soon, after which we will be able to swiftly proceed with the transfer of your manuscript for typesetting. Thank you for this interesting contribution to The EMBO Journal!

Best wishes,

Ieva

Ieva Gailite, PhD
Senior Scientific Editor
The EMBO Journal
Meyerhofstrasse 1
D-69117 Heidelberg
Tel: +4962218891309
i.gailite@embojournal.org

*****
Please note that it is The EMBO Journal policy for the transcript of the editorial process (containing referee reports and your response letters) to be published as an online supplement to each paper. If you should prefer removal of any referee-only figures included in the point-by-point response(s), e.g. because they may still be used for future publication or because they have been reproduced from published work by others, please do let us know immediately via response email.
More information is available here: https://link.springer.com/partners/embo-press/editorial-policies#Peer%20review

** Click here to be directed to your login page: https://emboj.msubmit.net

EMBO Press Author Checklist

Corresponding Author Name: Michael Boutros, Wolfgang Huber, Siamak Redhai
Journal Submitted to: The EMBO Journal
Manuscript Number: EMBOJ-2024-119585R-Q

USEFUL LINKS FOR COMPLETING THIS FORM
The EMBO Journal - Author Guidelines
EMBO Reports - Author Guidelines
Molecular Systems Biology - Author Guidelines
EMBO Molecular Medicine - Author Guidelines

Reporting Checklist for Life Science Articles (updated January
This checklist is adapted from Materials Design Analysis Reporting (MDAR) Checklist for Authors. MDAR establishes a minimum set of requirements in transparent reporting in the life sciences (see Statement of Task: 10.31222/osf.io/9sm4x). Please follow the journal's guidelines in preparing your
Please note that a copy of this checklist will be published alongside your article.

Abridged guidelines for figures
1. Data
The data shown in figures should satisfy the following conditions:
- the data were obtained and processed according to the field's best practice and are presented to reflect the results of the experiments in an accurate and unbiased manner.
- ideally, figure panels should include only measurements that are directly comparable to each other and obtained with the same assay.
- plots include clearly labeled error bars for independent experiments and sample sizes. Unless justified, error bars should not be shown for technical
- if n<5, the individual data points from each experiment should be plotted. Any statistical test employed should be justified.
- Source Data should be included to report the data underlying figures according to the guidelines set out in the authorship guidelines on Data

2. Captions
Each figure caption should contain the following information, for each panel where they are relevant:
- a specification of the experimental system investigated (eg cell line, species name).
- the assay(s) and method(s) used to carry out the reported observations and measurements.
- an explicit mention of the biological and chemical entity(ies) that are being measured.
- an explicit mention of the biological and chemical entity(ies) that are altered/varied/perturbed in a controlled manner.
- the exact sample size (n) for each experimental group/condition, given as a number, not a range;
- a description of the sample collection allowing the reader to understand whether the samples represent technical or biological replicates (including how many animals, litters, cultures, etc.).
- a statement of how many times the experiment shown was independently replicated in the laboratory.
- definitions of statistical methods and measures:
  - common tests, such as t-test (please specify whether paired vs. unpaired), simple χ2 tests, Wilcoxon and Mann-Whitney tests, can be unambiguously identified by name only, but more complex techniques should be described in the methods section;
  - are tests one-sided or two-sided?
  - are there adjustments for multiple comparisons?
  - exact statistical test results, e.g., P values = x but not P values < x;
  - definition of 'center values' as median or average;
  - definition of error bars as s.d. or s.e.m.

Please complete ALL of the questions below.
Select "Not Applicable" only when the requested information is not relevant for your study.

Materials

Newly Created Materials	Information included in the manuscript?	In which section is the information available? (Reagents and Tools Table, Materials and Methods, Figures, Data Availability Section)
New materials and reagents need to be available; do any restrictions apply?	Not Applicable	

Antibodies	Information included in the manuscript?	In which section is the information available? (Reagents and Tools Table, Materials and Methods, Figures, Data Availability Section)
For **antibodies** provide the following information:<br>- Commercial antibodies: RRID (if possible) or supplier name, catalogue number and or/clone number<br>- Non-commercial: RRID or citation	Yes	Reagents and Tools Table

DNA and RNA sequences	Information included in the manuscript?	In which section is the information available? (Reagents and Tools Table, Materials and Methods, Figures, Data Availability Section)
**Short novel DNA or RNA including primers, probes**: provide the sequences.	Yes	Reagents and Tools Table

Cell materials	Information included in the manuscript?	In which section is the information available? (Reagents and Tools Table, Materials and Methods, Figures, Data Availability Section)
**Cell lines:** Provide species information, strain. Provide accession number in repository **OR** supplier name, catalog number, clone number, and/**OR** RRID.	Not Applicable	
**Primary cultures:** Provide species, strain, sex of origin, genetic modification status.	Not Applicable	
Report if the cell lines were recently **authenticated** (e.g., by STR profiling) and tested for mycoplasma contamination.	Not Applicable	

Experimental animals	Information included in the manuscript?	In which section is the information available? (Reagents and Tools Table, Materials and Methods, Figures, Data Availability Section)
**Laboratory animals or Model organisms:** Provide species, strain, sex, age, genetic modification status. Provide accession number in repository **OR** supplier name, catalog number, clone number, **OR** RRID.	Yes	Reagents and Tools/ Materials and Methods
**Animal observed in or captured from the field:** Provide species, sex, and age where possible.	Not Applicable	
Please detail **housing and husbandry conditions**.	Yes	Materials and Methods

Plants and microbes	Information included in the manuscript?	In which section is the information available? (Reagents and Tools Table, Materials and Methods, Figures, Data Availability Section)
**Plants:** provide species and strain, ecotype and cultivar where relevant, unique accession number if available, and source (including location for collected wild specimens).	Not Applicable	
**Microbes:** provide species and strain, unique accession number if available, and source.	Not Applicable	

Human research participants	Information included in the manuscript?	In which section is the information available? (Reagents and Tools Table, Materials and Methods, Figures, Data Availability Section)
If collected and within the bounds of privacy constraints report on age, sex and gender or ethnicity for all study participants.	Not Applicable	

Core facilities	Information included in the manuscript?	In which section is the information available? (Reagents and Tools Table, Materials and Methods, Figures, Data Availability Section)
If your work benefited from core facilities, was their service mentioned in the acknowledgments section?	Yes	Acknowledgments

Design

Study protocol	Information included in the manuscript?	In which section is the information available? (Reagents and Tools Table, Materials and Methods, Figures, Data Availability Section)
If study protocol has been **pre-registered, provide DOI in the manuscript**. For clinical trials, provide the trial registration number **OR** cite DOI.	Not Applicable	
Report the **clinical trial registration number** (at ClinicalTrials.gov or equivalent), where applicable.	Not Applicable	

Laboratory protocol	Information included in the manuscript?	In which section is the information available? (Reagents and Tools Table, Materials and Methods, Figures, Data Availability Section)
Provide DOI OR other citation details if **external detailed step-by-step protocols** are available.	Not Applicable	

Experimental study design and statistics	Information included in the manuscript?	In which section is the information available? (Reagents and Tools Table, Materials and Methods, Figures, Data Availability Section)
Include a statement about **sample size** estimate even if no statistical methods were used.	Yes	Materials and Methods/ Table eV1/ Figure legends
Were any steps taken to minimize the effects of subjective bias when allocating animals/samples to treatment (e.g. **randomization procedure**)? If yes, have they been described?	Not Applicable	
Include a statement about **blinding** even if no blinding was done.	Not Applicable	
Describe **inclusion/exclusion criteria** if samples or animals were excluded from the analysis. Were the criteria pre-established?<br><br>If sample or data points were omitted from analysis, report if this was due to attrition or intentional exclusion and provide justification.	Not Applicable	
For every figure, are **statistical tests** justified as appropriate? Do the data meet the assumptions of the tests (e.g., normal distribution)? Describe any methods used to assess it. Is there an estimate of variation within each group of data? Is the variance similar between the groups that are being statistically compared?	Yes	Materials and Methods/ Figure legends

Sample definition and in-laboratory replication	Information included in the manuscript?	In which section is the information available? (Reagents and Tools Table, Materials and Methods, Figures, Data Availability Section)
In the figure legends: state number of times the experiment was **replicated** in laboratory.	Yes	Figure legend
In the figure legends: define whether data describe **technical or biological replicates**.	Yes	Figure legend

Ethics

Ethics	Information included in the manuscript?	In which section is the information available? (Reagents and Tools Table, Materials and Methods, Figures, Data Availability Section)
Studies involving **human participants**: State details of **authority granting ethics approval** (IRB or equivalent committee(s), provide reference number for approval.	Not Applicable	
Studies involving **human participants**: Include a statement confirming that **informed consent** was obtained from all subjects and that the experiments conformed to the principles set out in the WMA Declaration of Helsinki and the Department of Health and Human Services Belmont Report.	Not Applicable	
Studies involving **human participants:** For publication of **patient photos**, include a statement confirming that consent to publish was obtained.	Not Applicable	
Studies involving experimental **animals**: State details of **authority granting ethics approval** (IRB or equivalent committee(s), provide reference number for approval. Include a statement of compliance with ethical regulations.	Not Applicable	
Studies involving **specimen and field samples:** State if relevant **permits** obtained, provide details of authority approving study; if none were required, explain why.	Not Applicable	

Dual Use Research of Concern (DURC)	Information included in the manuscript?	In which section is the information available? (Reagents and Tools Table, Materials and Methods, Figures, Data Availability Section)
Could your study fall under dual use research restrictions? Please check biosecurity documents and list of **select agents and toxins** (CDC): https://www.selectagents.gov/sat/list.htm	Not Applicable	
If you used a select agent, is the security level of the lab appropriate and reported in the manuscript?	Not Applicable	
If a study is subject to dual use research of concern regulations, is the name of the **authority granting approval and reference number** for the regulatory approval provided in the manuscript?	Not Applicable	

Reporting

The MDAR framework recommends adoption of discipline-specific guidelines, established and endorsed through community initiatives. Journals have their own policy about requiring specific guidelines and recommendations to complement MDAR.

Adherence to community standards	Information included in the manuscript?	In which section is the information available? (Reagents and Tools Table, Materials and Methods, Figures, Data Availability Section)
State if relevant guidelines or checklists (e.g., **ICMJE, MIBBI, ARRIVE, PRISMA**) have been followed or provided.	Not Applicable	
For **tumor marker prognostic studies,** we recommend that you follow the **REMARK** reporting guidelines (see link list at top right). See author guidelines, under 'Reporting Guidelines'. Please confirm you have followed these guidelines.	Not Applicable	
For **phase II and III randomized controlled trials**, please refer to the **CONSORT** flow diagram (see link list at top right) and submit the CONSORT checklist (see link list at top right) with your submission. See author guidelines, under 'Reporting Guidelines'. Please confirm you have submitted this list.	Not Applicable	

Data Availability

Data availability	Information included in the manuscript?	In which section is the information available? (Reagents and Tools Table, Materials and Methods, Figures, Data Availability Section)
Have **primary datasets** been deposited according to the journal's guidelines (see 'Data Deposition' section) and the respective accession numbers provided in the Data Availability Section?	Yes	Materials and Methods/ Reagents and Tools
Were **human clinical and genomic datasets** deposited in a public access-controlled repository in accordance to ethical obligations to the patients and to the applicable consent agreement?	Not Applicable	
Are **computational models** that are central and integral to a study available without restrictions in a machine-readable form? Were the relevant accession numbers or links provided?	Not Applicable	
If publicly available data were reused, provide the respective **data citations in the reference list**.	Yes	Main body/ Reference list
